# Rh(II)/Pd(0) Dual Catalysis: Carbenoid N-H Insertion/Allylation Cascade Reaction to Construct Highly Functionalized and Polysubstituted Pyrrolidines

**DOI:** 10.3390/molecules29245880

**Published:** 2024-12-13

**Authors:** Maocheng Tang, Xianyan Jiao, Deping He, Ji-Xing Zhao, Ping Liu, Chun-Tian Li

**Affiliations:** 1 State Key Laboratory Incubation Base for Green Processing of Chemical Engineering, School of Chemistry and Chemical Engneering, Shihezi University, Shihezi 832003, China; 17830339005@163.com (M.T.); 15348654318@163.com (X.J.); 15519299129@163.com (D.H.); 2 Analysis and Testing Center, Shihezi University, Shihezi 832003, China; zhaojixing_2016@126.com

**Keywords:** pyrrolidines, carbenoid N-H insertion, allylation, Rh(II)/Pd(0) dual catalysis, high diastereoselectivities

## Abstract

In the category of drugs approved by the U.S. FDA, pyrrolidine is the most frequently used core of five-membered nonaromatic heterocycles containing nitrogen. Herein, a Rh(II)/Pd(0) dual-catalyzed carbenoid N-H insertion/allylation cascade reaction has been developed. This protocol provide an efficient approach for the construction of diverse highly functionalized and polysubstituted pyrrolidines in high yields (up to 91%) with excellent chemoselectivities and high diastereoselectivities (>20:1) under mild reaction conditions.

## 1. Introduction

**Figure 1:**
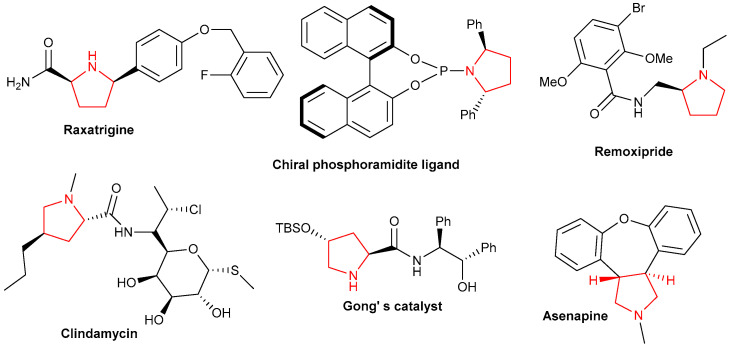
Functionalized compounds with pyrrolidine core structure.

Polysubstituted pyrrolidines are a critically important class of molecules found in pharmaceutical agents, natural products, and as chiral elements in asymmetric catalysis (Figure 1) [1,2,3,4,5,6,7]. In the category of drugs approved by the U.S. FDA, pyrrolidine is the most frequently used core of five-membered nonaromatic heterocycles containing nitrogen. Among them, a vast majority (92%) of pyrrolidine drugs are substituted at the N1-position and more than half (62%) are substituted at the C2 position [8]. Therefore, the development of new synthetic methods to construct polysubstituted pyrrolidines containing both N1 and C2 substitutions is expected to develop promising drug molecules.

**Figure 2:**
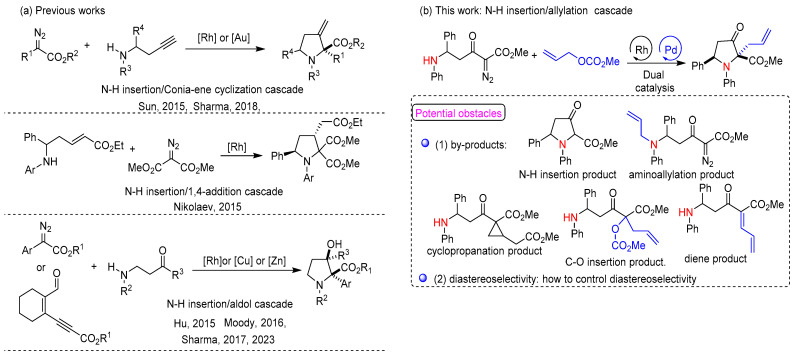
N-H insertion cascade reactions of metallocarbenes to synthesis of polysubstituted pyrrolidines [29,31,32,33,35,36,39].

Many impressive strategies have been developed for the synthesis of pyrrolidines [9,10,11], including azomethine ylide cycloaddition [12], alkene hydroamination [13,14,15], iodocyclization [16], rearrangement [17,18,19,20,21] intramolecular carbenes insertion into N-H [22,23,24,25,26] or C-H bonds [27,28], N-H insertion cascade reactions of metallocarbenes [29,30,31,32,33,34,35,36], and cycloisomerization [37,38]. Among them, N-H insertion cascade reactions of metallocarbenes represent powerful tools enabling quick and efficient access to highly functionalized and polysubstituted pyrrolidines in an atom-economical fashion. For example, Sun and Sharma groups used diazo-derived metallocarbenes for a diverted N–H insertion/Conia-ene cyclization cascade to access highly functionalized pyrrolidines (Figure 2a) [33,36]. Nikolaev group utilized N–H insertion/1,4-addition cascade to construct polysubstituted N-aryl pyrrolidines [29]. Hu, Moody and Sharma groups applied a cascade process involving N–H insertion/aldol cascade to access polysubstituted pyrrolidines [31,32,35,39]. The development of new N-H insertion cascade reactions for metallocarbenes to construct highly functionalized and polysubstituted pyrrolidines has always been a goal pursued by organic chemists.

The reaction comprises several active intermediates, including metallocarbenes, ammonium ylides, and allyl cations. As a result, several byproducts may form from these intermediates: an N−H insertion product resulting from a 1,2-H shift of ammonium ylides [40,41,42], metalcarbenes cyclopropylation product [43], a product resulting from allylic ester C−O bond insertion into a diazo compound [44], amine allylation product of diazo compound [45]. and diene product [46]. In addition, two stereocenters will be generated during the reaction process, and how to obtain the target product with high diastereoselectivity is also a challenge that needs to be overcome in this study. Therefore, achieving selective formation of highly functionalized and polysubstituted pyrrolidines with high chemoselectivity and diastereoselectivity through the Rh/Pd dual-catalyzed cascade process involving N–H insertion/allylation is a formidable challenge. Herein, we report our efforts to address above issues and obtain the highly functionalized and polysubstituted pyrrolidines with high diastereoselectivity, high chemoselectivity, and broad substrate scope under mild reaction conditions (Figure 2b).

## 2. Results and Discussion

To test our hypothesis, we first chose methyl 2-diazo-3-oxo-5-phenyl-5-(phenylamino)pentanoate **1a** (0.15 mmol) and allyl carbonate **2** (0.25 mmol) as model substrates for the reaction (Table 1). Rh_2_(OAc)_4_, Pd(dba)_2_, ligand X-phos, and **2** were stirred for 15 min at room temperature in 1 mL dichloromethane. **1a** dissolved in 1 mL of dichloromethane was slowly added to the reaction system dropwise. Fortunately, the desired pyrroline product **3a** was obtained in 87% yield as a single diastereoisomer (d.r. > 20:1) (entry 1). Subsequently, different ligands were screened, such as BINAP, Xantphos, DPPF, and TFP, revealing that X-phos was preferred (entries 2–5). Furthermore, various Rh complexes, Pd complexes, and solvents were screened, indicating that Rh_2_(OAc)_4_, Pd_2_(dba)_3_, and DCM were the best combination (entries 6), affording **3a** in 91% yield as a single diastereoisomer (for details, see Table S1). In a preliminary investigation of enantioselective conditions, chiral palladium complexes or mixtures of chiral phosphonic acid and chiral palladium complexes were applied without satisfactory results (for details, see Table S2).

**Scheme 1:**
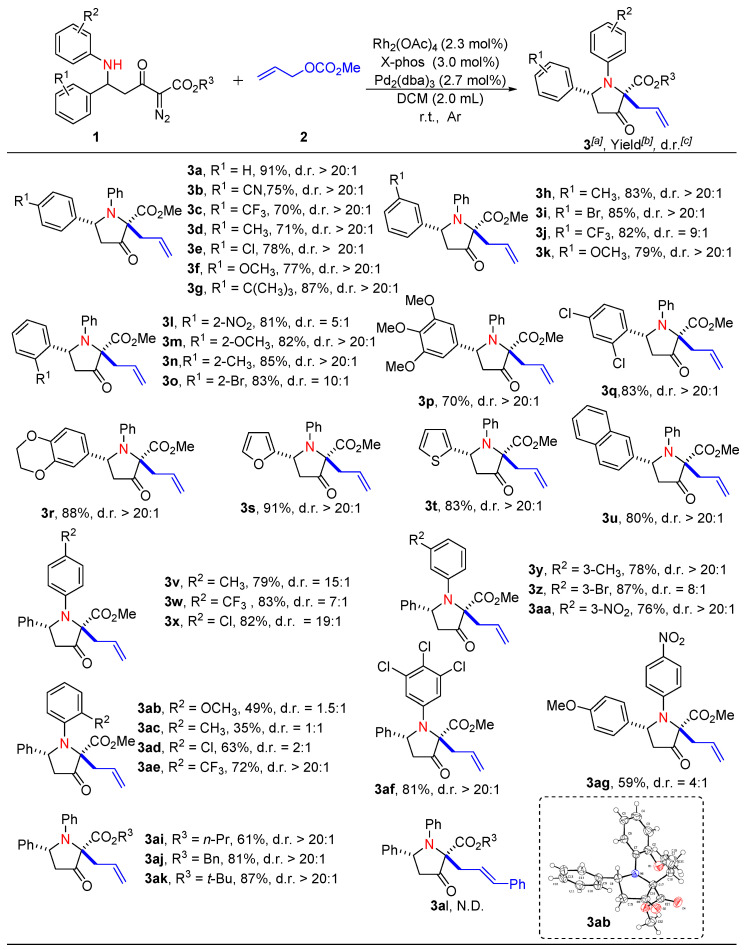
Diazo compounds 1, substrate scope. [a] For reaction conditions, see entry 6 of Table 1; [b] Isolated yield; [c] Diastereoselectivities were determined by 1H NMR spectroscopy of the crude reaction mixture; N.D. = not detected; d.r. = diastereomeric ratio.

With optimal conditions in hand (Table 1, entry 6), the scope of the 5-aryl moiety of diazo compound **1** was first explored (Scheme 1). 5-aryl moiety with electron-donating groups and electron-deficient groups at 4-position and 3-position of benzene ring underwent the reaction smoothly, affording the corresponding products **3b**–**3k** in 70–87% yields with good diastereoselectivities. The benzene ring substituted by electron-donating and electron-deficient groups at 2-position with high steric hindrance is also suitable for this reaction, and the target product **3l**–**3o** is obtained in 81–85% yields with moderate to good diastereoselectivities (5:1 to greater than 20:1). The multiple substituents on the 5-aryl moiety were well tolerated, giving the desired cyclization products in excellent yields with good diastereoselectivities (**3p**–**3r**). Transforming the benzene ring into other aromatic rings, such as furan, thiophene, and naphthalene rings, resulted in corresponding target products with excellent yields and diastereoselectivities (**3s**–**3u**).

Next, the scope of the N-aryl moiety of diazocompound **1** was explored. Similar to the 5-aryl moiety, the 4-position and 3-position of benzene ring exhibit good substrate tolerance when replaced by electron-donating and electron-withdrawing groups, respectively, resulting in a yield of 76–87% for the products **3v**–**3aa** with good diastereoselectivities. It is worth noting that the substituents at the 2-position of the N-aryl significantly affected the outcomes of the reaction: the methoxy, methyl, and chloride substituents gave lower diastereoselectivities (**3ab**–**3ad**), while the trifluoromethyl substituent afforded excellent diastereoselectivities (**3ae**). Substrate **1af** substituted with 3,4,5-trichloro obtained product **3af** with a yield of 81% and diastereoselectivities greater than 20:1. In addition, methyl 2-diazo-5-(4-methoxyphenyl)-5-(4 nitrophenyl)amino)-3-oxopentanoate **1ag** obtained product **3ag** with a yield of 59% and a diastereoselectivities of 4:1. Finally, we investigated the scope of the ester moiety of diazo compounds **1** and the phenyl allylation reagent. The results are as follows: *n*-propyl, benzyl, *t*-butyl esters were well tolerated, affording the corresponding products **3ai**–**3ak** in 61–87% yields with good diastereoselectivities. However, the phenyl allylation reagent failed to give desired product **3al** meso-positions as reactants, only monosubstituted 4-sulfenylated products **4k**–**4m** could be obtained, and we speculate that this may be mainly due to the electronic effect of the substituent groups.

**Scheme 2:**
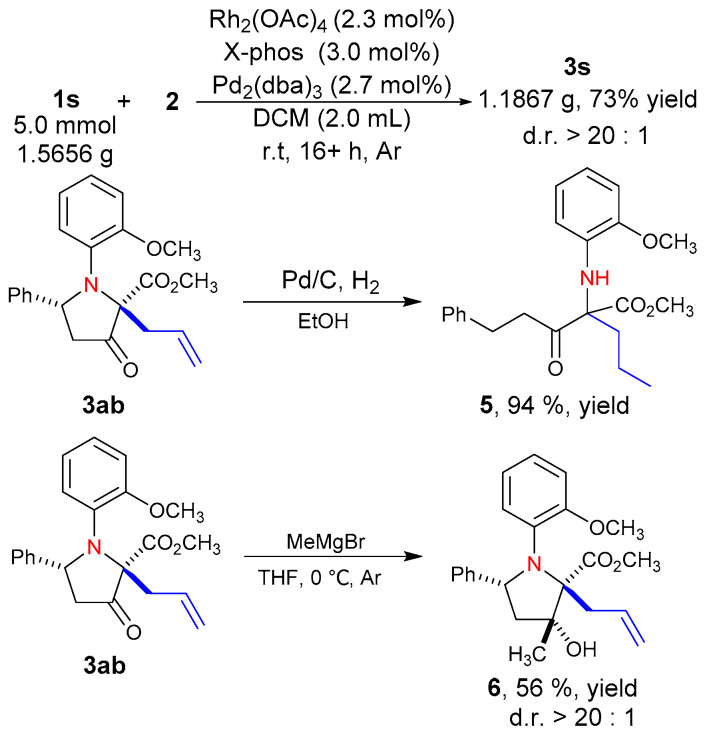
Application to large-scale synthesis and synthetic transformation.

To illustrate the synthetic utility of the reaction, a 5.0 mmol-scale reaction of **1s** and **2** was implemented under the optimal conditions, producing the **3s** in 73% yield (1.19 g) as a single diastereoisomer (d.r. > 20:1). Subsequently, synthetic transformations of **3ab** were performed (Scheme 2). Reduction of **3ab** with H_2_ gave the reduction/ring opening target product **5** in 94% yields. Methylmagnesium bromide undergoes a nucleophilic addition reaction with the carbonyl group of product **3ab**, affording desired product **6** in 56% yield with a d.r. value greater than 20:1.

**Scheme 3:**
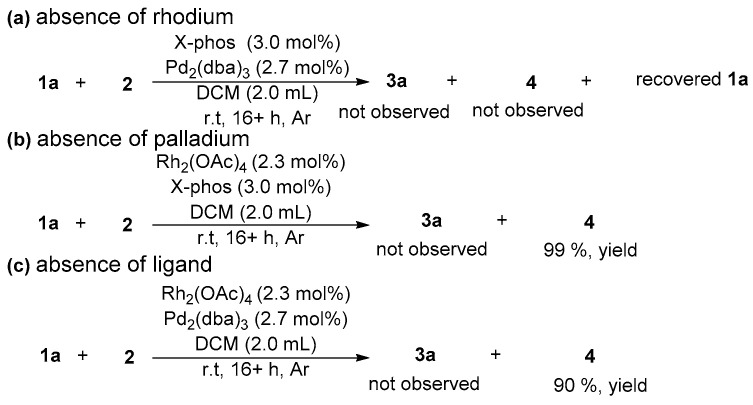
Mechanistic control experiments.

To gain insight into the mechanism of the reaction, control experiments were conducted (Scheme 3). First, the model reactions of **1a** with **2** in the absence of Rh_2_(OAc)_4_, Pd-complex, or ligand X-phos, were conducted, giving no product **3a**. Whereas, intramolecular N-H insertion product **4** was obtained in 99% and 90% yields, respectively, when the reaction was performed without Pd-complex, or ligand X-phos. The results indicated that Rh_2_(OAc)_4_, Pd_2_(dba)_3,_ and ligand X-phos were all necessary for successful reaction. Then, the control reaction of **4** and **2** was performed under standard conditions, and no conversions of **4** were performed. This observation suggests that byproduct **4** is not intermediates in this reaction, and a stepwise reaction pathway is unlikely.

**Figure 3:**
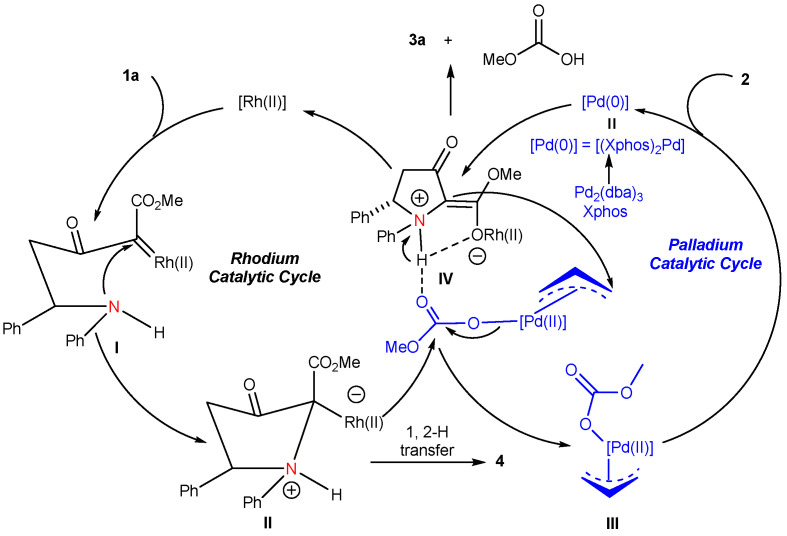
Plausible mechanism and preferential conformation for transition states.

Based on the above experimental results and literature precedent [23,25,31,32,47,48], a plausible mechanism is proposed, as shown in Figure 3. In the rhodium catalytic cycle, intramolecular nucleophilic attack of the amino group to the carbon atom of the Rh(II)-carbenoid may through the five membered cyclic transition state **I**, in which the carbomethoxy group at C-2 is forced into the axial position by the metal, leading to the carbomethoxy group at C-2 and the phenyl group at C-5 located on the same side. Subsequently, Rh(II)-associated ammonium ylide species **II** was formed. A 1,2-H shift could occur directly to produce the corresponding N–H insertion byproduct **4** via this ylide species. In the palladium catalytic cycle, Pd_2_(dba)_3_ undergoes ligand exchange with Xphos to produce a more active [(Xphos)_2_Pd]. In addition, [(Xphos)_2_Pd] has a higher electron density, which can promote the rate of oxidation addition of [Pd(0)] with allyl carbonate **2** to generate π-allyl−palladium intermediate **III**. Ylide species **II** can react with π-allyl−palladium intermediate **III** to generate **IV**, in which hydrogen bonding may be in existence between the proton hydrogen in ammonium ylide and the carbonyl oxygen in ester groups or the oxygen atoms in rhodium-associated enol ester. The existence of hydrogen bonds has three functions: (1) Shorten the distance between intermediates **II** and **III** and accelerate the reaction rate. (2) Maintain a high *Z*/*E* ratio in intermediate **IV**, which is the key to achieving high diastereoselectivity in the reaction. (3) Helps in inhibiting the undesired N-H insertion through 1,2-proton transfer. Furthermore, the size of the nitrogen phenyl group may also affect the *Z*/*E* ratio of intermediate **IV**, and a larger steric hindrance may reduce the *Z*/*E* ratio of intermediate **IV**, thereby reducing the diastereoselectivity of the reaction. This may be one of the reasons why products **3ab–3ad** exhibit lower diastereoselectivity.

## 3. Materials and Methods

**Scheme 4:**
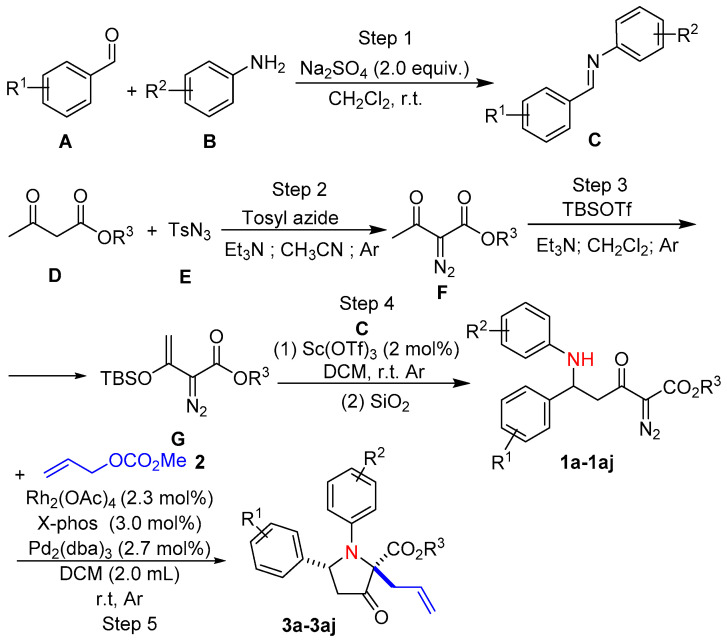
Substrate synthesis method.

Most reactions are performed in an argon atmosphere and in a dry glass instrument with magnetic stirring using standard Schlenk technology. Most of the ultra-dry solvents are purchased directly, except for CCl_4_ and CHCl_3_, which are purified by the purification manual. The reaction products were purified by rapid column chromatography using 200–300 mesh silica gel. Visualization of TLC (thin layer chromatography) is achieved by using ultraviolet light (254 nm) and invading the plate into a phosphomolybdenum solution and developing color with a hair dryer baking the plate. High-resolution mass spectrometry (HRMS) was measured on Thermofishek Eksaket Herms. The melting point (m.p.) was measured using the WRS-1B melting point meter. Proton and carbon magnetic resonance spectra (^1^H NMR and ^13^C NMR) were recorded as measured by Bruker AVANCE III HD 400 (^1^H NMR at 400 MHz and ^13^C NMR at 100 MHz) with CDCl_3_ solvent as the internal standard (^1^H NMR: 7.26 ppm for CDCl_3_ and 77.2 ppm for ^13^C NMR:CDCl_3_). The ^1^H NMR data are reported as follows: chemical shift, (s = single peak, d = double peak, t = triple peak, q = quadruple peak, m = multistate, br = broad peaks), coupling constant (Hz), integral. Known diazo compounds of **1a, 1e, 1t, 1ag** and **1aj** were prepared by known methods [49,50]. The method of preparing unknown diazo substrates is as follows (Scheme 4).

Step1: To a solution of aniline **B** (5.0 mmol) in dry CH_2_Cl_2_ (7 mL) was added sodium sulfate (4.0 equiv.), followed by aldehyde **A** (5.0 mmol) at room temperature. The resultant mixture was stirred for 16 h at room temperature. The reaction mixture was filtered and concentrated to afford the crude residue imine **C** for the next step without further purification [51].

Step2: Under an argon atmosphere, to a 250 mL oven-dried round bottom flask containing a magnetic stirring bar, acetylacetate **D** (34.0 mmol) and 4-methylbenzenesulfonyl azide **E** (47.6 mmol) in MeCN (120 mL) were added Et_3_N (51.0 mmol) slowly at 0 °C. After the reaction mixture was stirred for 12 h, H_2_O (10 mL) and DCM (20 mL) were added. The organic phase was separated and washed saturated aqueous NaCl (15 mL) and dried with anhydrous Na_2_SO_4_. After evaporating the solvents, diazo compounds **F** were purified by flash chromatography (petroleum ether/ethyl acetate =20:1) [52].

Step3: Under an argon atmosphere, to a 250 mL oven-dried round bottom flask containing a magnetic stirring bar, **F** (20.0 mmol) and Et_3_N (30.0 mmol) in DCM (90 mL) were added TBSOTf (22.0 mmol) slowly at 0 °C. After the reaction mixture was stirred for 12 h under these conditions, saturated aqueous NaHCO_3_ (15 mL) were added. The organic phase was separated and washed two more times with saturated aqueous NaHCO_3_ (15 mL) and dried with anhydrous Na_2_SO_4_. The reaction mixture was filtered and concentrated to afford the crude product **G** for the next step without further purification [52].

Step4: To a dry 250 mL round-bottomed flask containing a stirring bar and fitted with a septum was added anhydrous scandium (III) triflate (154.4 mg, 0.31 mmol) in anhydrous dichloromethane (70 mL). **G** (6.0 g, 0.023 mol) was then added dropwise over 5 min via a 10 mL syringe. Finally, imine **C** (2.8420 g, 0.0157 mol) in CH_2_Cl_2_ (5 mL) was added dropwise over 5 min to the reaction mixture. The reaction mixture was stirred at room temperature for 16 h. The colour of solution changed from orange to bright yellow and was subsequently passed through a silica gel plug with washings of CH_2_Cl_2_ to remove the catalyst. Solvent was removed under reduced pressure. The TBS group was deprotected by stirring the solution of the Mannich adduct in methanol (20 mL) and silica gel for 20 min at ambient temperature. Product was isolated by flash column chromatography eluting with petroleum ether/ethyl acetate (20:1), get products **1a**–**1aj** [25].

Step5: Allyl methyl carbonate **2** (29.0 mg, 0.25 mmol, 1.7 equiv.), Rh_2_(OAc)_4_ (1.5 mg, 2.3 mol%) X-phos (2.1 mg, 3.0 mol %), Pd_2_(dba)_3_ (3.7 mg, 2.7 mol%) were loaded into a drying tube. After three times of ventilation with argon, 1 mL of CH_2_Cl_2_ solution was added and stirred at room temperature for 15 min. The diazo **1** (48.7 mg, 0.15 mmol, 1.0 equiv.) was dissolved in 1 mL of CH_2_Cl_2_ and slowly added to the reaction solution. The resulting mixture was maintained at room temperature until the absence of starting material via TLC analysis (about 16 h) and quenched with saturated NaCl (5 mL). The aqueous layer was extracted with EtOAc (3 × 5 mL). The combined organic layers were dried over Na_2_SO_4_ and concentrated in vacuo. The residue was purified by chromatography (eluent: petroleum ether/ethyl acetate = 20/1) to afford product **3a**.

*Methyl 5-(4-cyanophenyl)-2-diazo-3-oxo-5-(phenylamino)pentanoate* **1b**. The title compound was prepared according to the general procedure using the corresponding imine (2.0 mmol, 1.0 equiv.), **G** (3.0 mmol, 1.5 equiv.), Sc(OTf)_3_ (0.01 g, 2.0 mol%). Column chromatography (petroleum/ethyl acetate = 20:1) afforded 0.1601 g (23%) of **1b** as a colorless oil. Analytical data for **1b**: ^1^H NMR (400 MHz, CDCl_3_) *δ* 7.63–7.57 (m, 4H), 7.12–7.07 (m, 2H), 6.70 (t, *J* = 7.2 Hz, 1H), 6.47 (d, *J* = 7.6 Hz, 2H), 5.09 (s, 1H), 4.91 (dd, *J* = 9.4, 4.2 Hz, 1H), 3.87 (s, 3H), 3.43 (dd, *J* = 14.4, 4.4 Hz, 1H), 3.21 (dd, *J* = 14.4, 9.2 Hz, 1H). ^13^C NMR (100 MHz, CDCl_3_) *δ* 189.9, 161.9, 148.2, 146.0, 132.8, 129.4, 127.4, 118.9, 118.6, 113.9, 111.4, 55.1, 52.6, 47.1. HRMS (ESI-TOF) *m/z*: [M + H]^+^ calcd for C_19_H_17_N_4_O_3_^+^ 349.1295; found 349.1291.
*Methyl 2-diazo-3-oxo-5-(phenylamino)-5-(4-(trifluoromethyl)phenyl)pentanoate* **1c**. The title compound was prepared according to the general procedure using the corresponding imine (2.0 mmol, 1.0 equiv.), **G** (3.0 mmol, 1.5 equiv.), Sc(OTf)_3_ (0.01 g, 2.0 mol%). Column chromatography (petroleum/ethyl acetate = 20:1) afforded 0.3050 g (39%) of **1c** as acolorless oil. Analytical data for **1c**: ^1^H NMR (400 MHz, CDCl_3_) *δ* 7.59 (s, 4H), 7.12–7.07 (m, 2H), 6.69 (t, *J* = 7.4 Hz, 1H), 6.50 (d, *J* = 7.6 Hz, 2H), 4.94 (dd, *J* = 9.4, 4.2 Hz, 1H), 3.87 (s, 3H), 3.43 (dd, *J* = 14.4, 4.0 Hz, 1H), 3.25 (dd, *J* = 14.4, 9.6 Hz, 1H). ^13^C NMR (100 MHz, CDCl_3_) *δ* 190.2, 161.9, 146.8, 146.3, 129.7 (q, *J*_C–F_ = 32.2 Hz), 129.3, 126.9, 125.9 (q, *J*_C–F_ = 3.7 Hz), 124.3 (d, *J*_C–F_ = 270.3 Hz) 118.3, 113.9, 55.0, 52.6, 47.4. HRMS (ESI-TOF) *m/z*: [M + H]^+^ calcd for C_19_H_17_F_3_N_3_O_3_^+^ 392.1217; found 392.1213.
*Methyl 2-diazo-3-oxo-5-(phenylamino)-5-(p-tolyl)pentanoate* **1d**. The title compound was prepared according to the general procedure using the corresponding imine (2.0 mmol, 1.0 equiv.), **G** (3.0 mmol, 1.5 equiv.), Sc(OTf)_3_ (0.01 g, 2.0 mol%). Column chromatography (petroleum/ethyl acetate = 20:1) afforded 0.1349 g (20%) of **1d** as a colorless oil. Analytical data for **1d**: ^1^H NMR (400 MHz, CDCl_3_) *δ* 7.34 (d, *J* = 8.0 Hz, 2H), 7.15–7.06 (m, 4H), 6.66 (t, *J* = 7.4 Hz, 1H), 6.55 (d, *J* = 7.6 Hz, 2H), 4.88 (dd, *J* = 9.2, 4.4 Hz, 1H), 3.87 (s, 3H), 3.40 (dd, *J* = 14.4, 4.8 Hz, 1H), 3.28 (dd, *J* = 14.2, 9.4 Hz, 1H), 2.32 (s, 3H). ^13^C NMR (100 MHz, CDCl_3_) *δ* 190.8, 162.0, 146.7, 139.5, 137.0, 129.5, 129.2, 126.4, 117.9, 113.9, 55.0, 52.5, 47.6, 21.2. HRMS (ESI-TOF) *m/z*: [M + H]^+^ calcd for C_19_H_20_N_3_O_3_^+^ 338.1499; found 338.1496.
*Methyl 2-diazo-5-(4-methoxyphenyl)-3-oxo-5-(phenylamino)pentanoate* **1f**. The title compound was prepared according to the general procedure using the corresponding imine (2.0 mmol, 1.0 equiv.), **G** (3.0 mmol, 1.5 equiv.), Sc(OTf)_3_ (0.01 g, 2.0 mol%). Column chromatography (petroleum/ethyl acetate = 20:1) afforded 0.1766 g (25%) of **1f** as a colorless oil. Analytical data for **1f**: ^1^H NMR (400 MHz, CDCl_3_) *δ* 7.36 (d, *J* = 8.8 Hz, 2H), 7.11–7.06 (m, 2H), 6.84 (d, *J* = 8.8 Hz, 2H), 6.67 (t, *J* = 7.2 Hz, 1H), 6.56 (d, *J* = 8.0 Hz, 2H), 4.85 (dd, *J* = 9.2, 4.8 Hz, 1H), 3.86 (s, 3H), 3.77 (s, 3H), 3.42 (dd, *J* = 14.4, 4.8 Hz, 1H), 3.29 (dd, *J* = 14.0, 9.6 Hz, 1H). ^13^C NMR (100 MHz, CDCl_3_) *δ* 190.8, 162.0, 158.9, 146.5, 134.4, 129.2, 127.6, 118.1, 114.2, 114.1, 55.4, 54.9, 52.5, 47.6. HRMS (ESI-TOF) *m/z*: [M + H]^+^ calcd for C_19_H_20_N_3_O_4_^+^ 354.1448; found 354.1444.
*Methyl 5-(4-(tert-butyl)phenyl)-2-diazo-3-oxo-5-(phenylamino)pentanoate* **1g**. The title compound was prepared according to the general procedure using the corresponding imine (2.0 mmol, 1.0 equiv.), **G** (3.0 mmol, 1.5 equiv.), Sc(OTf)_3_ (0.01 g, 2.0 mol%). Column chromatography (petroleum/ethyl acetate = 20:1) afforded 0.2351 g (31%) of **1g** as a colorless oil. Analytical data for **1g**: ^1^H NMR (400 MHz, CDCl_3_) *δ* 7.38–7.31 (m, 4H), 7.12–7.07 (m, 2H), 6.66 (t, *J* = 7.1 Hz, 1H), 6.56 (d, *J* = 7.6 Hz, 2H), 4.90 (dd, *J* = 9.0, 4.6 Hz, 1H), 3.86 (s, 3H), 3.41 (dd, *J* = 14.6, 4.6 Hz, 1H), 3.30 (dd, *J* = 14.4, 9.2 Hz, 1H), 1.30 (s, 9H). ^13^C NMR (100 MHz, CDCl_3_) *δ* 190.9, 162.0, 150.2, 146.7, 139.3, 129.2, 126.1, 125.7, 117.9, 114.0, 55.0, 52.5, 47.5, 34.6, 31.5. HRMS (ESI-TOF) *m/z*: [M + H]^+^ calcd for C_22_H_26_N_3_O_3_^+^ 380.1969; found 380.1965.
*Methyl 2-diazo-3-oxo-5-(phenylamino)-5-(m-tolyl)pentanoate* **1h.** m.p.: 138.4–140.3 °C. The title compound was prepared according to the general procedure using the corresponding imine (2.0 mmol, 1.0 equiv.), **G** (3.0 mmol, 1.5 equiv.), Sc(OTf)_3_ (0.01 g, 2.0 mol%). Column chromatography (petroleum/ethyl acetate = 20:1) afforded 0.1146 g (17%) of **1h** as a colorless solid. Analytical data for **1h**: ^1^H NMR (400 MHz, CDCl_3_) *δ* 7.26–7.18 (m, 3H), 7.11–7.03 (m, 3H), 6.66 (t, *J* = 7.4 Hz, 1H), 6.55 (d, *J* = 7.6 Hz, 2H), 4.86 (dd, *J* = 9.2, 4.4 Hz, 1H), 3.87 (s, 3H), 3.39 (dd, *J* = 14.4, 4.4 Hz, 1H), 3.29 (dd, *J* = 14.4, 9.2 Hz, 1H), 2.33 (s, 3H). ^13^C NMR (100 MHz, CDCl_3_) *δ* 190.8, 162.0, 146.9, 142.7, 138.5, 129.2, 128.7, 128.2, 127.1, 123.5, 117.7, 113.8, 55.2, 52.5, 47.7, 21.7. HRMS (ESI-TOF) *m/z*: [M + H]^+^ calcd for C_19_H_20_N_3_O_3_^+^ 338.1499; found 338.1495.
*Methyl 5-(3-bromophenyl)-2-diazo-3-oxo-5-(phenylamino)pentanoate* **1i**. The title compound was prepared according to the general procedure using the corresponding imine (2.0 mmol, 1.0 equiv.), **G** (3.0 mmol, 1.5 equiv.), Sc(OTf)_3_ (0.01 g, 2.0 mol%). Column chromatography (petroleum/ethyl acetate = 20:1) afforded 0.1765 g (22%) of **1i** as a colorless oil. Analytical data for **1i**: ^1^H NMR (400 MHz, CDCl_3_) *δ* 7.60 (s, 1H), 7.41–7.35 (m, 2H), 7.19 (t, *J* = 7.8 Hz, 1H), 7.10 (t, *J* = 8.0 Hz, 2H), 6.69 (t, *J* = 7.2 Hz, 1H), 6.53 (d, *J* = 8.0 Hz, 2H), 4.84 (dd, *J* = 9.4, 4.2 Hz, 1H), 3.87 (s, 3H), 3.40 (dd, *J* = 14.4, 4.4 Hz, 1H), 3.25 (dd, *J* = 14.2, 9.4 Hz, 1H). ^13^C NMR (100 MHz, CDCl_3_) *δ* 190.3, 161.9, 146.2, 145.2, 130.7, 130.5, 129.6, 129.3, 125.2, 123.0, 118.4, 114.1, 55.0, 52.6, 47.4. HRMS (ESI-TOF) *m/z*: [M + H]^+^ calcd for C_18_H_17_BrN_3_O_3_^+^ 402.0448; found 402.0445.
*Methyl 2-diazo-3-oxo-5-(phenylamino)-5-(3-(trifluoromethyl)phenyl)pentanoate* **1j**. m.p.: 108.6–110.2 °C. The title compound was prepared according to the general procedure using the corresponding imine (2.0 mmol, 1.0 equiv.), **G** (3.0 mmol, 1.5 equiv.), Sc(OTf)_3_ (0.01 g, 2.0 mol%). Column chromatography (petroleum/ethyl acetate = 20:1) afforded 0.2660 g (34%) of **1j** as a colorless solid. Analytical data for **1j**: ^1^H NMR (400 MHz, CDCl_3_) *δ* 7.74 (s, 1H), 7.67 (d, *J* = 7.6 Hz, 1H), 7.62 (s, 1H), 7.51 (d, *J* = 8.0 Hz, 1H), 7.47–7.36 (m, 3H), 7.20 (t, *J* = 7.8 Hz, 1H), 7.10 (t, *J* = 8.0 Hz, 4H), 6.68 (t, *J* = 7.2 Hz, 2H), 6.52 (d, *J* = 8.4 Hz, 4H), 4.95 (dd, *J* = 9.4, 4.2 Hz, 1H), 4.86 (dd, *J* = 9.4, 4.2 Hz, 1H), 4.78 (s, 1H), 3.87 (d, *J* = 2.0 Hz, 6H), 3.45–3.36 (m, 2H), 3.31–3.20 (m, 2H). ^13^C NMR (100 MHz, CDCl_3_) *δ* 190.3, 190.2, 161.9, 161.9, 146.4, 146.4, 145.2, 143.9, 131.2 (q, J_C–F_ = 32.0 Hz), 130.6, 130.5, 129.9, 129.9, 129.5, 129.4, 129.3, 129.3, 125.2, 124.4 (q, *J*_C–F_ = 4.0 Hz), 124.2 (d, *J*_C–F_ = 270.7 Hz), 123.3 (q, *J*_C–F_ = 3.7 Hz), 123.0, 118.2, 118.1, 113.8, 55.0, 54.8, 52.6, 47.5, 47.4. HRMS (ESI-TOF) *m/z*: [M + H]^+^ calcd for C_19_H_17_F_3_N_3_O_3_^+^ 392.1217; found 392.1212.
*Methyl 2-diazo-5-(3-methoxyphenyl)-3-oxo-5-(phenylamino)pentanoate* **1k**. m.p.: 126.4–128.2 °C. The title compound was prepared according to the general procedure using the corresponding imine (2.0 mmol, 1.0 equiv.), **G** (3.0 mmol, 1.5 equiv.), Sc(OTf)_3_ (0.01 g, 2.0 mol%). Column chromatography (petroleum/ethyl acetate = 20:1) afforded 0.1413 g (20%) of **1k** as a colorless solid. Analytical data for **1k**: ^1^H NMR (400 MHz, CDCl_3_) *δ* 7.23 (d, *J* = 8.0 Hz, 1H), 7.11–7.01 (m, 4H), 6.78 (dd, *J* = 7.8, 2.2 Hz, 1H), 6.66 (t, *J* = 7.4 Hz, 1H), 6.54 (d, *J* = 8.0 Hz, 2H), 4.87 (dd, *J* = 9.4, 4.2 Hz, 1H), 3.86 (s, 3H), 3.79 (s, 3H), 3.41 (dd, *J* = 14.4, 4.4 Hz, 1H), 3.27 (dd, *J* = 14.2, 9.4 Hz, 1H). ^13^C NMR (100 MHz, CDCl_3_) *δ* 190.7, 161.9, 160.1, 146.8, 144.5, 129.9, 129.2, 118.8, 117.9, 113.8, 112.7, 112.1, 55.32, 55.3, 52.5, 47.6. HRMS (ESI-TOF) *m/z*: [M + H]^+^ calcd for C_19_H_20_N_3_O_4_^+^ 354.1448; found 354.1444.
*Methyl 2-diazo-5-(2-nitrophenyl)-3-oxo-5-(phenylamino)pentanoate* **1l**. The title compound was prepared according to the general procedure using the corresponding imine (2.0 mmol, 1.0 equiv.), **G** (3.0 mmol, 1.5 equiv.), Sc(OTf)_3_ (0.01 g, 2.0 mol%). Column chromatography (petroleum/ethyl acetate = 20:1) afforded 0.2650 g (36%) of **1l** as yellow oil. Analytical data for **1l**: ^1^H NMR (400 MHz, CDCl_3_) *δ* 7.93 (dd, *J* = 8.2, 1.4 Hz, 1H), 7.79 (d, *J* = 7.6 Hz, 1H), 7.54 (td, *J* = 7.6, 1.2 Hz, 1H), 7.41–7.36 (m, 1H), 7.10–7.06 (m, 2H), 6.69 (t, *J* = 7.2 Hz, 1H), 6.52 (d, *J* = 7.6 Hz, 2H), 5.60 (dd, *J* = 8.2, 4.2 Hz, 1H), 3.83 (s, 3H), 3.58 (dd, *J* = 15.8, 8.6 Hz, 1H), 3.37 (dd, *J* = 15.6, 4.0 Hz, 1H). ^13^C NMR (100 MHz, CDCl_3_) *δ* 189.9, 161.9, 149.0, 145.7, 137.7, 133.7, 129.4, 129.0, 128.5, 125.1, 118.8, 114.0, 52.6, 51.1, 45.7. HRMS (ESI-TOF) *m/z*: [M + H]^+^ calcd for C_18_H_17_N_4_O_5_^+^ 369.1193; found 369.1189.
*Methyl 2-diazo-5-(2-methoxyphenyl)-3-oxo-5-(phenylamino)pentanoate* **1m**. The title compound was prepared according to the general procedure using the corresponding imine (2.0 mmol, 1.0 equiv.), **G** (3.0 mmol, 1.5 equiv.), Sc(OTf)_3_ (0.01 g, 2.0 mol%). Column chromatography (petroleum/ethyl acetate = 20:1) afforded 0.1907 g (27%) of **1m** as a colorless oil. Analytical data for **1m**: ^1^H NMR (400 MHz, CDCl_3_) *δ* 7.35 (dd, *J* = 7.8, 1.4 Hz, 1H), 7.23–7.18 (m, 1H), 7.11–7.06 (m, 2H), 6.90–6.86 (m, 2H), 6.64 (t, *J* = 7.4 Hz, 1H), 6.59–6.56 (m, 2H), 5.26 (dd, *J* = 8.4, 4.8 Hz, 1H), 5.02 (s, 1H), 3.92 (s, 3H), 3.83 (s, 3H), 3.59 (dd, *J* = 15.2, 8.4 Hz, 1H), 3.25 (dd, *J* = 15.2, 4.8 Hz, 1H). ^13^C NMR (100 MHz, CDCl_3_) *δ* 191.2, 162.0, 157.1, 147.0, 129.7, 129.2, 128.4, 128.0, 120.8, 117.7, 113.9, 110.7, 55.5, 52.4, 51.0, 44.8. HRMS (ESI-TOF) *m/z*: [M + H]^+^ calcd for C_19_H_20_N_3_O_4_^+^ 354.1448; found 354.1444.
*Methyl 2-diazo-3-oxo-5-(phenylamino)-5-(o-tolyl)pentanoate* **1n**. The title compound was prepared according to the general procedure using the corresponding imine (2.0 mmol, 1.0 equiv.), **G** (3.0 mmol, 1.5 equiv.), Sc(OTf)_3_ (0.01 g, 2.0 mol%). Column chromatography (petroleum/ethyl acetate = 20:1) afforded 0.1281 g (19%) of **1n** as a colorless oil. Analytical data for **1n**: ^1^H NMR (400 MHz, CDCl_3_) *δ* 7.43 (d, *J* = 3.6 Hz, 1H), 7.16–7.13 (m, 3H), 7.10–7.05 (m, 2H), 6.65 (t, *J* = 7.4 Hz, 1H), 6.49 (d, *J* = 8.0 Hz, 2H), 5.11 (dd, *J* = 9.2, 4.8 Hz, 1H), 3.84 (s, 3H), 3.43 (dd, *J* = 14.8, 9.2 Hz, 1H), 3.26 (dd, *J* = 15.0, 4.6 Hz, 1H), 2.50 (s, 3H). ^13^C NMR (100 MHz, CDCl_3_) *δ* 191.0, 162.1, 146.7, 140.2, 135.4, 130.9, 129.3, 127.3, 126.7, 125.5, 117.8, 113.6, 52.5, 51.9, 45.3, 19.2. HRMS (ESI-TOF) *m/z*: [M + H]^+^ calcd for C_19_H_20_N_3_O_3_^+^ 338.1499; found 338.1492.
*Methyl 5-(2-bromophenyl)-2-diazo-3-oxo-5-(phenylamino)pentanoate* **1o**. The title compound was prepared according to the general procedure using the corresponding imine (2.0 mmol, 1.0 equiv.), **G** (3.0 mmol, 1.5 equiv.), Sc(OTf)_3_ (0.01 g, 2.0 mol%). Column chromatography (petroleum/ethyl acetate = 20:1) afforded 0.2085 g (26%) of **1o** as a colorless oil. Analytical data for **1o**: ^1^H NMR (400 MHz, CDCl_3_) *δ* 7.55 (dd, *J* = 8.0, 1.2 Hz, 1H), 7.49 (dd, *J* = 7.8, 1.8 Hz, 1H), 7.23 (td, *J* = 7.6, 1.4 Hz, 1H), 7.12–7.06 (m, 3H), 6.66 (t, *J* = 7.4 Hz, 1H), 6.50 (d, *J* = 7.6 Hz, 2H), 5.28 (dd, *J* = 8.8, 4.0 Hz, 1H), 3.84 (s, 3H), 3.59 (dd, *J* = 15.2, 8.8 Hz, 1H), 3.19 (dd, *J* = 15.6, 4.0 Hz, 1H). ^13^C NMR (100 MHz, CDCl_3_) *δ* 190.4, 162.1, 146.2, 140.7, 133.2, 129.3, 129.0, 128.2, 128.0, 123.2, 118.1, 113.9, 54.6, 52.6, 44.6. HRMS (ESI-TOF) *m/z*: [M + H]^+^ calcd for C_18_H_17_BrN_3_O_3_^+^ 402.0448; found 402.0445.
*Methyl 2-diazo-3-oxo-5-(phenylamino)-5-(3,4,5-trimethoxyphenyl)pentanoate* **1p**. The title compound was prepared according to the general procedure using the corresponding imine (2.0 mmol, 1.0 equiv.), **G** (3.0 mmol, 1.5 equiv.), Sc(OTf)_3_ (0.01 g, 2.0 mol%). Column chromatography (petroleum/ethyl acetate = 20:1) afforded 0.1901 g (23%) of **1p** as a colorless oil. Analytical data for **1p**: ^1^H NMR (400 MHz, CDCl_3_) *δ* 7.10 (t, *J* = 7.8 Hz, 2H), 6.68 (t, *J* = 7.2 Hz, 3H), 6.55 (d, *J* = 7.6 Hz, 2H), 4.79 (dd, *J* = 9.2, 4.4 Hz, 1H), 3.86 (s, 3H), 3.84 (s, 6H), 3.82 (s, 3H), 3.40 (dd, *J* = 14.4, 4.4 Hz, 1H), 3.27 (dd, *J* = 14.2, 9.4 Hz, 1H). ^13^C NMR (100 MHz, CDCl_3_) *δ* 190.8, 161.9, 153.6, 146.9, 138.5, 137.1, 129.2, 118.0, 113.9, 103.2, 60.9, 56.3, 55.8, 52.5, 47.4. HRMS (ESI-TOF) *m/z*: [M + H]^+^ calcd for C_21_H_24_N_3_O_6_^+^ 414.1660; found 414.1656.
*Methyl 2-diazo-5-(2,4-dichlorophenyl)-3-oxo-5-(phenylamino)pentanoate* **1q**. The title compound was prepared according to the general procedure using the corresponding imine (2.0 mmol, 1.0 equiv.), **G** (3.0 mmol, 1.5 equiv.), Sc(OTf)_3_ (0.01 g, 2.0 mol%). Column chromatography (petroleum/ethyl acetate = 20:1) afforded 0.2581 g (33%) of **1q** as a colorless oil. Analytical data for **1q**: ^1^H NMR (400 MHz, CDCl_3_) *δ* 7.42 (d, *J* = 8.4 Hz, 1H), 7.39 (d, *J* = 2.0 Hz, 1H), 7.16 (dd, *J* = 8.4, 2.0 Hz, 1H), 7.09 (dd, *J* = 8.6, 7.4 Hz, 2H), 6.67 (t, *J* = 7.4 Hz, 1H), 6.46 (d, *J* = 7.6 Hz, 2H), 5.25 (dd, *J* = 8.8, 4.0 Hz, 1H), 5.00 (s, 1H), 3.85 (s, 3H), 3.54 (dd, *J* = 15.6, 8.8 Hz, 1H), 3.19 (dd, *J* = 15.2, 4.0 Hz, 1H). ^13^C NMR (100 MHz, CDCl_3_) *δ* 190.2, 162.0, 146.2, 138.1, 133.7, 133.6, 129.7, 129.3, 129.1, 127.7, 118.2, 113.6, 52.6, 51.8, 44.3. HRMS (ESI-TOF) *m/z*: [M + H]^+^ calcd for C_18_H_16_Cl_2_N_3_O_3_^+^ 392.0563; found 392.0555.
*Methyl 2-diazo-5-(2,3-dihydrobenzo[b][1,4]dioxin-6-yl)-3-oxo-5-(phenylamino)pentanoate* **1r**. m.p.: 139.2–141.0 °C. The title compound was prepared according to the general procedure using the corresponding imine (2.0 mmol, 1.0 equiv.), **G** (3.0 mmol, 1.5 equiv.), Sc(OTf)_3_ (0.01 g, 2.0 mol%). Column chromatography (petroleum/ethyl acetate = 20:1) afforded 0.2744 g (36%) of **1r** as a colorless solid. Analytical data for **1r**: ^1^H NMR (400 MHz, CDCl_3_) *δ* 7.09 (dd, *J* = 8.8, 7.2 Hz, 2H), 6.97 (d, *J* = 2.4 Hz, 1H), 6.93 (dd, *J* = 8.2, 2.2 Hz, 1H), 6.81 (d, *J* = 8.4 Hz, 1H), 6.66 (t, *J* = 7.2 Hz, 1H), 6.55 (d, *J* = 7.6 Hz, 2H), 4.81 (dd, *J* = 9.2, 4.4 Hz, 1H), 4.21 (s, 4H), 3.86 (s, 3H), 3.37 (dd, *J* = 14.6, 4.6 Hz, 1H), 3.24 (dd, *J* = 14.4, 9.2 Hz, 1H). ^13^C NMR (100 MHz, CDCl_3_) *δ* 190.6, 161.9, 146.8, 143.7, 142.8, 136.0, 129.1, 119.3, 117.7, 117.5, 115.2, 113.8, 64.4, 54.6, 52.4, 47.7. HRMS (ESI-TOF) *m/z*: [M + H]^+^ calcd for C_20_H_20_N_3_O_5_^+^ 382.1397; found 382.1391.
*Methyl 2-diazo-5-(furan-2-yl)-3-oxo-5-(phenylamino)pentanoate* **1s**. m.p.: 56.2–57.0 °C. The title compound was prepared according to the general procedure using the corresponding imine (2.0 mmol, 1.0 equiv.), **G** (3.0 mmol, 1.5 equiv.), Sc(OTf)_3_ (0.01 g, 2.0 mol%). Column chromatography (petroleum/ethyl acetate = 20:1) afforded 0.2004 g (32%) of **1s** as a colorless solid. Analytical data for **1s**: ^1^H NMR (400 MHz, CDCl_3_) *δ* 7.33 (d, *J* = 1.2 Hz, 1H), 7.17–7.13 (m, 2H), 6.72 (t, *J* = 7.4 Hz, 1H), 6.66 (d, *J* = 7.6 Hz, 2H), 6.27 (dd, *J* = 3.2, 1.6 Hz, 1H), 6.20 (d, *J* = 3.6 Hz, 1H), 5.13 (dd, *J* = 7.6, 5.6 Hz, 1H), 4.40 (s, 1H), 3.85 (s, 3H), 3.55 (d, *J* = 15.0, 7.6 Hz, 1H), 3.39 (dd, *J* = 15.8, 5.4 Hz, 1H). ^13^C NMR (100 MHz, CDCl_3_) *δ* 190.2, 161.9, 154.9, 146.6, 141.9, 129.3, 118.4, 114.0, 110.4, 106.4, 52.5, 49.0, 43.9. HRMS (ESI-TOF) *m/z*: [M + H]^+^ calcd for C_16_H_16_N_3_O_4_^+^ 314.1135; found 314.1130.
*Methyl 2-diazo-5-(naphthalen-2-yl)-3-oxo-5-(phenylamino)pentanoate* **1u**. m.p.: 114.2–115.8 °C. The title compound was prepared according to the general procedure using the corresponding imine (2.0 mmol, 1.0 equiv.), **G** (3.0 mmol, 1.5 equiv.), Sc(OTf)_3_ (0.01 g, 2.0 mol%). Column chromatography (petroleum/ethyl acetate = 20:1) afforded 0.1940 g (26%) of **1u** as a colorless solid. Analytical data for **1u**: ^1^H NMR (400 MHz, CDCl_3_) *δ* 7.89 (s, 1H), 7.84–7.79 (m, 3H), 7.61 (dd, *J* = 8.4, 2.0 Hz, 1H), 7.47–7.43 (m, 2H), 7.08 (dd, *J* = 8.4, 7.2 Hz, 2H), 6.68 (t, *J* = 7.2 Hz, 1H), 6.63 (d, *J* = 8.0 Hz, 2H), 5.06 (dd, *J* = 9.2, 4.8 Hz, 1H), 3.85 (s, 3H), 3.56 (dd, *J* = 15.0, 4.6 Hz, 1H), 3.42 (dd, *J* = 14.2, 9.4 Hz, 1H). ^13^C NMR (100 MHz, CDCl_3_) *δ* 190.7, 162.0, 146.7, 140.1, 133.6, 133.0, 129.2, 128.7, 128.1, 127.8, 126.2, 125.9, 125.2, 124.7, 117.9, 114.0, 55.5, 52.5, 47.6. HRMS (ESI-TOF) *m/z*: [M + H]^+^ calcd for C_22_H_20_N_3_O_3_^+^ 374.1499; found 374.1498.
*Methyl 2-diazo-3-oxo-5-phenyl-5-(p-tolylamino)pentanoate* **1v**. m.p.: 128.3–129.7 °C. The title compound was prepared according to the general procedure using the corresponding imine (2.0 mmol, 1.0 equiv.), **G** (3.0 mmol, 1.5 equiv.), Sc(OTf)_3_ (0.01 g, 2.0 mol%). Column chromatography (petroleum/ethyl acetate = 20:1) afforded 0.1618 g (24%) of **1v** as a colorless solid. Analytical data for **1v**: ^1^H NMR (400 MHz, CDCl_3_) *δ* 7.44 (d, *J* = 6.8 Hz, 2H), 7.31 (t, *J* = 7.6 Hz, 2H), 7.23 (t, *J* = 7.2 Hz, 1H), 6.90 (d, *J* = 8.4 Hz, 2H), 6.51 (d, *J* = 8.4 Hz, 2H), 4.86 (dd, *J* = 9.0, 4.6 Hz, 1H), 3.85 (s, 3H), 3.47 (dd, *J* = 15.0, 4.6 Hz, 1H), 3.35 (dd, *J* = 14.8, 9.2 Hz, 1H), 2.18 (s, 3H). ^13^C NMR (100 MHz, CDCl_3_) *δ* 190.8, 162.0, 144.5, 142.8, 129.7, 128.8, 127.3, 127.0, 126.4, 114.0, 55.5, 52.5, 47.7, 20.5. HRMS (ESI-TOF) *m/z*: [M + H]^+^ calcd for C_19_H_20_N_3_O_3_^+^ 338.1499; found 338.1492.
*Methyl 2-diazo-3-oxo-5-phenyl-5-((4-(trifluoromethyl)phenyl)amino)pentanoate* **1w**. The title compound was prepared according to the general procedure using the corresponding imine (2.0 mmol, 1.0 equiv.), **G** (3.0 mmol, 1.5 equiv.), Sc(OTf)_3_ (0.01 g, 2.0 mol%). Column chromatography (petroleum/ethyl acetate = 20:1) afforded 0.1877 g (24%) of **1w** as a colorless oil. Analytical data for **1w**: ^1^H NMR (400 MHz, CDCl_3_) *δ* 7.39 (d, *J* = 7.2 Hz, 2H), 7.33–7.26 (m, 4H), 7.24–7.20 (m, 1H), 6.50 (d, *J* = 8.8 Hz, 2H), 5.14 (s, 1H), 4.89 (dd, *J* = 9.4, 4.6 Hz, 1H), 3.83 (s, 3H), 3.37 (dd, *J* = 14.6, 4.6 Hz, 1H), 3.28 (dd, *J* = 14.4, 9.2 Hz, 1H). ^13^C NMR (100 MHz, CDCl_3_) *δ* 190.5, 162.0, 149.4, 141.8, 129.0, 127.7, 126.5 (q, *J*_C–F_ = 3.7 Hz), 126.3, 125.0 (d, *J*_C–F_ = 268.6 Hz) 119.2 (q, *J*_C–F_ = 32.4 Hz) 112.9, 54.8, 52.6, 47.4. HRMS (ESI-TOF) *m/z*: [M + H]^+^ calcd for C_19_H_17_F_3_N_3_O_3_^+^ 392.1217; found 392.1207.
*Methyl 5-((4-chlorophenyl)amino)-2-diazo-3-oxo-5-phenylpentanoate* **1x**. m.p.: 125.2–127.0 °C. The title compound was prepared according to the general procedure using the corresponding imine (2.0 mmol, 1.0 equiv.), **G** (3.0 mmol, 1.5 equiv.), Sc(OTf)_3_ (0.01 g, 2.0 mol%). Column chromatography (petroleum/ethyl acetate = 20:1) afforded 0.1928 g (27%) of **1x** as a colorless solid. Analytical data for **1x**: ^1^H NMR (400 MHz, CDCl_3_) *δ* 7.43–7.40 (m, 2H), 7.33 (t, *J* = 7.4 Hz, 2H), 7.23 (d, *J* = 7.2 Hz, 1H), 7.02 (t, *J* = 2.6 Hz, 1H), 7.00 (t, *J* = 2.6 Hz, 1H), 6.45 (t, *J* = 2.6 Hz, 1H), 6.44 (t, *J* = 2.6 Hz, 1H), 4.85 (dd, *J* = 9.2, 4.4 Hz, 1H), 3.86 (s, 3H), 3.38 (dd, *J* = 14.4, 4.4 Hz, 1H), 3.29 (dd, *J* = 14.6, 9.4 Hz, 1H). ^13^C NMR (100 MHz, CDCl_3_) *δ* 190.6, 161.9, 145.3, 142.0, 129.0, 128.9, 127.6, 126.4, 122.5, 115.0, 55.4, 52.5, 47.4. HRMS (ESI-TOF) *m/z*: [M + H]^+^ calcd for C_18_H_17_ClN_3_O_3_^+^ 358.0953; found 358.0949.
*Methyl 2-diazo-3-oxo-5-phenyl-5-(m-tolylamino)pentanoate* **1y**. The title compound was prepared according to the general procedure using the corresponding imine (2.0 mmol, 1.0 equiv.), **G** (3.0 mmol, 1.5 equiv.), Sc(OTf)_3_ (0.01 g, 2.0 mol%). Column chromatography (petroleum/ethyl acetate = 20:1) afforded 0.1686 g (25%) of **1y** as a colorless oil. Analytical data for **1y**: ^1^H NMR (400 MHz, CDCl_3_) *δ* 7.45 (d, *J* = 7.2 Hz, 2H), 7.32 (t, *J* = 7.6 Hz, 2H), 7.23 (t, *J* = 7.4 Hz, 1H), 6.96 (t, *J* = 7.8 Hz, 1H), 6.49 (d, *J* = 7.2 Hz, 1H), 6.39 (s, 1H), 6.33 (dd, *J* = 8.2, 1.8 Hz, 1H), 4.89 (dd, *J* = 9.4, 4.6 Hz, 1H), 3.86 (s, 3H), 3.41 (dd, *J* = 14.4, 4.4 Hz, 1H), 3.28 (dd, *J* = 14.4, 9.2 Hz, 1H), 2.20 (s, 3H). ^13^C NMR (100 MHz, CDCl_3_) *δ* 190.8, 162.0, 146.7, 142.6, 139.0, 129.1, 128.8, 127.4, 126.5, 118.9, 114.8, 110.9, 55.3, 52.5, 47.6, 21.7. HRMS (ESI-TOF) *m/z*: [M + H]^+^ calcd for C_19_H_20_N_3_O_3_^+^ 338.1499; found 338.1491.
*Methyl 5-((3-bromophenyl)amino)-2-diazo-3-oxo-5-phenylpentanoate* **1z**. m.p.: 124.3–126.1 °C. The title compound was prepared according to the general procedure using the corresponding imine (2.0 mmol, 1.0 equiv.), **G** (3.0 mmol, 1.5 equiv.), Sc(OTf)_3_ (0.01 g, 2.0 mol%). Column chromatography (petroleum/ethyl acetate = 20:1) afforded 0.2406 g (30%) of **1z** as a colorless solid. Analytical data for **1z**: ^1^H NMR (400 MHz, CDCl_3_) *δ* 7.41 (d, *J* = 7.2 Hz, 2H), 7.33 (t, *J* = 7.6 Hz, 2H), 7.26–7.22 (m, 1H), 6.90 (t, *J* = 8.0 Hz, 1H), 6.76–6.73 (m, 1H), 6.67 (t, *J* = 2.0 Hz, 1H), 6.41 (dd, *J* = 8.2, 1.8 Hz, 1H), 4.85 (dd, *J* = 9.2, 4.4 Hz, 1H), 4.72 (s, 1H), 3.87 (s, 3H), 3.36 (dd, *J* = 14.2, 4.6 Hz, 1H), 3.28 (dd, *J* = 14.4, 9.2 Hz, 1H). ^13^C NMR (100 MHz, CDCl_3_) *δ* 190.6, 162.0, 148.2, 142.0, 130.5, 129.0, 127.6, 126.3, 123.2, 120.6, 116.5, 112.3, 55.1, 52.6, 47.5. HRMS (ESI-TOF) *m/z*: [M + H]^+^ calcd for C_18_H_17_BrN_3_O_3_^+^ 402.0447; found 402.0445.
*Methyl 2-diazo-5-((3-nitrophenyl)amino)-3-oxo-5-phenylpentanoate* **1aa**. The title compound was prepared according to the general procedure using the corresponding imine (2.0 mmol, 1.0 equiv.), **G** (3.0 mmol, 1.5 equiv.), Sc(OTf)_3_ (0.01 g, 2.0 mol%). Column chromatography (petroleum/ethyl acetate = 20:1) afforded 0.1988 g (27%) of **1aa** as yellow oil. Analytical data for **1aa**: ^1^H NMR (400 MHz, CDCl_3_) *δ* 7.46–7.40 (m, 3H), 7.35–7.30 (m, 3H), 7.25–7.22 (m, 1H), 7.16 (t, *J* = 8.2 Hz, 1H), 6.76 (dd, *J* = 8.0, 2.0 Hz, 1H), 5.25 (s, 1H), 4.91 (t, *J* = 6.8 Hz, 1H), 3.87 (s, 3H), 3.35 (d, *J* = 7.2 Hz, 2H). ^13^C NMR (100 MHz, CDCl_3_) *δ* 190.4, 162.0, 149.3, 147.6, 141.3, 129.8, 129.1, 127.9, 126.4, 119.4, 112.5, 107.9, 55.2, 52.7, 47.3. HRMS (ESI-TOF) *m/z*: [M + H]^+^ calcd for C_18_H_17_N_4_O_5_^+^ 369.1193; found 369.1184.
*Methyl 2-diazo-5-((2-methoxyphenyl)amino)-3-oxo-5-phenylpentanoate* **1ab**. m.p.: 106.7–108.3 °C. The title compound was prepared according to the general procedure using the corresponding imine (2.0 mmol, 1.0 equiv.), **G** (3.0 mmol, 1.5 equiv.), Sc(OTf)_3_ (0.01 g, 2.0 mol%). Column chromatography (petroleum/ethyl acetate = 20:1) afforded 0.1130 g (16%) of **1ab** as a colorless oil. Analytical data for **1ab**: ^1^H NMR (400 MHz, CDCl_3_) *δ* 7.45 (d, *J* = 7.2 Hz, 2H), 7.31 (t, *J* = 7.6 Hz, 2H), 7.22 (t, *J* = 7.4 Hz, 1H), 6.75 (dd, *J* = 8.0, 1.2 Hz, 1H), 6.70 (td, *J* = 7.6, 1.2 Hz, 1H), 6.62 (td, *J* = 7.8, 1.4 Hz, 1H), 6.43–6.40 (m, 1H), 5.29 (s, 1H), 4.95 (dd, *J* = 8.2, 5.8 Hz, 1H), 3.88 (s, 3H), 3.86 (s, 3H), 3.44–3.35 (m, 2H). ^13^C NMR (100 MHz, CDCl_3_) *δ* 190.5, 161.9, 147.1, 142.6, 136.5, 128.8, 127.4, 126.5, 121.2, 117.1, 111.5, 109.6, 55.7, 54.9, 52.4, 47.8. HRMS (ESI-TOF) *m/z*: [M + H]^+^ calcd for C_19_H_20_N_3_O_4_^+^ 354.1448; found 354.1444.
*Methyl 2-diazo-3-oxo-5-phenyl-5-(o-tolylamino)pentanoate* **1ac**. The title compound was prepared according to the general procedure using the corresponding imine (2.0 mmol, 1.0 equiv.), **G** (3.0 mmol, 1.5 equiv.), Sc(OTf)_3_ (0.01 g, 2.0 mol%). Column chromatography (petroleum/ethyl acetate = 20:1) afforded 0.1281 g (19%) of **1ac** as a colorless oil. Analytical data for **1ac**: ^1^H NMR (400 MHz, CDCl_3_) *δ* 7.48–7.45 (m, 2H), 7.35–7.31 (m, 2H), 7.26–7.21 (m, 1H), 7.04 (d, *J* = 6.8 Hz, 1H), 6.96–6.91 (m, 1H), 6.63–6.58 (m, 1H), 6.35 (d, *J* = 8.0 Hz, 1H), 4.92 (dd, *J* = 9.8, 4.2 Hz, 1H), 4.77 (s, 1H), 3.87 (s, 3H), 3.44 (dd, *J* = 13.8, 4.2 Hz, 1H), 3.33 (dd, *J* = 13.8, 9.8 Hz, 1H), 2.23 (s, 3H). ^13^C NMR (100 MHz, CDCl_3_) *δ* 190.9, 162.0, 144.8, 142.7, 130.1, 128.9, 127.4, 127.0, 126.3, 122.4, 117.3, 111.4, 55.5, 52.5, 47.7, 17.8. HRMS (ESI-TOF) *m/z*: [M + H]^+^ calcd for C_19_H_20_N_3_O_3_^+^ 338.1499; found 338.1497.
*Methyl 5-((2-chlorophenyl)amino)-2-diazo-3-oxo-5-phenylpentanoate* **1ad**. The title compound was prepared according to the general procedure using the corresponding imine (2.0 mmol, 1.0 equiv.), **G** (3.0 mmol, 1.5 equiv.), Sc(OTf)_3_ (0.01 g, 2.0 mol%). Column chromatography (petroleum/ethyl acetate = 20:1) afforded 0.1500 g (21%) of **1ad** as a colorless oil. Analytical data for **1ad**: ^1^H NMR (400 MHz, CDCl_3_) *δ* 7.43 (d, *J* = 7.2 Hz, 2H), 7.33 (t, *J* = 7.4 Hz, 2H), 7.25–7.21 (m, 2H), 6.95 (t, *J* = 7.4 Hz, 1H), 6.58 (t, *J* = 7.6 Hz, 1H), 6.45 (d, *J* = 8.0 Hz, 1H), 5.45 (s, 1H), 4.97 (dd, *J* = 9.4, 4.6 Hz, 1H), 3.87 (s, 3H), 3.52 (dd, *J* = 14.2, 9.4 Hz, 1H), 3.31 (dd, *J* = 14.4, 4.4 Hz, 1H). ^13^C NMR (100 MHz, CDCl_3_) *δ* 190.4, 162.1, 142.7, 142.0, 129.2, 128.9, 127.7, 127.6, 126.4, 119.6, 117.8, 112.9, 55.27, 52.6, 47.4. HRMS (ESI-TOF) *m/z*: [M + H]^+^ calcd for C_18_H_17_ClN_3_O_3_^+^ 358.0953; found 358.0945.
*Methyl 2-diazo-3-oxo-5-phenyl-5-((2-(trifluoromethyl)phenyl)amino)pentanoate* **1ae.** The title compound was prepared according to the general procedure using the corresponding imine (2.0 mmol, 1.0 equiv.), **G** (3.0 mmol, 1.5 equiv.), Sc(OTf)_3_ (0.01 g, 2.0 mol%). Column chromatography (petroleum/ethyl acetate = 20:1) afforded 0.1877 g (24%) of **1ae** as a colorless oil. Analytical data for **1ae**: ^1^H NMR (400 MHz, CDCl_3_) *δ* 7.44–7.40 (m, 3H), 7.36–7.31 (m, 2H), 7.26–7.22 (m, 1H), 7.19–7.14 (m, 1H), 6.65 (t, *J* = 7.6 Hz, 1H), 6.50 (d, *J* = 8.4 Hz, 1H), 5.48 (s, 1H), 4.97 (dd, *J* = 9.4, 3.4 Hz, 1H), 3.89 (s, 3H), 3.63 (dd, *J* = 14.0, 10.0 Hz, 1H), 3.15 (dd, *J* = 14.2, 4.2 Hz, 1H). ^13^C NMR (100 MHz, CDCl_3_) *δ* 190.5, 162.2, 144.3, 141.9, 133.0, 129.0, 127.7, 126.6 (q, *J*_C–F_ = 5.5 Hz), 126.3, 121.8 (d, *J*_C–F_ = 415.1 Hz), 116.4, 113.9 (q, *J*_C–F_ = 27.9 Hz), 113.4, 55.4, 52.6, 47.2. HRMS (ESI-TOF) *m/z*: [M + H]^+^ calcd for C_19_H_17_F_3_N_3_O_3_^+^ 392.1216; found 392.1214.
*Methyl 2-diazo-3-oxo-5-phenyl-5-((3,4,5-trichlorophenyl)amino)pentanoate* **1af**. m.p.: 145.2–146.8 °C. The title compound was prepared according to the general procedure using the corresponding imine (2.0 mmol, 1.0 equiv.), **G** (3.0 mmol, 1.5 equiv.), Sc(OTf)_3_ (0.01 g, 2.0 mol%). Column chromatography (petroleum/ethyl acetate = 20:1) afforded 0.2635 g (31%) of **1af** as a colorless solid. Analytical data for **1af**: ^1^H NMR (400 MHz, CDCl_3_) *δ* 7.41–7.33 (m, 4H), 7.30–7.27 (m, 1H), 6.53 (s, 2H), 4.96 (s, 1H), 4.80 (dd, *J* = 8.4, 5.2 Hz, 1H), 3.89 (s, 3H), 3.38–3.27 (m, 2H). ^13^C NMR (100 MHz, CDCl_3_) *δ* 190.4, 162.0, 146.0, 141.1, 134.2, 129.1, 127.9, 126.2, 119.0, 115.2, 113.7, 55.1, 52.7, 47.2. HRMS (ESI-TOF) *m/z*: [M + H]^+^ calcd for C_18_H_15_Cl_3_N_3_O_3_^+^ 426.0173; found 426.0164.
*Propyl 2-diazo-3-oxo-5-phenyl-5-(phenylamino)pentanoate* **1ah**. The title compound was prepared according to the general procedure using the corresponding imine (2.0 mmol, 1.0 equiv.), **G** (3.0 mmol, 1.5 equiv.), Sc(OTf)_3_ (0.01 g, 2.0 mol%). Column chromatography (petroleum/ethyl acetate = 20:1) afforded 0.1966 g (28%) of **1ah** as a colorless oil. Analytical data for **1ah**: ^1^H NMR (400 MHz, CDCl_3_) *δ* 7.46 (d, *J* = 6.8 Hz, 2H), 7.33 (t, *J* = 7.4 Hz, 2H), 7.26–7.21 (m, 1H), 7.09 (dd, *J* = 8.8, 7.2 Hz, 2H), 6.66 (t, *J* = 7.4 Hz, 1H), 6.54 (d, *J* = 7.6 Hz, 2H), 4.91 (dd, *J* = 9.6, 4.4 Hz, 1H), 4.24 (t, *J* = 6.8 Hz, 2H), 3.41 (dd, *J* = 14.4, 4.4 Hz, 1H), 3.30 (dd, *J* = 14.2, 9.4 Hz, 1H), 1.74 (m, 2H), 0.99 (t, *J* = 7.4 Hz, 3H). ^13^C NMR (100 MHz, CDCl_3_) *δ* 190.9, 161.6, 146.8, 142.6, 129.2, 128.8, 127.4, 126.4, 117.8, 113.8, 67.3, 55.3, 47.6, 22.2, 10.4. HRMS (ESI-TOF) *m/z*: [M + H]^+^ calcd for C_20_H_22_N_3_O_3_^+^ 352.1656; found 352.1648.
*Benzyl 2-diazo-3-oxo-5-phenyl-5-(phenylamino)pentanoate* **1ai**. m.p.: 118.2–119.7 °C. The title compound was prepared according to the general procedure using the corresponding imine (2.0 mmol, 1.0 equiv.), **G** (3.0 mmol, 1.5 equiv.), Sc(OTf)_3_ (0.01 g, 2.0 mol%). Column chromatography (petroleum/ethyl acetate = 20:1) afforded 0.2076 g (26%) of **1ai** as a colorless solid. Analytical data for **1ai**: ^1^H NMR (400 MHz, CDCl_3_) *δ* 7.43–7.39 (m, 7H), 7.31 (t, *J* = 7.6 Hz, 2H), 7.23 (t, *J* = 7.2 Hz, 1H), 7.07 (dd, *J* = 8.6, 7.4 Hz, 2H), 6.66 (t, *J* = 7.4 Hz, 1H), 6.51 (d, *J* = 7.6 Hz, 2H), 5.34–5.25 (m, 2H), 4.89 (dd, *J* = 9.0, 4.6 Hz, 1H), 3.42–3.29 (m, 2H). ^13^C NMR (100 MHz, CDCl_3_) *δ* 190.7, 161.5, 146.6, 142.5, 135.1, 129.2, 128.9, 128.9, 128.8, 128.6, 127.4, 126.5, 117.9, 113.9, 67.3, 55.4, 47.6. HRMS (ESI-TOF) *m/z*: [M + H]^+^ calcd for C_24_H_22_N_3_O_3_^+^ 400.1656; found 400.1649.
Methyl (2S*,5R*)-2-allyl-3-oxo-1,5-diphenylpyrrolidine-2-carboxylate **3a.** The title compound was prepared according to the general procedure using the corresponding diazo (0.0487 g, 0.15 mmol, 1.0 equiv), allyl carbonate (0.0290 g, 0.25 mmol 1.7 equiv), Rh_2_(OAc)_4_ (0.0015 g, 2.3 mol%), Pd_2_(dba)_3_ (0.0037 g, 2.7 mol%), and X-phos (0.0021 g, 3.0 mol%). Column chromatography (petroleum/ethyl acetate = 20:1) afforded 0.0457 g (91%, d.r. > 20:1) of **3a** as a colorless oil. Analytical data for **3a**: ^1^H NMR (400 MHz, CDCl_3_) *δ* 7.49 (d, *J* = 8.0 Hz, 2H), 7.31 (t, *J* = 7.8 Hz, 2H), 7.22 (t, *J* = 7.0 Hz, 1H), 7.11 (t, *J* = 7.8 Hz, 2H), 6.75 (t, *J* = 7.6 Hz, 1H), 6.58 (d, *J* = 8.4 Hz, 2H), 5.45–5.34 (m, 1H), 5.09 (t, *J* = 8.2 Hz, 1H), 5.02 (d, *J* = 9.6 Hz, 1H), 4.92 (d, *J* = 17.2 Hz, 1H), 3.91 (s, 3H), 3.38 (dd, *J* = 14.0, 6.4 Hz, 1H), 3.14 (dd, *J* = 19.2, 8.4 Hz, 1H), 2.96 (dd, *J* = 14.0, 8.4 Hz, 1H), 2.78 (dd, *J* = 19.2, 7.6 Hz, 1H). ^13^C NMR (100 MHz, CDCl_3_) *δ* 208.2, 171.4, 143.6, 143.2, 130.7, 129.2, 129.0, 127.6, 126.2, 121.1, 119.2, 116.7, 76.4, 59.8, 53.6, 47.2, 35.0. HRMS (ESI-TOF) *m/z*: [M + H]^+^ calcd for C_21_H_22_NO_3_^+^ 336.1594; found 336.1590.
Methyl (2S*,5R*)-2-allyl-5-(4-cyanophenyl)-3-oxo-1-phenylpyrrolidine-2-carboxylate **3b.** The title compound was prepared according to the general procedure using the corresponding diazo (0.0.0522 g, 0.15 mmol, 1.0 equiv), allyl carbonate (0.0290 g, 0.25 mmol 1.7 equiv), Rh_2_(OAc)_4_ (0.0015 g, 2.3 mol%), Pd_2_(dba)_3_ (0.0037 g, 2.7 mol%), and X-phos (0.0021 g, 3.0 mol%). Column chromatography (petroleum/ethyl acetate = 20:1) afforded 0.0405 g (75%, d.r. > 20:1) of **3b** as a colorless solid. m.p.: 137.0–138.2 °C. Analytical data for **3b**: ^1^H NMR (400 MHz, CDCl_3_) *δ* 7.65–7.59 (m, 4H), 7.13 (dd, *J* = 8.8, 7.6 Hz, 2H), 6.79 (t, *J* = 7.4 Hz, 1H), 6.51 (d, *J* = 8.0 Hz, 2H), 5.42–5.31 (m, 1H), 5.15 (t, *J* = 8.0 Hz, 1H), 5.02 (dd, *J* = 10.0, 2.0 Hz, 1H), 4.90 (dd, *J* = 17.0, 1.4 Hz, 1H), 3.91 (s, 3H), 3.34 (dd, *J* = 14.2, 6.6 Hz, 1H), 3.16 (dd, *J* = 19.2, 8.4 Hz, 1H), 2.96 (dd, *J* = 14.2, 8.2 Hz, 1H), 2.70 (dd, *J* = 19.0, 7.4 Hz, 1H). ^13^C NMR (100 MHz, CDCl_3_) *δ* 206.9, 171.0, 148.8, 142.9, 133.1, 130.4, 129.2, 127.1, 121.5, 119.8, 118.7, 116.6, 111.6, 76.3, 59.3, 53.7, 46.4, 34.9. HRMS (ESI-TOF) *m/z*: [M + H]^+^ calcd for C_22_H_21_N_2_O_3_^+^ 361.1547; found 361.1543.
Methyl (2S*, 5R*)-2-allyl-3-oxo-1-phenyl-5-(4-(trifluoromethyl)phenyl)pyrrolidine-2-carboxylate **3c**. The title compound was prepared according to the general procedure using the corresponding diazo (0.0587 g, 0.15 mmol, 1.0 equiv), allyl carbonate (0.0290 g, 0.25 mmol 1.7 equiv), Rh_2_(OAc)_4_ (0.0015 g, 2.3 mol%), Pd_2_(dba)_3_ (0.0037 g, 2.7 mol%), and X-phos (0.0021 g, 3.0 mol%). Column chromatography (petroleum/ethyl acetate = 20:1) afforded 0.0423 g (70%, d.r. > 20:1) of **3c** as a colorless oil. Analytical data for **3c**: ^1^H NMR (400 MHz, CDCl_3_) *δ* 7.63 (d, *J* = 8.4 Hz, 2H), 7.57 (d, *J* = 8.4 Hz, 2H), 7.16–7.12 (m, 2H), 6.79 (t, *J* = 7.4 Hz, 1H), 6.55 (d, *J* = 8.0 Hz, 2H), 5.44–5.33 (m, 1H), 5.16 (t, *J* = 8.0 Hz, 1H), 5.03 (dd, *J* = 10.4, 1.6 Hz, 1H), 4.94–4.89 (m, 1H), 3.91 (s, 3H), 3.37 (dd, *J* = 14.0, 6.4 Hz, 1H), 3.17 (dd, *J* = 19.2, 8.4 Hz, 1H), 2.97 (dd, *J* = 14.4, 8.4 Hz, 1H), 2.73 (dd, *J* = 19.2, 7.6 Hz, 1H). ^13^C NMR (100 MHz, CDCl_3_) *δ* 207.4, 171.1, 147.4, 143.1, 130.5, 129.9 (q, *J*_C–F_ = 32.6 Hz), 129.2, 126.6, 126.2 (q, *J*_C–F_ = 3.5 Hz), 124.2 (d, *J*_C–F_ = 270.4 Hz), 121.4, 119.6, 116.6, 76.4, 59.3, 53.7, 46.7, 35.0. HRMS (ESI-TOF) *m/z*: [M + H]^+^ calcd for C_22_H_21_F_3_NO_3_^+^ 404.1468; found 404.1466.
Methyl (2S*,5R*)-2-allyl-3-oxo-1-phenyl-5-(p-tolyl)pyrrolidine-2-carboxylate **3d**. The title compound was prepared according to the general procedure using the Corresponding diazo (0.0506 g, 0.15 mmol, 1.0 equiv), allyl carbonate (0.0290 g, 0.25 mmol 1.7 equiv), Rh_2_(OAc)_4_ (0.0015 g, 2.3 mol%), Pd_2_(dba)_3_ (0.0037 g, 2.7 mol%), and X-phos (0.0021 g, 3.0 mol%). Column chromatography (petroleum/ethyl acetate = 20:1) afforded 0.0372 g (71%, d.r. > 20:1) of **3d** as a colorless oil. Analytical data for **3d**: ^1^H NMR (400 MHz, CDCl_3_) *δ* 7.37 (d, *J* = 8.4 Hz, 2H), 7.14–7.10 (m, 4H), 6.75 (t, *J* = 7.2 Hz, 1H), 6.59 (d, *J* = 8.0 Hz, 2H), 5.45–5.34 (m, 1H), 5.06 (t, *J* = 8.0 Hz, 1H), 5.01 (dd, *J* = 10.0, 2.0 Hz, 1H), 4.91 (dd, *J* = 17.2, 1.6 Hz, 1H), 3.90 (s, 3H), 3.38 (dd, *J* = 14.4, 6.4 Hz, 1H), 3.13 (dd, *J* = 19.2, 8.4 Hz, 1H), 2.95 (dd, *J* = 14.0, 8.4 Hz, 1H), 2.76 (dd, *J* = 19.2, 7.6 Hz, 1H), 2.30 (s, 3H). ^13^C NMR (100 MHz, CDCl_3_) *δ* 208.3, 171.4, 143.6, 140.2, 137.1, 130.7, 129.8, 128.9, 126.1, 121.1, 119.1, 116.7, 76.4, 59.5, 53.5, 47.3, 35.0, 21.2. HRMS (ESI-TOF) *m/z*: [M + H]^+^ calcd for C_22_H_24_NO_3_^+^ 350.1751; found 350.1747.
Methyl (2S*,5R*)-2-allyl-5-(4-chlorophenyl)-3-oxo-1-phenylpyrrolidine-2-carboxylate **3e**. The title compound was prepared according to the general procedure using the corresponding diazo (0.0536 g, 0.15 mmol, 1.0 equiv), allyl carbonate (0.0290 g, 0.25 mmol 1.7 equiv), Rh_2_(OAc)_4_ (0.0015 g, 2.3 mol%), Pd_2_(dba)_3_ (0.0037 g, 2.7 mol%), and X-phos (0.0021 g, 3.0 mol%). Column chromatography (petroleum/ethyl acetate = 20:1) afforded 0.0432 g (78%, d.r. > 20:1) of **3e** as a colorless oil. Analytical data for **3e**: ^1^H NMR (400 MHz, CDCl_3_) *δ* 7.43 (d, *J* = 8.4 Hz, 2H), 7.27 (d, *J* = 8.4 Hz, 2H), 7.13 (dd, *J* = 8.8, 7.6 Hz, 2H), 6.77 (t, *J* = 7.4 Hz, 1H), 6.54 (d, *J* = 8.0 Hz, 2H), 5.42–5.32 (m, 1H), 5.07 (t, *J* = 8.0 Hz, 1H), 5.01 (dd, *J* = 10.4, 2.0 Hz, 1H), 4.90 (dd, *J* = 17.2, 1.6 Hz, 1H), 3.90 (s, 3H), 3.35 (dd, *J* = 14.2, 6.6 Hz, 1H), 3.13 (dd, *J* = 19.2, 8.0 Hz, 1H), 2.95 (dd, *J* = 14.0, 8.4 Hz, 1H), 2.72 (dd, *J* = 19.2, 7.6 Hz, 1H). ^13^C NMR (100 MHz, CDCl_3_) *δ* 207.7, 171.2, 143.2, 141.8, 133.2, 130.5, 129.4, 129.1, 127.7, 121.3, 119.5, 116.7, 76.4, 59.1, 53.6, 46.9, 35.0. HRMS (ESI-TOF) *m/z*: [M + H]^+^ calcd for C_21_H_21_ClNO_3_ 370.1204; found 370.1201.
Methyl (2S*,5R*)-2-allyl-5-(4-methoxyphenyl)-3-oxo-1-phenylpyrrolidine-2-carboxylate**3f.** The title compound was prepared according to the general procedure using the corresponding diazo (0.0530 g, 0.15 mmol, 1.0 equiv), allyl carbonate (0.0290 g, 0.25 mmol 1.7 equiv), Rh_2_(OAc)_4_ (0.0015 g, 2.3 mol%), Pd_2_(dba)_3_ (0.0037 g, 2.7 mol%), and X-phos (0.0021 g, 3.0 mol%). Column chromatography (petroleum/ethyl acetate = 20:1) afforded 0.0422 g (77%, d.r. > 20:1) of **3f** as a colorless oil. Analytical data for **3f**: ^1^H NMR (400 MHz, CDCl_3_) *δ* 7.40 (d, *J* = 8.8 Hz, 2H), 7.14–7.09 (m, 2H), 6.84 (d, *J* = 8.8 Hz, 2H), 6.75 (t, *J* = 7.2 Hz, 1H), 6.58 (d, *J* = 8.0 Hz, 2H), 5.44–5.33 (m, 1H), 5.06–4.99 (m, 2H), 4.90 (d, *J* = 17.2 Hz, 1H), 3.90 (s, 3H), 3.76 (s, 3H), 3.36 (dd, *J* = 14.2, 6.6 Hz, 1H), 3.11 (dd, *J* = 19.2, 8.4 Hz, 1H), 2.94 (dd, *J* = 14.0, 8.4 Hz, 1H), 2.75 (dd, *J* = 19.2, 7.6 Hz, 1H). ^13^C NMR (100 MHz, CDCl_3_) *δ* 208.4, 171.4, 158.9, 143.6, 135.1, 130.7, 128.9, 127.4, 121.1, 119.1, 116.8, 114.5, 76.4, 59.2, 55.3, 53.5, 47.3, 35.0. HRMS (ESI-TOF) *m/z*: [M + H]^+^ calcd for C_22_H_24_NO_4_^+^ 366.1700; found 366.1699.
Methyl (2S*,5R*)-2-allyl-5-(4-(tert-butyl)phenyl)-3-oxo-1-phenylpyrrolidine-2-carboxylate **3g**. The title compound was prepared according to the general procedure using the Corresponding diazo (0.0569 g, 0.15 mmol, 1.0 equiv), allyl carbonate (0.0290 g, 0.25 mmol 1.7 equiv), Rh_2_(OAc)_4_ (0.0015 g, 2.3 mol%), Pd_2_(dba)_3_ (0.0037 g, 2.7 mol%), and X-phos (0.0021 g, 3.0 mol%). Column chromatography (petroleum/ethyl acetate = 20:1) afforded 0.0510 g (87%, d.r. > 20:1) of **3g** as a colorless oil. Analytical data for **3g**: ^1^H NMR (400 MHz, CDCl_3_) *δ* 7.41 (d, *J* = 8.4 Hz, 2H), 7.33 (d, *J* = 8.4 Hz, 2H), 7.16–7.11 (m, 2H), 6.76 (t, *J* = 7.4 Hz, 1H), 6.61 (d, *J* = 7.6 Hz, 2H), 5.46–5.34 (m, 1H), 5.08 (t, *J* = 8.0 Hz, 1H), 5.02 (dd, *J* = 10.4, 1.6 Hz, 1H), 4.92 (d, *J* = 16.8 Hz, 1H), 3.91 (s, 3H), 3.40 (dd, *J* = 14.0, 6.4 Hz, 1H), 3.14 (dd, *J* = 19.2, 8.4 Hz, 1H), 2.96 (dd, *J* = 14.0, 8.4 Hz, 1H), 2.79 (dd, *J* = 19.4, 7.4 Hz, 1H), 1.29 (s, 9H). ^13^C NMR (100 MHz, CDCl_3_) *δ* 208.4, 171.4, 150.3, 143.7, 140.1, 130.7, 128.9, 126.0, 125.8, 121.0, 119.0, 116.6, 76.4, 59.4, 53.5, 47.2, 35.0, 34.6, 31.5. HRMS (ESI-TOF) *m/z*: [M + H]^+^ calcd for C_25_H_30_NO_3_^+^ 392.2220; found 392.2217.
Methyl (2S*,5R*)-2-allyl-3-oxo-1-phenyl-5-(m-tolyl)pyrrolidine-2-carboxylate **3h**. The title compound was prepared according to the general procedure using the corresponding diazo (0.0506 g, 0.15 mmol, 1.0 equiv), allyl carbonate (0.0290 g, 0.25 mmol 1.7 equiv), Rh_2_(OAc)_4_ (0.0015 g, 2.3 mol%), Pd_2_(dba)_3_ (0.0037 g, 2.7 mol%), and X-phos (0.0021 g, 3.0 mol%). Column chromatography (petroleum/ethyl acetate = 20:1) afforded 0.0435 g (83%, d.r. > 20:1) of **3h** as a colorless oil. Analytical data for **3h**: ^1^H NMR (400 MHz, CDCl_3_) *δ* 7.29 (d, *J* = 6.0 Hz, 2H), 7.18 (t, *J* = 7.8 Hz, 1H), 7.14–7.09 (m, 2H), 7.03 (d, *J* = 7.6 Hz, 1H), 6.75 (t, *J* = 7.4 Hz, 1H), 6.59 (d, *J* = 8.4 Hz, 2H), 5.44–5.33 (m, 1H), 5.06–4.99 (m, 2H), 4.91 (d, *J* = 17.2 Hz, 1H), 3.90 (s, 3H), 3.38 (dd, *J* = 14.2, 6.6 Hz, 1H), 3.12 (dd, *J* = 19.2, 8.4 Hz, 1H), 2.94 (dd, *J* = 14.2, 8.6 Hz, 1H), 2.76 (dd, *J* = 19.2, 7.6 Hz, 1H), 2.31 (s, 3H). ^13^C NMR (100 MHz, CDCl_3_) *δ* 208.3, 171.4, 143.7, 143.3, 138.6, 130.7, 129.0, 128.9, 128.3, 127.0, 123.2, 121.1, 119.1, 116.6, 76.4, 59.8, 53.5, 47.2, 35.0, 21.7. HRMS (ESI-TOF) *m/z*: [M + H]^+^ calcd for C_22_H_24_NO_3_^+^ 350.1751; found 350.1749.
Methyl (2S*,5R*)-2-allyl-5-(3-bromophenyl)-3-oxo-1-phenylpyrrolidine-2-carboxylate **3i**. The title compound was prepared according to the general procedure using the Corresponding diazo (0.0602 g, 0.15 mmol, 1.0 equiv), allyl carbonate (0.0290 g, 0.25 mmol 1.7 equiv), Rh_2_(OAc)_4_ (0.0015 g, 2.3 mol%), Pd_2_(dba)_3_ (0.0037 g, 2.7 mol%), and X-phos (0.0021 g, 3.0 mol%). Column chromatography (petroleum/ethyl acetate = 20:1) afforded 0.0527 g (85%, d.r. > 20:1) of **3i** as a colorless oil. Analytical data for **3i**: ^1^H NMR (400 MHz, CDCl_3_) *δ* 7.67 (s, 1H), 7.44 (d, *J* = 7.6 Hz, 1H), 7.37–7.34 (m, 1H), 7.20–7.12 (m, 1H), 6.78 (t, *J* = 7.2 Hz, 1H), 6.56 (d, *J* = 8.0 Hz, 2H), 5.42–5.31 (m, 1H), 5.05 (t, *J* = 8.0 Hz, 1H), 5.01 (dd, *J* = 10.4, 1.2 Hz, 1H), 4.90 (d, *J* = 17.2 Hz, 1H), 3.91 (s, 3H), 3.36 (dd, *J* = 14.2, 6.6 Hz, 1H), 3.14 (dd, *J* = 19.4, 8.6 Hz, 1H), 2.94 (dd, *J* = 14.4, 8.4 Hz, 1H), 2.73 (dd, *J* = 19.2, 7.2 Hz, 1H). ^13^C NMR (100 MHz, CDCl_3_) *δ* 207.6, 171.2, 145.9, 143.2, 130.8, 130.8, 130.5, 129.5, 129.2, 124.9, 123.2, 121.3, 119.5, 116.5, 76.4, 59.2, 53.6, 46.8, 35.0. HRMS (ESI-TOF) *m/z*: [M + H]^+^ calcd for C_21_H_21_BrNO_3_^+^ 414.0699; found 414.0696.
Methyl (2S*,5R*)-2-allyl-3-oxo-1-phenyl-5-(3-(trifluoromethyl)phenyl)pyrrolidine-2-carboxylate **3j**. The title compound was prepared according to the general procedure using the corresponding diazo (0.0587 g, 0.15 mmol, 1.0 equiv), allyl carbonate (0.0290 g, 0.25 mmol 1.7 equiv), Rh_2_(OAc)_4_ (0.0015 g, 2.3 mol%), Pd_2_(dba)_3_ (0.0037 g, 2.7 mol%), and X-phos (0.0021 g, 3.0 mol%). Column chromatography (petroleum/ethyl acetate = 20:1) afforded 0.0496 g (82%, d.r. = 9:1) of **3j** as a colorless oil. Analytical data for **3j**: ^1^H NMR (400 MHz, CDCl_3_) *δ* 7.80 (s, 1H), 7.71 (d, *J* = 7.6 Hz, 1H), 7.49 (d, *J* = 8.0 Hz, 1H), 7.43 (t, *J* = 7.8 Hz, 1H), 7.13 (t, *J* = 8.2 Hz, 2H), 6.78 (t, *J* = 7.2 Hz, 1H), 6.55 (d, *J* = 8.0 Hz, 2H), 5.44–5.33 (m, 1H), 5.18 (t, *J* = 8.0 Hz, 1H), 5.02 (d, *J* = 10.8 Hz, 1H), 4.91 (d, *J* = 16.8 Hz, 1H), 3.91 (s, 3H), 3.37 (dd, *J* = 14.0, 6.8 Hz, 1H), 3.18 (dd, *J* = 19.2, 8.8 Hz, 1H), 2.95 (dd, *J* = 14.4, 8.4 Hz, 1H), 2.73 (dd, *J* = 19.2, 7.2 Hz, 1H). ^13^C NMR (100 MHz, CDCl_3_) *δ* 207.5, 171.2, 144.6, 143.0, 131.5 (d, *J*_C–F_ = 32.0 Hz), 130.5, 129.7, 129.7, 129.2, 124.6 (q, *J*_C–F_ = 3.3 Hz), 123.2 (q, *J*_C–F_ = 3.8 Hz), 121.4, 119.6, 116.5, 76.4, 59.2, 53.6, 46.8, 35.0. HRMS (ESI-TOF) *m/z*: [M + H]^+^ calcd for C_22_H_21_F_3_NO_3_^+^ 404.1468; found 404.1465.
Methyl (2S*,5R*)-2-allyl-5-(3-methoxyphenyl)-3-oxo-1-phenylpyrrolidine-2-carboxylate **3k**. The title compound was prepared according to the general procedure using the corresponding diazo (0.0530 g, 0.15 mmol, 1.0 equiv), allyl carbonate (0.0290 g, 0.25 mmol 1.7 equiv), Rh_2_(OAc)_4_ (0.0015 g, 2.3 mol%), Pd_2_(dba)_3_ (0.0037 g, 2.7 mol%), and X-phos (0.0021 g, 3.0 mol%). Column chromatography (petroleum/ethyl acetate = 20:1) afforded 0.0433 g (79%, d.r. > 20:1) of **3k** as a colorless oil. Analytical data for **3k**: ^1^H NMR (400 MHz, CDCl_3_) *δ* 7.22 (t, *J* = 8.0 Hz, 1H), 7.14–7.05 (m, 4H), 6.77–6.73 (m, 2H), 6.58 (d, *J* = 8.0 Hz, 2H), 5.44–5.32 (m, 1H), 5.06–4.99 (m, 2H), 4.91 (d, *J* = 17.2 Hz, 1H), 3.90 (s, 3H), 3.75 (s, 3H), 3.37 (dd, *J* = 14.0, 6.4 Hz, 1H), 3.13 (dd, *J* = 19.2, 8.4 Hz, 1H), 2.94 (dd, *J* = 14.2, 8.4 Hz, 1H), 2.77 (dd, *J* = 19.2, 7.6 Hz, 1H). ^13^C NMR (100 MHz, CDCl_3_) *δ* 208.2, 171.3, 160.3, 145.1, 143.6, 130.7, 130.2, 129.0, 121.2, 119.2, 118.6, 116.5, 112.9, 111.7, 76.4, 59.7, 55.2, 53.6, 47.1, 35.1. HRMS (ESI-TOF) *m/z*: [M + H]^+^ calcd for C_22_H_24_NO_4_^+^ 366.1700; found 366.1698.
Methyl (2S*,5R*)-2-allyl-5-(2-nitrophenyl)-3-oxo-1-phenylpyrrolidine-2-carboxylate **3l**. The title compound was prepared according to the general procedure using the corresponding diazo (0.0552 g, 0.15 mmol, 1.0 equiv), allyl carbonate (0.0290 g, 0.25 mmol 1.7 equiv), Rh_2_(OAc)_4_ (0.0015 g, 2.3 mol%), Pd_2_(dba)_3_ (0.0037 g, 2.7 mol%), and X-phos (0.0021 g, 3.0 mol%). Column chromatography (petroleum/ethyl acetate = 20:1) afforded 0.0462 g (81%, d.r. = 9:1) of **3l** as a colorless oil. Analytical data for **3l**: ^1^H NMR (400 MHz, CDCl_3_) *δ* 8.14 (dd, *J* = 8.0, 1.6 Hz, 1H), 8.00 (dd, *J* = 8.2, 1.4 Hz, 1H), 7.52 (td, *J* = 7.6, 1.2 Hz, 1H), 7.39 (td, *J* = 8.0, 1.2 Hz, 1H), 7.12 (dd, *J* = 8.8, 7.6 Hz, 2H), 6.78 (t, *J* = 7.4 Hz, 1H), 6.44 (d, *J* = 8.0 Hz, 2H), 5.64 (dd, *J* = 9.0, 7.0 Hz, 1H), 5.43–5.31 (m, 1H), 5.05 (dd, *J* = 10.2, 1.8 Hz, 1H), 4.91 (dd, *J* = 17.2, 1.6 Hz, 1H), 3.93 (s, 3H), 3.49 (dd, *J* = 19.6, 8.8 Hz, 1H), 3.36 (dd, *J* = 14.2, 6.6 Hz, 1H), 2.97 (dd, *J* = 14.2, 8.2 Hz, 1H), 2.75 (dd, *J* = 19.8, 7.0 Hz, 1H). ^13^C NMR (100 MHz, CDCl_3_) *δ* 207.6, 171.0, 148.7, 142.8, 138.8, 134.7, 130.1, 129.3, 128.5, 128.3, 125.0, 121.7, 119.8, 116.2, 76.5, 55.7, 53.7, 45.9, 35.2. HRMS (ESI-TOF) *m/z*: [M + H]^+^ calcd for C_21_H_21_N_2_O_5_^+^ 381.1445; found 381.1447.
Methyl (2S*,5R*)-2-allyl-5-(2-methoxyphenyl)-3-oxo-1-phenylpyrrolidine-2-carboxylate **3m**. The title compound was prepared according to the general procedure using the corresponding diazo (0.0530 g, 0.15 mmol, 1.0 equiv), allyl carbonate (0.0290 g, 0.25 mmol 1.7 equiv), Rh_2_(OAc)_4_ (0.0015 g, 2.3 mol%), Pd_2_(dba)_3_ (0.0037 g, 2.7 mol%), and X-phos (0.0021 g, 3.0 mol%). Column chromatography (petroleum/ethyl acetate = 20:1) afforded 0.0449 g (82%, d.r. > 20:1) of **3m** as a colorless oil. Analytical data for **3m**: ^1^H NMR (400 MHz, CDCl_3_) *δ* 7.54 (dd, *J* = 7.8, 1.8 Hz, 1H), 7.20 (td, *J* = 8.4, 1.6 Hz, 1H), 7.16–7.11 (m, 2H), 6.91 (d, *J* = 8.0 Hz, 1H), 6.84 (t, *J* = 7.4 Hz, 1H), 6.75 (t, *J* = 7.4 Hz, 1H), 6.55 (d, *J* = 8.0 Hz, 2H), 5.49–5.36 (m, 1H), 5.04 (dd, *J* = 10.0, 2.0 Hz, 1H), 4.93 (d, *J* = 17.2 Hz, 1H), 3.93 (s, 3H), 3.87 (s, 3H), 3.44 (dd, *J* = 14.2, 6.2 Hz, 1H), 3.25 (dd, *J* = 19.6, 8.8 Hz, 1H), 2.94 (dd, *J* = 14.0, 8.4 Hz, 1H), 2.64 (dd, *J* = 19.6, 6.8 Hz, 1H). ^13^C NMR (100 MHz, CDCl_3_) *δ* 209.2, 171.5, 156.5, 143.7, 130.7, 130.7, 129.0, 128.2, 126.7, 121.2, 121.0, 118.8, 116.1, 110.2, 76.1, 55.5, 53.4, 53.1, 45.4, 35.1. HRMS (ESI-TOF) *m/z*: [M + H]^+^ calcd for C_22_H_24_NO_4_^+^ 366.1700; found 366.1697.
Methyl (2S*,5R*)-2-allyl-3-oxo-1-phenyl-5-(o-tolyl)pyrrolidine-2-carboxylate **3n**. The title compound was prepared according to the general procedure using the corresponding diazo (0.0506 g, 0.15 mmol, 1.0 equiv), allyl carbonate (0.0290 g, 0.25 mmol 1.7 equiv), Rh_2_(OAc)_4_ (0.0015 g, 2.3 mol%), Pd_2_(dba)_3_ (0.0037 g, 2.7 mol%), and X-phos (0.0021 g, 3.0 mol%). Column chromatography (petroleum/ethyl acetate = 20:1) afforded 0.0445 g (85%, d.r. > 20:1) of **3n** as a colorless oil. Analytical data for **3n**: ^1^H NMR (400 MHz, CDCl_3_) *δ* 7.65 (dd, *J* = 7.4, 1.8 Hz, 1H), 7.17 (d, *J* = 7.2 Hz, 1H), 7.15–7.12 (m, 2H), 7.12–7.07 (m, 2H), 6.76 (t, *J* = 7.2 Hz, 1H), 6.48 (d, *J* = 8.0 Hz, 2H), 5.48–5.36 (m, 1H), 5.24 (t, *J* = 8.0 Hz, 1H), 5.04 (dd, *J* = 10.0, 2.0 Hz, 1H), 4.97–4.91 (m, 1H), 3.90 (s, 3H), 3.44 (dd, *J* = 14.4, 6.4 Hz, 1H), 3.20 (dd, *J* = 19.2, 8.4 Hz, 1H), 2.97 (dd, *J* = 14.0, 8.4 Hz, 1H), 2.66 (dd, *J* = 19.2, 7.6 Hz, 1H), 2.46 (s, 3H). ^13^C NMR (100 MHz, CDCl_3_) *δ* 208.4, 171.3, 143.5, 140.8, 134.0, 130.7, 130.6, 129.0, 127.2, 127.2, 125.3, 121.1, 119.1, 116.2, 76.3, 56.1, 53.5, 45.1, 35.1, 19.5. HRMS (ESI-TOF) *m/z*: [M + H]^+^ calcd for C_22_H_24_NO_3_ 350.1750; found 350.1745.
Methyl (2S*,5R*)-2-allyl-5-(2-bromophenyl)-3-oxo-1-phenylpyrrolidine-2-carboxylate **3o**. The title compound was prepared according to the general procedure using the corresponding diazo (0.0602 g, 0.15 mmol, 1.0 equiv), allyl carbonate (0.0290 g, 0.25 mmol 1.7 equiv), Rh_2_(OAc)_4_ (0.0015 g, 2.3 mol%), Pd_2_(dba)_3_ (0.0037 g, 2.7 mol%), and X-phos (0.0021 g, 3.0 mol%). Column chromatography (petroleum/ethyl acetate = 20:1) afforded 0.0514 g (83%, d.r. = 10:1) of **3o** as a colorless oil. Analytical data for **3o**: ^1^H NMR (400 MHz, CDCl_3_) *δ* 7.71 (dd, *J* = 7.8, 1.8 Hz, 1H), 7.56 (dd, *J* = 8.0, 1.2 Hz, 1H), 7.19 (td, *J* = 7.6, 1.6 Hz, 1H), 7.14 (dd, *J* = 8.8, 7.2 Hz, 2H), 7.09 (td, *J* = 7.6, 1.6 Hz, 1H), 6.78 (t, *J* = 7.2 Hz, 1H), 6.48 (d, *J* = 8.0 Hz, 2H), 5.47–5.34 (m, 2H), 5.06 (dd, *J* = 10.2, 1.8 Hz, 1H), 4.93 (dd, *J* = 17.0, 1.4 Hz, 1H), 3.90 (s, 3H), 3.42–3.36 (m, 1H), 3.33 (dd, *J* = 19.4, 8.6 Hz, 1H), 2.95 (dd, *J* = 14.4, 8.4 Hz, 1H), 2.62 (dd, *J* = 19.2, 7.2 Hz, 1H). ^13^C NMR (101 MHz, CDCl_3_) *δ* 207.9, 171.1, 143.2, 141.6, 132.9, 130.4, 129.2, 129.0, 128.5, 127.7, 122.5, 121.4, 119.4, 116.4, 76.5, 58.7, 53.6, 44.8, 35.1. HRMS (ESI-TOF) *m/z*: [M + H]^+^ calcd for C_21_H_21_BrNO_3_^+^ 414.0699; found 414.0691.
Methyl (2S*,5R*)-2-allyl-3-oxo-1-phenyl-5-(3,4,5-trimethoxyphenyl)pyrrolidine-2-carboxylate **3p**. The title compound was prepared according to the general procedure using the corresponding diazo (0.0620 g, 0.15 mmol, 1.0 equiv), allyl carbonate (0.0290 g, 0.25 mmol 1.7 equiv), Rh_2_(OAc)_4_ (0.0015 g, 2.3 mol%), Pd_2_(dba)_3_ (0.0037 g, 2.7 mol%), and X-phos (0.0021 g, 3.0 mol%). Column chromatography (petroleum/ethyl acetate = 20:1) afforded 0.0446 g (70%, d.r. > 20:1) of **3p** as a colorless oil. Analytical data for **3p**: ^1^H NMR (400 MHz, CDCl_3_) *δ* 7.13 (dd, *J* = 8.8, 7.2 Hz, 2H), 6.79 (s, 2H), 6.76 (d, *J* = 7.2 Hz, 1H), 6.58 (d, *J* = 8.0 Hz, 2H), 5.43–5.31 (m, 1H), 5.02–4.97 (m, 2H), 4.93–4.88 (m, 1H), 3.89 (s, 3H), 3.80 (s, 3H), 3.79 (s, 6H), 3.37 (dd, *J* = 14.0, 6.4 Hz, 1H), 3.13 (dd, *J* = 19.4, 8.6 Hz, 1H), 2.94 (dd, *J* = 14.0, 8.4 Hz, 1H), 2.75 (dd, *J* = 19.6, 7.2 Hz, 1H). ^13^C NMR (100 MHz, CDCl_3_) *δ* 208.1, 171.2, 153.8, 143.5, 139.2, 137.0, 130.7, 129.2, 121.1, 119.2, 116.4, 102.8, 76.5, 60.9, 59.8, 56.1, 53.6, 47.1, 35.2. HRMS (ESI-TOF) *m/z*: [M + H]^+^ calcd for C_24_H_28_NO_6_^+^ 426.1911; found 426.1902.
Methyl (2S*,5R*)-2-allyl-5-(2,4-dichlorophenyl)-3-oxo-1-phenylpyrrolidine-2-carboxylate **3q**. The title compound was prepared according to the general procedure using the corresponding diazo (0.0587 g, 0.15 mmol, 1.0 equiv), allyl carbonate (0.0290 g, 0.25 mmol 1.7 equiv), Rh_2_(OAc)_4_ (0.0015 g, 2.3 mol%), Pd_2_(dba)_3_ (0.0037 g, 2.7 mol%), and X-phos (0.0021 g, 3.0 mol%). Column chromatography (petroleum/ethyl acetate = 20:1) afforded 0.0502 g (83%, d.r. > 20:1) of **3q** as a colorless oil. Analytical data for **3q**: ^1^H NMR (400 MHz, CDCl_3_) *δ* 7.67 (d, *J* = 8.4 Hz, 1H), 7.40 (d, *J* = 2.0 Hz, 1H), 7.18–7.11 (m, 3H), 6.80 (t, *J* = 7.4 Hz, 1H), 6.48 (d, *J* = 8.4 Hz, 2H), 5.47–5.32 (m, 2H), 5.05 (dd, *J* = 10.0, 2.0 Hz, 1H), 4.94–4.89 (m, 1H), 3.89 (s, 3H), 3.38 (dd, *J* = 14.0, 6.8 Hz, 1H), 3.29 (dd, *J* = 19.2, 8.8 Hz, 1H), 2.95 (dd, *J* = 14.0, 8.4 Hz, 1H), 2.60 (dd, *J* = 19.4, 7.0 Hz, 1H). ^13^C NMR (101 MHz, CDCl_3_) *δ* 207.5, 171.0, 142.9, 138.8, 133.7, 133.0, 130.3, 129.4, 129.3, 128.6, 128.3, 121.5, 119.7, 116.3, 76.3, 55.8, 53.6, 44.6, 35.1. HRMS (ESI-TOF) *m/z*: [M + H]^+^ calcd for C_21_H_20_Cl_2_NO_3_^+^ 404.0815; found 404.0808.
Methyl(2S*,5R*)-2-allyl-5-(2,3-dihydrobenzo[b][1,4]dioxin-6-yl)-3-oxo-1-phenylpyrrolidine-2-carboxylate **3r**. The title compound was prepared according to the general procedure using the corresponding diazo (0.0572 g, 0.15 mmol, 1.0 equiv), allyl carbonate (0.0290 g, 0.25 mmol 1.7 equiv), Rh_2_(OAc)_4_ (0.0015 g, 2.3 mol%), Pd_2_(dba)_3_ (0.0037 g, 2.7 mol%), and X-phos (0.0021 g, 3.0 mol%). Column chromatography (petroleum/ethyl acetate = 20:1) afforded 0.0519 g (88%, d.r. > 20:1) of **3r** as a colorless oil. Analytical data for **3r**: ^1^H NMR (400 MHz, CDCl_3_) *δ* 7.13 (t, *J* = 8.0 Hz, 2H), 7.03 (d, *J* = 2.0 Hz, 1H), 6.94 (dd, *J* = 8.4, 2.0 Hz, 1H), 6.77 (dd, *J* = 15.0, 7.8 Hz, 2H), 6.59 (d, *J* = 8.4 Hz, 2H), 5.42–5.31 (m, 1H), 5.01–4.94 (m, 2H), 4.89 (d, *J* = 16.8 Hz, 1H), 4.20 (s, 4H), 3.89 (s, 3H), 3.35 (dd, *J* = 14.4, 6.4 Hz, 1H), 3.09 (dd, *J* = 19.2, 8.4 Hz, 1H), 2.92 (dd, *J* = 14.0, 8.4 Hz, 1H), 2.74 (dd, *J* = 19.4, 7.4 Hz, 1H). ^13^C NMR (100 MHz, CDCl_3_) *δ* 208.3, 171.4, 144.0, 143.6, 142.9, 136.6, 130.7, 128.9, 121.1, 119.2, 119.1, 117.8, 116.7, 115.0, 76.4, 64.4, 59.2, 53.5, 47.2, 35.0. HRMS (ESI-TOF) *m/z*: [M + H]^+^ calcd for C_23_H_24_NO_5_^+^ 394.1649; found 394.1646.
Methyl (2S*,5R*)-2-allyl-5-(furan-2-yl)-3-oxo-1-phenylpyrrolidine-2-carboxylate **3s**. The title compound was prepared according to the general procedure using the corresponding diazo (0.0470 g, 0.15 mmol, 1.0 equiv), allyl carbonate (0.0290 g, 0.25 mmol 1.7 equiv), Rh_2_(OAc)_4_ (0.0015 g, 2.3 mol%), Pd_2_(dba)_3_ (0.0037 g, 2.7 mol%), and X-phos (0.0021 g, 3.0 mol%). Column chromatography (petroleum/ethyl acetate = 20:1) afforded 0.0444 g (91%, d.r. > 20:1) of **3s** as a colorless solid. m.p.: 68.6–70.3 °C. Analytical data for **3s**: ^1^H NMR (400 MHz, CDCl_3_) *δ* 7.33 (dd, *J* = 2.0, 0.8 Hz, 1H), 7.19 (dd, *J* = 8.6, 7.4 Hz, 2H), 6.80 (t, *J* = 7.4 Hz, 1H), 6.65 (d, *J* = 8.0 Hz, 2H), 6.25 (dd, *J* = 3.4, 1.8 Hz, 1H), 6.22 (d, *J* = 3.2 Hz, 1H), 5.44–5.37 (m, 1H), 5.32 (dd, *J* = 8.8, 5.2 Hz, 1H), 5.02 (dd, *J* = 10.2, 1.8 Hz, 1H), 4.95–4.90 (m, 1H), 3.80 (s, 3H), 3.37 (dd, *J* = 14.4, 6.4 Hz, 1H), 3.09 (dd, *J* = 19.2, 9.2 Hz, 1H), 3.00–2.86 (m, 2H). ^13^C NMR (100 MHz, CDCl_3_) *δ* 207.9, 171.0, 154.8, 143.3, 141.7, 130.7, 129.1, 121.0, 119.1, 115.4, 110.7, 107.0, 75.4, 53.4, 52.7, 43.7, 35.1. HRMS (ESI-TOF) *m/z*: [M + H]^+^ calcd for C_19_H_20_NO_4_^+^ 326.1381; found 326.1382.
Methyl (2S*,5R*)-2-allyl-3-oxo-1-phenyl-5-(thiophen-2-yl)pyrrolidine-2-carboxylate **3t**. The title compound was prepared according to the general procedure using the corresponding diazo (0.0494 g, 0.15 mmol, 1.0 equiv), allyl carbonate (0.0290 g, 0.25 mmol 1.7 equiv), Rh_2_(OAc)_4_ (0.0015 g, 2.3 mol%), Pd_2_(dba)_3_ (0.0037 g, 2.7 mol%), and X-phos (0.0021 g, 3.0 mol%). Column chromatography (petroleum/ethyl acetate = 20:1) afforded 0.0425 g (83%, d.r. > 20:1) of **3t** as a colorless oil. Analytical data for **3t**: ^1^H NMR (400 MHz, CDCl_3_) *δ* 7.19–7.14 (m, 3H), 7.10–7.08 (m, 1H), 6.90 (dd, *J* = 5.0, 3.4 Hz, 1H), 6.80 (t, *J* = 7.4 Hz, 1H), 6.69 (d, *J* = 8.0 Hz, 2H), 5.46 (dd, *J* = 8.6, 7.0 Hz, 1H), 5.42–5.31 (m, 1H), 5.01 (dd, *J* = 10.0, 2.0 Hz, 1H), 4.93–4.87 (m, 1H), 3.88 (s, 3H), 3.33 (dd, *J* = 14.0, 6.4 Hz, 1H), 3.17 (dd, *J* = 19.2, 8.4 Hz, 1H), 2.97–2.88 (m, 2H). ^13^C NMR (100 MHz, CDCl_3_) *δ* 207.8, 171.2, 147.7, 143.4, 130.6, 129.0, 126.8, 124.9, 124.8, 121.2, 119.6, 116.5, 76.1, 55.2, 53.4, 47.2, 34.8. HRMS (ESI-TOF) *m/z*: [M + H]^+^ calcd for C_19_H_20_NO_3_S^+^ 342.1158; found 342.1156.
Methyl (2S*,5R*)-2-allyl-5-(naphthalen-2-yl)-3-oxo-1-phenylpyrrolidine-2-carboxylate **3u**. The title compound was prepared according to the general procedure using the corresponding diazo (0.0560 g, 0.15 mmol, 1.0 equiv), allyl carbonate (0.0290 g, 0.25 mmol 1.7 equiv), Rh_2_(OAc)_4_ (0.0015 g, 2.3 mol%), Pd_2_(dba)_3_ (0.0037 g, 2.7 mol%), and X-phos (0.0021 g, 3.0 mol%). Column chromatography (petroleum/ethyl acetate = 20:1) afforded 0.0462 g (80%, d.r. > 20:1) of **3u** as a colorless oil. Analytical data for **3u**: ^1^H NMR (400 MHz, CDCl_3_) *δ* 7.93 (s, 1H), 7.82–7.77 (m, 3H), 7.67 (dd, *J* = 8.4, 2.0 Hz, 1H), 7.49–7.42 (m, 2H), 7.11–7.06 (m, 2H), 6.73 (t, *J* = 7.2 Hz, 1H), 6.65 (d, *J* = 8.0 Hz, 2H), 5.50–5.38 (m, 1H), 5.26 (t, *J* = 8.0 Hz, 1H), 5.05 (dd, *J* = 10.2, 1.8 Hz, 1H), 4.97–4.91 (m, 1H), 3.96 (s, 3H), 3.41 (dd, *J* = 14.0, 6.4 Hz, 1H), 3.20 (dd, *J* = 19.2, 8.4 Hz, 1H), 2.99 (dd, *J* = 14.0, 8.4 Hz, 1H), 2.85 (dd, *J* = 19.2, 7.6 Hz, 1H). ^13^C NMR (100 MHz, CDCl_3_) *δ* 208.1, 171.4, 143.6, 140.8, 133.6, 133.1, 130.7, 129.4, 129.0, 127.9, 127.9, 126.3, 126.0, 125.4, 123.9, 121.2, 119.3, 116.7, 76.5, 60.0, 53.6, 47.1, 35.1. HRMS (ESI-TOF) *m/z*: [M + H]^+^ calcd for C_25_H_24_NO_3_^+^ 386.1750; found 386.1747.
Methyl (2S*,5R*)-2-allyl-3-oxo-5-phenyl-1-(p-tolyl)pyrrolidine-2-carboxylate **3v**. The title compound was prepared according to the general procedure using the corresponding diazo (0.0506 g, 0.15 mmol, 1.0 equiv), allyl carbonate (0.0290 g, 0.25 mmol 1.7 equiv), Rh_2_(OAc)_4_ (0.0015 g, 2.3 mol%), Pd_2_(dba)_3_ (0.0037 g, 2.7 mol%), and X-phos (0.0021 g, 3.0 mol%). Column chromatography (petroleum/ethyl acetate = 20:1) afforded 0.0414 g (79%, d.r. = 15:1) of **3v** as a colorless oil. Analytical data for **3v**: ^1^H NMR (400 MHz, CDCl_3_) *δ* 7.48 (d, *J* = 7.2 Hz, 2H), 7.30 (t, *J* = 7.6 Hz, 2H), 7.22 (t, *J* = 7.4 Hz, 1H), 6.92 (d, *J* = 8.4 Hz, 2H), 6.49 (d, *J* = 8.4 Hz, 2H), 5.46–5.35 (m, 1H), 5.09–5.00 (m, 2H), 4.94 (d, *J* = 18.0 Hz, 1H), 3.90 (s, 3H), 3.35 (dd, *J* = 14.0, 6.4 Hz, 1H), 3.12 (dd, *J* = 19.2, 8.4 Hz, 1H), 2.93 (dd, *J* = 14.0, 8.4 Hz, 1H), 2.76 (dd, *J* = 19.0, 7.8 Hz, 1H), 2.18 (s, 3H). ^13^C NMR (100 MHz, CDCl_3_) *δ* 208.4, 171.6, 143.4, 141.1, 130.8, 129.6, 129.1, 128.5, 127.5, 126.2, 121.0, 116.7, 76.4, 59.8, 53.5, 47.2, 35.0, 20.5. HRMS (ESI-TOF) *m/z*: [M + H]^+^ calcd for C_22_H_24_NO_3_^+^ 350.1750; found 350.1747.
Methyl (2S*,5R*)-2-allyl-3-oxo-5-phenyl-1-(4-(trifluoromethyl)phenyl)pyrrolidine-2-carboxylate **3w**. The title compound was prepared according to the general procedure using the corresponding diazo (0.0587 g, 0.15 mmol, 1.0 equiv), allyl carbonate (0.0290 g, 0.25 mmol 1.7 equiv), Rh_2_(OAc)_4_ (0.0015 g, 2.3 mol%), Pd_2_(dba)_3_ (0.0037 g, 2.7 mol%), and X-phos (0.0021 g, 3.0 mol%). Column chromatography (petroleum/ethyl acetate = 20:1) afforded 0.0502 g (83%, d.r. = 7:1) of **3w** as a colorless oil. Analytical data for **3w**: ^1^H NMR (400 MHz, CDCl_3_) *δ* 7.49–7.45 (m, 2H), 7.37–7.31 (m, 4H), 7.28–7.23 (m, 1H), 6.61 (d, *J* = 8.8 Hz, 2H), 5.42–5.30 (m, 1H), 5.09 (t, *J* = 8.0 Hz, 1H), 5.04 (dd, *J* = 10.2, 1.8 Hz, 1H), 4.91 (dd, *J* = 17.2, 1.6 Hz, 1H), 3.92 (s, 3H), 3.37 (dd, *J* = 14.4, 6.8 Hz, 1H), 3.19 (dd, *J* = 19.4, 8.6 Hz, 1H), 3.00 (dd, *J* = 14.2, 8.2 Hz, 1H), 2.80 (dd, *J* = 19.2, 7.6 Hz, 1H). ^13^C NMR (100 MHz, CDCl_3_) *δ* 207.2, 170.6, 146.4, 142.4, 130.1, 129.4, 128.8 (d, *J*_C–F_ = 57.5Hz), 127.9, 126.26 (q, *J*_C–F_ = 3.7Hz), 126.1, 121.7, 120.2 (q, *J*_C–F_ = 32.5Hz), 115.8, 76.3, 60.2, 53.8, 46.9, 34.9. HRMS (ESI-TOF) *m/z*: [M + H]^+^ calcd for C_22_H_21_F_3_NO_3_^+^ 404.1468; found 404.1461.
Methyl (2S*,5R*)-2-allyl-1-(4-chlorophenyl)-3-oxo-5-phenylpyrrolidine-2-carboxylate **3x**. The title compound was prepared according to the general procedure using the corresponding diazo (0.0536 g, 0.15 mmol, 1.0 equiv), allyl carbonate (0.0290 g, 0.25 mmol 1.7 equiv), Rh_2_(OAc)_4_ (0.0015 g, 2.3 mol%), Pd_2_(dba)_3_ (0.0037 g, 2.7 mol%), and X-phos (0.0021 g, 3.0 mol%). Column chromatography (petroleum/ethyl acetate = 20:1) afforded 0.0454 g (82%, d.r. = 19:1) of **3x** as a colorless oil. Analytical data for **3x**: ^1^H NMR (400 MHz, CDCl_3_) *δ* 7.46 (d, *J* = 7.2 Hz, 2H), 7.31 (t, *J* = 7.4 Hz, 2H), 7.24 (t, *J* = 7.2 Hz, 1H), 7.06 (d, *J* = 8.8 Hz, 2H), 6.50 (d, *J* = 9.2 Hz, 2H), 5.42–5.31 (m, 1H), 5.05–5.00 (m, 2H), 4.92 (d, *J* = 16.8 Hz, 1H), 3.91 (s, 3H), 3.30 (dd, *J* = 14.4, 6.8 Hz, 1H), 3.14 (dd, *J* = 19.2, 8.4 Hz, 1H), 2.96 (dd, *J* = 14.2, 8.2 Hz, 1H), 2.78 (dd, *J* = 19.2, 7.6 Hz, 1H). ^13^C NMR (100 MHz, CDCl_3_) *δ* 207.6, 171.1, 142.6, 142.2, 130.3, 129.3, 128.9, 127.8, 126.2, 124.3, 121.5, 117.8, 76.3, 60.0, 53.7, 47.1, 34.9. HRMS (ESI-TOF) *m/z*: [M + H]^+^ calcd for C_21_H_21_ClNO_3_^+^ 370.1204; found 370.1202.
Methyl (2S*,5R*)-2-allyl-3-oxo-5-phenyl-1-(m-tolyl)pyrrolidine-2-carboxylate **3y**. The title compound was prepared according to the general procedure using the corresponding diazo (0.0506 g, 0.15 mmol, 1.0 equiv), allyl carbonate (0.0290 g, 0.25 mmol 1.7 equiv), Rh_2_(OAc)_4_ (0.0015 g, 2.3 mol%), Pd_2_(dba)_3_ (0.0037 g, 2.7 mol%), and X-phos (0.0021 g, 3.0 mol%). Column chromatography (petroleum/ethyl acetate = 20:1) afforded 0.0409 g (78%, d.r. > 20:1) of **3y** as a colorless oil. Analytical data for **3y**: ^1^H NMR (400 MHz, CDCl_3_) *δ* 7.51–7.48 (m, 2H), 7.31 (t, *J* = 7.4 Hz, 2H), 7.22 (t, *J* = 7.2 Hz, 1H), 6.99 (t, *J* = 7.8 Hz, 1H), 6.57 (d, *J* = 7.6 Hz, 1H), 6.41–6.37 (m, 2H), 5.47–5.35 (m, 1H), 5.08 (t, *J* = 8.0 Hz, 1H), 5.03 (dd, *J* = 10.4, 2.0 Hz, 1H), 4.97–4.92 (m, 1H), 3.91 (s, 3H), 3.39 (dd, *J* = 14.2, 6.2 Hz, 1H), 3.13 (dd, *J* = 19.2, 8.4 Hz, 1H), 2.94 (dd, *J* = 14.2, 8.6 Hz, 1H), 2.77 (dd, *J* = 19.2, 7.6 Hz, 1H), 2.19 (s, 3H). ^13^C NMR (100 MHz, CDCl_3_) *δ* 208.3, 171.5, 143.6, 143.4, 138.5, 130.8, 129.1, 128.8, 127.5, 126.2, 121.0, 120.2, 117.4, 113.9, 76.4, 59.8, 53.5, 47.2, 35.0, 21.9. HRMS (ESI-TOF) *m/z*: [M + H]^+^ calcd for C_22_H_24_NO_3_^+^ 350.1751; found 350.1745.
Methyl (2S*,5R*)-2-allyl-1-(3-bromophenyl)-3-oxo-5-phenylpyrrolidine-2-carboxylate**3z**. The title compound was prepared according to the general procedure using the corresponding diazo (0.0602 g, 0.15 mmol, 1.0 equiv), allyl carbonate (0.0290 g, 0.25 mmol 1.7 equiv), Rh_2_(OAc)_4_ (0.0015 g, 2.3 mol%), Pd_2_(dba)_3_ (0.0037 g, 2.7 mol%), and X-phos (0.0021 g, 3.0 mol%). Column chromatography (petroleum/ethyl acetate = 20:1) afforded 0.0539 g (87%, d.r. = 8:1) of **3z** as a colorless oil. Analytical data for **3z**: ^1^H NMR (400 MHz, CDCl_3_) *δ* 7.47 (d, *J* = 7.6 Hz, 2H), 7.32 (t, *J* = 7.6 Hz, 2H), 7.26–7.22 (m, 1H), 6.96 (t, *J* = 8.0 Hz, 1H), 6.87 (d, *J* = 8.0 Hz, 1H), 6.71 (s, 1H), 6.49 (dd, *J* = 8.4, 2.0 Hz, 1H), 5.43–5.32 (m, 1H), 5.06–5.00 (m, 2H), 4.93 (d, *J* = 17.2 Hz, 1H), 3.91 (s, 3H), 3.34 (dd, *J* = 14.2, 6.6 Hz, 1H), 3.14 (dd, *J* = 19.2, 8.4 Hz, 1H), 2.97 (dd, *J* = 14.4, 8.4 Hz, 1H), 2.78 (dd, *J* = 19.2, 8.0 Hz, 1H). ^13^C NMR (100 MHz, CDCl_3_) *δ* 207.4, 170.8, 145.0, 142.5, 130.3, 130.2, 129.3, 127.8, 126.2, 123.0, 122.2, 121.5, 119.5, 115.2, 76.3, 60.0, 53.7, 47.0, 34.9. HRMS (ESI-TOF) *m/z*: [M + H]^+^ calcd for C_21_H_21_BrNO_3_^+^ 414.0699; found 414.0696.
Methyl (2S*,5R*)-2-allyl-1-(3-nitrophenyl)-3-oxo-5-phenylpyrrolidine-2-carboxylate **3aa**. The title compound was prepared according to the general procedure using the corresponding diazo (0.0552 g, 0.15 mmol, 1.0 equiv), allyl carbonate (0.0290 g, 0.25 mmol 1.7 equiv), Rh_2_(OAc)_4_ (0.0015 g, 2.3 mol%), Pd_2_(dba)_3_ (0.0037 g, 2.7 mol%), and X-phos (0.0021 g, 3.0 mol%). Column chromatography (petroleum/ethyl acetate = 20:1) afforded 0.0433 g (76%, d.r. > 20:1) of **3aa** as a colorless oil. Analytical data for **3aa**: ^1^H NMR (400 MHz, CDCl_3_) *δ* 7.59 (dd, *J* = 8.2, 1.4 Hz, 1H), 7.51 (d, *J* = 7.2 Hz, 2H), 7.45 (t, *J* = 2.4 Hz, 1H), 7.34 (t, *J* = 7.4 Hz, 2H), 7.28 (s, 1H), 7.24 (s, 1H), 6.86 (dd, *J* = 8.4, 2.4 Hz, 1H), 5.41–5.31 (m, 1H), 5.09 (t, *J* = 8.2 Hz, 1H), 5.06–5.02 (m, 1H), 4.88 (dd, *J* = 17.2, 1.2 Hz, 1H), 3.95 (s, 3H), 3.30 (dd, *J* = 14.2, 7.0 Hz, 1H), 3.20 (dd, *J* = 19.2, 8.4 Hz, 1H), 3.04 (dd, *J* = 14.4, 8.0 Hz, 1H), 2.85 (dd, *J* = 19.2, 8.0 Hz, 1H). ^13^C NMR (100 MHz, CDCl_3_) *δ* 206.8, 170.4, 149.0, 144.6, 141.7, 129.8, 129.62, 129.5, 128.2, 126.3, 122.0, 121.9, 113.8, 111.0, 76.2, 60.4, 53.9, 46.9, 34.9. HRMS (ESI-TOF) *m/z*: [M + H]^+^ calcd for C_21_H_21_N_2_O_5_^+^ 381.1445; found 381.1446.
Methyl (2S*,5R*)-2-allyl-1-(2-methoxyphenyl)-3-oxo-5-phenylpyrrolidine-2-carboxylate **3ab**. The title compound was prepared according to the general procedure using the corresponding diazo (0.0530 g, 0.15 mmol, 1.0 equiv), allyl carbonate (0.0290 g, 0.25 mmol 1.7 equiv), Rh_2_(OAc)_4_ (0.0015 g, 2.3 mol%), Pd_2_(dba)_3_ (0.0037 g, 2.7 mol%), and X-phos (0.0021 g, 3.0 mol%). Column chromatography (petroleum/ethyl acetate = 20:1) afforded 0.027 g (49%, d.r. = 1.5:1) of **3ab** as a colorless solid. Analytical data for **3ab**: m.p.: 103.6–105.2 °C. ^1^H NMR (400 MHz, CDCl_3_) *δ* 7.53 (d, *J* = 7.6 Hz, 2H), 7.30–7.27 (m, 2H), 7.18 (t, *J* = 7.4 Hz, 1H), 6.86–6.76 (m, 3H), 6.72–6.67 (m, 1H), 5.39 (dq, *J* = 16.8, 8.0 Hz, 1H), 5.12 (dd, *J* = 9.8, 7.4 Hz, 1H), 4.94 (d, *J* = 10.0 Hz, 1H), 4.81 (d, *J* = 17.2 Hz, 1H), 3.86 (s, 3H), 3.72 (s, 3H), 3.07 (dd, *J* = 14.2, 8.2 Hz, 1H), 2.97 (dd, *J* = 18.0, 7.2 Hz, 1H), 2.86 (dd, *J* = 18.0, 10.0 Hz, 1H), 2.53 (dd, *J* = 14.4, 6.4 Hz, 1H). ^13^C NMR (100 MHz, CDCl_3_) *δ* 208.7 171.4, 151.4, 142.7, 133.3, 131.7, 128.9, 127.5, 126.7, 122.0, 120.9, 120.5, 111.3, 77.0, 60.1, 54.7, 52.8, 46.4, 36.4. HRMS (ESI-TOF) *m/z*: [M + H]^+^ calcd for C_22_H_24_NO_4_^+^ 366.1700; found 366.1693.
Methyl (2S*,5R*)-2-allyl-3-oxo-5-phenyl-1-(o-tolyl)pyrrolidine-2-carboxylate **3ac**. The title compound was prepared according to the general procedure using the corresponding diazo (0.0506 g, 0.15 mmol, 1.0 equiv), allyl carbonate (0.0290 g, 0.25 mmol 1.7 equiv), Rh_2_(OAc)_4_ (0.0015 g, 2.3 mol%), Pd_2_(dba)_3_ (0.0037 g, 2.7 mol%), and X-phos (0.0021 g, 3.0 mol%). Column chromatography (petroleum/ethyl acetate = 20:1) afforded 0.0183 g (35%, d.r. = 1:1) of **3ac** as a colorless oil. Analytical data for **3ac**: ^1^H NMR (400 MHz, CDCl_3_) *δ* 7.36 (d, *J* = 6.8 Hz, 3H), 7.23–7.18 (m, 2H), 7.17–7.12 (m, 1H), 7.07–7.01 (m, 2H), 6.95 (t, *J* = 7.4 Hz, 1H), 5.76 (s, 1H), 5.12–4.99 (m, 3H), 3.80 (s, 3H), 2.98 (d, *J* = 8.0 Hz, 2H), 2.74–2.68 (m, 1H), 2.53–2.48 (m, 1H), 2.13 (s, 3H). ^13^C NMR (100 MHz, CDCl_3_) *δ* 207.7, 170.7, 140.7, 131.6, 131.4, 131.1, 129.1, 128.6, 127.8, 127.2, 126.8, 125.8, 120.6, 105.0, 75.9, 62.1, 52.9, 46.8, 35.7, 29.8. HRMS (ESI-TOF) *m/z*: [M + H]^+^ calcd for C_22_H_26_NO_3_^+^ 350.1750; found 350.1752.
Methyl (2S*,5R*)-2-allyl-1-(2-chlorophenyl)-3-oxo-5-phenylpyrrolidine-2-carboxylate**3ad**. The title compound was prepared according to the general procedure using the corresponding diazo (0.0536 g, 0.15 mmol, 1.0 equiv), allyl carbonate (0.0290 g, 0.25 mmol 1.7 equiv), Rh_2_(OAc)_4_ (0.0015 g, 2.3 mol%), Pd_2_(dba)_3_ (0.0037 g, 2.7 mol%), and X-phos (0.0021 g, 3.0 mol%). Column chromatography (petroleum/ethyl acetate = 20:1) afforded 0.0349 g (63%, d.r. = 2:1) of **3ad** as a colorless oil. Analytical data for **3ad**: ^1^H NMR (400 MHz, CDCl_3_) *δ* 7.46 (s, 2H), 7.25–7.22 (m, 4H), 7.17 (t, *J* = 7.2 Hz, 1H), 7.07 (td, *J* = 7.6, 1.4 Hz, 1H), 6.97–6.91 (m, 1H), 5.69–5.58 (m, 1H), 5.24–5.01 (m, 2H), 4.93 (dd, *J* = 16.8, 1.6 Hz, 1H), 3.82 (s, 3H), 2.98 (d, *J* = 8.0 Hz, 2H), 2.87 (s, 1H), 2.59 (s, 1H). ^13^C NMR (100 MHz, CDCl_3_) *δ* 209.0, 188.7, 170.7, 141.6, 132.1, 131.5, 128.7, 128.5, 127.8, 127.5, 127.1, 126.7, 125.7, 120.1, 75.6, 52.9, 52.7, 47.0, 35.4. HRMS (ESI-TOF) *m/z*: [M + H]^+^ calcd for C_21_H_21_ClNO_3_^+^ 370.1204; found 370.1207.
Methyl (2S*,5R*)-2-allyl-3-oxo-5-phenyl-1-(2-(trifluoromethyl)phenyl)pyrrolidine-2-carboxylate**3ae**. The title compound was prepared according to the general procedure using the corresponding diazo (0.0587 g, 0.15 mmol, 1.0 equiv), allyl carbonate (0.0290 g, 0.25 mmol 1.7 equiv), Rh_2_(OAc)_4_ (0.0015 g, 2.3 mol%), Pd_2_(dba)_3_ (0.0037 g, 2.7 mol%), and X-phos (0.0021 g, 3.0 mol%). Column chromatography (petroleum/ethyl acetate = 20:1) afforded 0.0435 g (72%, d.r. > 20:1) of **3ae** as a colorless oil. Analytical data for **3ae**: ^1^H NMR (400 MHz, CDCl_3_) *δ* 7.52–7.44 (m, 3H), 7.25–7.16 (m, 6H), 6.13–6.02 (m, 1H), 5.17–5.10 (m, 2H), 5.09–5.06 (m, 1H), 3.66 (s, 3H), 3.08 (d, *J* = 17.2 Hz, 1H), 2.81–2.71 (m, 2H), 2.63–2.56 (m, 1H). ^13^C NMR (100 MHz, CDCl_3_) *δ* 208.8, 169.3, 142.3, 137.8, 132.2, 132.2, 132.1, 130.6 (q, *J*_C–F_ = 24.8 Hz), 128.8, 128.5 (q, *J*_C–F_ = 5.1 Hz), 128.3, 127.5, 123.5 (d, *J*_C–F_ = 274.6 Hz), 119.5, 114.7, 78.3, 64.7, 52.0, 45.7, 37.5. HRMS (ESI-TOF) *m/z*: [M + H]^+^ calcd for C_22_H_21_F_3_NO_3_^+^ 404.1468; found 404.1470.
Methyl (2S*,5R*)-2-allyl-3-oxo-5-phenyl-1-(3,4,5-trichlorophenyl)pyrrolidine-2-carboxylate **3af**. The title compound was prepared according to the general procedure using the corresponding diazo (0.0638 g, 0.15 mmol, 1.0 equiv), allyl carbonate (0.0290 g, 0.25 mmol 1.7 equiv), Rh_2_(OAc)_4_ (0.0015 g, 2.3 mol%), Pd_2_(dba)_3_ (0.0037 g, 2.7 mol%), and X-phos (0.0021 g, 3.0 mol%). Column chromatography (petroleum/ethyl acetate = 20:1) afforded 0.0531 g (81%, d.r. > 20:1) of **3af** as a colorless oil. Analytical data for **3af**: ^1^H NMR (400 MHz, CDCl_3_) *δ* 7.47–7.44 (m, 2H), 7.37–7.32 (m, 2H), 7.30–7.26 (m, 1H), 6.59 (s, 2H), 5.42–5.32 (m, 1H), 5.08 (dd, *J* = 10.2, 1.8 Hz, 1H), 5.00–4.94 (m, 2H), 3.93 (s, 3H), 3.29 (dd, *J* = 14.4, 6.8 Hz, 1H), 3.15 (dd, *J* = 19.2, 8.4 Hz, 1H), 3.00 (dd, *J* = 14.4, 8.0 Hz, 1H), 2.80 (dd, *J* = 19.4, 7.8 Hz, 1H). ^13^C NMR (100 MHz, CDCl_3_) *δ* 206.5, 170.2, 142.9, 141.6, 134.2, 129.8, 129.5, 128.2 126.1, 122.0, 121.3, 116.7, 76.1, 60.3, 53.9, 46.8, HRMS (ESI-TOF) *m/z*: [M + H]^+^ calcd for C_21_H_19_Cl_3_NO_3_^+^ 438.0425; found 438.0420.
(2S*,5R*)-2-allyl-5-(4-methoxyphenyl)-2-methyl-1-(4-nitrophenyl)pyrrolidin-3-one **3ag.** The title compound was prepared according to the general procedure using the corresponding diazo (0.0597 g, 0.15 mmol, 1.0 equiv), allyl carbonate (0.0290 g, 0.25 mmol 1.7 equiv), Rh_2_(OAc)_4_ (0.0015 g, 2.3 mol%), Pd_2_(dba)_3_ (0.0037 g, 2.7 mol%), and X-phos (0.0021 g, 3.0 mol%). Column chromatography (petroleum/ethyl acetate = 20:1) afforded 0.0324 g (59%, d.r. = 4:1) of **3ag** as a colorless oil. Analytical data for **3ag**: ^1^H NMR (400 MHz, CDCl_3_) *δ* 8.00 (d, *J* = 9.2 Hz, 2H), 7.37 (d, *J* = 8.8 Hz, 2H), 6.86 (d, *J* = 8.8 Hz, 2H), 6.57 (d, *J* = 9.2 Hz, 2H), 5.40–5.28 (m, 1H), 5.11–5.02 (m, 2H), 4.89 (dd, *J* = 16.8, 1.6 Hz, 1H), 3.92 (s, 3H), 3.77 (s, 3H), 3.34 (dd, *J* = 14.4, 7.2 Hz, 1H), 3.20 (dd, *J* = 19.6, 8.8 Hz, 1H), 3.05 (dd, *J* = 14.2, 7.8 Hz, 1H), 2.82 (dd, *J* = 19.6, 7.2 Hz, 1H). ^13^C NMR (100 MHz, CDCl_3_) *δ* 206.4, 169.8, 159.3, 149.2, 139.4, 133.5, 129.6, 127.2, 125.4, 122.0, 115.3, 114.9, 76.4, 60.1, 55.4, 53.9, 46.8, 35.1. HRMS (ESI-TOF) *m/z*: [M + H]^+^ calcd for C_21_H_23_N_2_O_4_^+^ 411.1551; found 411.1544.
Propyl (2S*,5R*)-2-allyl-3-oxo-1,5-diphenylpyrrolidine-2-carboxylate **3ah**. The title compound was prepared according to the general procedure using the corresponding diazo (0.0549 g, 0.15 mmol, 1.0 equiv), allyl carbonate (0.0290 g, 0.25 mmol 1.7 equiv), Rh_2_(OAc)_4_ (0.0015 g, 2.3 mol%), Pd_2_(dba)_3_ (0.0037 g, 2.7 mol%), and X-phos (0.0021 g, 3.0 mol%). Column chromatography (petroleum/ethyl acetate = 20:1) afforded 0.0332 g (61%, d.r. > 20:1) of **3ah** as a colorless oil. Analytical data for **3ah**: ^1^H NMR (400 MHz, CDCl_3_) *δ* 7.53–7.50 (m, 2H), 7.32–7.27 (m, 2H), 7.24–7.19 (m, 1H), 7.11 (dd, *J* = 8.8, 7.2 Hz, 2H), 6.74 (t, *J* = 7.4 Hz, 1H), 6.60 (d, *J* = 7.6 Hz, 2H), 5.45–5.34 (m, 1H), 5.08 (t, *J* = 8.2 Hz, 1H), 5.01 (dd, *J* = 10.0, 2.0 Hz, 1H), 4.94–4.88 (m, 1H), 4.32–4.21 (m, 2H), 3.37 (dd, *J* = 14.0, 6.4 Hz, 1H), 3.14 (dd, *J* = 19.2, 8.4 Hz, 1H), 2.94 (dd, *J* = 14.2, 8.6 Hz, 1H), 2.77 (dd, *J* = 19.2, 7.6 Hz, 1H), 1.75 (m, 2H), 0.96 (t, *J* = 7.4 Hz, 3H). ^13^C NMR (100 MHz, CDCl_3_) *δ* 208.3, 171.0, 143.6, 143.3, 130.8, 129.1, 128.9, 127.5, 126.2, 121.1, 119.1, 116.7, 76.5, 68.3, 59.7, 47.2, 34.9, 22.1, 10.6. HRMS (ESI-TOF) *m/z*: [M + H]^+^ calcd for C_23_H_26_NO_3_^+^ 364.1907; found 364.1900.
Benzyl (2S*,5R*)-2-allyl-3-oxo-1,5-diphenylpyrrolidine-2-carboxylate **3ai**. The title compound was prepared according to the general procedure using the corresponding diazo (0.0599 g, 0.15 mmol, 1.0 equiv), allyl carbonate (0.0290 g, 0.25 mmol 1.7 equiv), Rh_2_(OAc)_4_ (0.0015 g, 2.3 mol%), Pd_2_(dba)_3_ (0.0037 g, 2.7 mol%), and X-phos (0.0021 g, 3.0 mol%). Column chromatography (petroleum/ethyl acetate = 20:1) afforded 0.0500 g (81%, d.r. > 20:1) of **3ai** as a colorless oil. Analytical data for **3ai**: ^1^H NMR (400 MHz, CDCl_3_) *δ* 7.42–7.364 (m, 7H), 7.16–7.14 (m, 3H), 7.08 (dd, *J* = 8.8, 7.6 Hz, 2H), 6.74 (t, *J* = 7.4 Hz, 1H), 6.56 (d, *J* = 8.0 Hz, 2H), 5.46–5.35 (m, 2H), 5.29 (d, *J* = 12.0 Hz, 1H), 5.06 (t, *J* = 8.2 Hz, 1H), 5.04–5.01 (m, 1H), 4.92 (dd, *J* = 17.0, 1.4 Hz, 1H), 3.39 (dd, *J* = 14.0, 6.4 Hz, 1H), 3.13 (dd, *J* = 19.2, 8.4 Hz, 1H), 2.99 (dd, *J* = 14.4, 8.4 Hz, 1H), 2.74 (dd, *J* = 19.2, 8.0 Hz, 1H). ^13^C NMR (100 MHz, CDCl_3_) *δ* 208.1, 170.7, 143.5, 143.1, 135.0, 130.7, 129.0, 128.9, 128.8, 128.8, 128.7, 127.4, 126.3, 121.1, 119.2, 116.7, 76.5, 68.5, 59.7, 47.2, 35.0. HRMS (ESI-TOF) *m/z*: [M + H]^+^ calcd for C_27_H_26_NO_3_^+^ 412.1907; found 412.1899.
Tert-butyl (2S*,5R*)-2-allyl-3-oxo-1,5-diphenylpyrrolidine-2-carboxylate **3aj**. The title compound was prepared according to the general procedure using the corresponding diazo (0.0548 g, 0.15 mmol, 1.0 equiv), allyl carbonate (0.0290 g, 0.25 mmol 1.7 equiv), Rh_2_(OAc)_4_ (0.0015 g, 2.3 mol%), Pd_2_(dba)_3_ (0.0037 g, 2.7 mol%), and X-phos (0.0021 g, 3.0 mol%). Column chromatography (petroleum/ethyl acetate = 20:1) afforded 0.0492 g (87%, d.r. > 20:1) of **3aj** as a colorless oil. Analytical data for **3aj**: ^1^H NMR (400 MHz, CDCl_3_) *δ* 7.50–7.46 (m, 2H), 7.29–7.23 (m, 2H), 7.18 (t, *J* = 7.4 Hz, 1H), 7.08 (dd, *J* = 8.8, 7.2 Hz, 2H), 6.71 (t, *J* = 7.2 Hz, 1H), 6.59 (d, *J* = 8.0 Hz, 2H), 5.41–5.30 (m, 1H), 5.03 (t, *J* = 8.2 Hz, 1H), 4.96 (dd, *J* = 10.0, 2.0 Hz, 1H), 4.90–4.84 (m, 1H), 3.31 (dd, *J* = 14.2, 6.6 Hz, 1H), 3.08 (dd, *J* = 19.2, 8.4 Hz, 1H), 2.84 (dd, *J* = 14.0, 8.4 Hz, 1H), 2.71 (dd, *J* = 19.2, 8.0 Hz, 1H), 1.53 (s, 9H). ^13^C NMR (100 MHz, CDCl_3_) *δ* 208.9, 167.0, 143.8, 143.5, 131.0, 129.0, 128.9, 127.5, 126.2, 120.8, 118.9, 116.6, 83.4, 76.8, 59.7, 47.3, 34.8, 28.0. HRMS (ESI-TOF) *m/z*: [M + H]^+^ calcd for C_24_H_28_NO_3_ 378.2063; found 378.2055.
*Methyl 2-((2-methoxyphenyl)amino)-3-oxo-5-phenyl-2-propylpentanoate* **5**. To an oven-dried double-necked flask equipped with a magnetic stir bar were added **3ab** (54.6 mg, 1.0 mmol,), and Pd/C (31.9 mg, 0.3 mmol). The flask was evacuated and refilled with N_2_ for three times. To this flask were added anhydrous EtOH (20.0 mL). The flask was evacuated and refilled with H_2_ for three times. The reaction mixture was stirred under H_2_ atmosphere (hydrogen balloon) at room temperature for 8 h. Upon completion (monitored by TLC), the reaction mixture was filtrated through a pad of celite and washed with EtOAc. The filtrate was removed in vacuo to give the crude product, which was then purified by silica gel column chromatography using ethyl acetate/petroleum ether system (eluent: petroleum ether/ethyl acetate = 10/1) afforded (51.8 mg, 94% yield,) of **5** as a colorless oil. Analytical data for **5**: ^1^H NMR (400 MHz, CDCl_3_) *δ* 7.26–7.22 (m, 2H), 7.19–7.13 (m, 1H), 7.13–7.11 (m, 2H), 6.80 (dd, *J* = 7.6, 2.0 Hz, 1H), 6.75–6.66 (m, 2H), 6.27 (dd, *J* = 7.6, 1.6 Hz, 1H), 5.90 (s, 1H), 3.89 (s, 3H), 3.57 (s, 3H), 2.96–2.89 (m, 1H), 2.84–2.78 (m, 3H), 2.23 (t, *J* = 8.4 Hz, 2H), 1.06–0.92 (m, 2H), 0.79 (t, *J* = 7.4 Hz, 3H). ^13^C NMR (100 MHz, CDCl_3_) *δ* 205.4, 170.9, 147.4, 140.9, 133.8, 128.6, 128.5, 126.2, 121.0, 117.6, 111.0, 110.0, 73.2, 55.7, 53.3, 39.3, 31.7, 29.8, 16.6, 14.0. HRMS (ESI-TOF) *m/z*: [M + H]^+^ calcd for C_22_H_28_NO_4_^+^ 370.2013; found 370.2007.
Methyl(2S*,5R*)-2-allyl-3-hydroxy-1-(2-methoxyphenyl)-3-methyl-5-phenylpyrrolidine-2-carboxylate **6.** To an oven-dried Schlenk tube equipped with a magnetic stir bar, compound **3ab** (54.6 mg, 0.15 mmol) were added. The tube was evacuated and backfilled with N_2_ for 3 times and then charged with dry THF (1.0 mL) via syringe. To above stirred solution was added MeMgBr (0.3 mL of 3.0 M solution in THF, 6.0 equiv.) at 0 °C, and then the reaction was stirred at room temperature for 12 h. Upon completion (monitored by TLC), the reaction was quenched with sat. aq NaHCO_3_ and extracted with EtOAc. The organic layer was dried over Na_2_SO_4_ and concentrated in vacuo. The dr ratio was determined by ^1^H NMR of the crude reaction mixture. The residue was then purified by preparative thin layer chromatography (eluent: petroleum ether/ethyl acetate = 10/1) afforded (32.0 mg, 56% yield, >20: 1 d.r.) of **6** as a colorless oil. Analytical data for **6**: ^1^H NMR (400 MHz, CDCl_3_) *δ* 7.41 (dd, *J* = 7.8, 1.8 Hz, 1H), 7.31–7.28 (m, 2H), 7.15 (t, *J* = 7.4 Hz, 2H), 7.10–7.06 (m, 1H), 7.04–7.01 (m, 1H), 6.79 (td, *J* = 7.6, 1.2 Hz, 1H), 6.69 (dd, *J* = 8.2, 1.4 Hz, 1H), 5.51–5.40 (m, 1H), 5.01–4.95 (m, 2H), 4.86–4.82 (m, 1H), 3.71 (s, 3H), 3.68 (s, 3H), 2.85 (dd, *J* = 14.6, 8.2 Hz, 1H), 2.74 (dd, *J* = 13.8, 10.2 Hz, 1H), 2.66 (dd, *J* = 14.6, 6.0 Hz, 1H), 2.07 (dd, *J* = 13.8, 4.2 Hz, 1H), 1.41 (s, 3H). ^13^C NMR (100 MHz, CDCl_3_) *δ* 172.6, 157.9, 143.6, 134.4, 132.6, 130.2, 128.1, 127.6, 127.4, 126.8, 120.6, 117.6, 111.4, 80.5, 80.1, 63.6, 55.3, 51.2, 48.4, 36.0, 22.0. HRMS (ESI-TOF) *m/z*: [M + H]^+^ calcd for C_23_H_28_NO_4_^+^ 382.2013; found 382.2006.

## 4. Conclusions

In summary, we have developed a convenient and efficient protocol for carbenoid N-H insertion/allylation cascade reactions using the commercially available rhodium catalyst Rh_2_(OAc)_4_ and palladium catalyst Pd_2_(dba)_3_. This method delivers valuable pyrrolidines with broad substrate scope, high chemoselectivities, and excellent diastereoselectivity under mild reaction conditions. Moreover, scale-up of the optimal procedure and synthetic transformation of the products and its derivatives demonstrated the utility of the protocol.

## Figures and Tables

**Table 1:** Optimization of the reaction conditions [a].

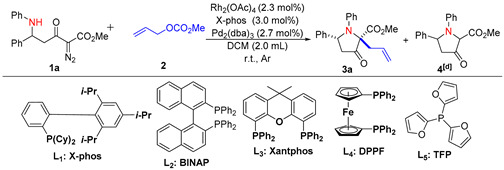					
Entry	L	[Rh]	[Pd]	d.r. ^[*b*]^	Yield ^[*c*]^
1	L_1_	Rh_2_(OAc)_4_	Pd(dba)_2_	>20:1	87%
2	L_2_	Rh_2_(OAc)_4_	Pd(dba)_2_	>20:1	82%
3	L_3_	Rh_2_(OAc)_4_	Pd(dba)_2_	>20:1	76%
4	L_4_	Rh_2_(OAc)_4_	Pd(dba)_2_	>20:1	80%
5	L_5_	Rh_2_(OAc)_4_	Pd(dba)_2_	>20:1	67%
**6**	**L_1_**	**Rh_2_(OAc)_4_**	**Pd_2_(dba)_3_**	**>20:1**	**91**%

[a] Reaction conditions: **1a** (0.15 mmol), **2** (0.25 mmol), Rh_2_(OAc)_4_ (2.3 mol%), Pd-complex (2.7 mol%), ligand (3.0 mol%), Rh_2_(OAc)_4_, Pd-complex, ligand, and **2** in solvent (1 mL) were stirred for 15 min at rt. **1a** dissolved in solvent (1 mL) were slowly added dropwise; [b] Diastereoselectivities were determined by ^1^H NMR spectroscopy of the crude reaction mixture; [c] Isolated yield; [d] The reaction produced byproduct **4**, unless otherwise note.

## Data Availability

Data are contained within the article and Supplementary Materials.

## Supplementary Materials

The following supporting information can be downloaded at: https://www.mdpi.com/article/10.3390/molecules29245880/s1, the charts of ^1^H-NMR, ^13^C-NMR, and HRMS of products. Ref. [53] is cited in Supplementary Materials.
