# Supplementary material for: Rh(II)/Pd(0) Dual Catalysis: Carbenoid N-H Insertion/Allylation Cascade Reaction to Construct Highly Functionalized and Polysubstituted Pyrrolidines
Source: Molecules. 2024 Dec 13;29(24):5880. doi: 10.3390/molecules29245880 (PMC11676634; doi:10.3390/molecules29245880)
Supplement: Supplementary file 1 [file molecules-29-05880-s001.zip › molecules-3354255-supplementary.pdf]

Supporting Information

Rh(II)/Pd(0) Dual Catalysis: Carbenoid N-H Insertion/Allylation Cascade Reaction to Construct Highly Functionalized and Polysubstituted Pyrrolidines

Maocheng Tang, [†] Xianyan Jiao, [†] Deping He, [†] Ji-Xing Zhao, [‡] Ping Liu*,[†] Chun-Tian Li*[†]

[†]State Key Laboratory Incubation Base for Green Processing of Chemical Engineering, School of Chemistry and Chemical Engneering, Shihezi University, Xinjiang Uygur Autonomous Region 832000, People's Republic of China

[‡] Analysis and Testing Center, Shihezi University, Xinjiang 832003, P. R. China

Table of Contents

Table S1 Chiral reaction conditions optimized....2
Table S2 Chiral reaction conditions optimized.[a] 2
General procedure for the synthesis of compound **5**....3
General procedure for synthesis of compound **6**....4
Gram-level reaction procedure....5
NMR spectrum of substrates and products....6
Figure S1. X-Ray crystal structure of the compound **3ab**....77
Table S3. Crystal data and structure refinement for compound **3ab** 78
References....79

Table S1 Chiral reaction conditions optimized.

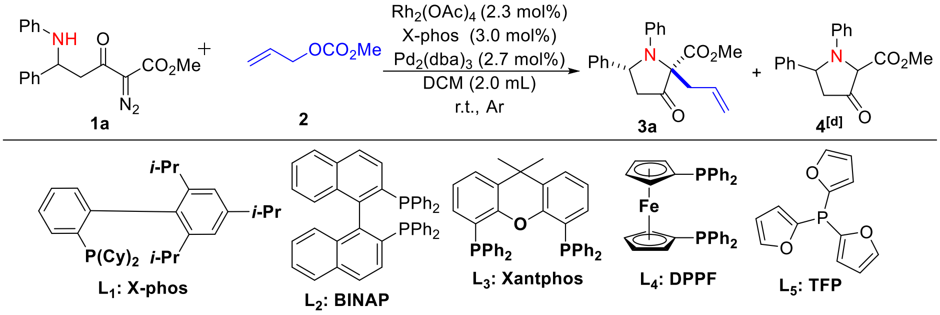

Entry	L	[Rh]	[Pd]	d.r.[b]	Yeild[c]
1	L_1	$Rh_2(OCt)_4$	$Pd(dba)_2$	-	69%
2	L_1	$[Rh_2(TPA)_4]$	$Pd(dba)_2$	-	63%
3	L_1	$[[Rh(COD)Cl]_2]$	$Pd(dba)_2$	-	41%
4	L_1	$[(CF_3COO)_2Rh]_2$	$Pd(dba)_2$	-	trace
5	L_1	$[[(CH_3)_3CCO_2]_2Rh]_2$	$Pd(dba)_2$	-	58%
**6**	**L_1**	**$Rh_2(OAc)_4$**	**$Pd_2(dba)_3$**	**> 20 : 1**	**91%**
7	L_1	$Rh_2(OAc)_4$	$Pd(PPh_3)_4$	-	81%
8	L_1	$Rh_2(OAc)_4$	$Pd(allyl)Cl_2$	-	15%
9	L_1	$Rh_2(OAc)_4$	$DPPFPdCl_2$	-	81%

[*a*] Reaction conditions: **1a** (0.15 mmol), **2** (0.25 mmol), $Rh_2(OAc)_4$ (2.3 mol%), Pd-complex (2.7 mol%), ligand (3.0 mol%), $Rh_2(OAc)_4$, Pd-complex, ligand and **2** in solvent (1 mL) were stirred for 15 min at rt. **1a** dissolved in solvent (1 mL) were slowly added dropwise; [*b*] Diastereoselectivities were determined by ^1H NMR spectroscopy of the crude reaction mixture; [*c*] Isolated yield; [*d*] The reaction produced byproduct **4**, unless otherwise noted.

Table S2 Chiral reaction conditions optimized.[a]

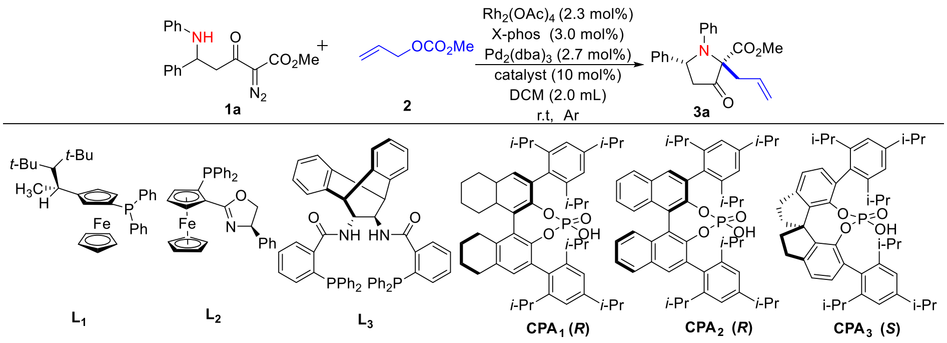

Entry	Solvent	L/x	Cat.	Rh	Pd	er[c]	Yeild[b]	d.r.[d]

							（%）	
1	DCM	L_1		$Rh_2(TPA)_4$	$Pd(dba)_2$	39:61	32	1:5
2	$CDCl_3$	L_1		$Rh_2(OAc)_4$	$Pd(dba)_2$	64:36	27	1:2
3	DCM	L_1		$Rh_2(Oct)_4$	$Pd(dba)_2$	63:37	23	1:3
4	DCM	L_2		$Rh_2(OAc)_4$	$Pd(PPh_3)_4$	63:37	12	1:3
5	DCM	L_2		$Rh_2(OAc)_4$	$Pd(dba)_2$	60:40	41	1:18
6	$CDCl_3$	L_2		$Rh_2(Oct)_4$	$Pd(dba)_2$	61:39	16	1:4
7	DCM	L_2		$Rh_2(Oct)_4$	$Pd(allyl)Cl_2$	63:37	12	1:2
8	DCM	L_3		$Rh_2(OAc)_4$	$Pd(dba)_2$	55:45	39	1:15
9[e]	DCM	X-phos	CPA_1	$Rh_2(OAc)_4$	$Pd(dba)_2$		NR	
10	DCM	X-phos	CPA_2	$Rh_2(OAc)_4$	$Pd(dba)_2$		NR	
11	DCM	X-phos	CPA_3	$Rh_2(OAc)_4$	$Pd(dba)_2$	50:50	10	＞ 20:1

[a] Reaction conditions: **1a** (0.15 mmol), **2** (0.25 mmol), $Rh_2(OAc)_4$ (2.3 mol%), Pd-complex (2.7 mol%), ligand (3.0 mol%), $Rh_2(OAc)_4$, Pd-complex, ligand and **2** in solvent (1 mL) were stirred for 15 min at rt. **1a** dissolved in solvent (1 mL) were slowly added dropwise; [b] Yield was determined by ^1H NMR spectroscopy of the crude reaction mixture using 1,3,5-triacetylbenzene as the internal standard; [c] Determined by chiral HPLC analysis; [d] Diastereoselectivities were determined by ^1H NMR spectroscopy of the crude reaction mixture; [e] Chiral phosphoric acid catalyst is pre-stirred with metal catalyst for 15 minutes;

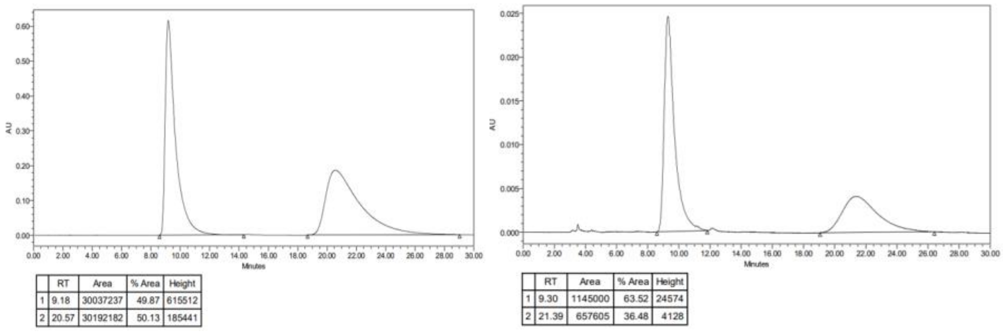

Entry 2：Daicel Chiralcel OJ-H column; n-hexane/2-propanol = 99:1 (v/v); temp: 30 °C; flow rate: 1.0 mL/min; uv-vis detection: λ = 254 nm; t_R (major) = 9.18 min; t_R (minor) = 20.57 min.

General procedure for the synthesis of compound 5

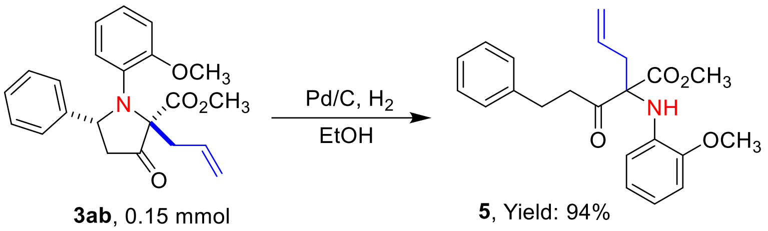

To an oven-dried double-necked flask equipped with a magnetic stir bar were added **3ab** (54.6 mg, 1.0 mmol,), and Pd/C (31.9 mg, 0.3 mmol). The flask was evacuated and refilled with N_2 for 3 times. To this flask were added anhydrous EtOH (20.0 mL). The flask was evacuated and refilled with H_2 for three times. The reaction mixture was stirred under H_2 atmosphere (hydrogen balloon) at room temperature for 8 h. Upon completion (monitored by TLC), the reaction mixture was filtrated through a pad of celite and washed with EtOAc. The filtrate were removed in vacuo to give the crude product, which was then purified by silica gel column chromatography using ethyl acetate/petroleum ether system (eluent: petroleum ether/ethyl acetate = 10/1) to afford 51.8 mg (94%) of **5** as a colorless oil.[1]

General procedure for synthesis of compound 6

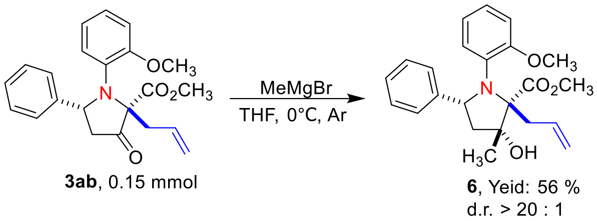

To an oven-dried Schlenk tube equipped with a magnetic stir bar, compound **3ab** (54.6 mg, 0.15 mmol) were added. The tube was evacuated and backfilled with N_2 for 3 times and then charged with dry THF (1.0 mL) via syringe. To above stirred solution was added MeMgBr (0.3 mL of 3.0 M solution in THF, 6.0 equiv.) at 0 °C, and then the reaction was stirred at room temperature for 12 h. Upon completion (monitored by TLC), the reaction was quenched with sat. aq $NaHCO_3$ (5 mL), and extracted with EtOAc (3 × 5 mL). The organic layer was dried over Na_2SO_4 and concentrated in vacuo. The dr ratio was determined by ^{1}H NMR of the crude reaction mixture. The residue was then purified by preparative thin layer chromatography (eluent: petroleum ether/ethyl acetate = 10/1) to give the product **6** as a colorless oil (32.0 mg, 56% yield, >20: 1 d.r.). [1]

Gram-level reaction procedure

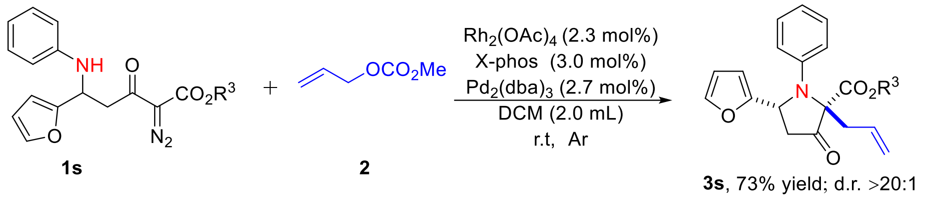

Allyl methyl carbonate **2** (0.9870 g, 8.5 mmol, 1.7 equiv.), $Rh_2(OAc)_4$ (0.0508 g, 2.3 mol%) X-phos (0.1374 g, 3.0 mol %), $Pd_2(dba)_3$ (0.0644 g, 2.7 mol%) were loaded into a drying tube. After three times of ventilation with argon, 40 mL of CH_2Cl_2 solution was added and stirred at room temperature for 15 min. The diazo **1s** (1.5656 g, 5.0 mmol, 1.0 equiv.) was dissolved in 20 mL of CH_2Cl_2 and slowly added to the reaction solution. The resulting mixture was maintained at room temperature until the absence of starting material via TLC analysis (about 16 h) and quenched with saturated NaCl (20 mL). The aqueous layer was extracted with EtOAc (3 × 15 mL). The combined organic layers were dried over Na_2SO_4 and concentrated in vacuo. The residue was purified by chromatography (eluent: petroleum ether/ethyl acetate = 20/1) to afford product **3s**.

NMR spectrum of substrates and products

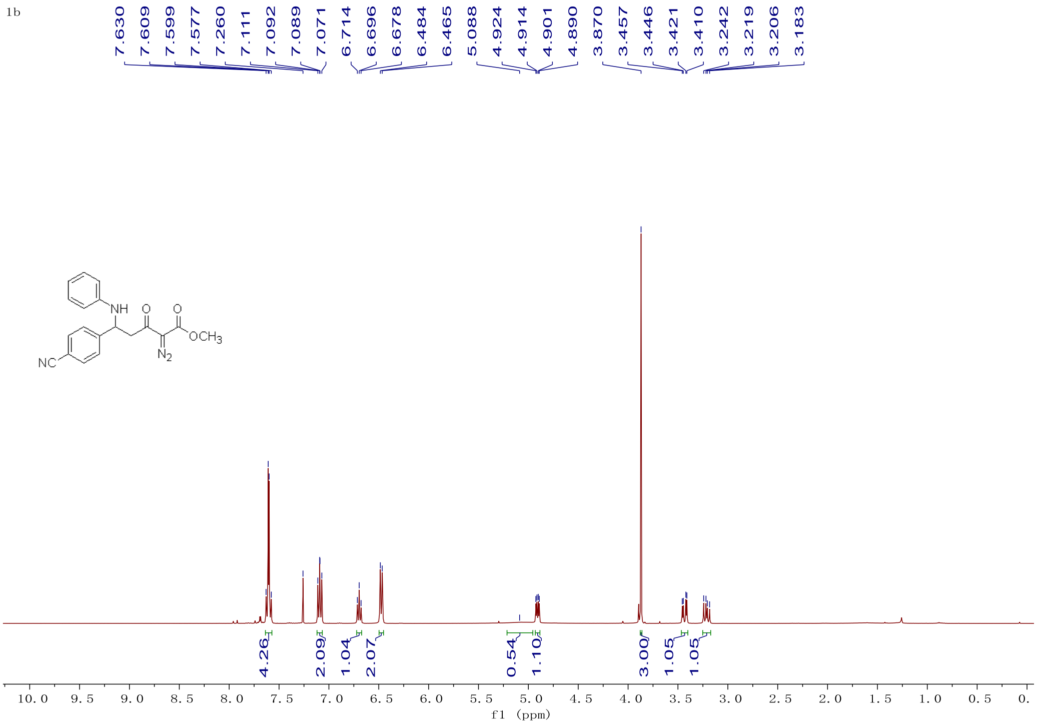

^{1}H NMR spectrum ($CDCl_3$, 400 MHz) of **1b**

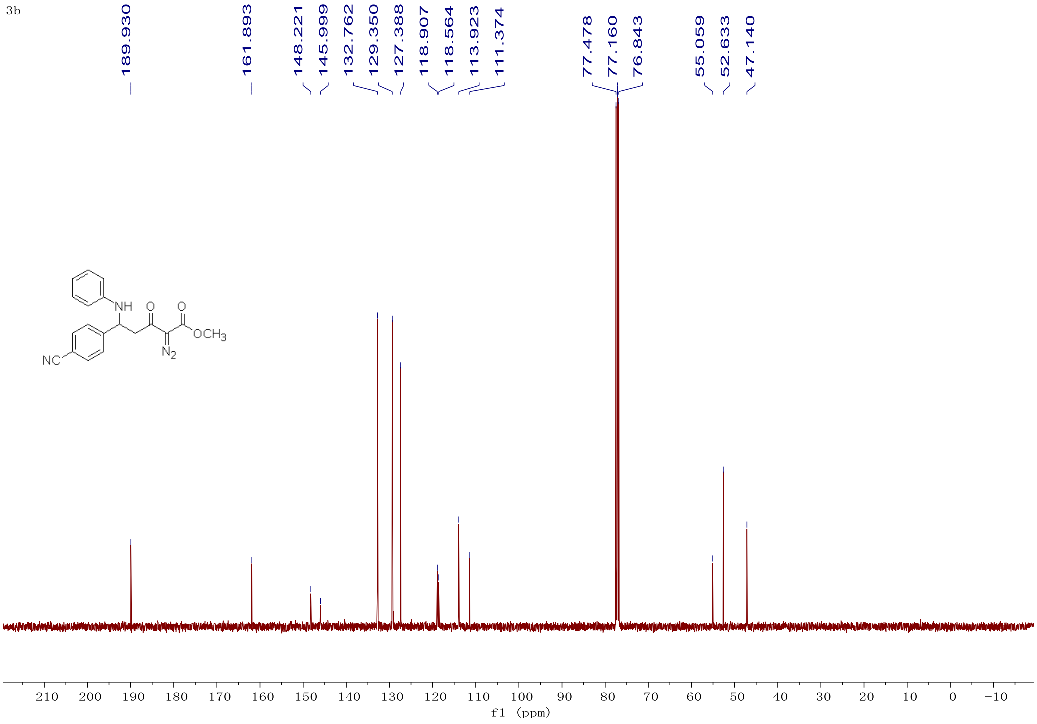

^{13}C NMR spectrum ($CDCl_3$, 100 MHz) of **1b**

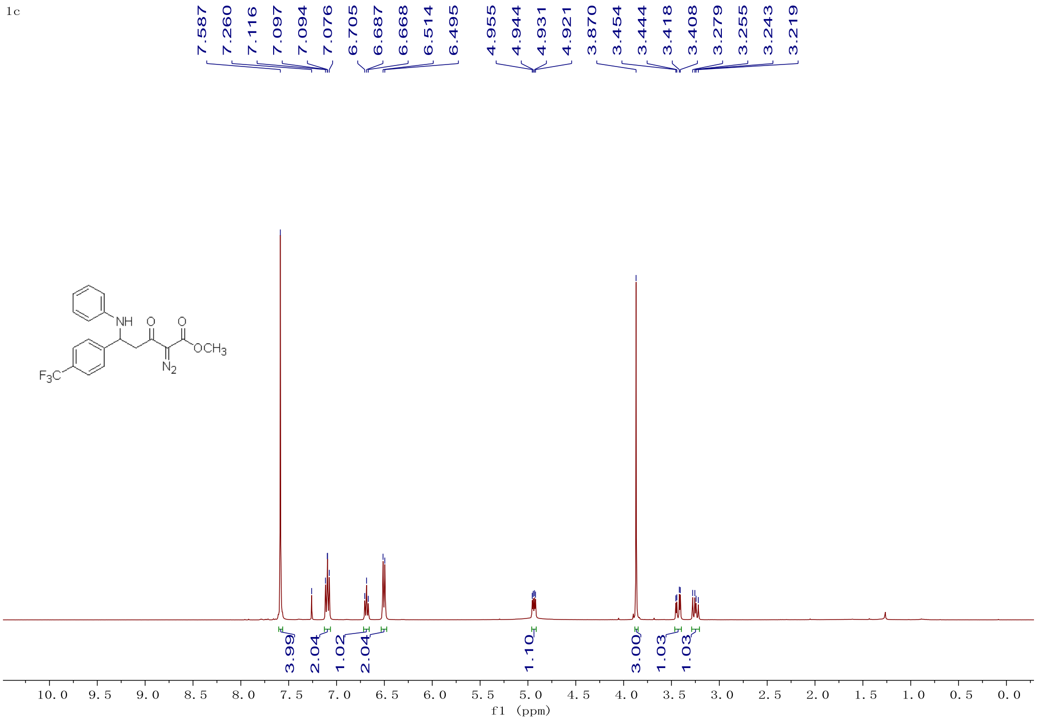

^{1}H NMR spectrum ($CDCl_3$, 400 MHz) of **1c**

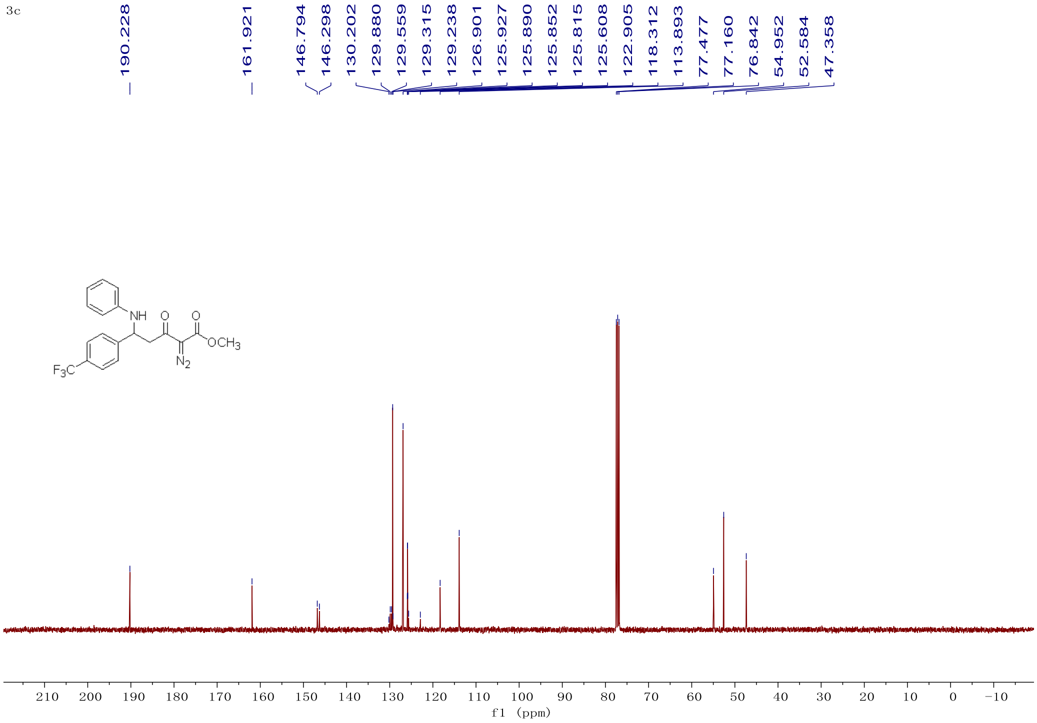

^{13}C NMR spectrum ($CDCl_3$, 100 MHz) of **1c**

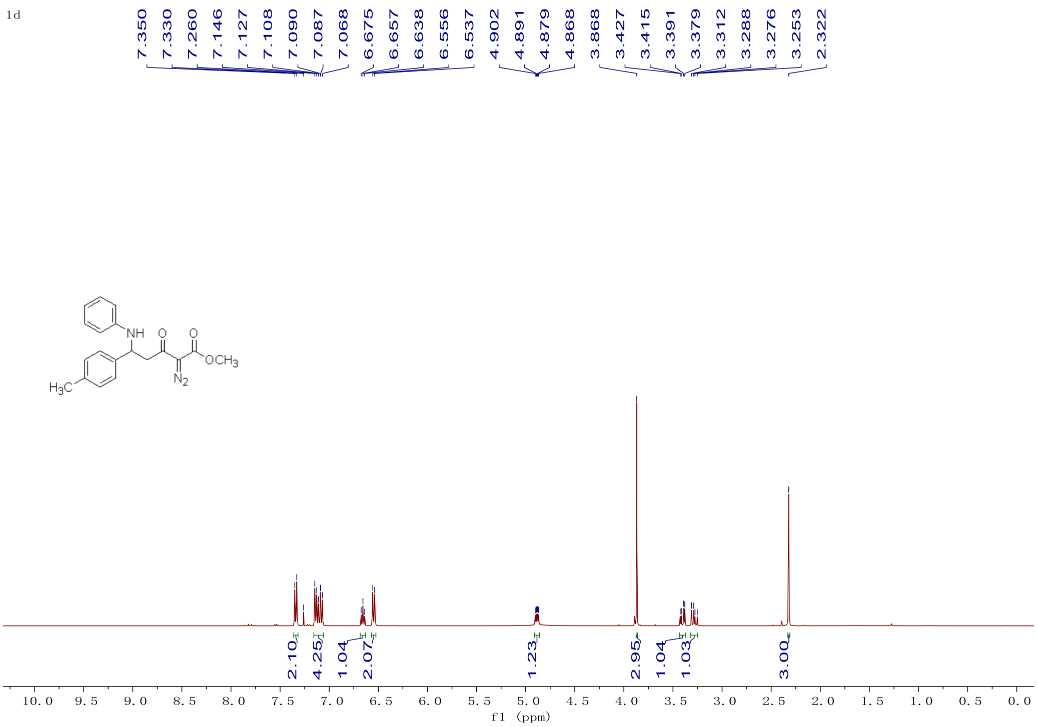

^{1}H NMR spectrum ($CDCl_3$, 400 MHz) of **1d**

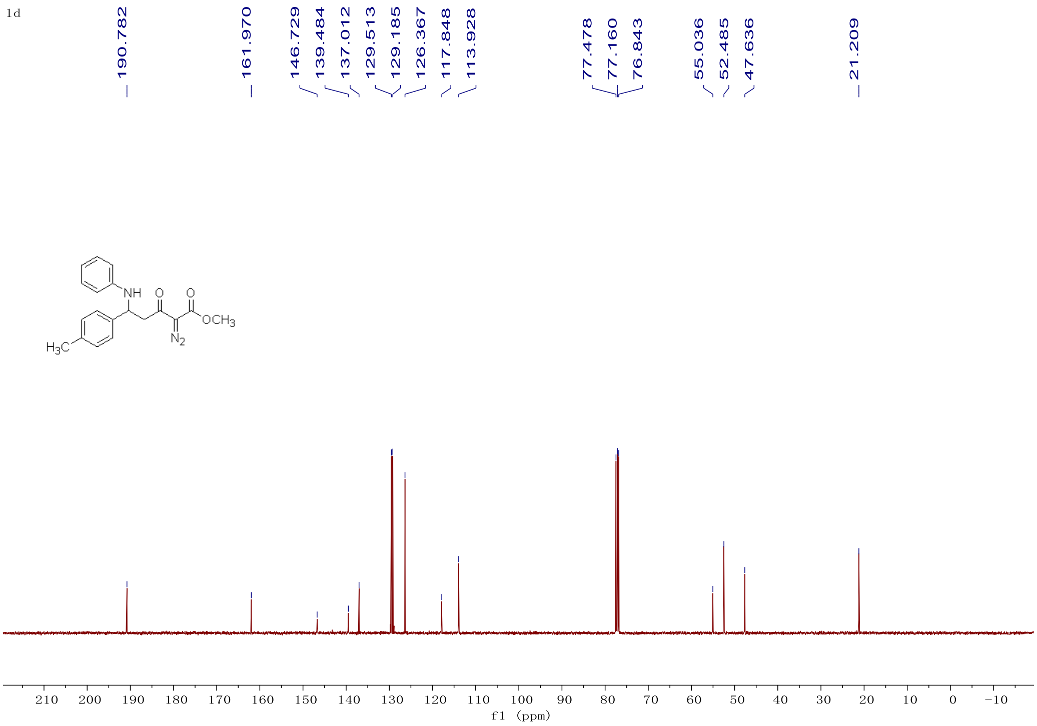

^{13}C NMR spectrum ($CDCl_3$, 100 MHz) of **1d**

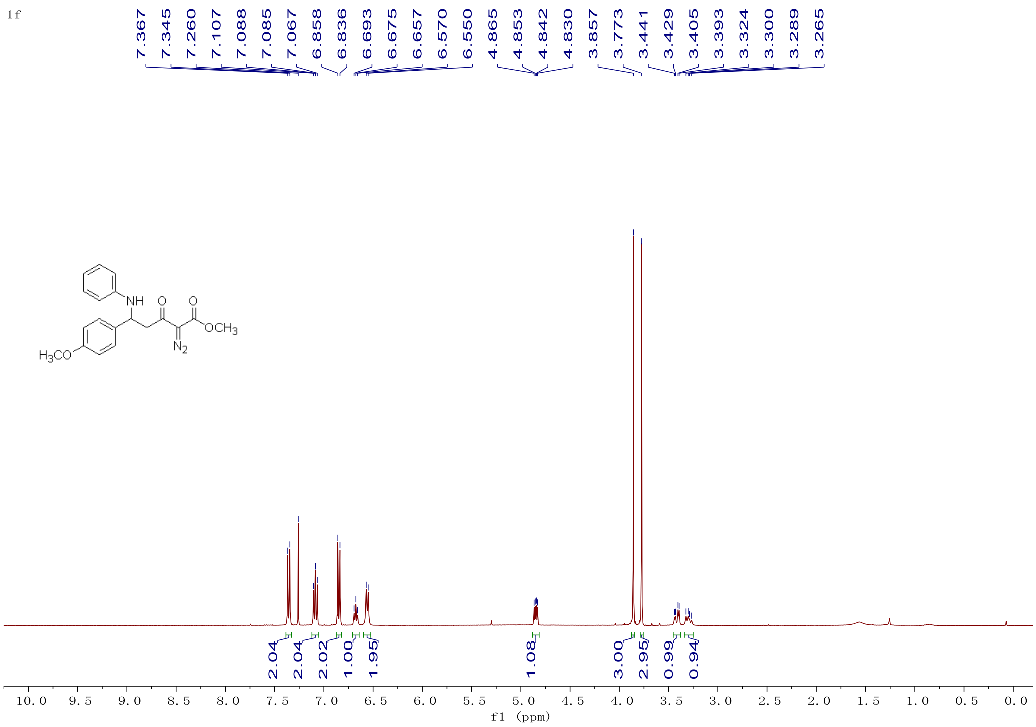

^{1}H NMR spectrum ($CDCl_3$, 400 MHz) of **1f**

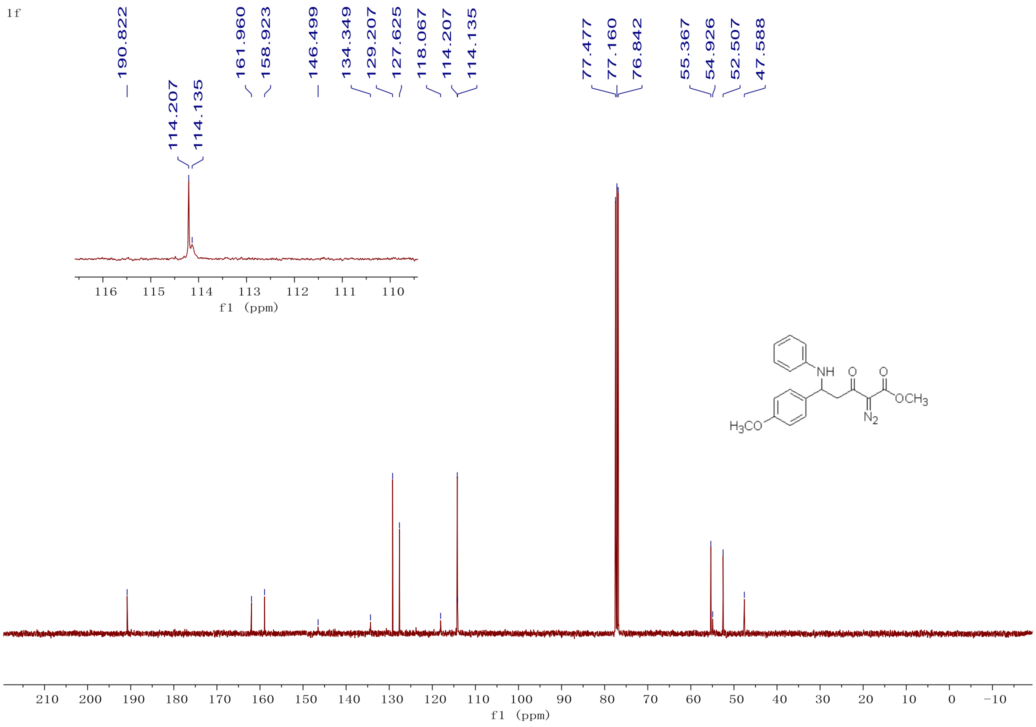

^{13}C NMR spectrum ($CDCl_3$, 100 MHz) of **1f**

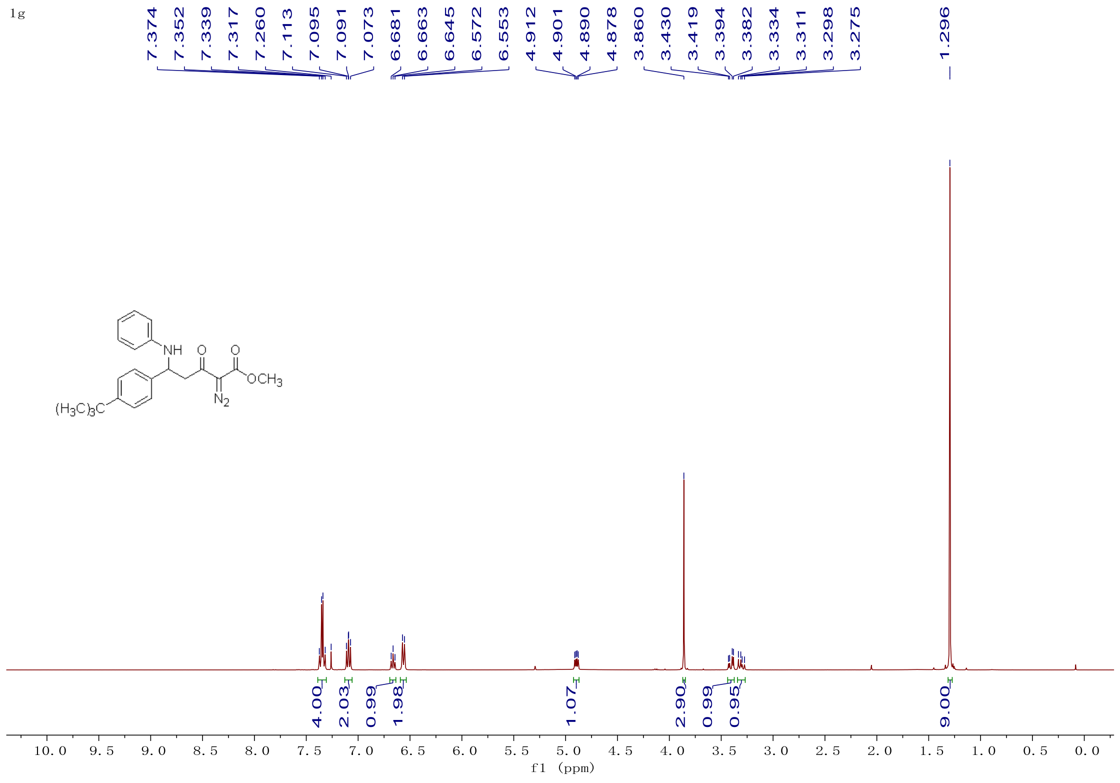

^{1}H NMR spectrum ($CDCl_3$, 400 MHz) of **1g**

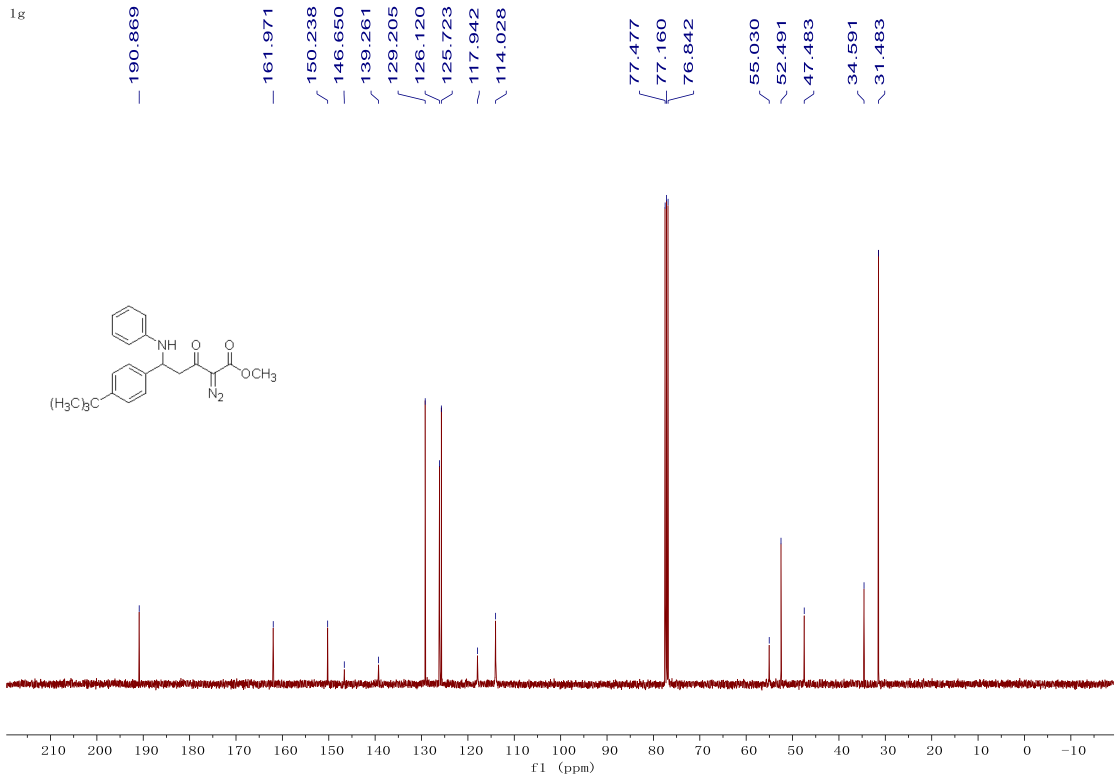

^{13}C NMR spectrum ($CDCl_3$, 100 MHz) of **1g**

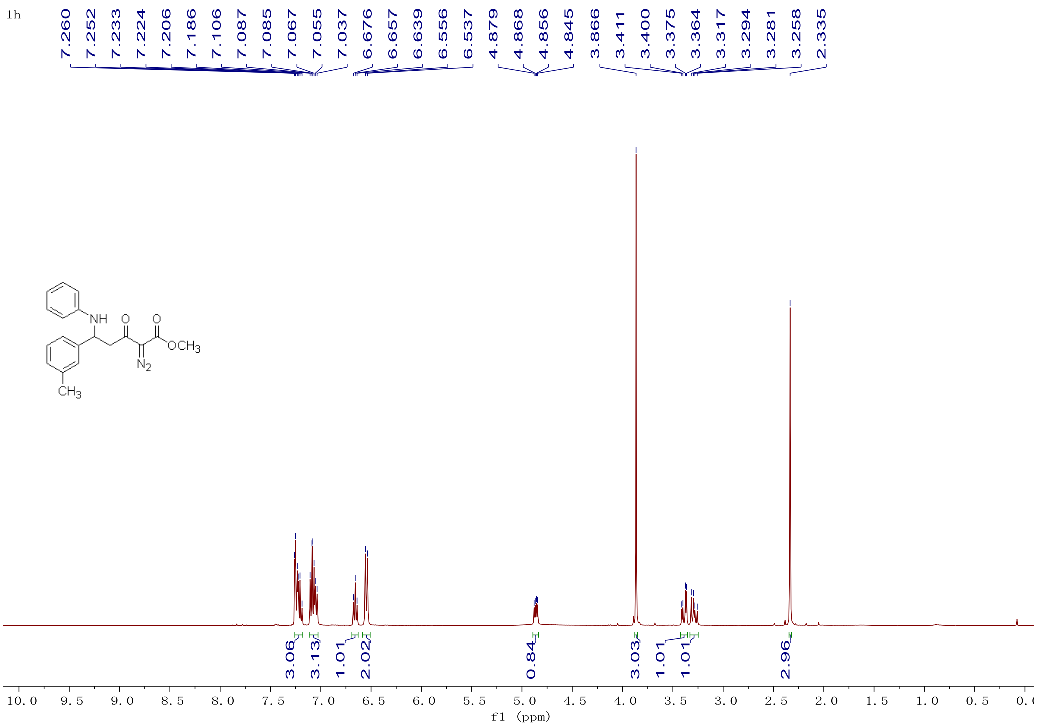

^{1}H NMR spectrum ($CDCl_3$, 400 MHz) of **1h**

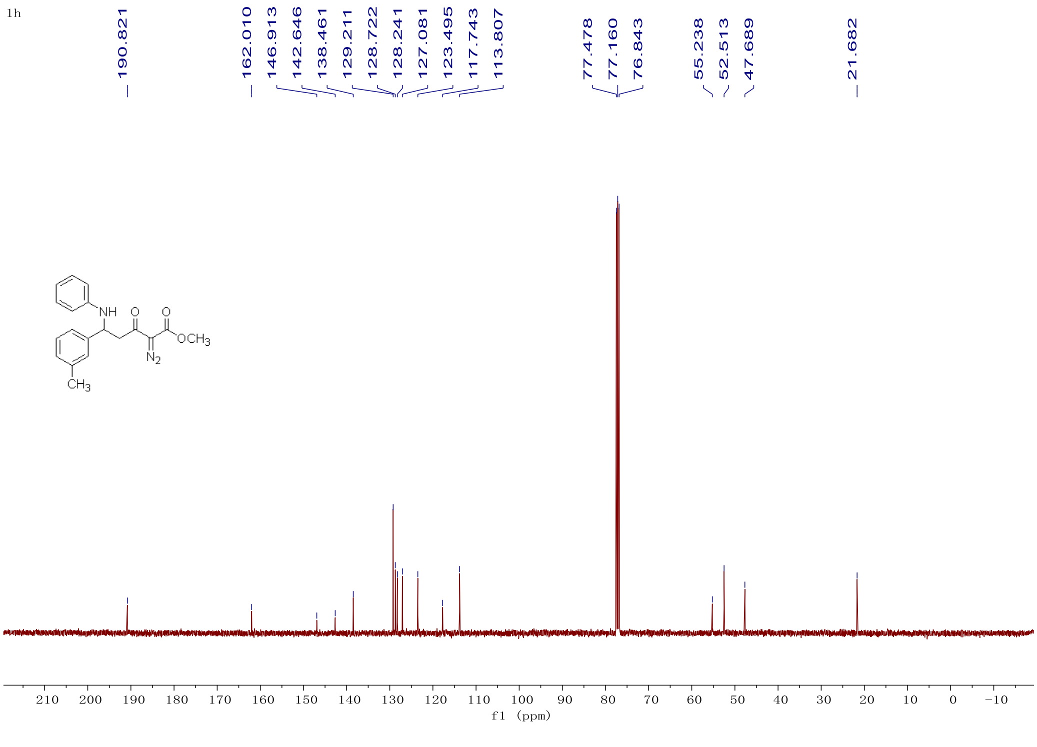

^{13}C NMR spectrum ($CDCl_3$, 100 MHz) of **1h**

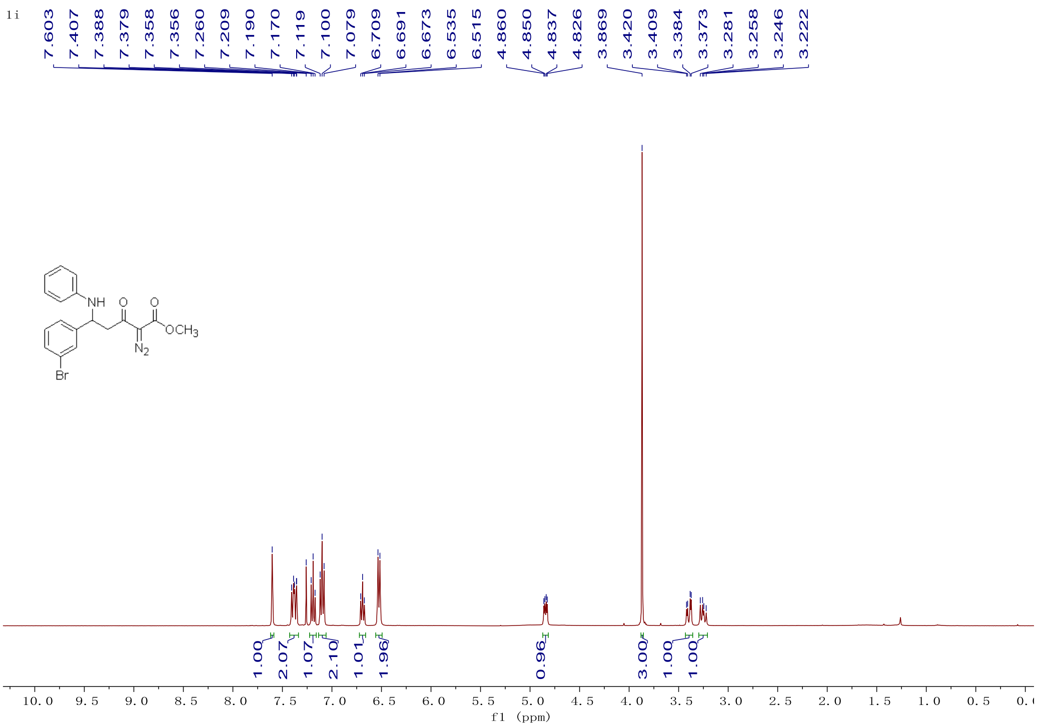

^{1}H NMR spectrum ($CDCl_3$, 400 MHz) of **1i**

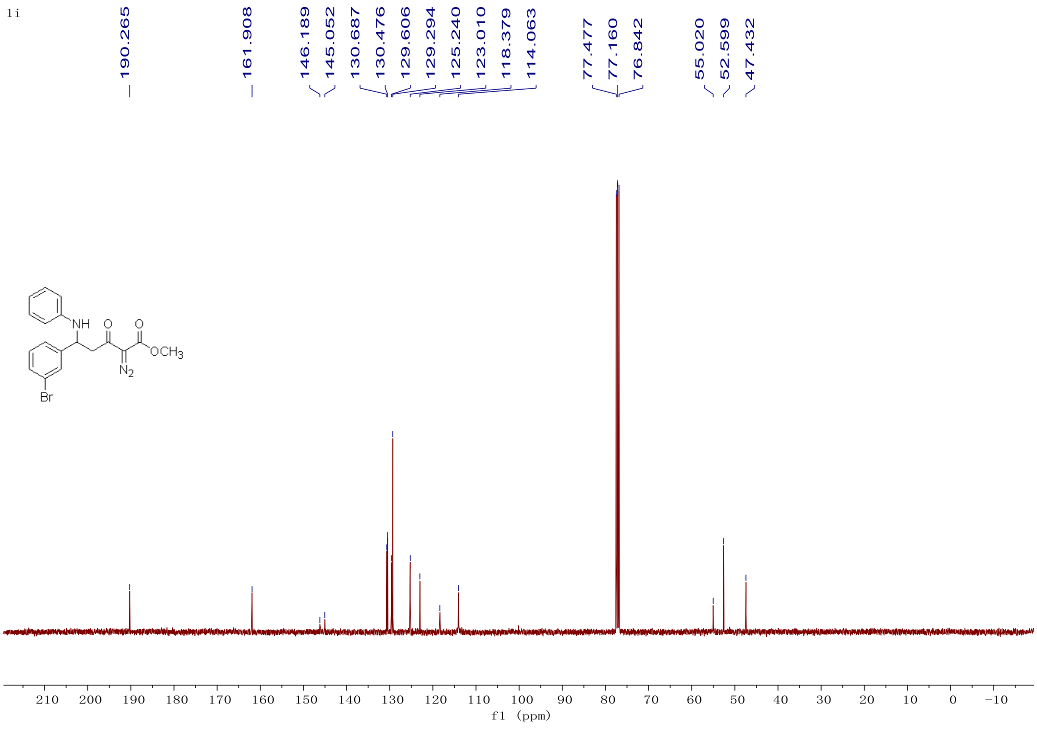

^{13}C NMR spectrum ($CDCl_3$, 100 MHz) of **1i**

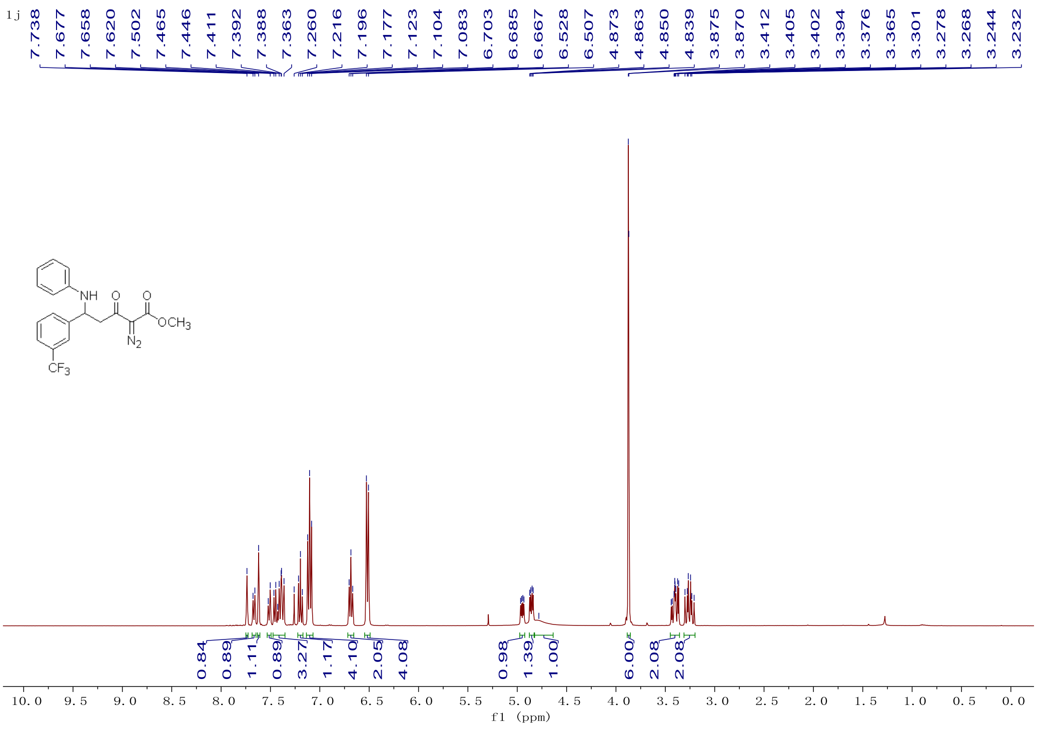

^{1}H NMR spectrum ($CDCl_3$, 400 MHz) of **1j**

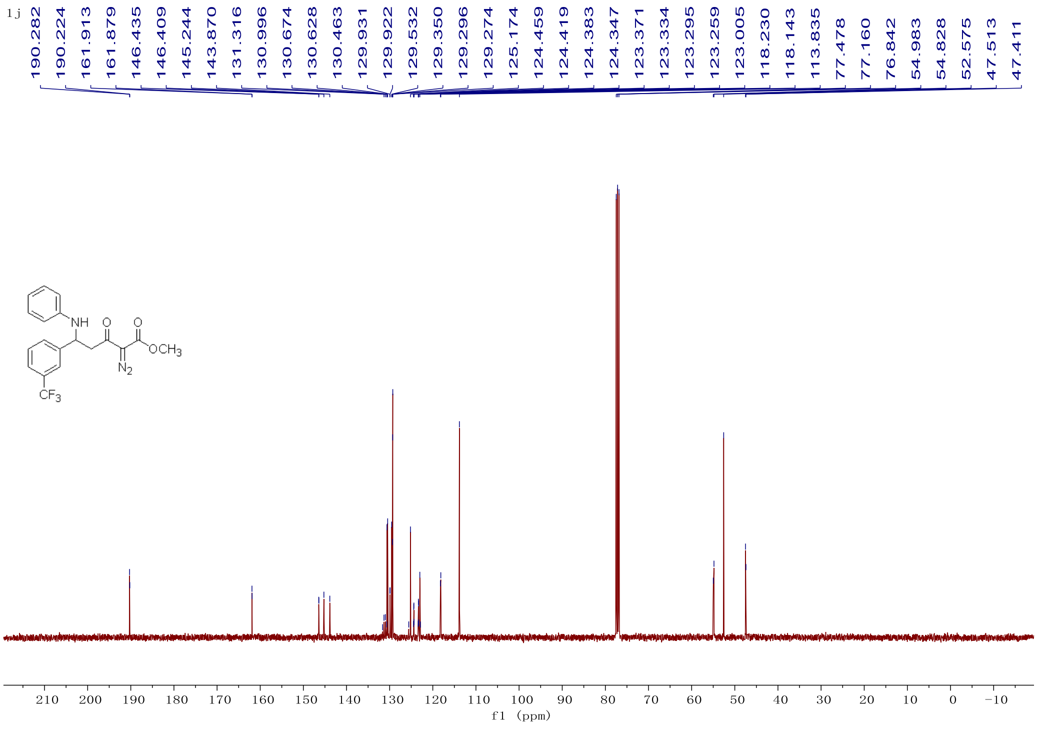

^{13}C NMR spectrum ($CDCl_3$, 100 MHz) of **1j**

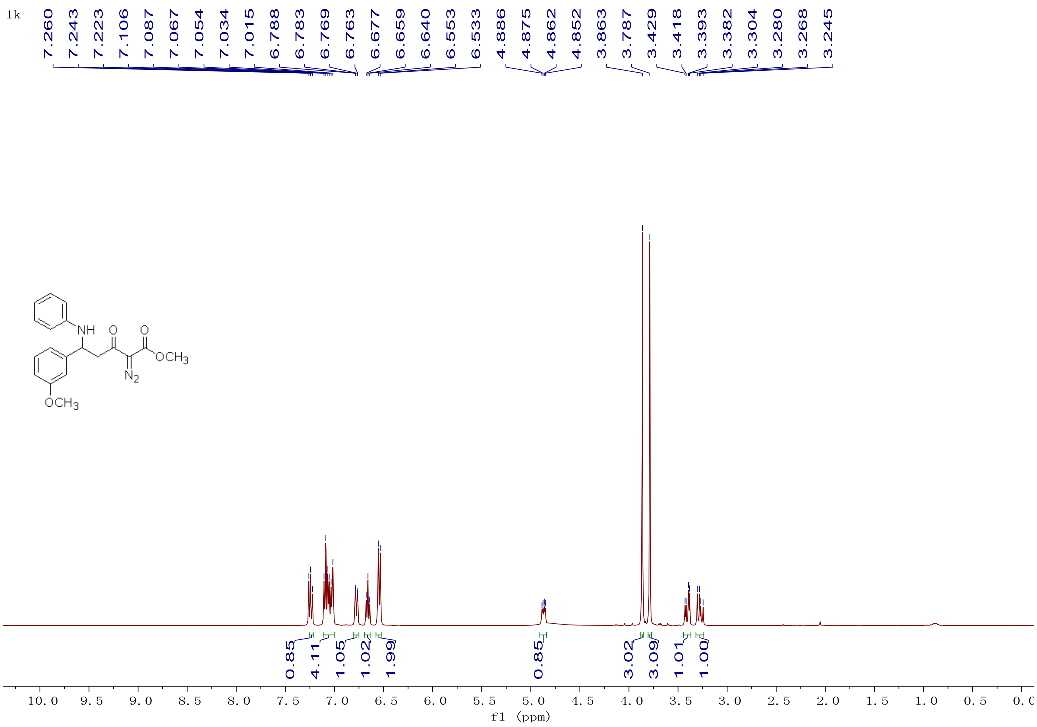

^{1}H NMR spectrum ($CDCl_3$, 400 MHz) of **1k**

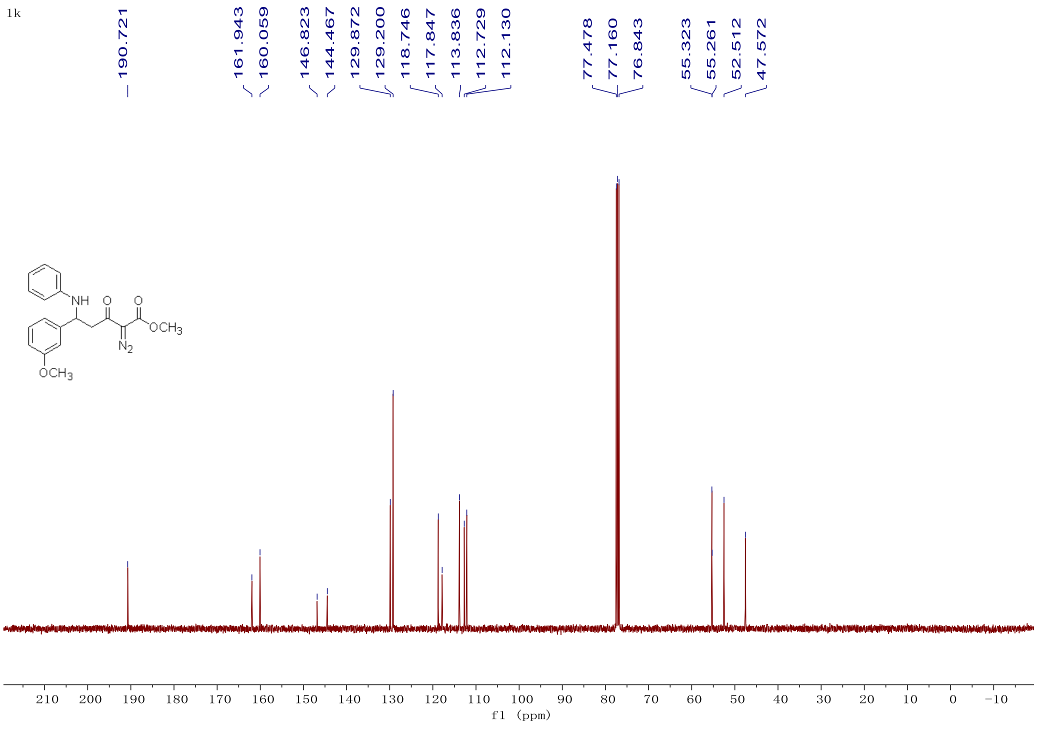

^{13}C NMR spectrum ($CDCl_3$, 100 MHz) of **1k**

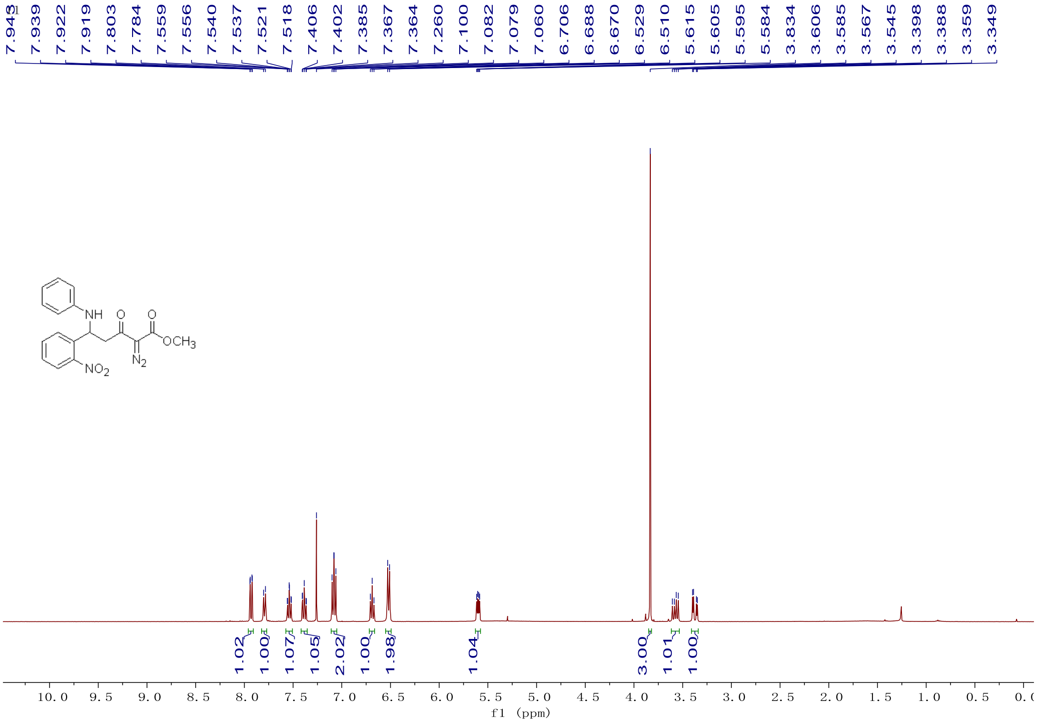

^{1}H NMR spectrum (CDCl$_3$, 400 MHz) of **1l**

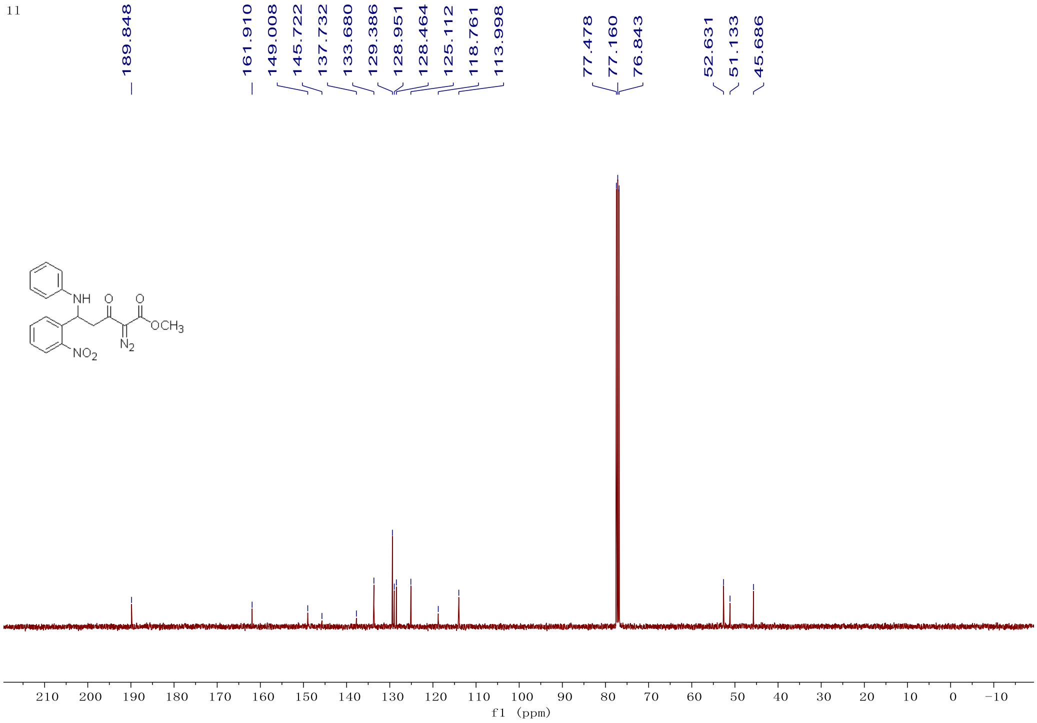

^{13}C NMR spectrum (CDCl$_3$, 100 MHz) of **1l**

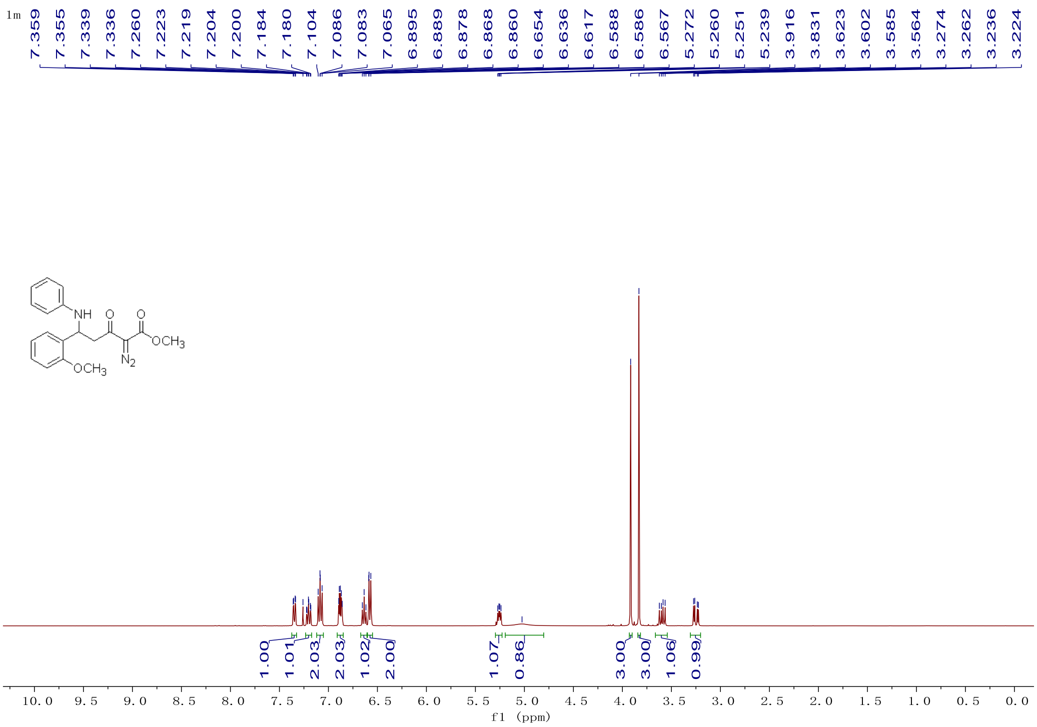

^{1}H NMR spectrum ($CDCl_3$, 400 MHz) of **1m**

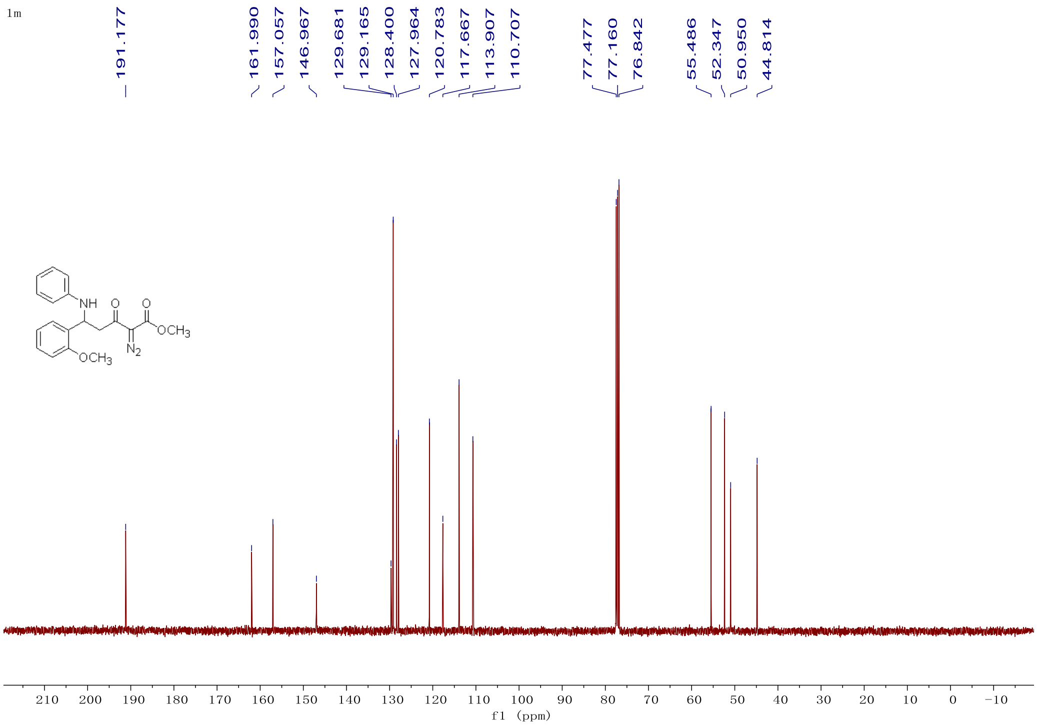

^{13}C NMR spectrum ($CDCl_3$, 100 MHz) of **1m**

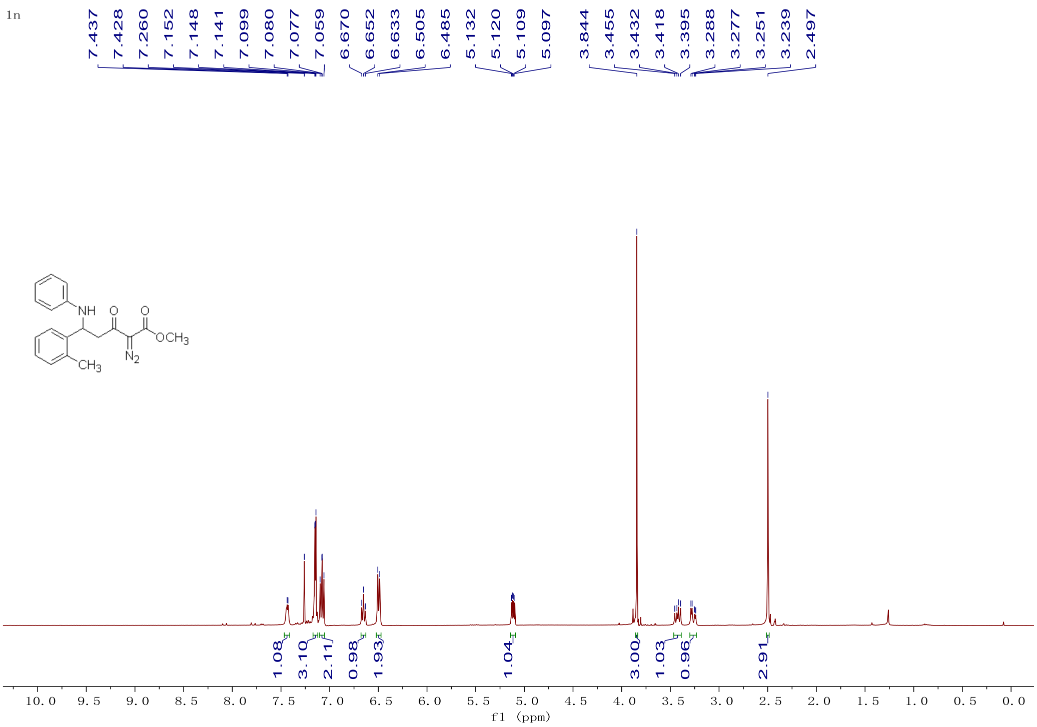

^{1}H NMR spectrum (CDCl₃, 400 MHz) of **1n**

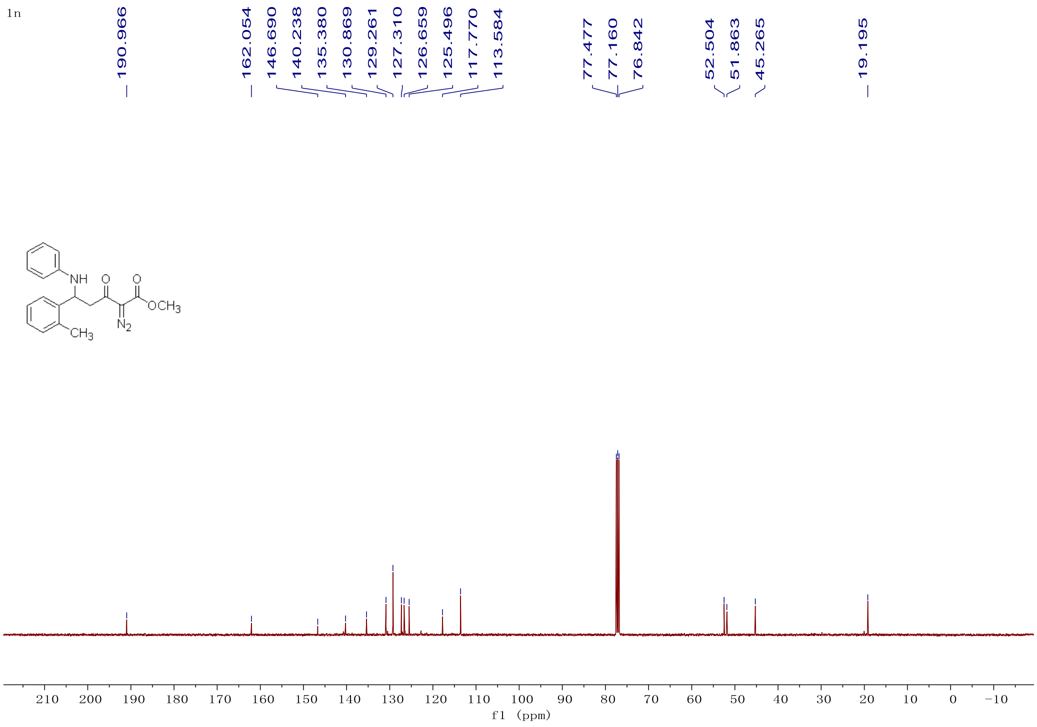

^{13}C NMR spectrum ($CDCl_3$, 100 MHz) of **1n**

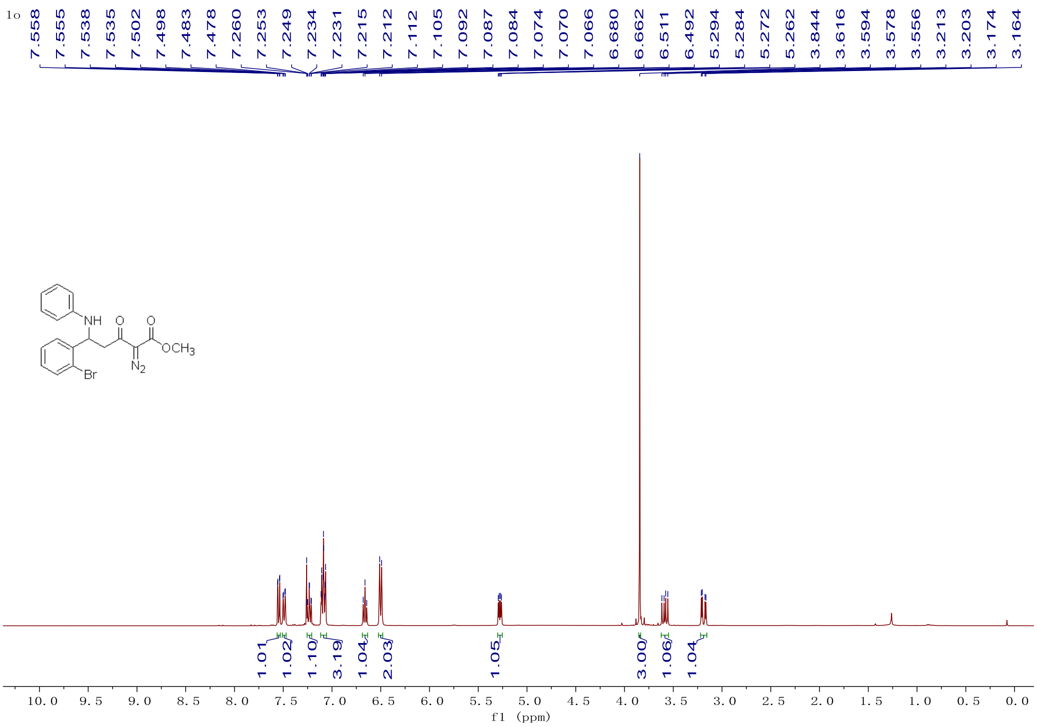

^{1}H NMR spectrum ($CDCl_3$, 400 MHz) of **1o**

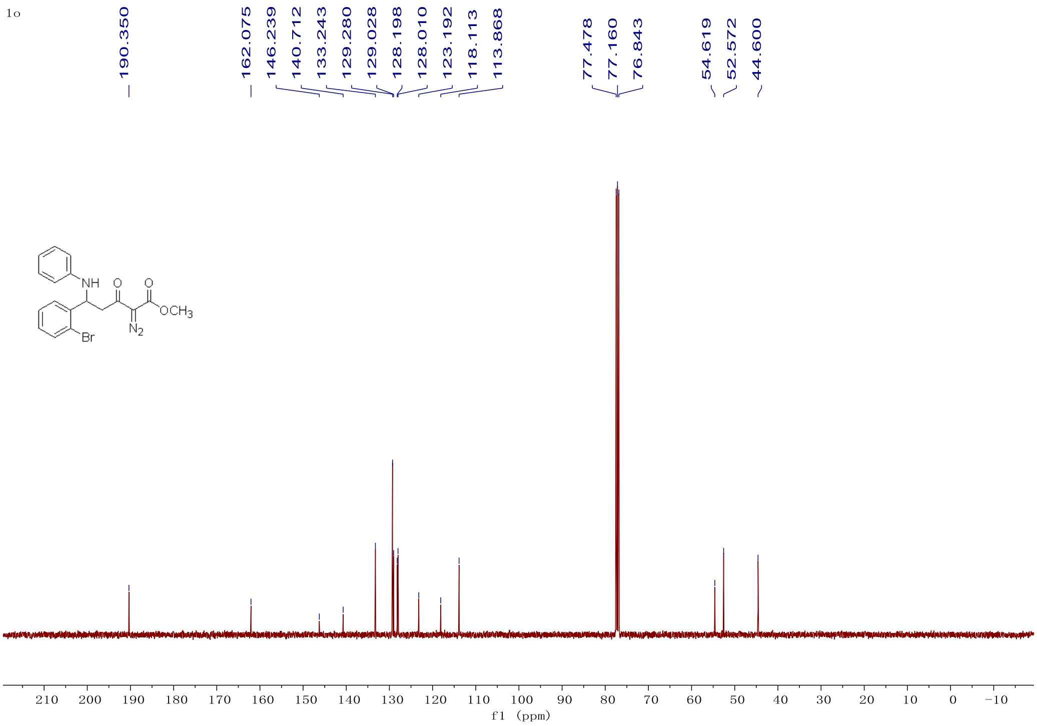

^{13}C NMR spectrum ($CDCl_3$, 100 MHz) of **1o**

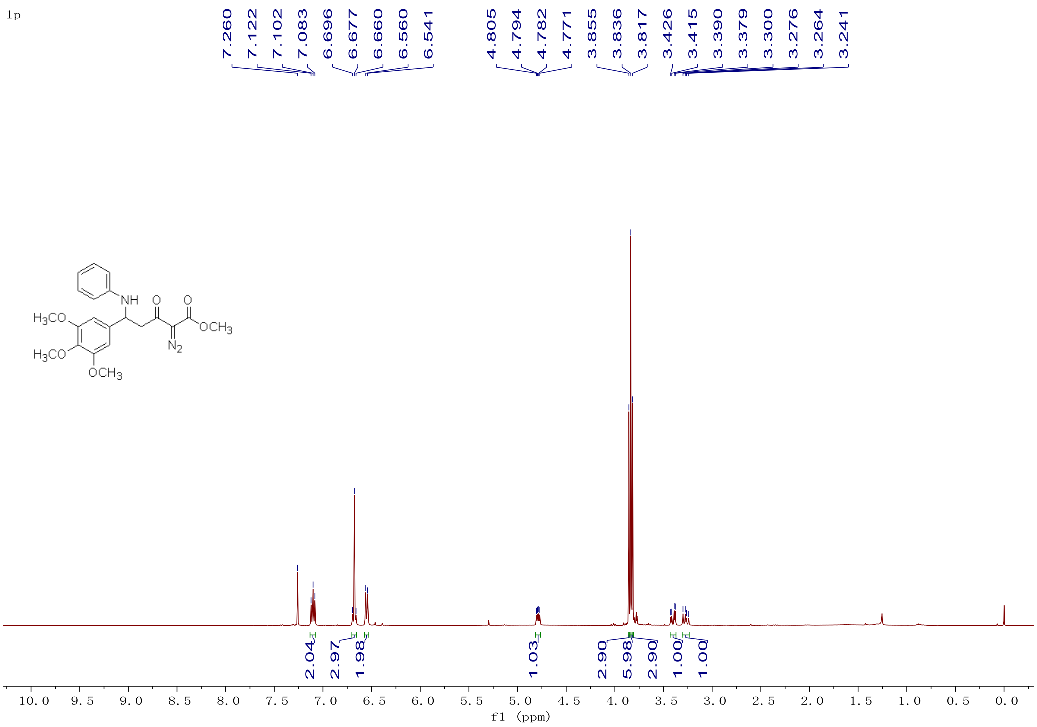

^{1}H NMR spectrum (CDCl$_3$, 400 MHz) of **1p**

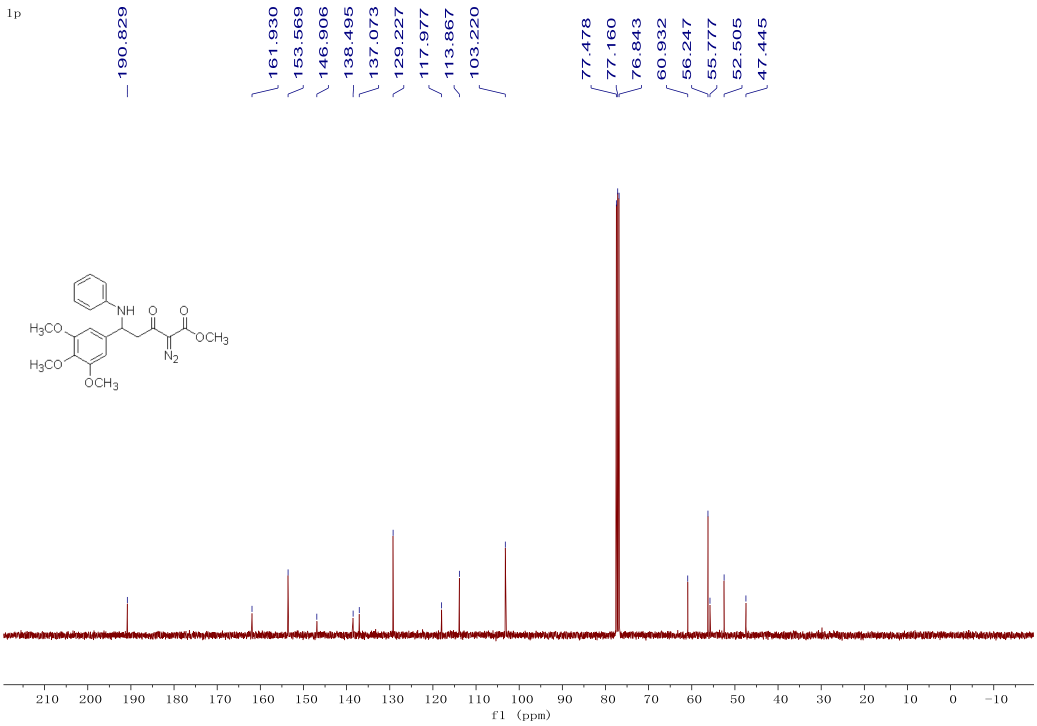

^{13}C NMR spectrum (CDCl$_3$, 100 MHz) of **1p**

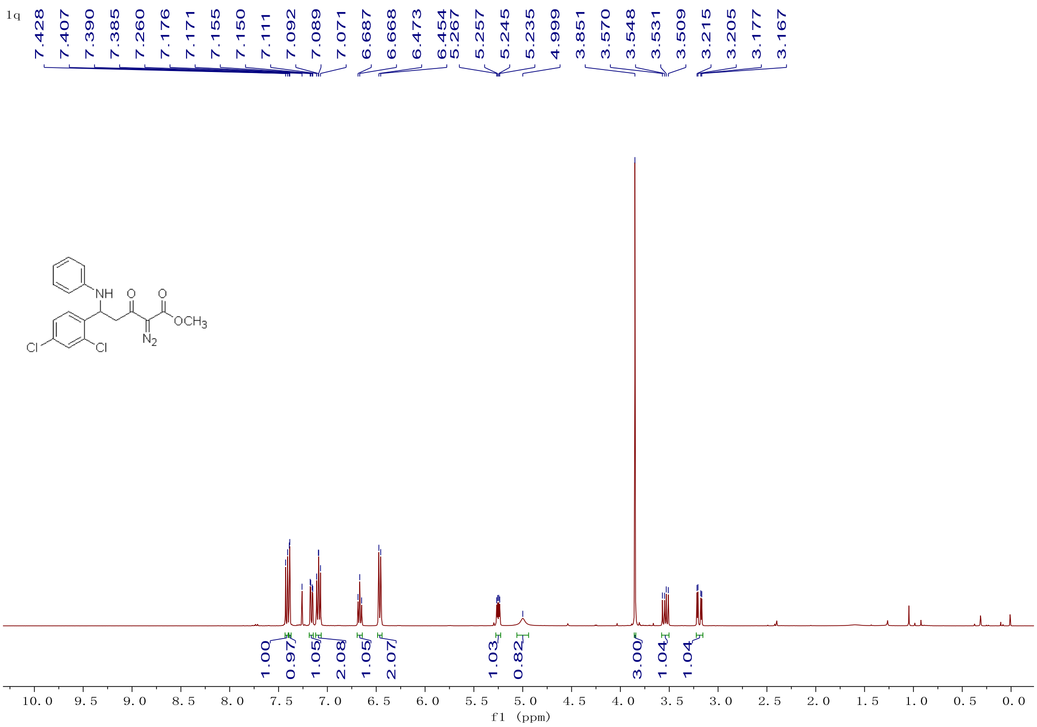

^{1}H NMR spectrum (CDCl$_3$, 400 MHz) of **1q**

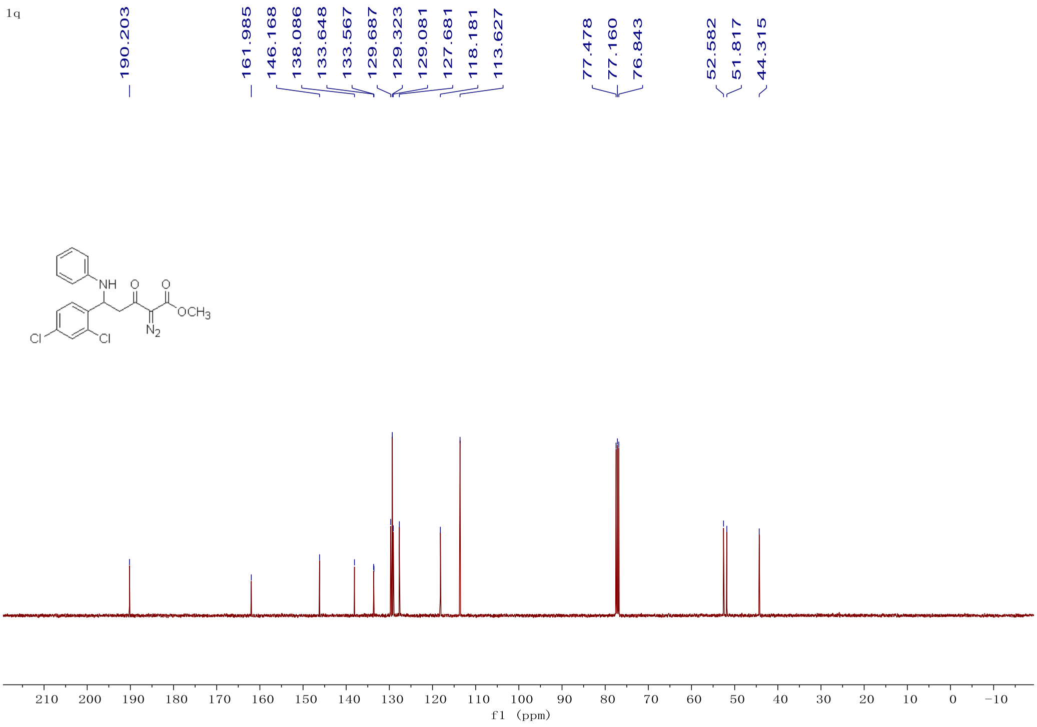

^{13}C NMR spectrum (CDCl$_3$, 100 MHz) of **1q**

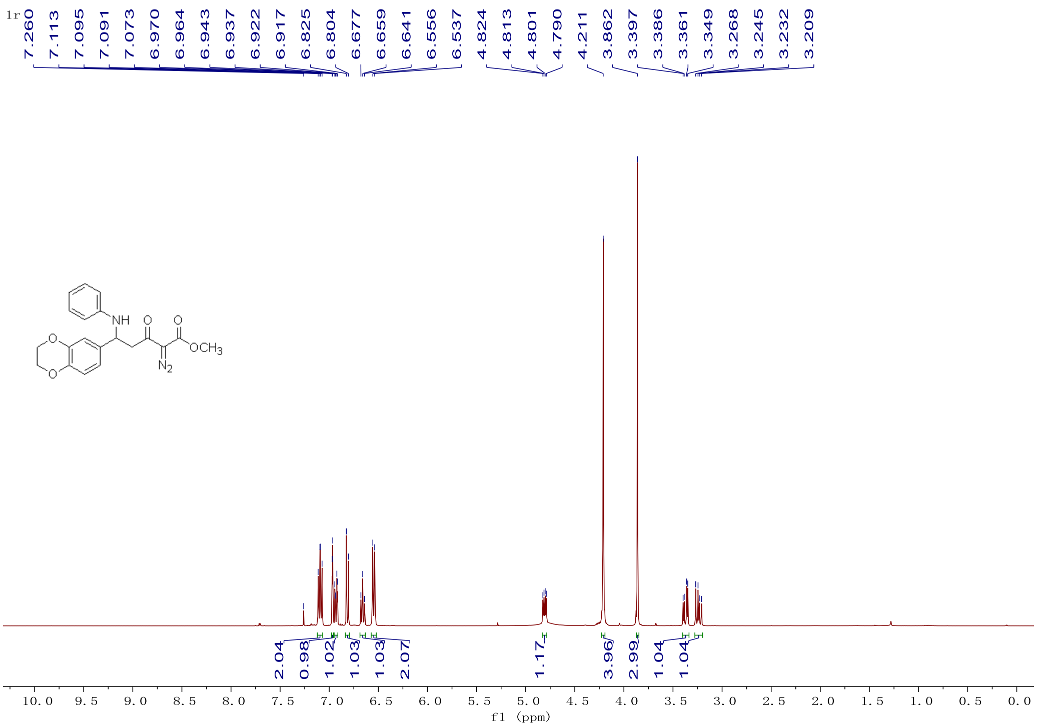

^{1}H NMR spectrum ($CDCl_3$, 400 MHz) of **1r**

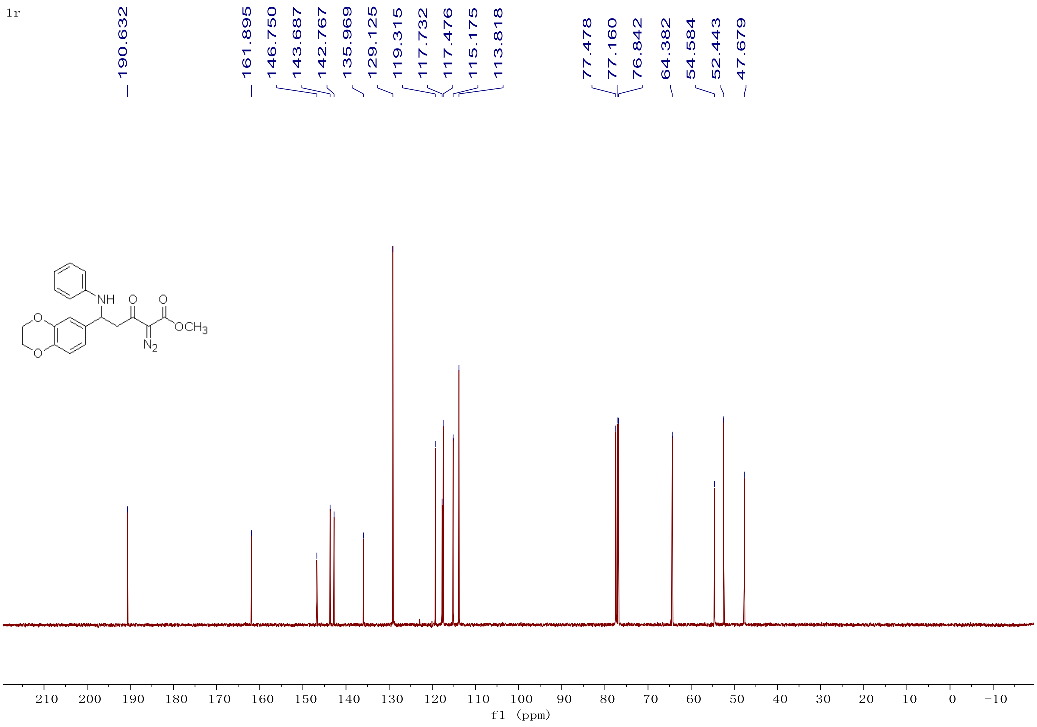

^{13}C NMR spectrum ($CDCl_3$, 100 MHz) of **1r**

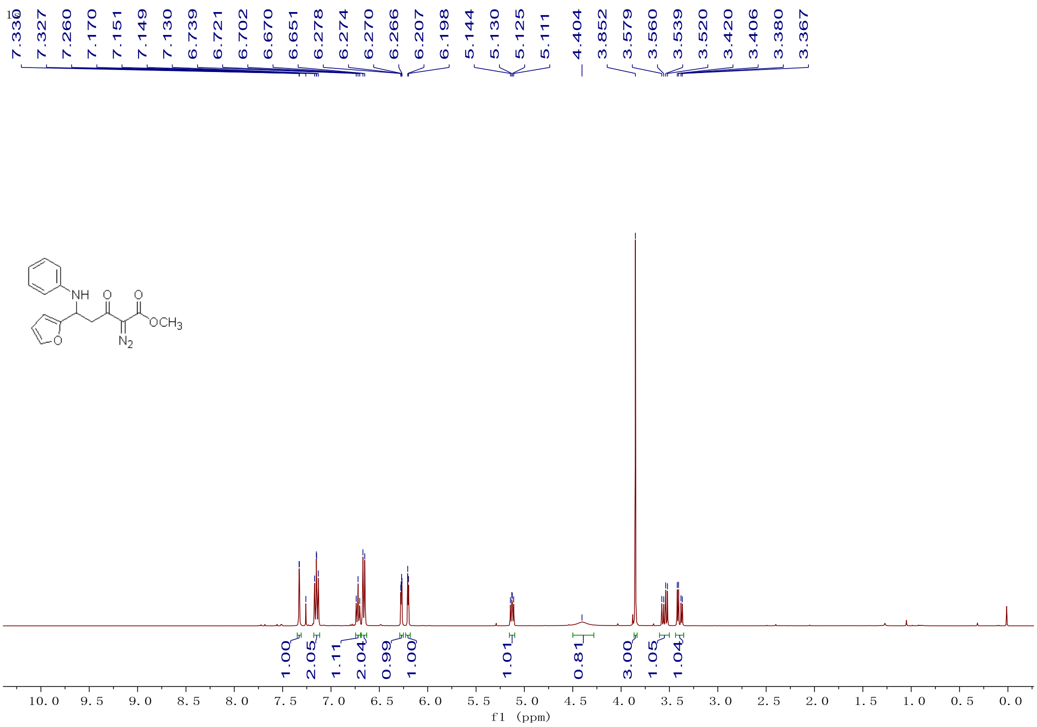

^{1}H NMR spectrum ($CDCl_3$, 400 MHz) of **1s**

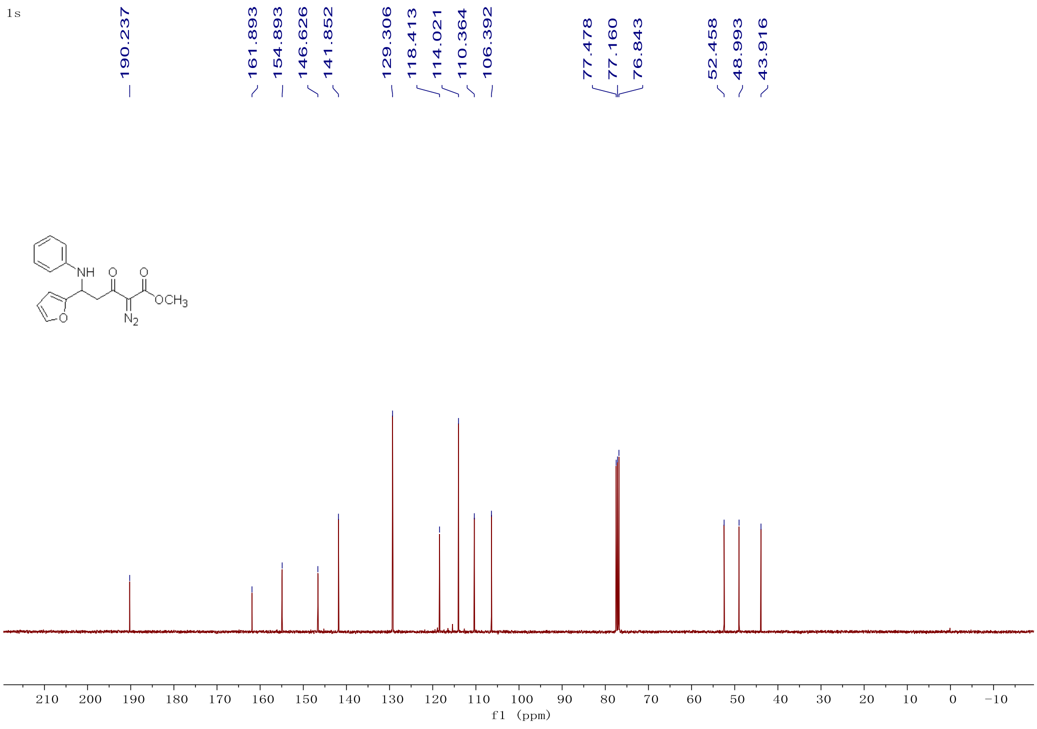

^{13}C NMR spectrum ($CDCl_3$, 100 MHz) of **1s**

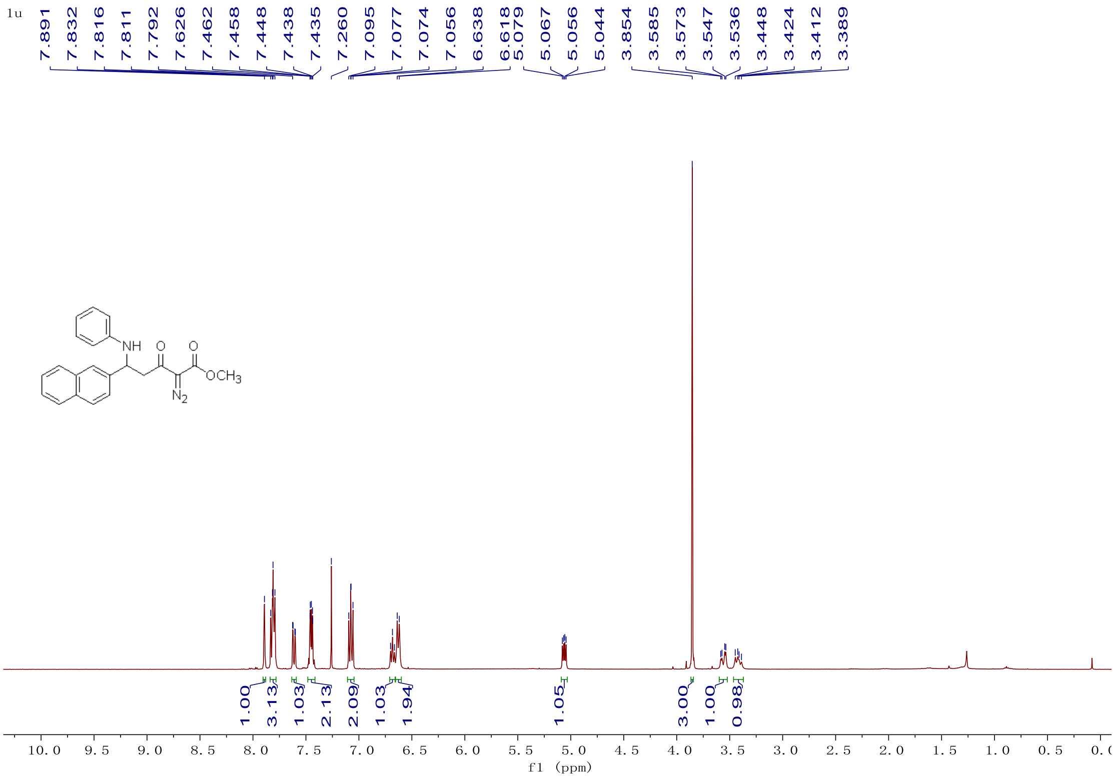

^{1}H NMR spectrum (CDCl3, 400 MHz) of **1u**

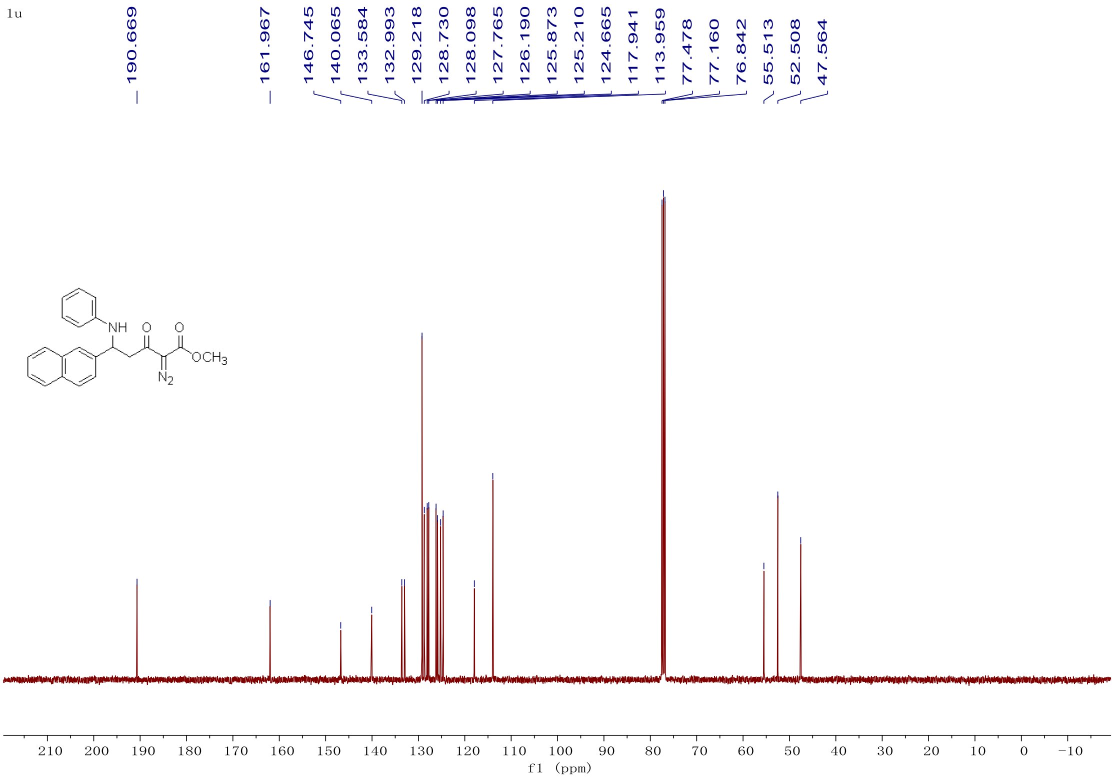

^{13}C NMR spectrum (CDCl3, 100 MHz) of **1u**

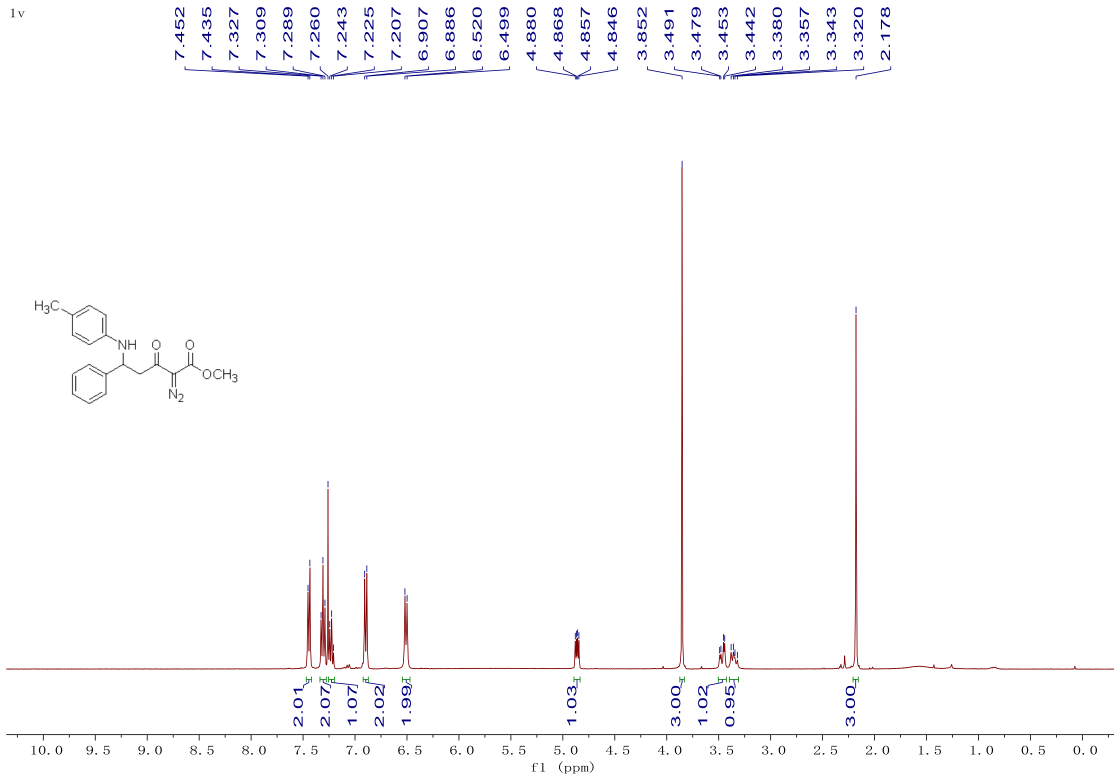

^{1}H NMR spectrum ($CDCl_3$, 400 MHz) of **1v**

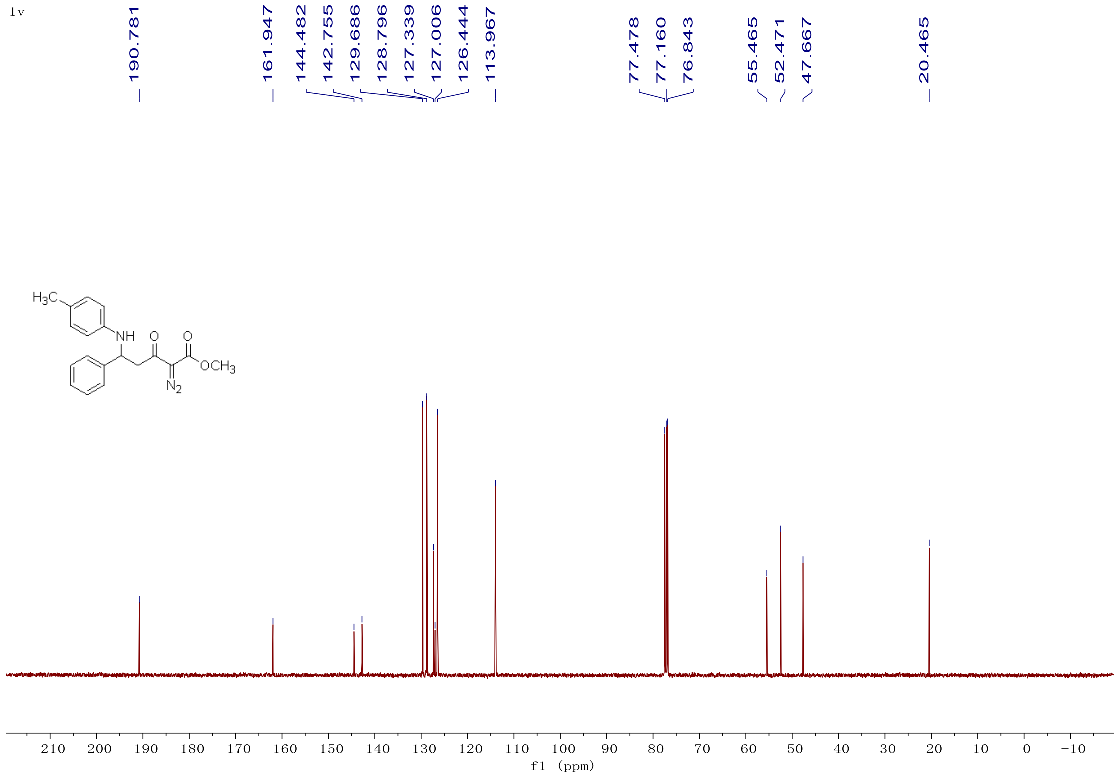

^{13}C NMR spectrum ($CDCl_3$, 100 MHz) of **1v**

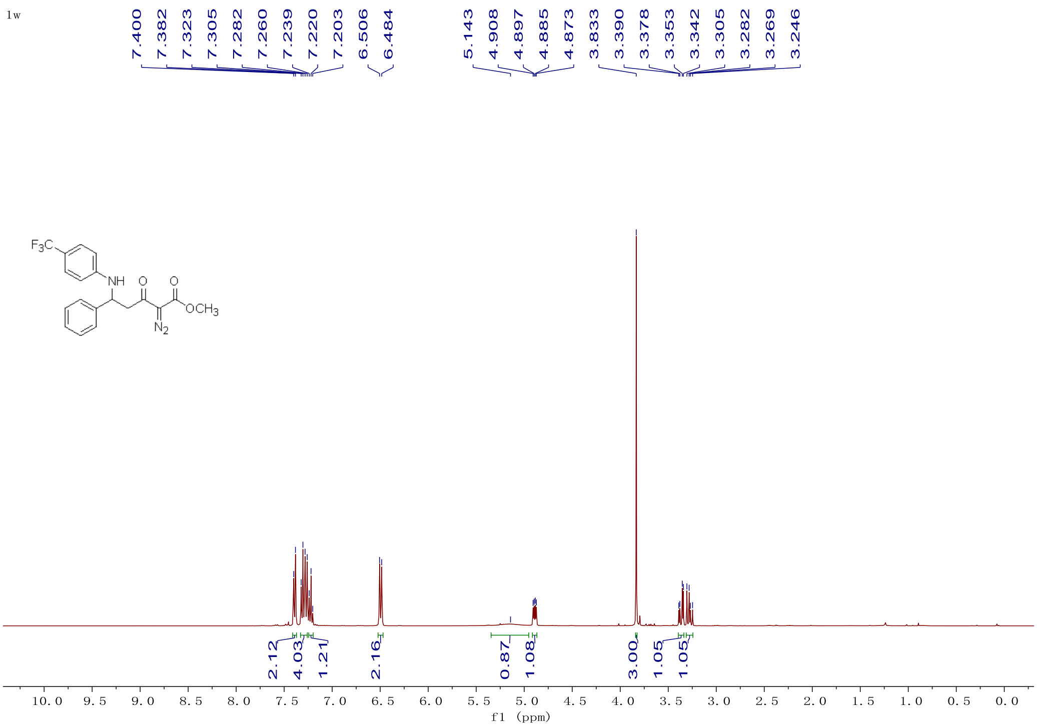

^{1}H NMR spectrum ($CDCl_3$, 400 MHz) of **1w**

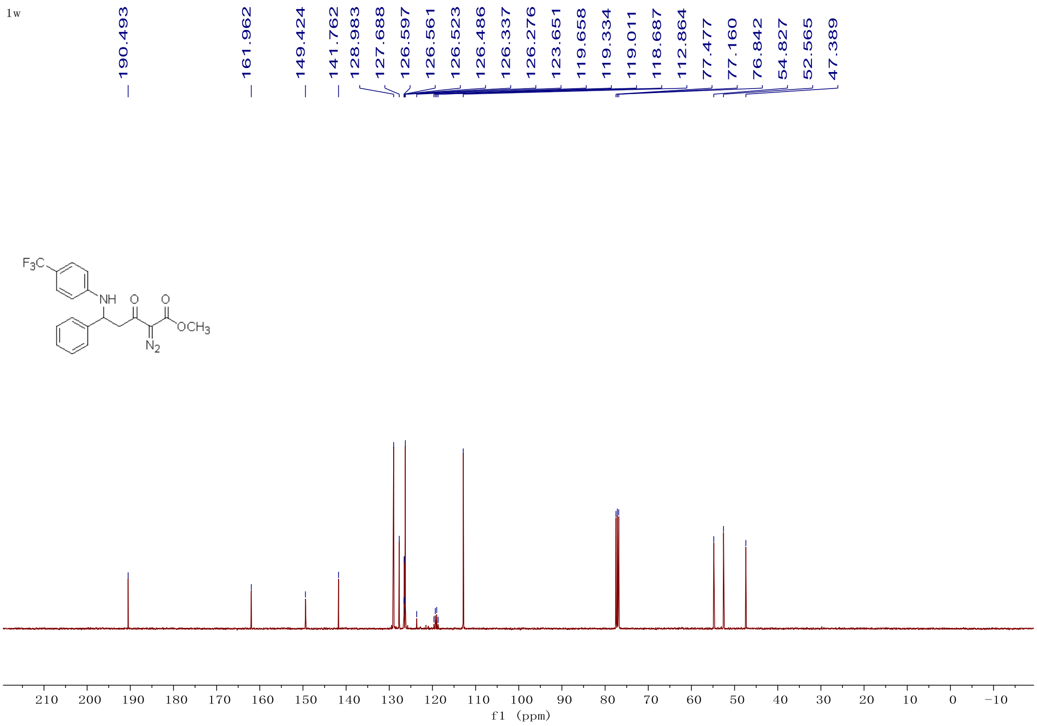

^{13}C NMR spectrum ($CDCl_3$, 100 MHz) of **1w**

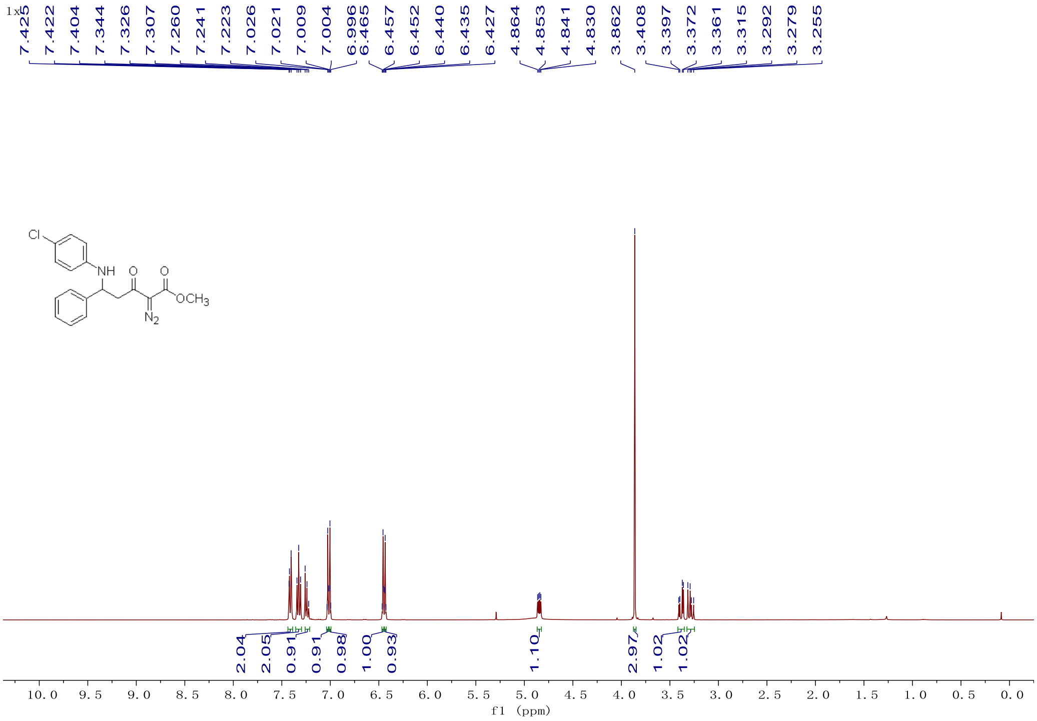

^{1}H NMR spectrum (CDCl$_3$, 400 MHz) of **1x**

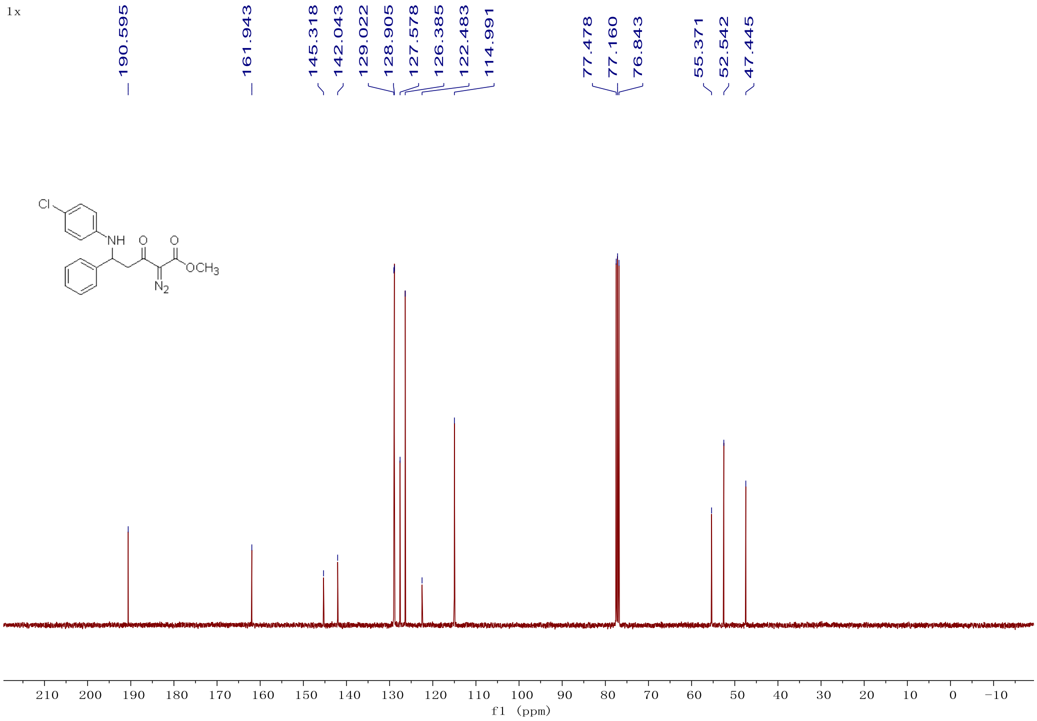

^{13}C NMR spectrum (CDCl$_3$, 100 MHz) of **1x**

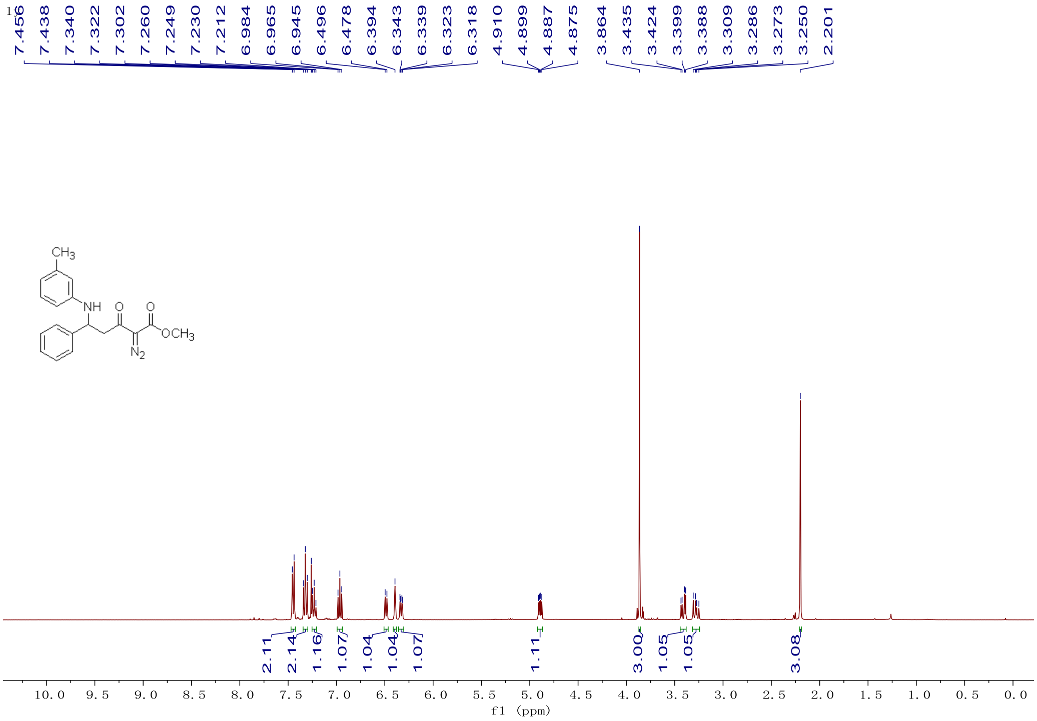

^{1}H NMR spectrum ($CDCl_3$, 400 MHz) of **1y**

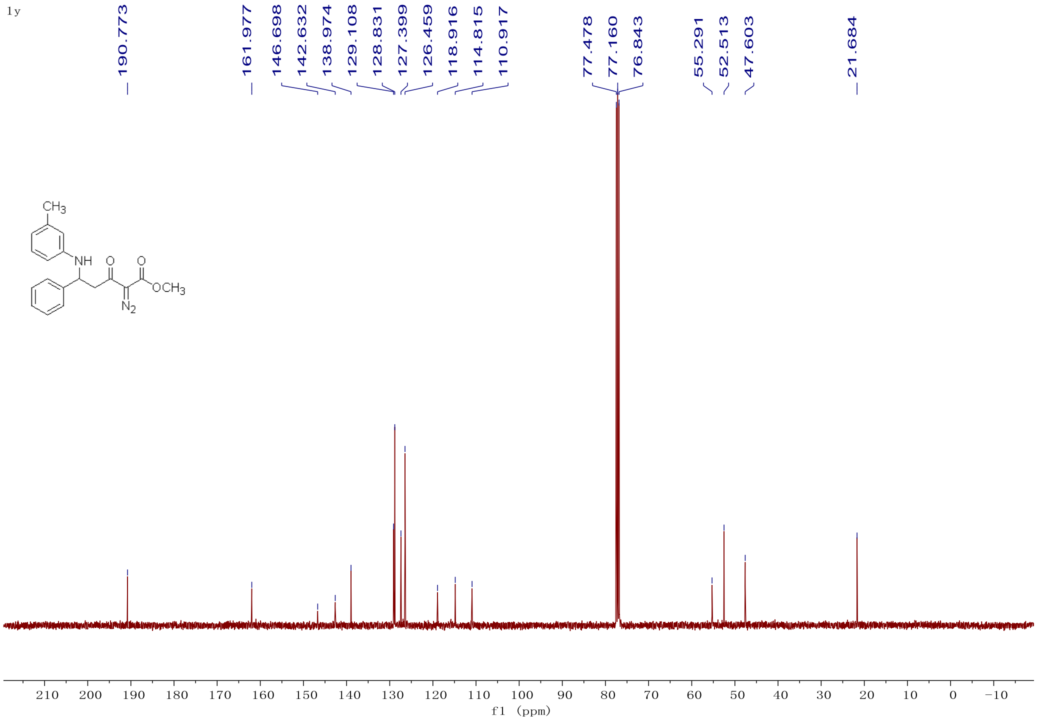

^{13}C NMR spectrum ($CDCl_3$, 100 MHz) of **1y**

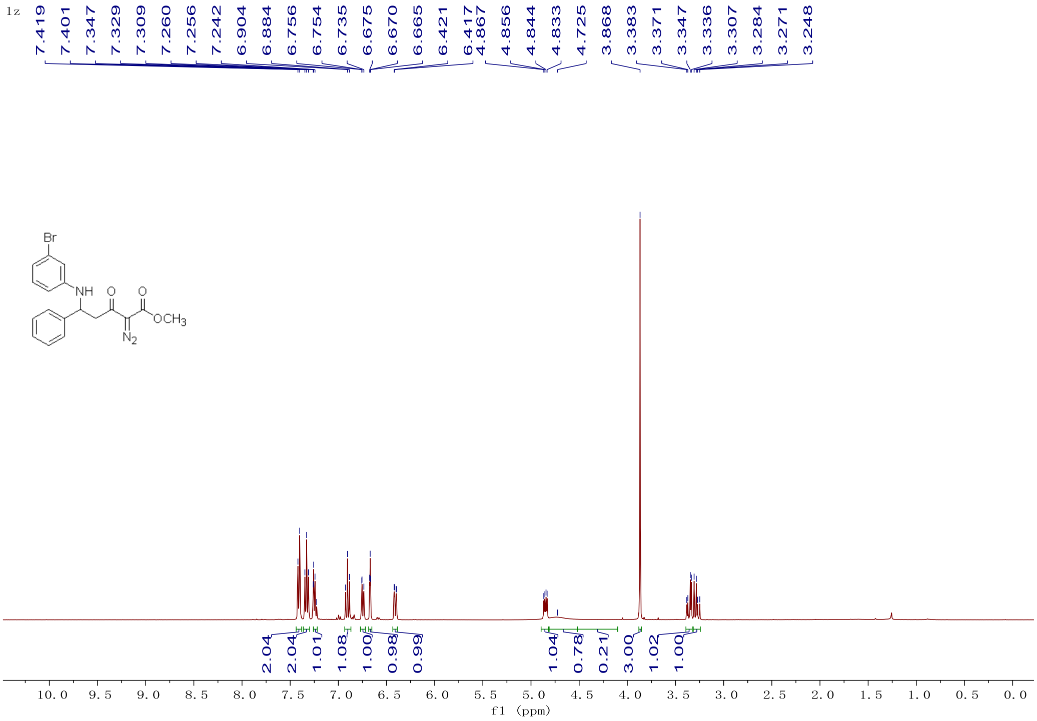

^{1}H NMR spectrum ($CDCl_3$, 400 MHz) of **1z**

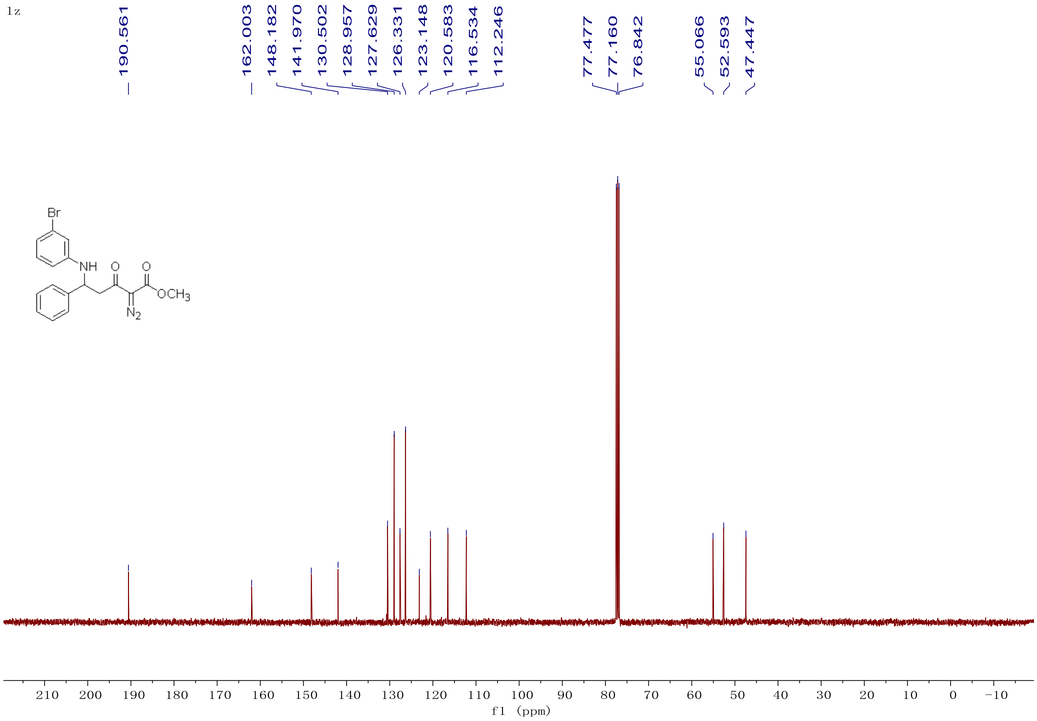

^{13}C NMR spectrum ($CDCl_3$, 100 MHz) of **1z**

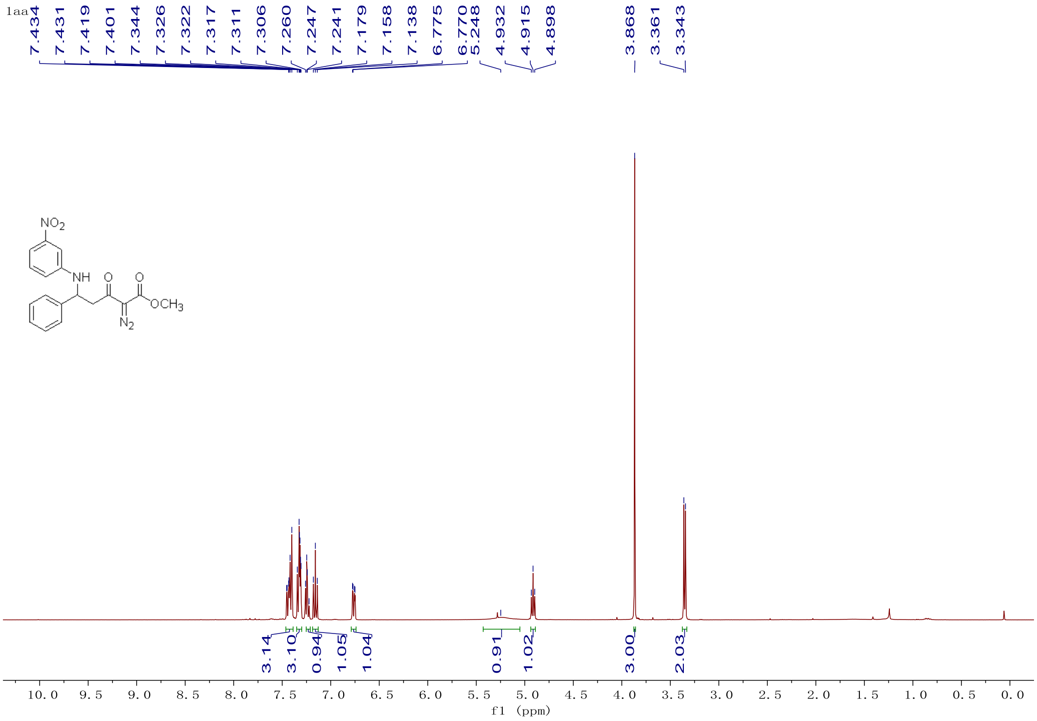

^{1}H NMR spectrum ($CDCl_3$, 400 MHz) of **1aa**

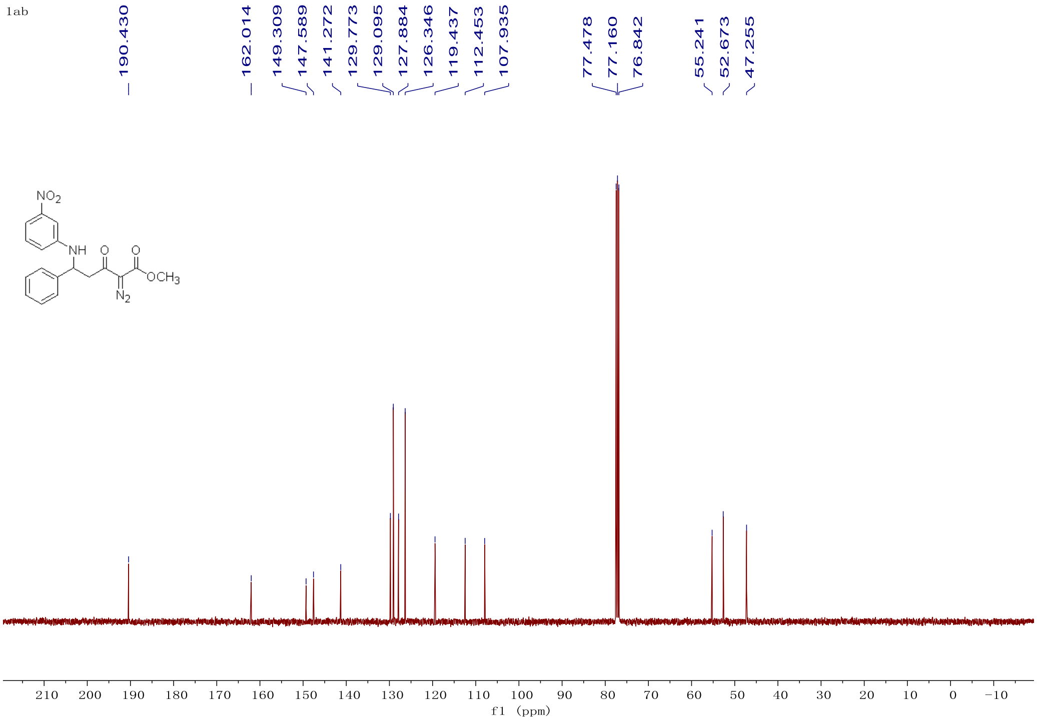

^{13}C NMR spectrum ($CDCl_3$, 100 MHz) of **1aa**

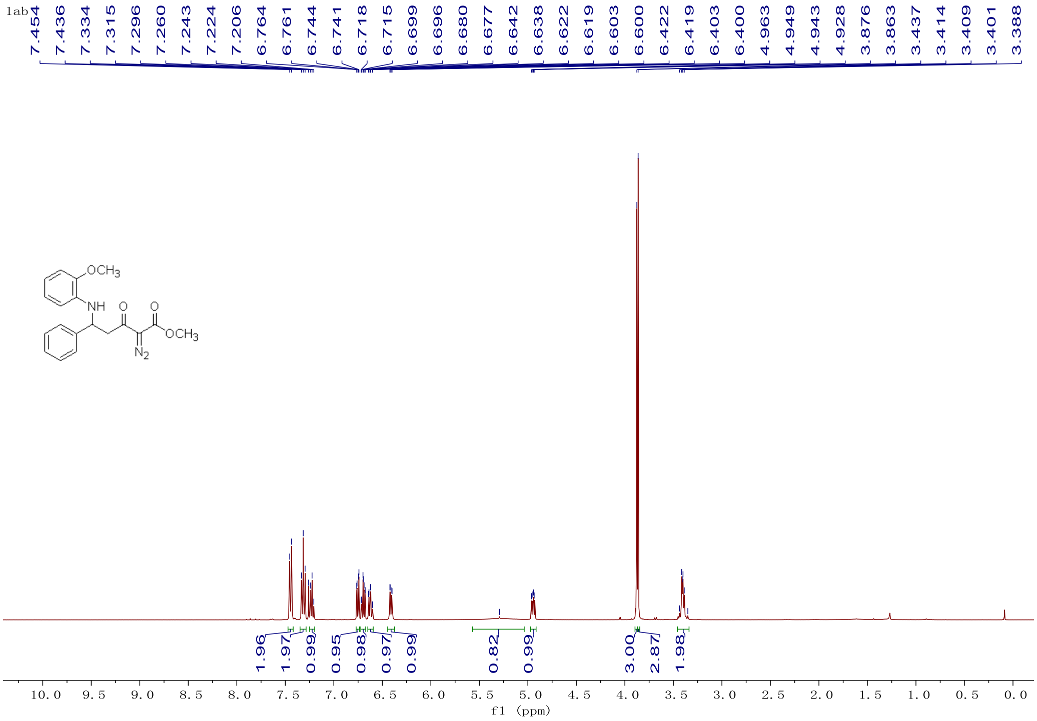

^{1}H NMR spectrum (CDCl$_3$, 400 MHz) of **1ab**

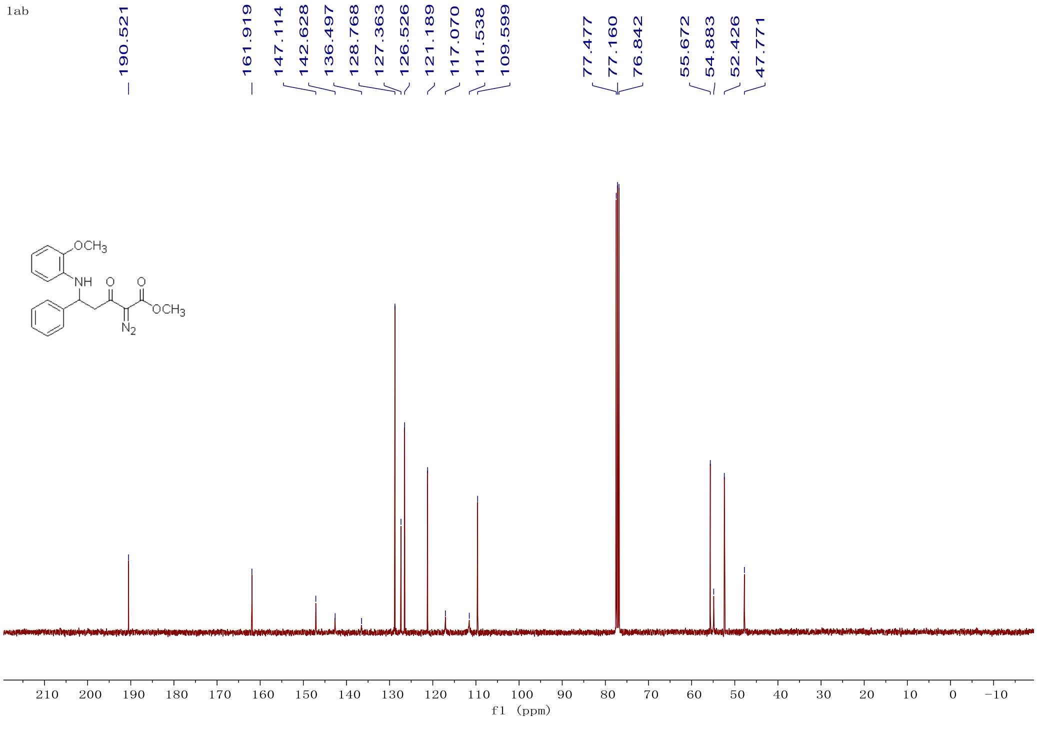

^{13}C NMR spectrum (CDCl$_3$, 100 MHz) of **1ab**

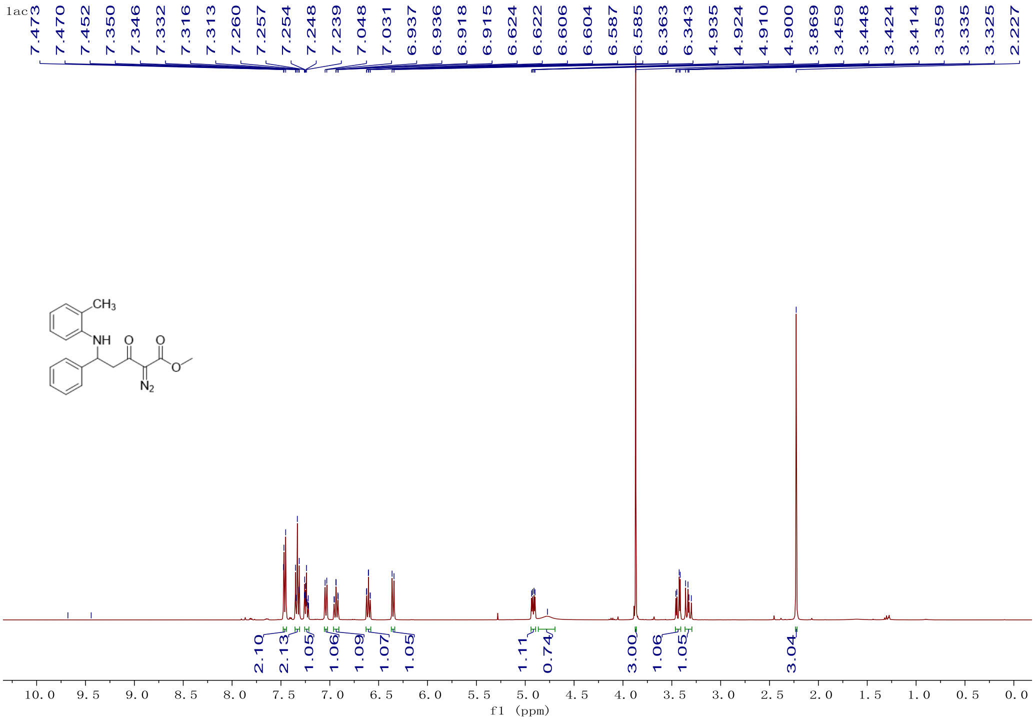

^{1}H NMR spectrum (CDCl$_3$, 400 MHz) of **1ac**

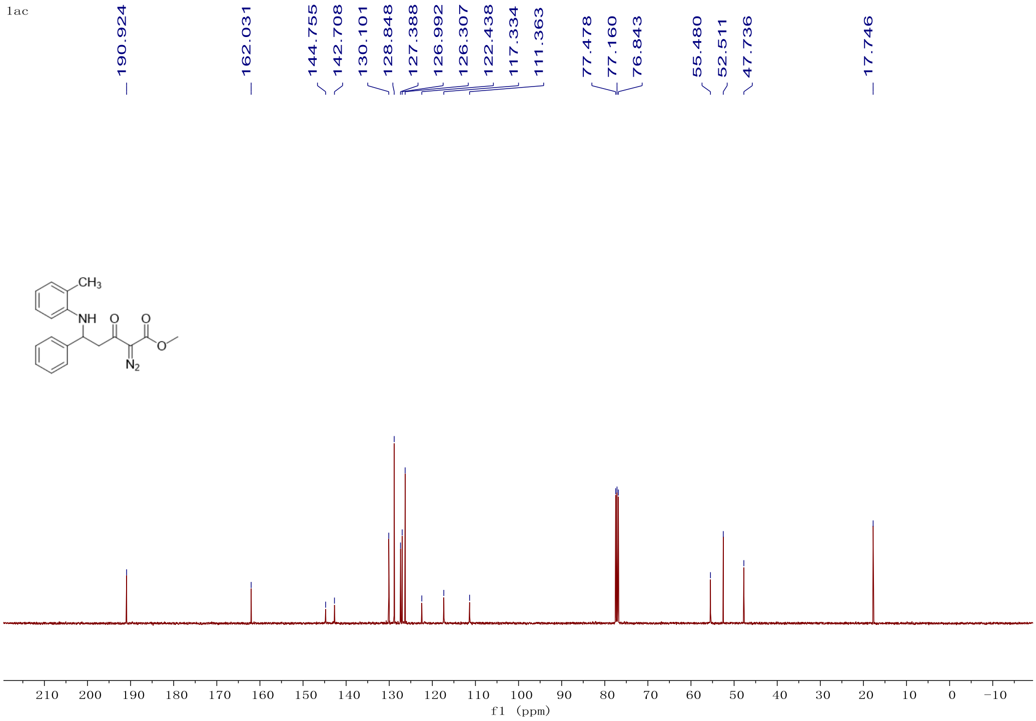

^{13}C NMR spectrum (CDCl$_3$, 100 MHz) of **1ac**

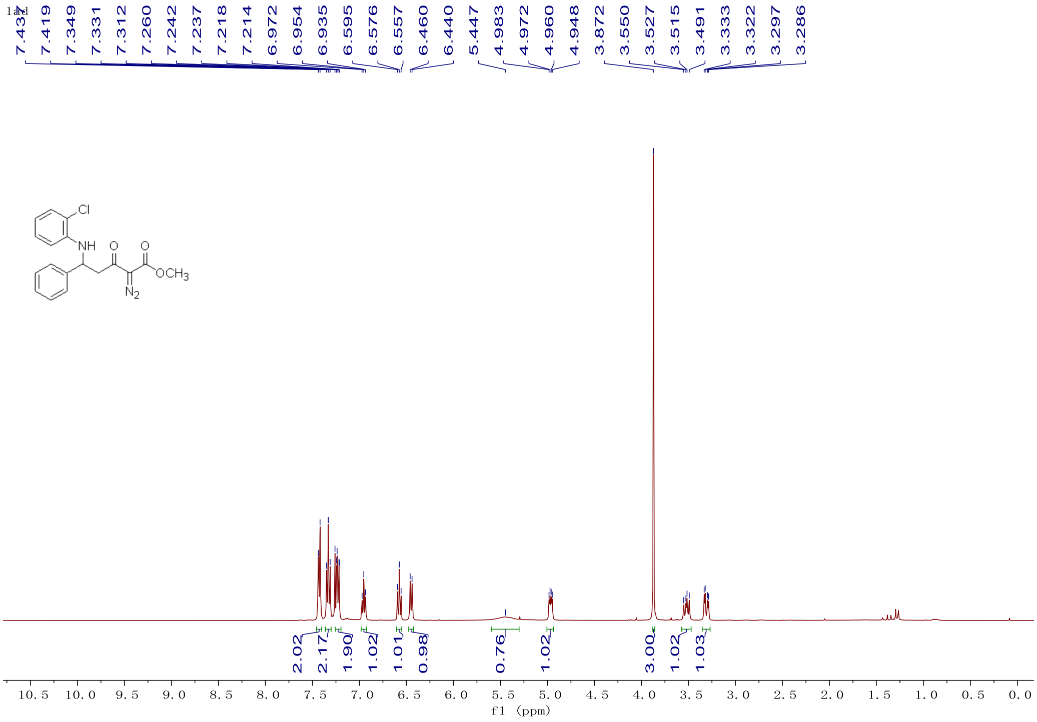

^{1}H NMR spectrum ($CDCl_3$, 400 MHz) of **1ad**

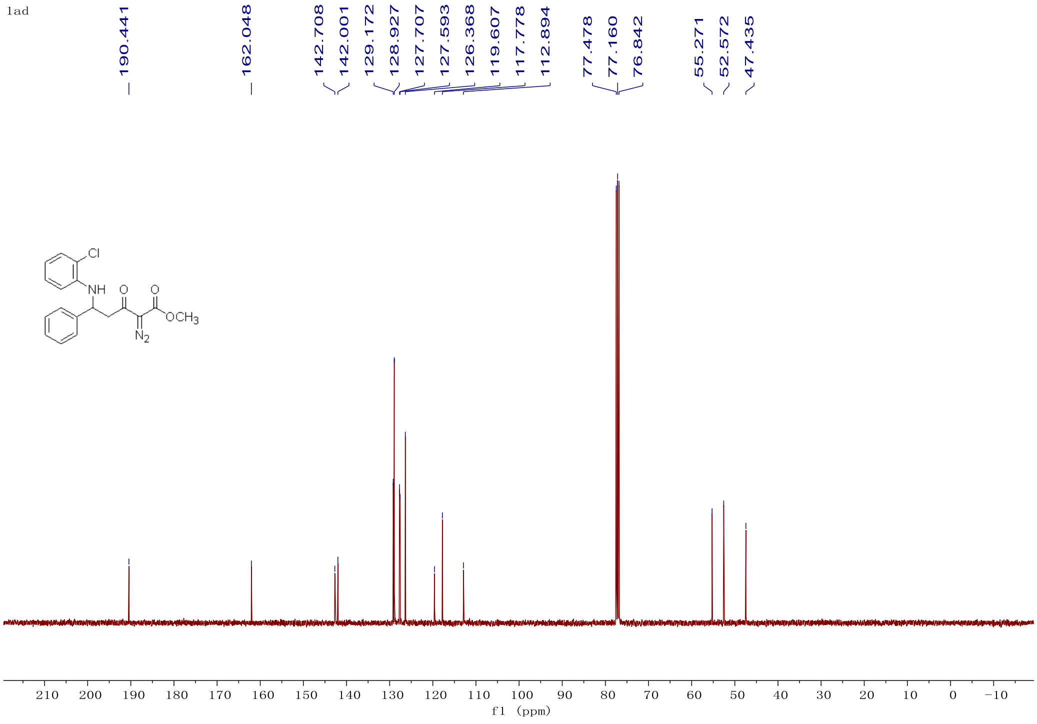

^{13}C NMR spectrum ($CDCl_3$, 100 MHz) of **1ad**

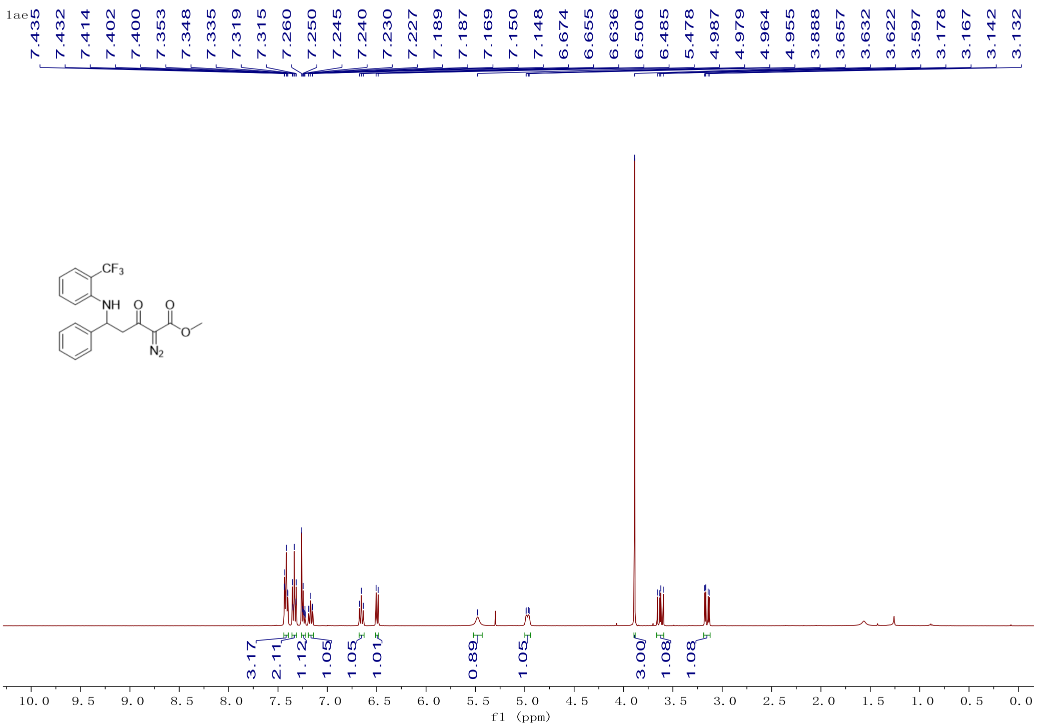

^{1}H NMR spectrum ($CDCl_3$, 400 MHz) of **1ae**

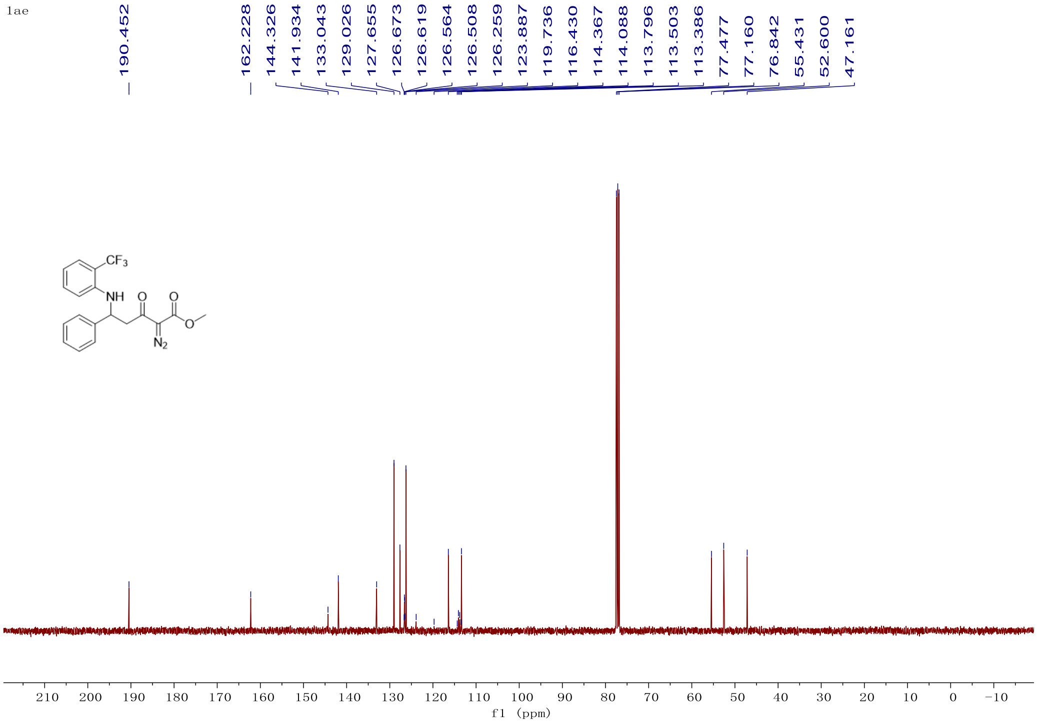

^{13}C NMR spectrum ($CDCl_3$, 100 MHz) of **1ae**

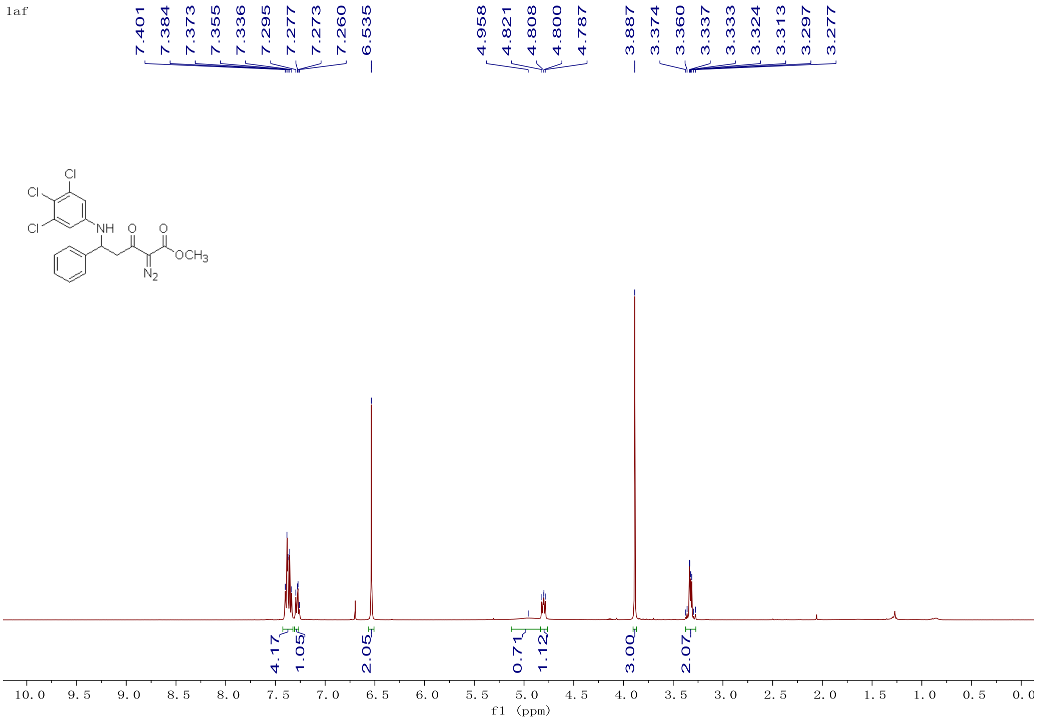

^{1}H NMR spectrum ($CDCl_3$, 400 MHz) of **1af**

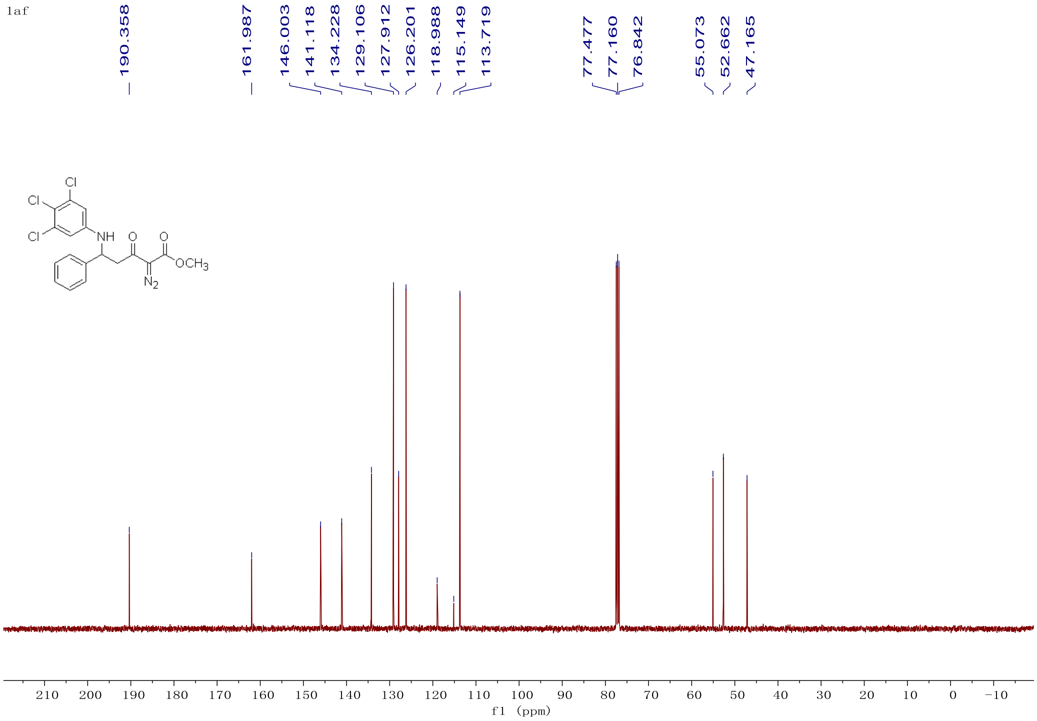

^{13}C NMR spectrum ($CDCl_3$, 100 MHz) of **1af**

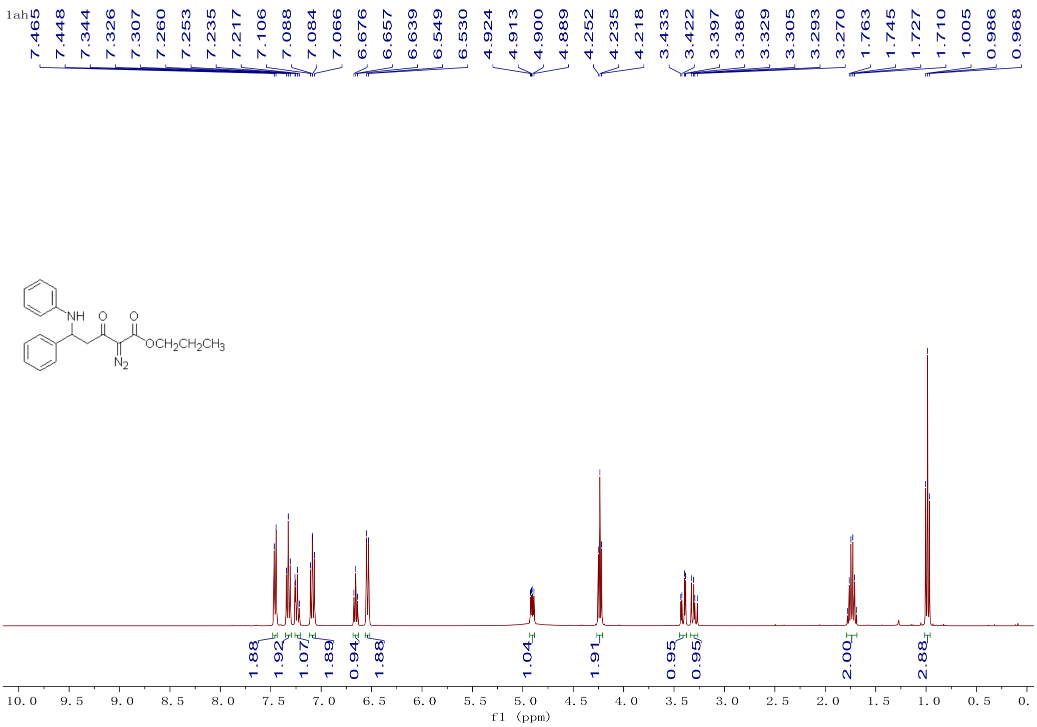

^{1}H NMR spectrum (CDCl$_3$, 400 MHz) of **1ah**

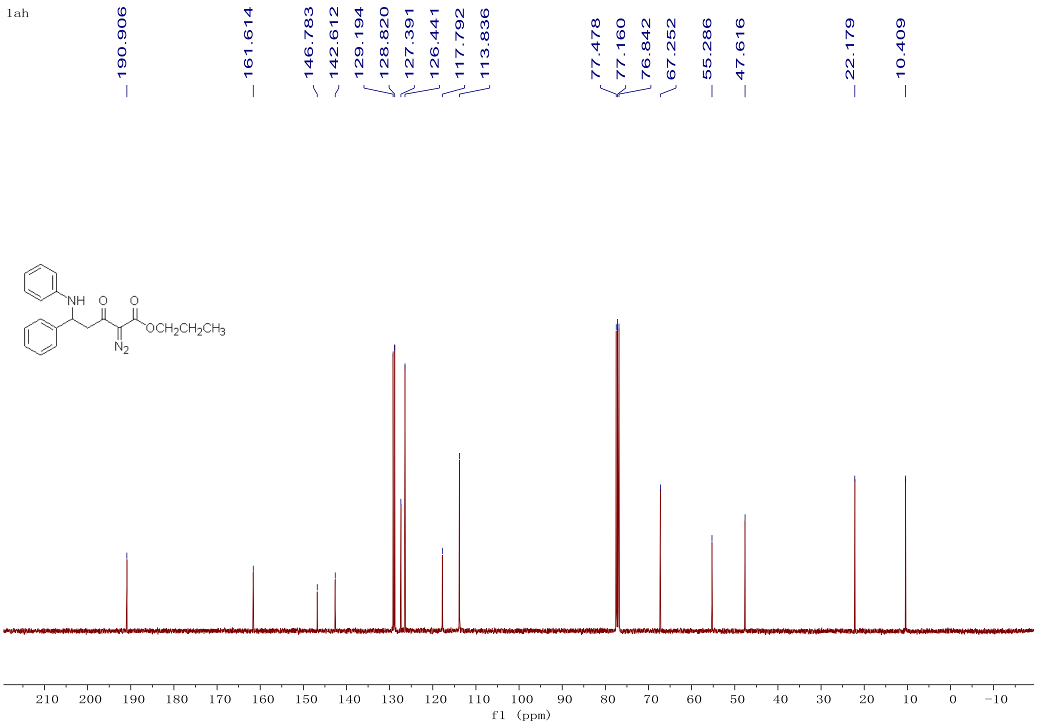

^{13}C NMR spectrum (CDCl$_3$, 100 MHz) of **1ah**

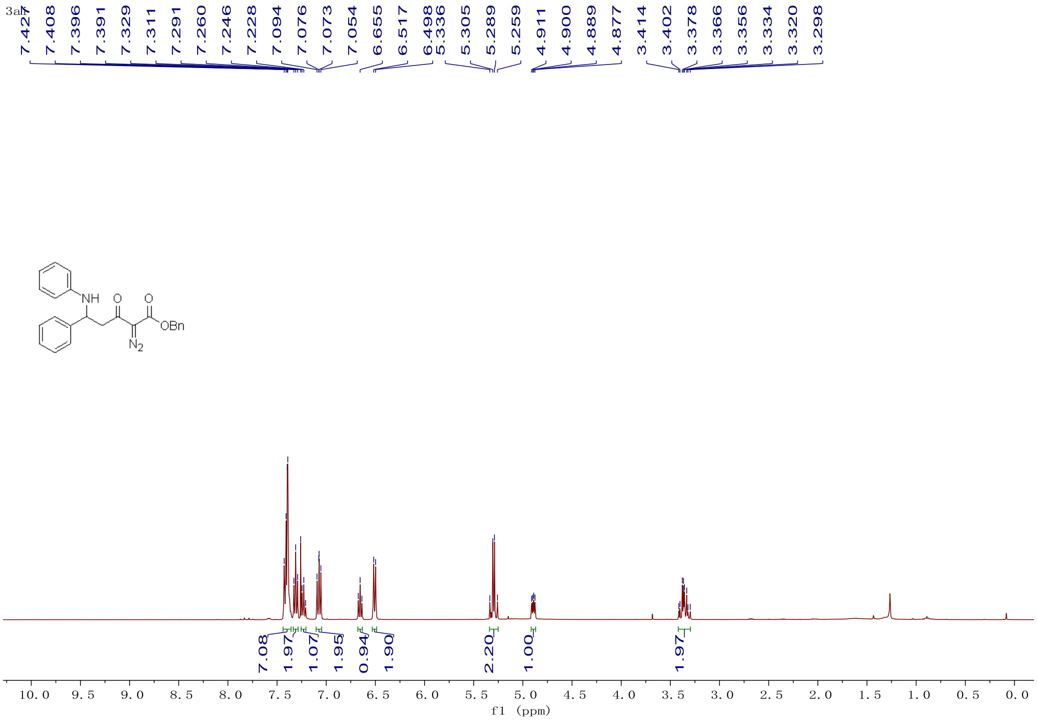

^{1}H NMR spectrum ($CDCl_3$, 400 MHz) of **1ai**

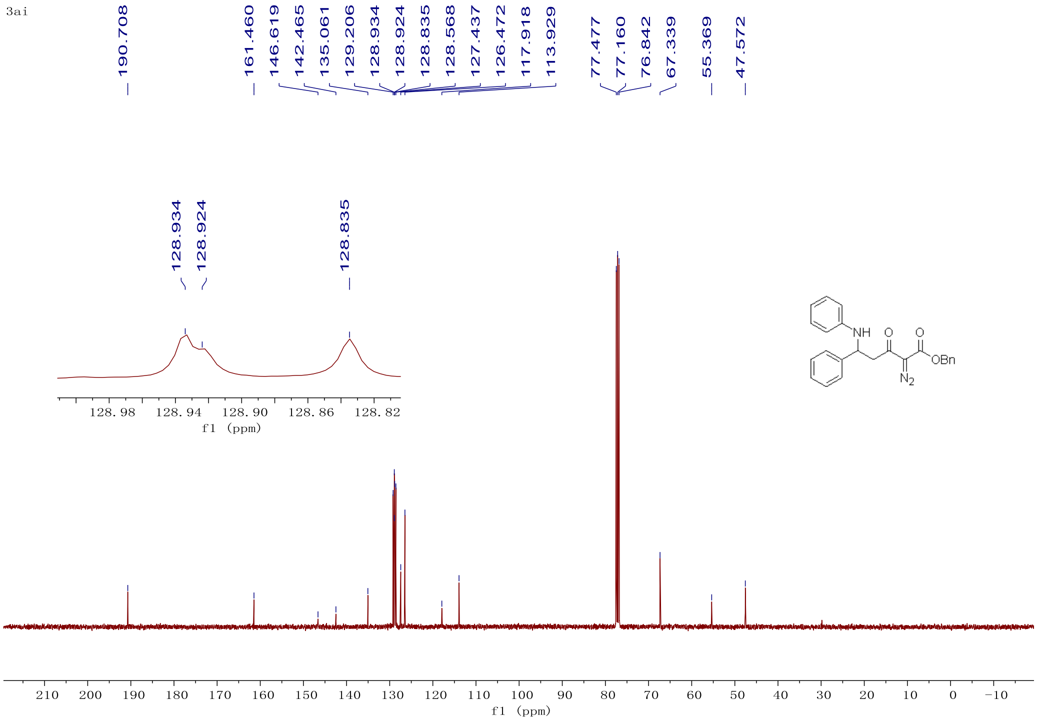

^{13}C NMR spectrum ($CDCl_3$, 100 MHz) of **1ai**

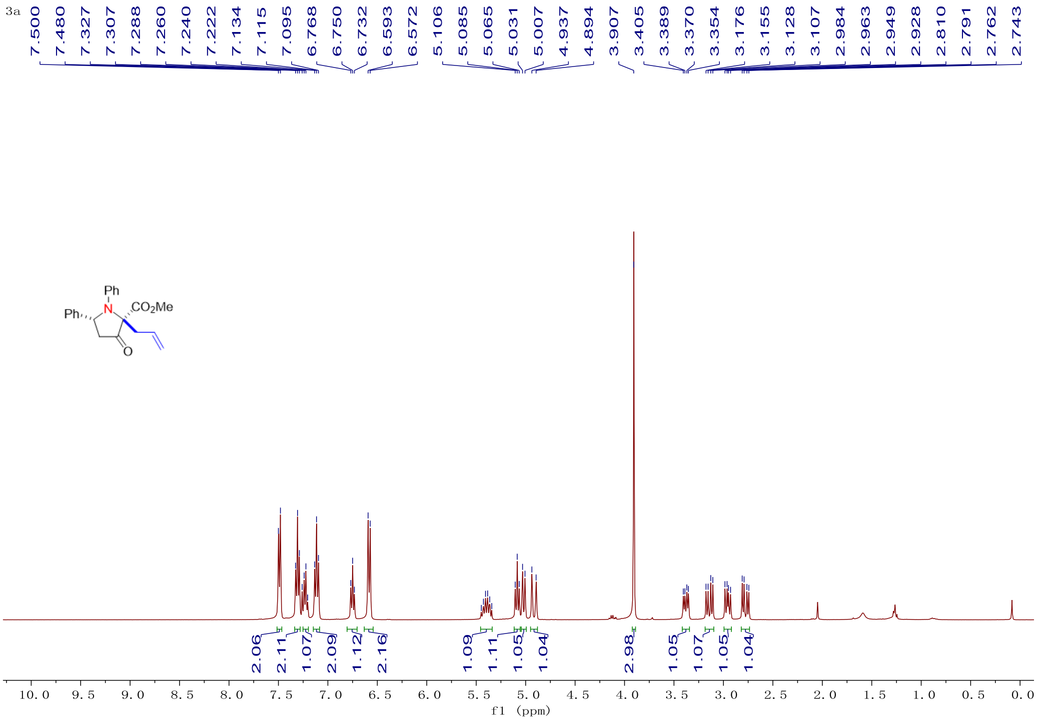

^{1}H NMR spectrum ($CDCl_3$, 400 MHz) of **3a**

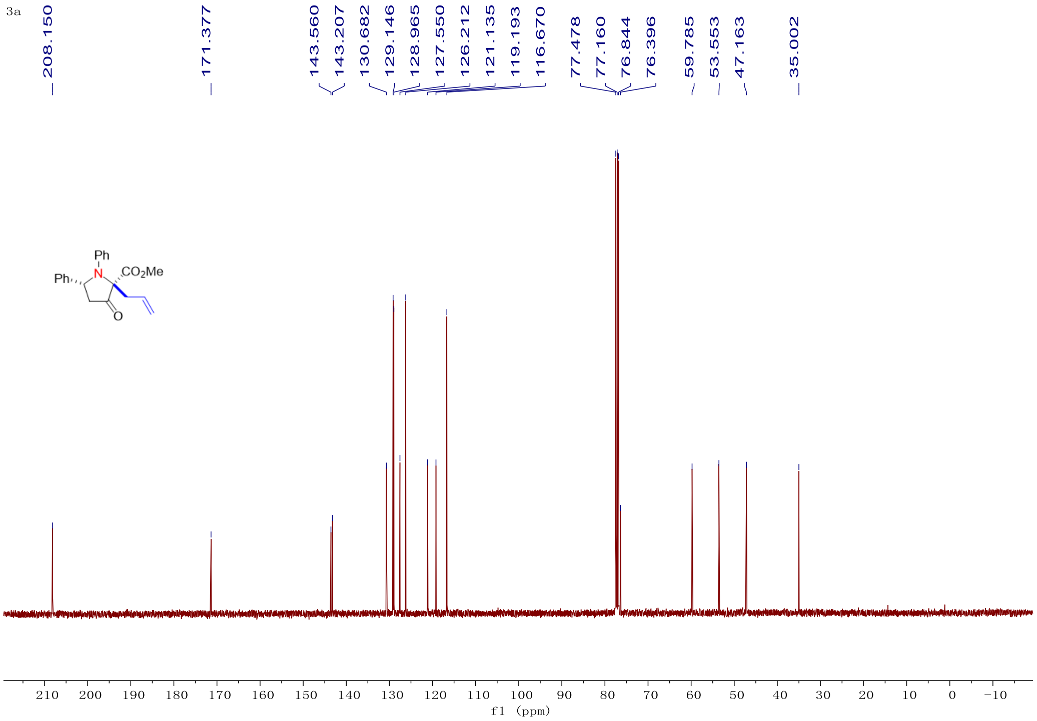

^{13}C NMR spectrum ($CDCl_3$, 100 MHz) of **3a**

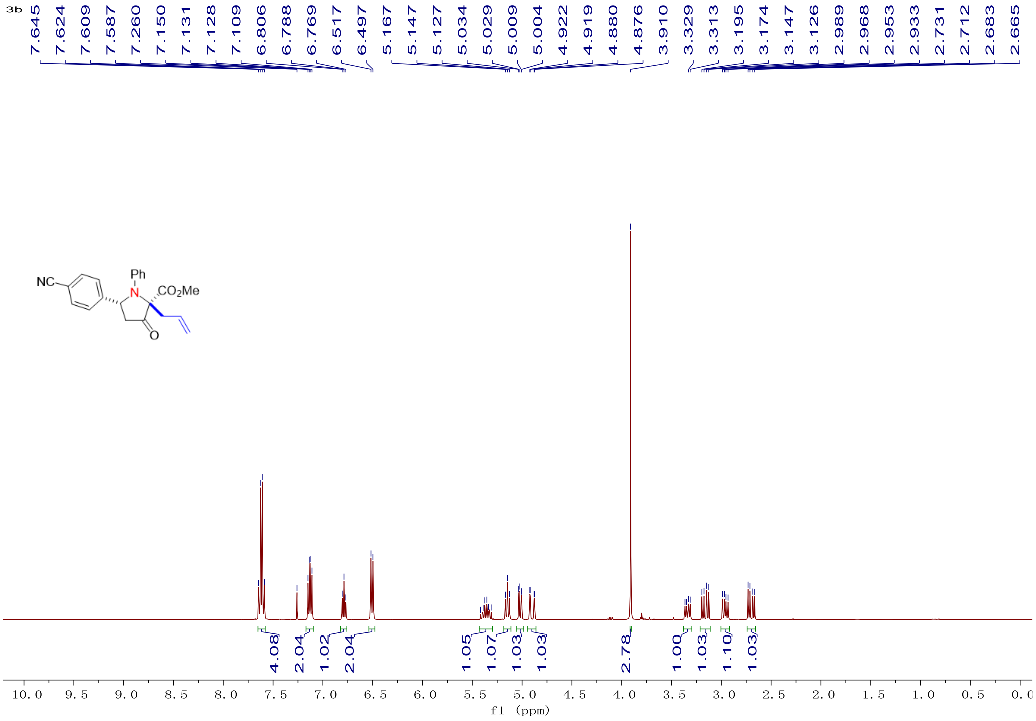

^{1}H NMR spectrum (CDCl$_3$, 400 MHz) of **3b**

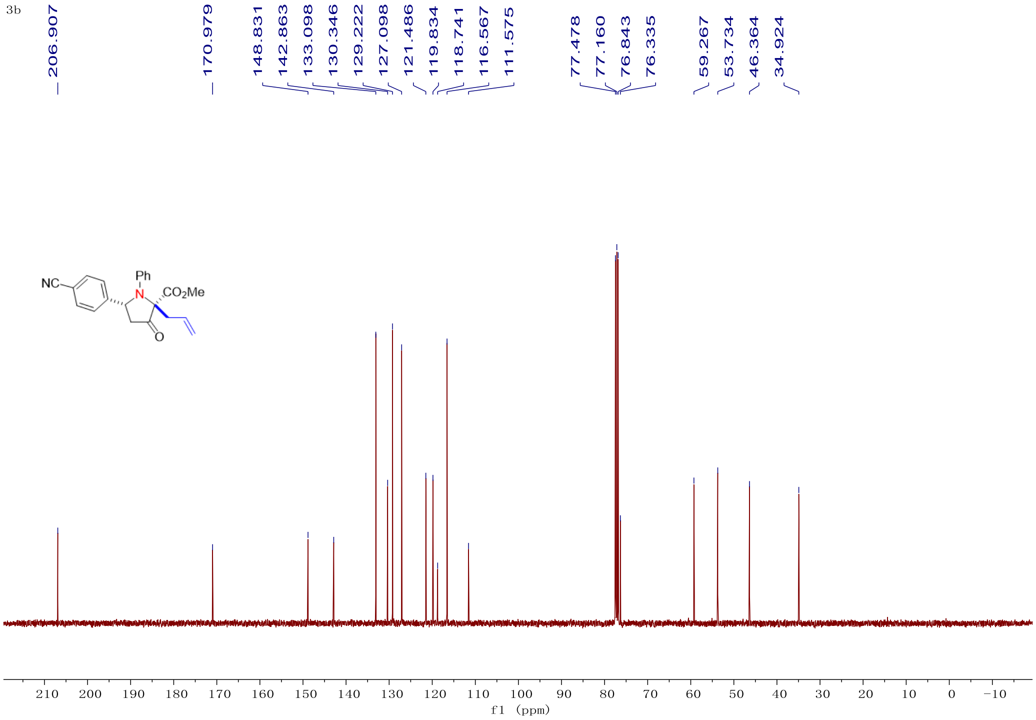

^{13}C NMR spectrum (CDCl$_3$, 100 MHz) of **3b**

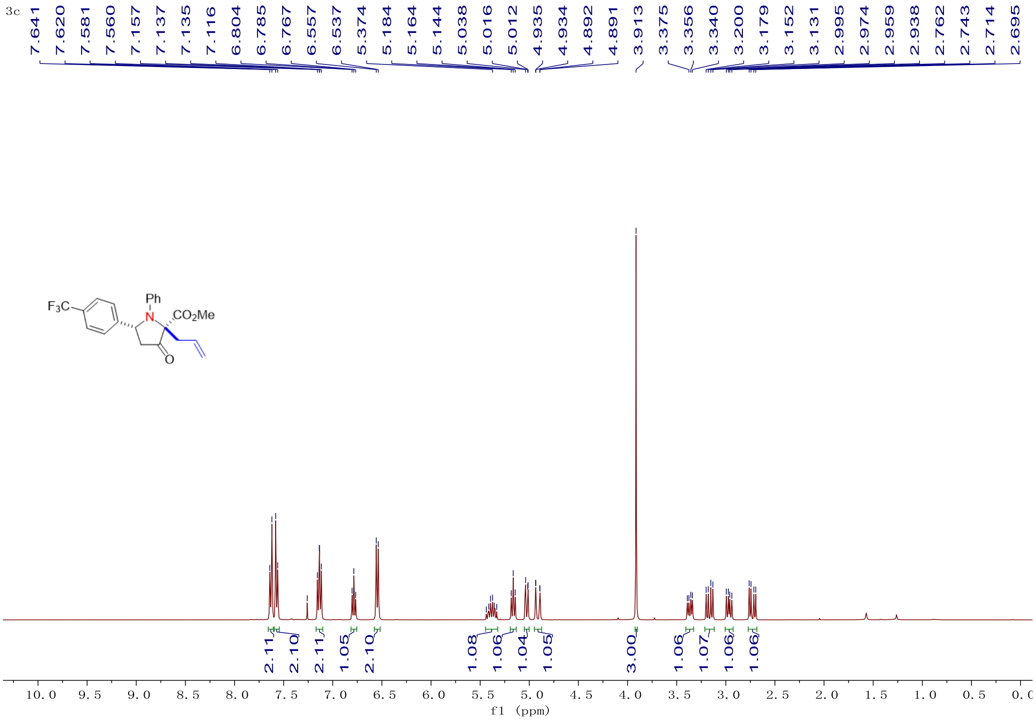

^{1}H NMR spectrum ($CDCl_3$, 400 MHz) of **3c**

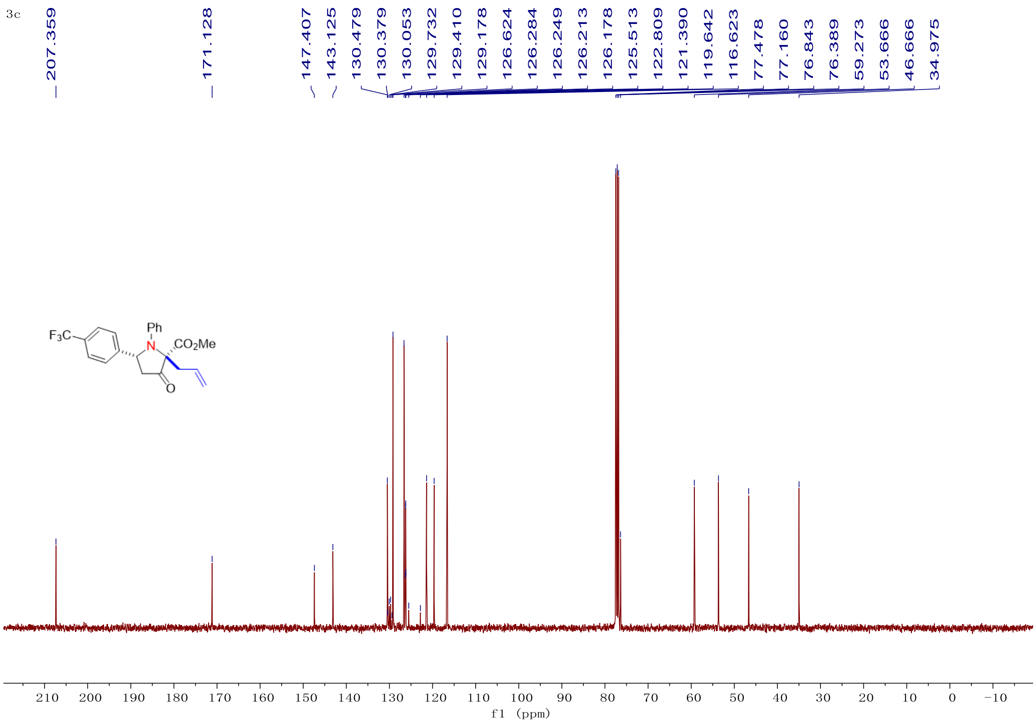

^{13}C NMR spectrum ($CDCl_3$, 100 MHz) of **3c**

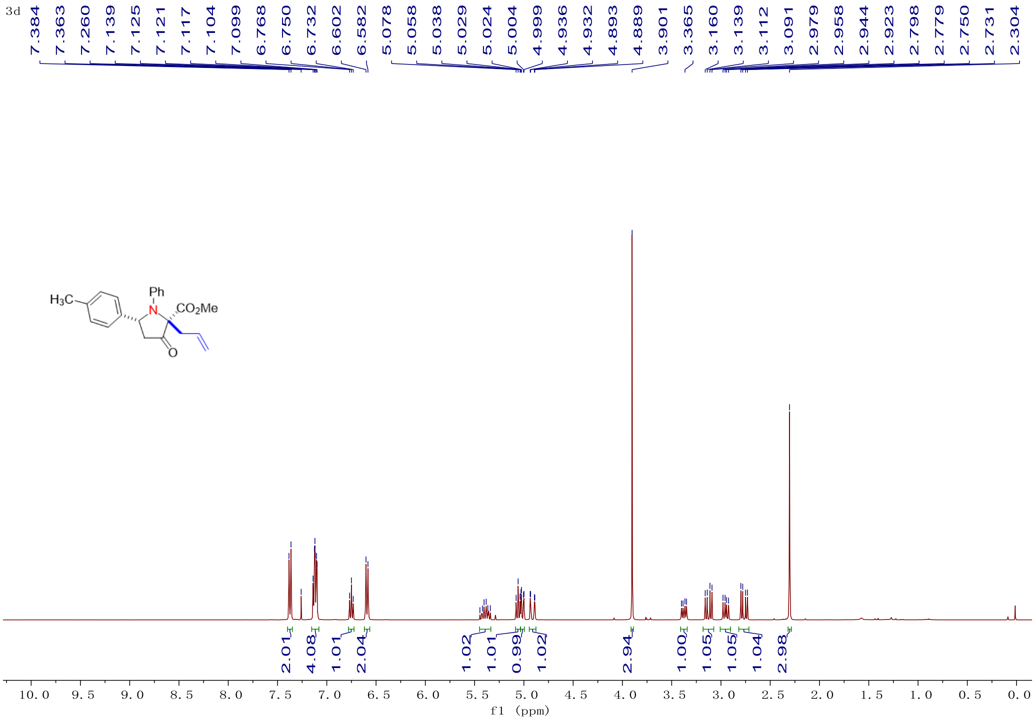

^{1}H NMR spectrum ($CDCl_3$, 400 MHz) of **3d**

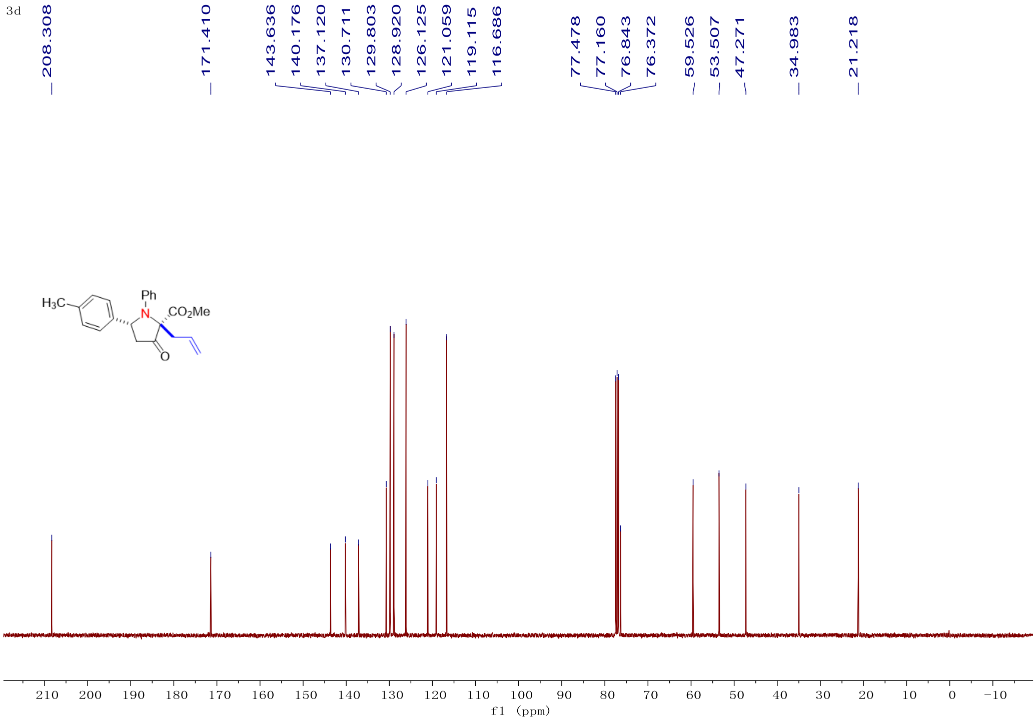

^{13}C NMR spectrum ($CDCl_3$, 100 MHz) of **3d**

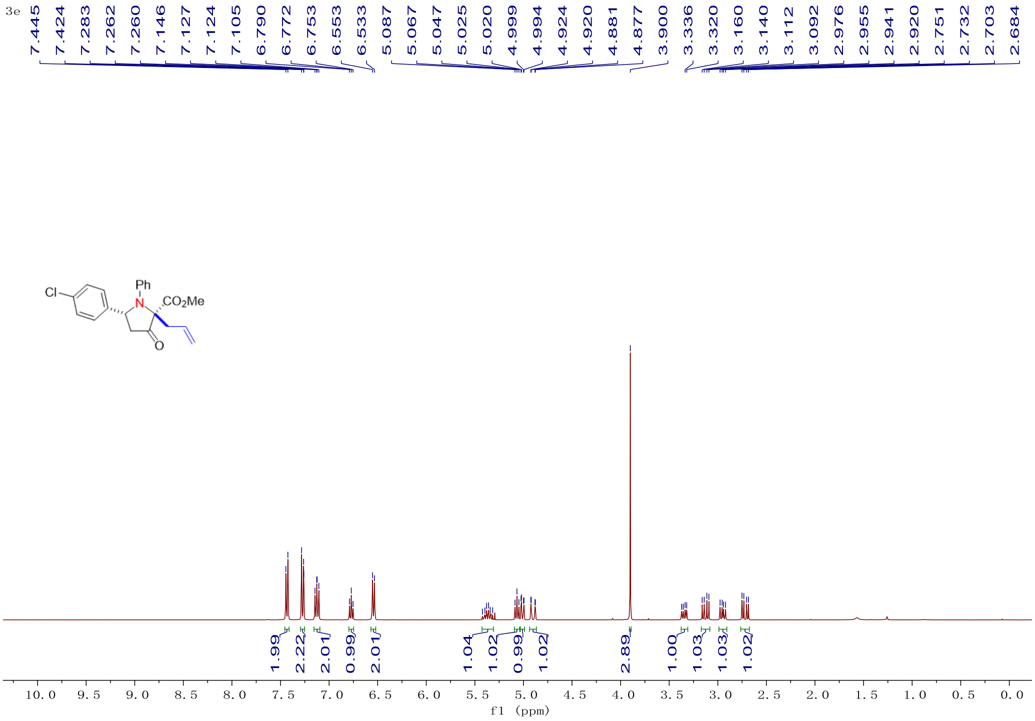

^{1}H NMR spectrum ($CDCl_3$, 400 MHz) of **3e**

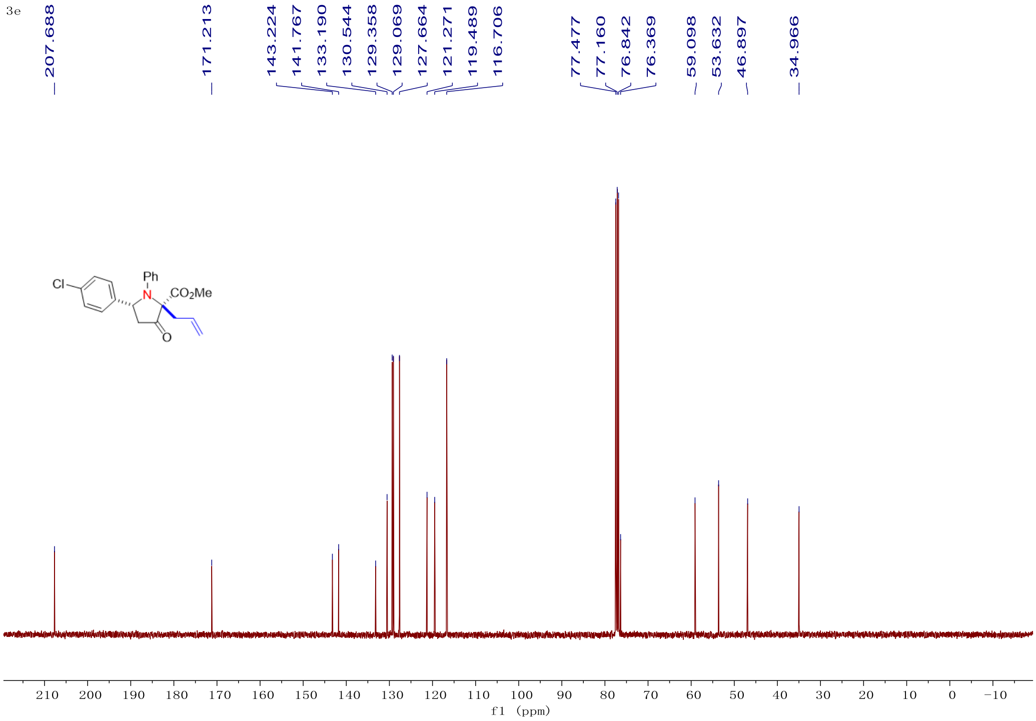

^{13}C NMR spectrum ($CDCl_3$, 100 MHz) of **3e**

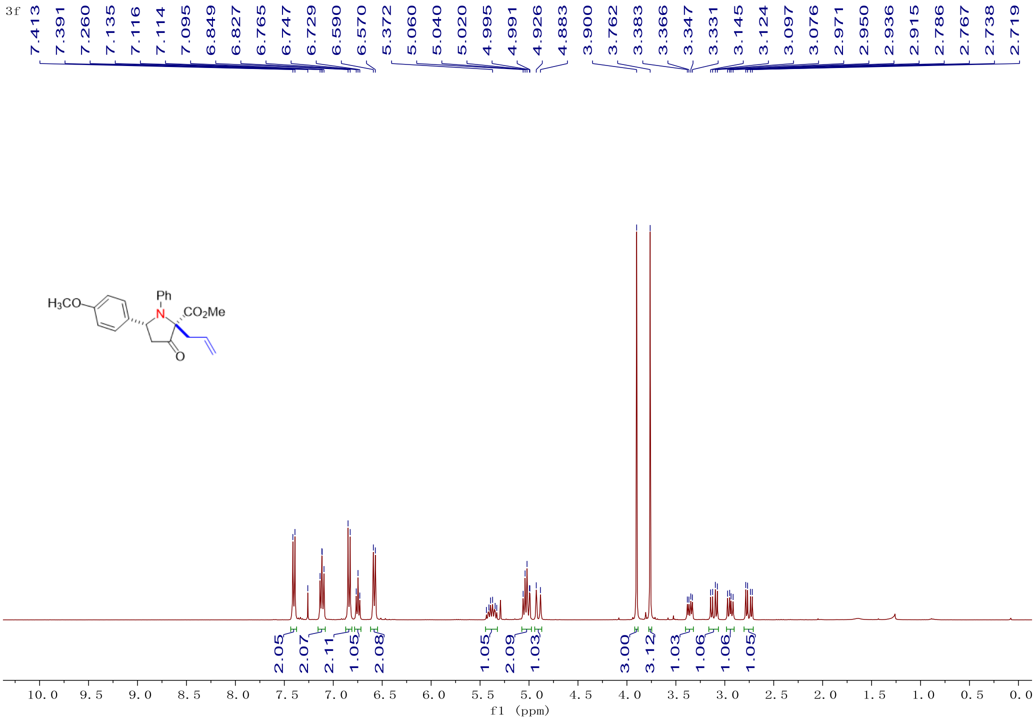

^{1}H NMR spectrum ($CDCl_3$, 400 MHz) of **3f**

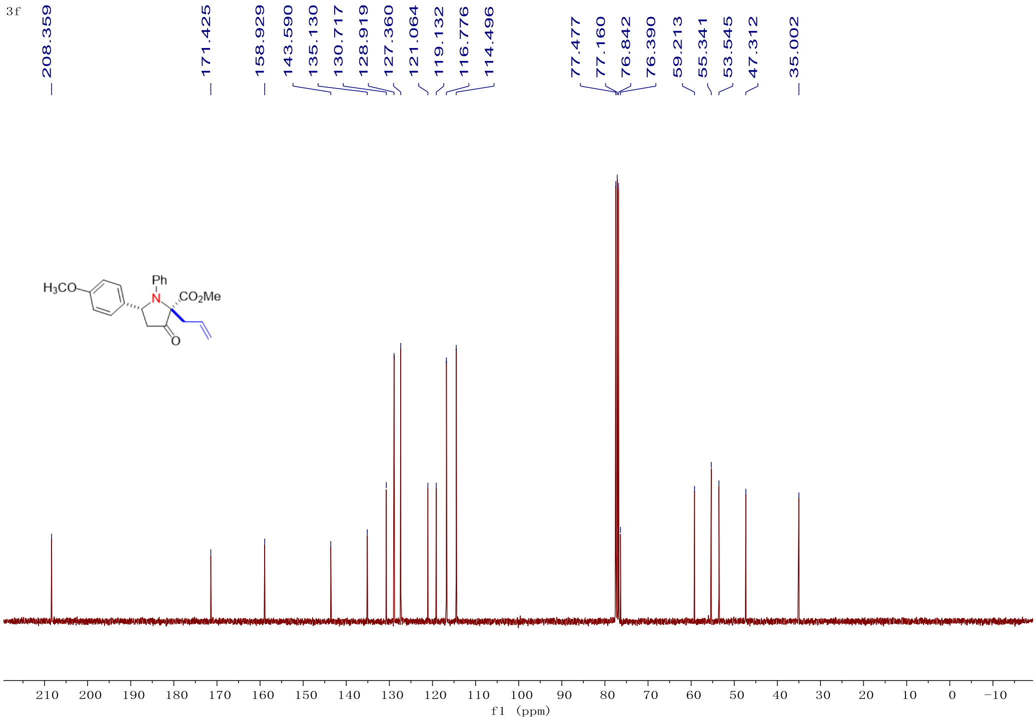

^{13}C NMR spectrum ($CDCl_3$, 100 MHz) of **3f**

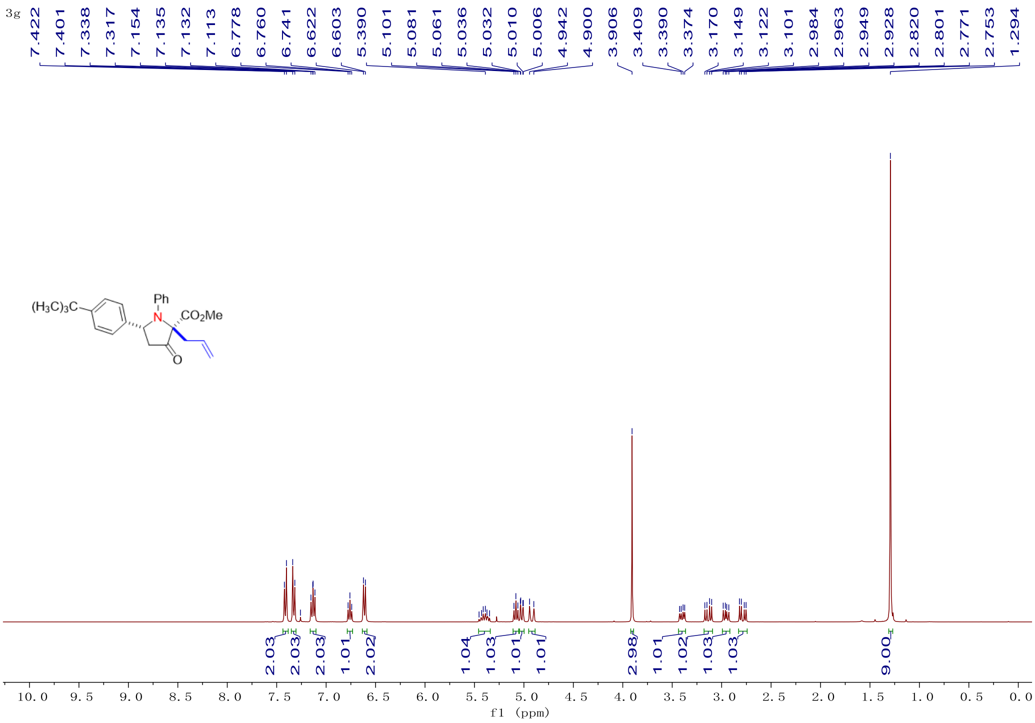

^{1}H NMR spectrum ($CDCl_3$, 400 MHz) of **3g**

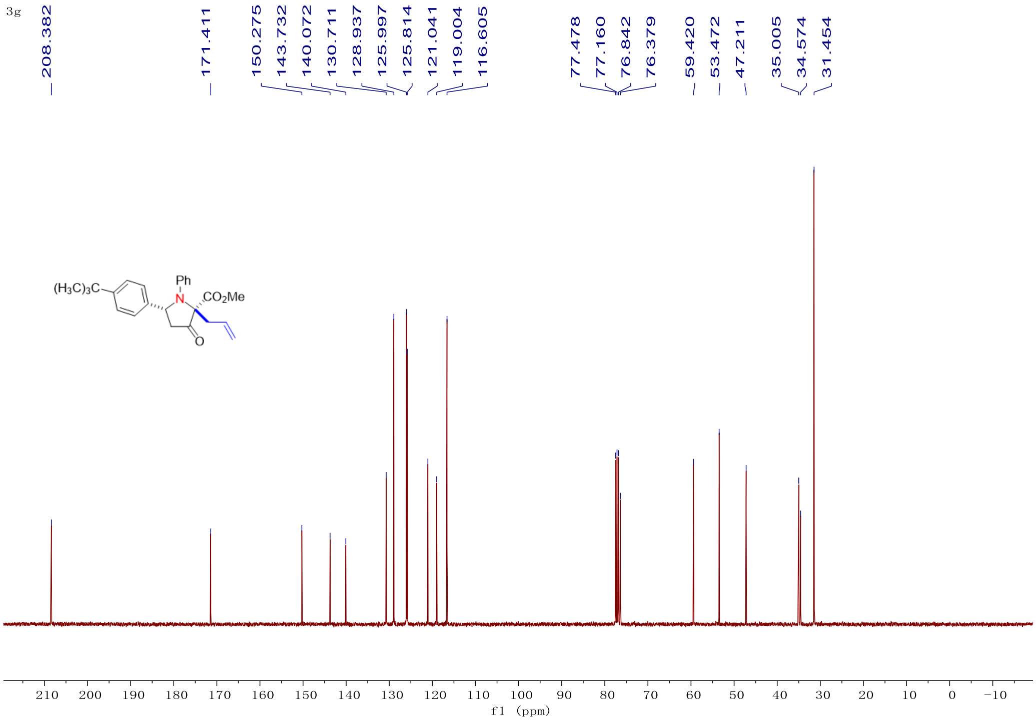

^{13}C NMR spectrum ($CDCl_3$, 100 MHz) of **3g**

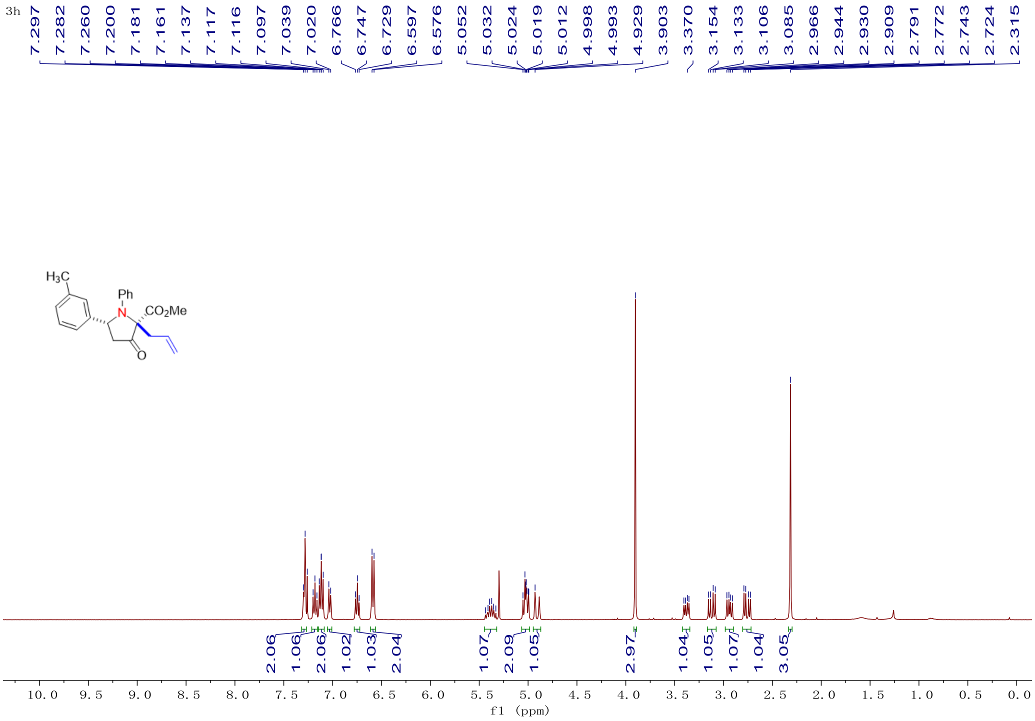

^{1}H NMR spectrum ($CDCl_3$, 400 MHz) of **3h**

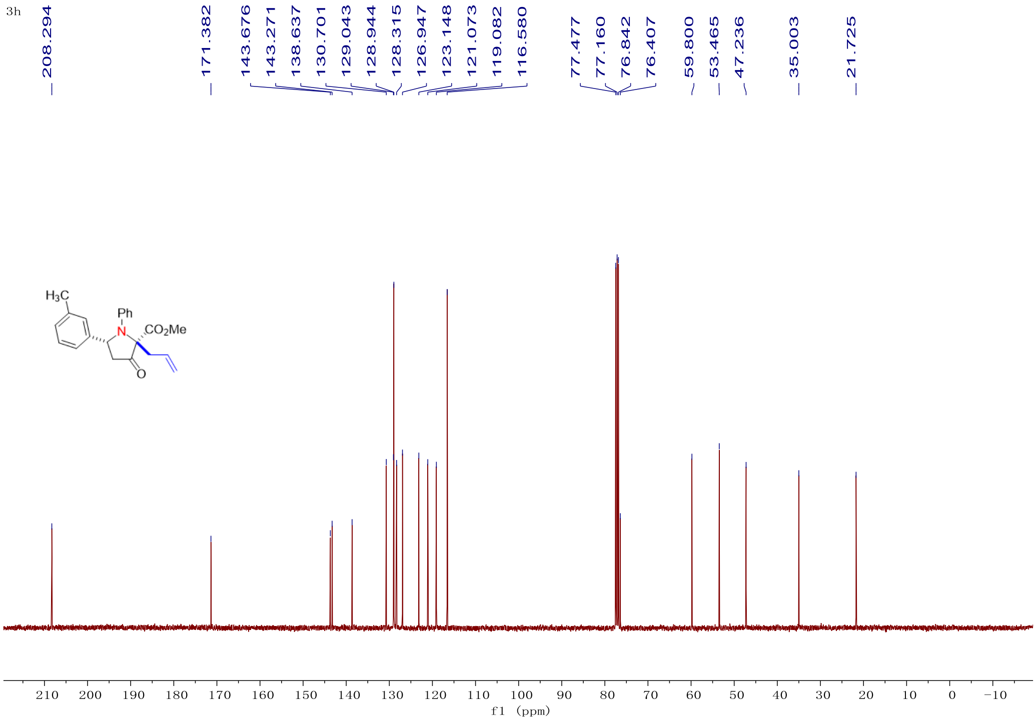

^{13}C NMR spectrum ($CDCl_3$, 100 MHz) of **3h**

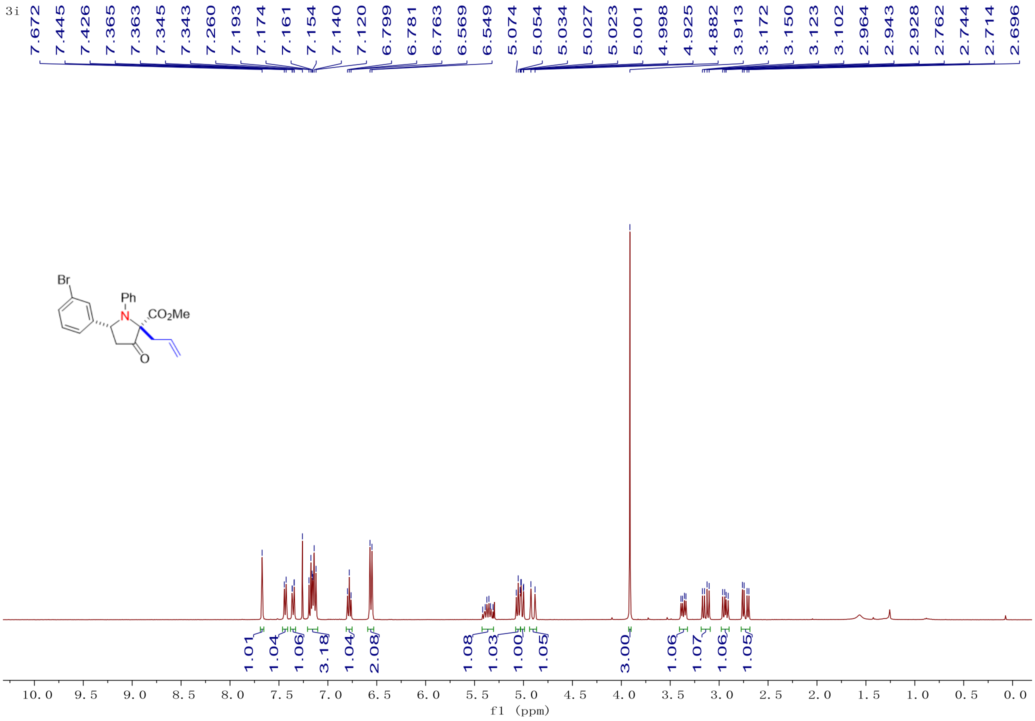

^{1}H NMR spectrum ($CDCl_3$, 400 MHz) of **3i**

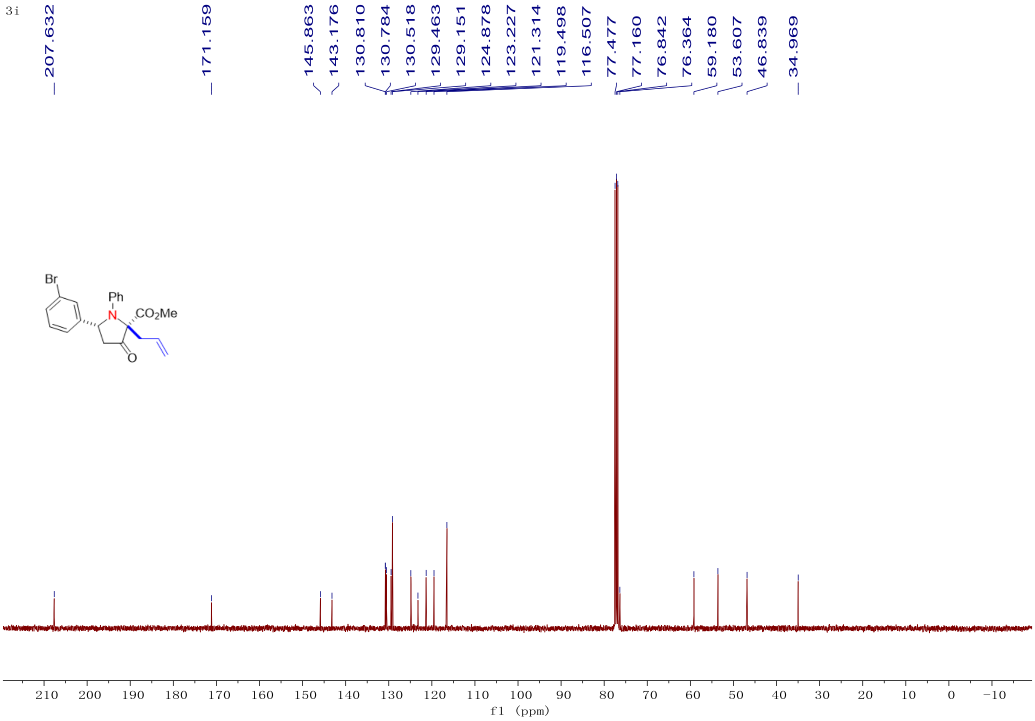

^{13}C NMR spectrum ($CDCl_3$, 100 MHz) of **3i**

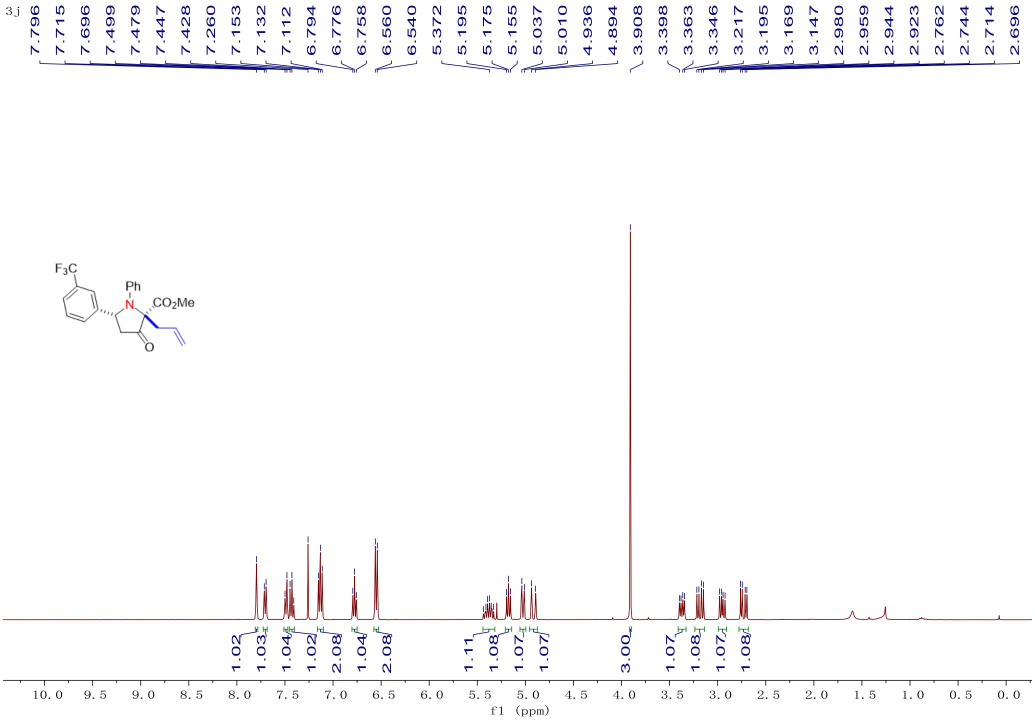

^{1}H NMR spectrum ($CDCl_3$, 400 MHz) of **3j**

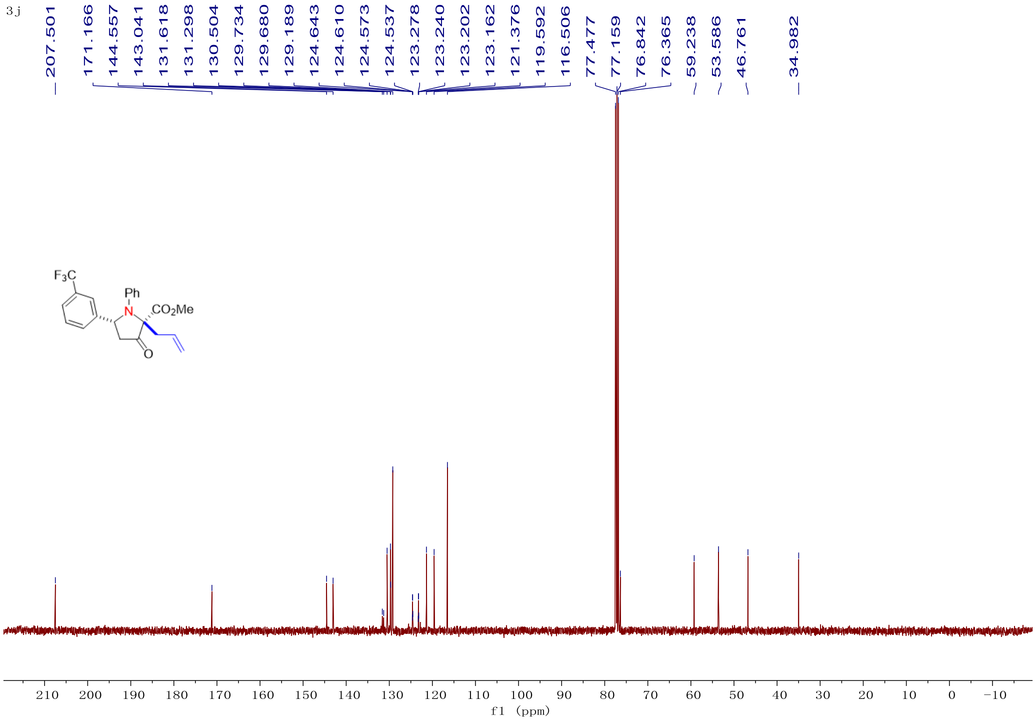

^{13}C NMR spectrum ($CDCl_3$, 100 MHz) of **3j**

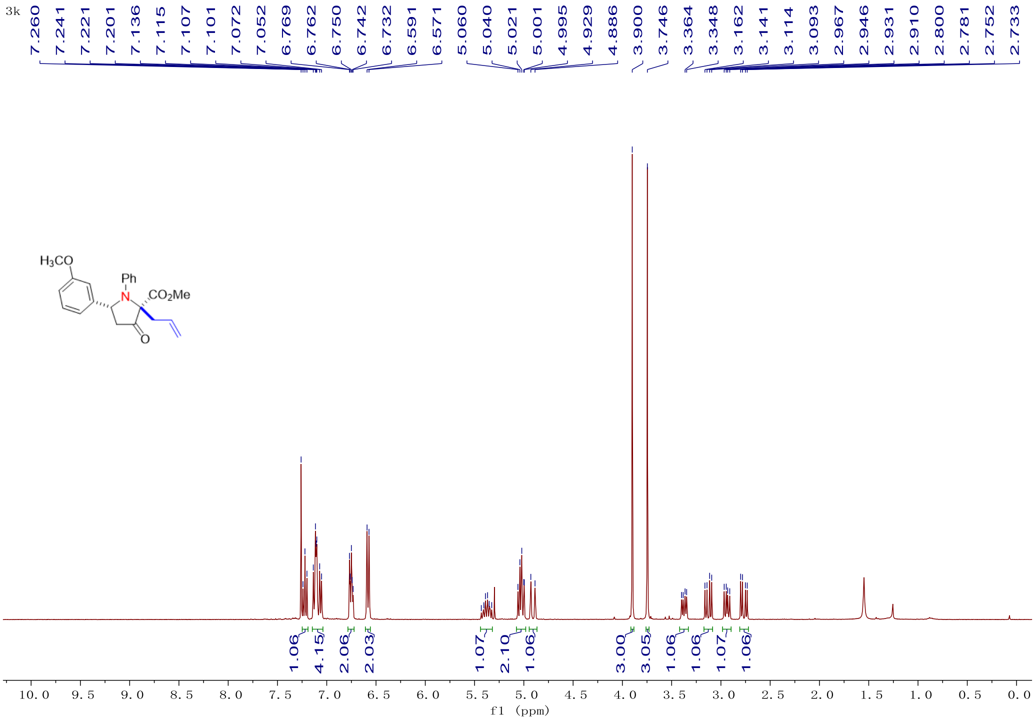

^{1}H NMR spectrum ($CDCl_3$, 400 MHz) of **3k**

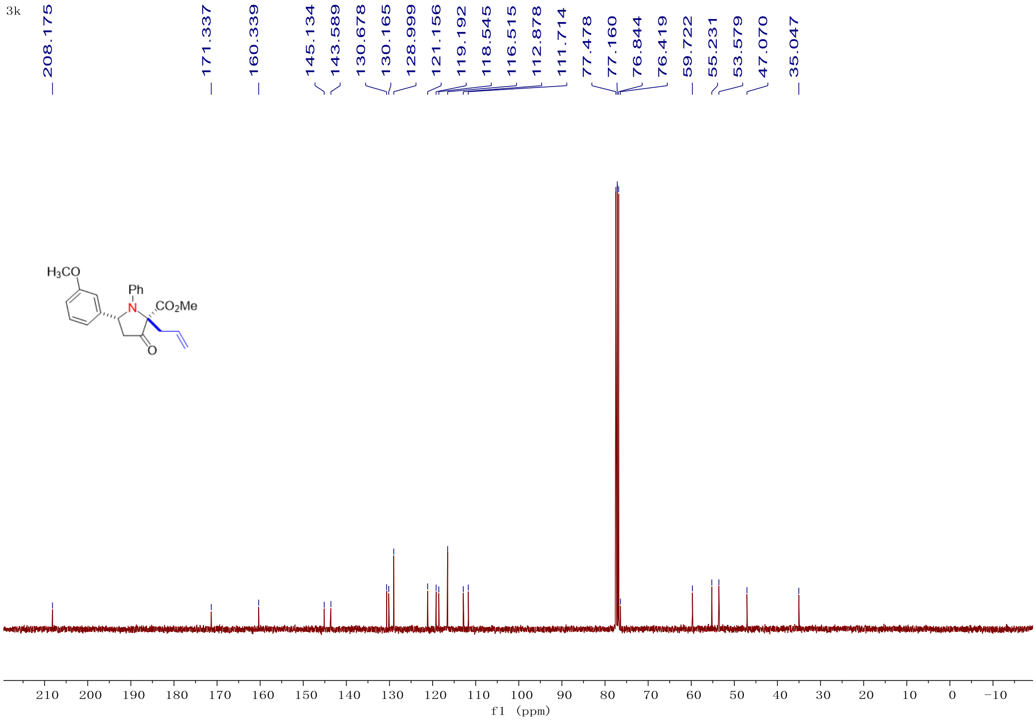

^{13}C NMR spectrum ($CDCl_3$, 100 MHz) of **3k**

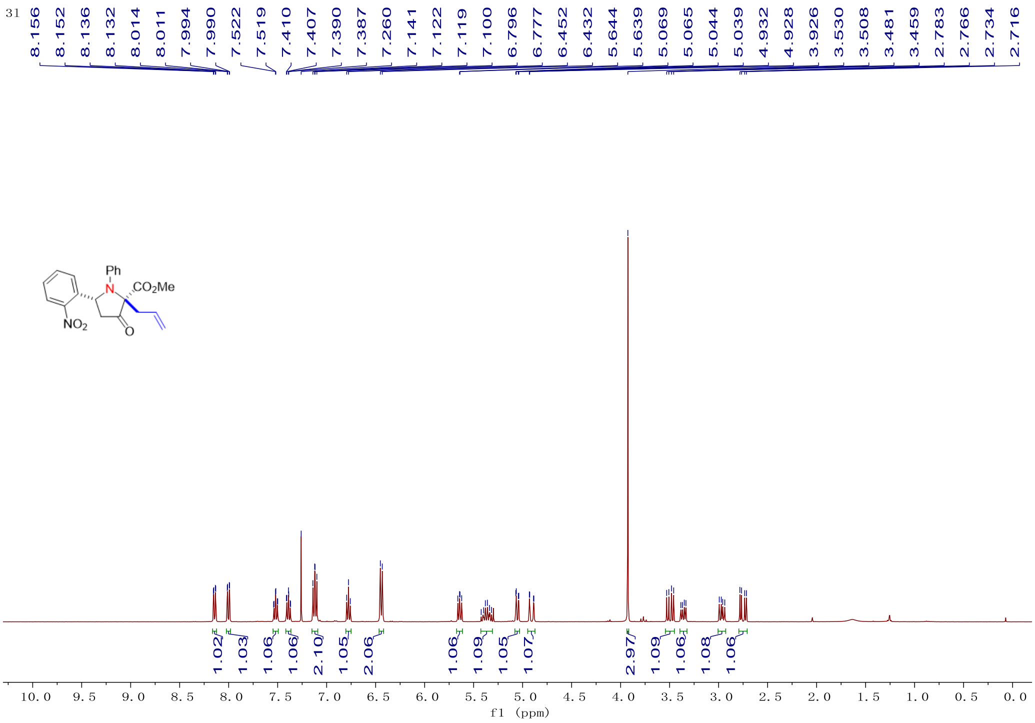

^{1}H NMR spectrum (CDCl$_3$, 400 MHz) of **3l**

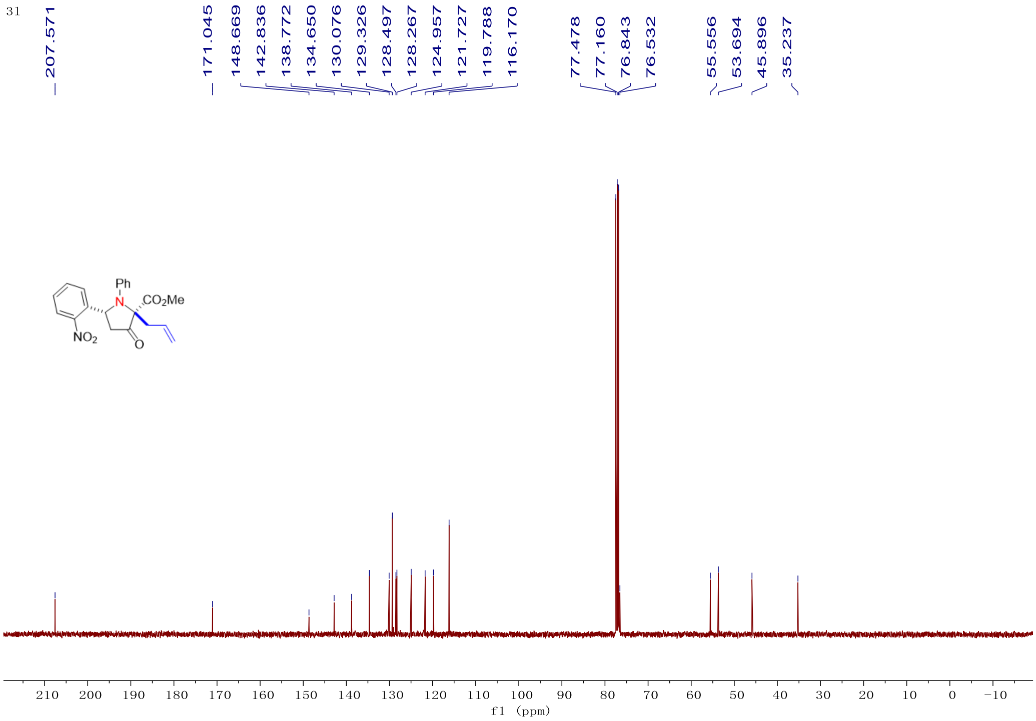

^{13}C NMR spectrum (CDCl$_3$, 100 MHz) of **3l**

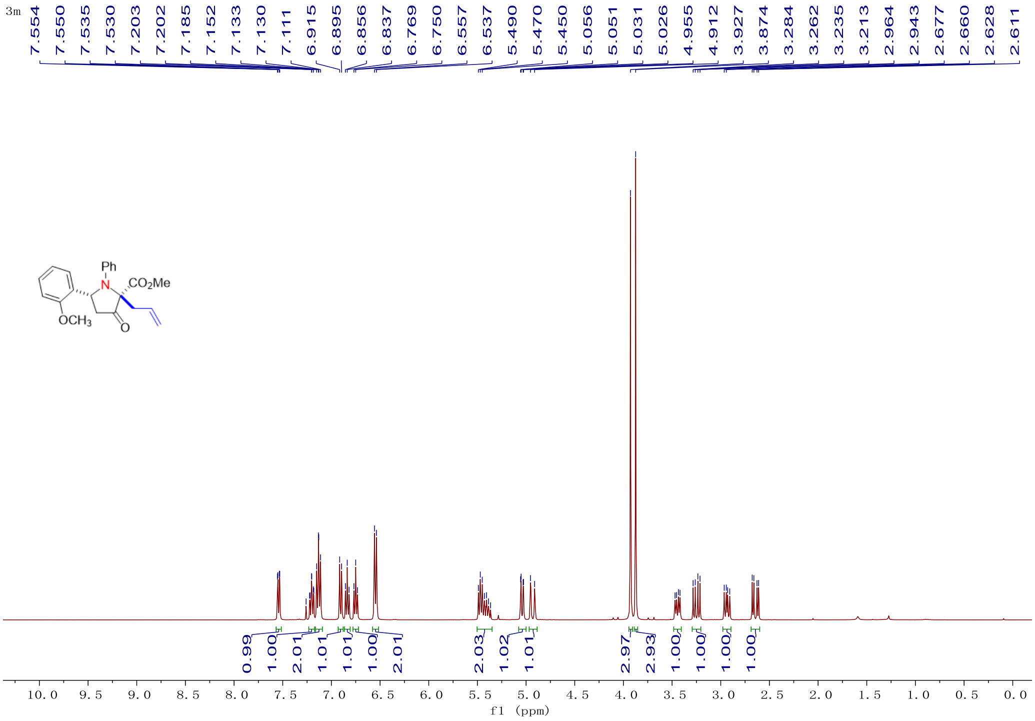

^{1}H NMR spectrum ($CDCl_3$, 400 MHz) of **3m**

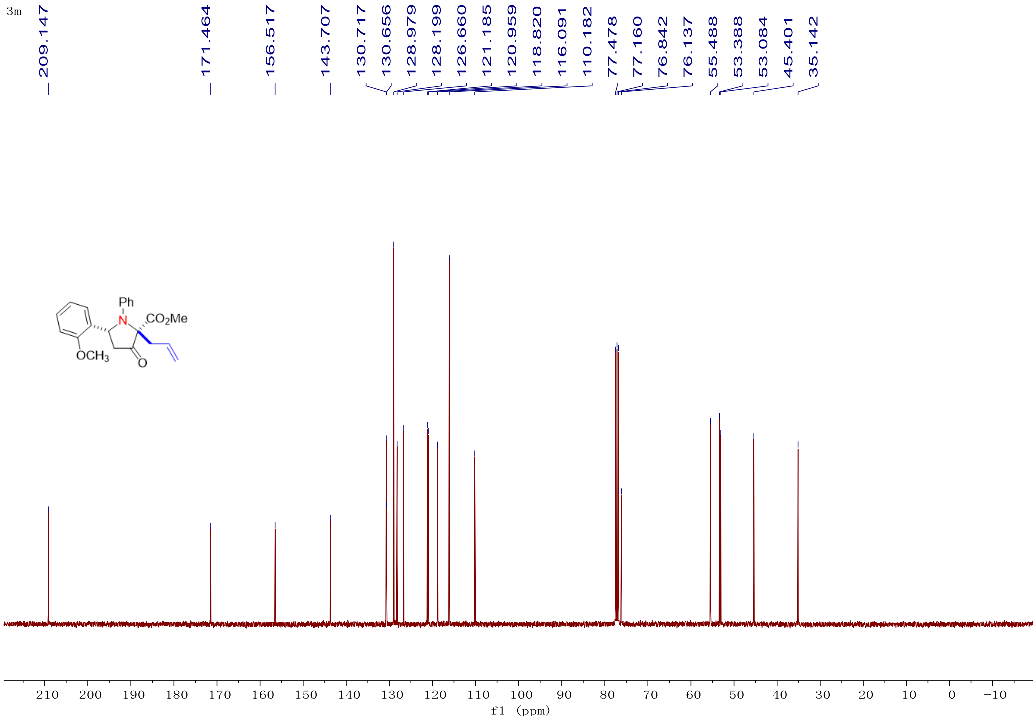

^{13}C NMR spectrum ($CDCl_3$, 100 MHz) of **3m**

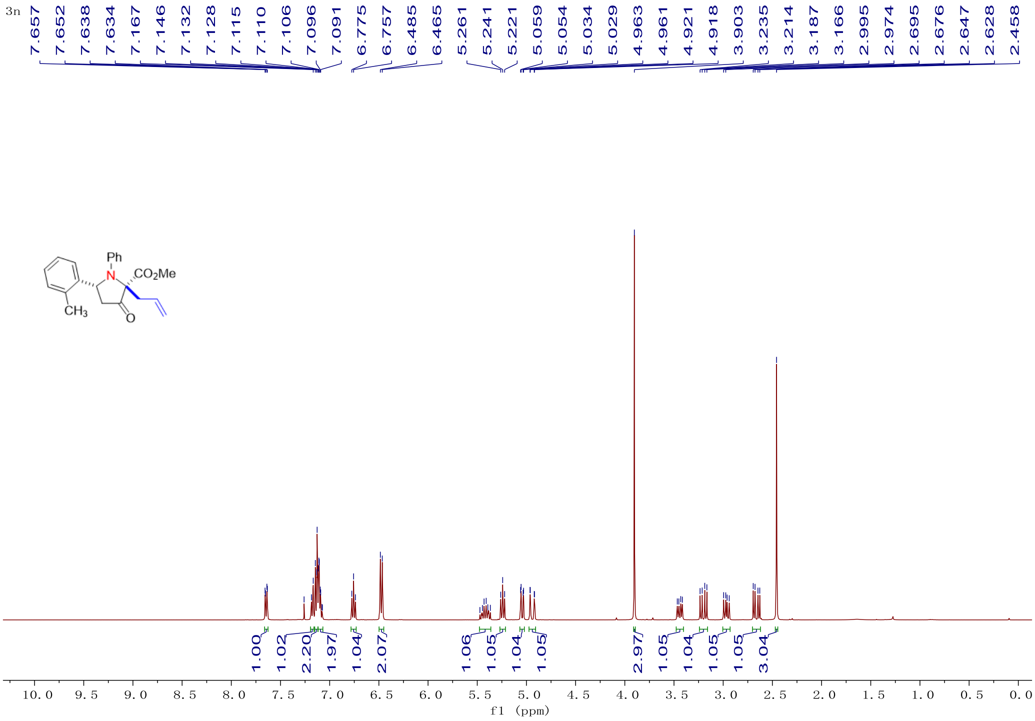

^{1}H NMR spectrum ($CDCl_3$, 400 MHz) of **3n**

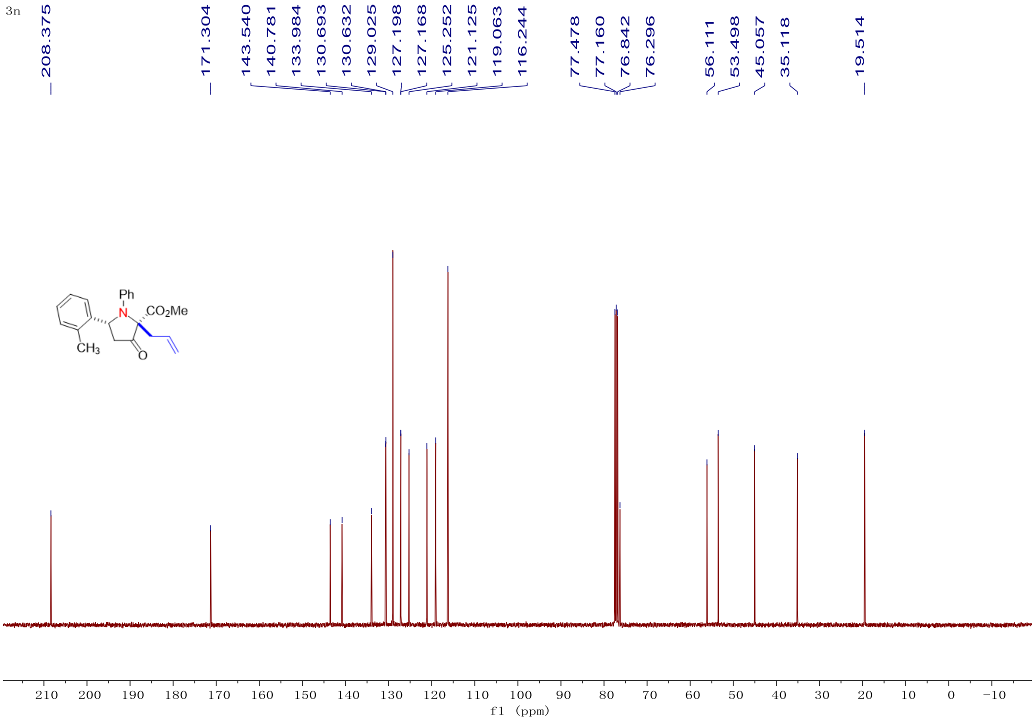

^{13}C NMR spectrum ($CDCl_3$, 100 MHz) of **3n**

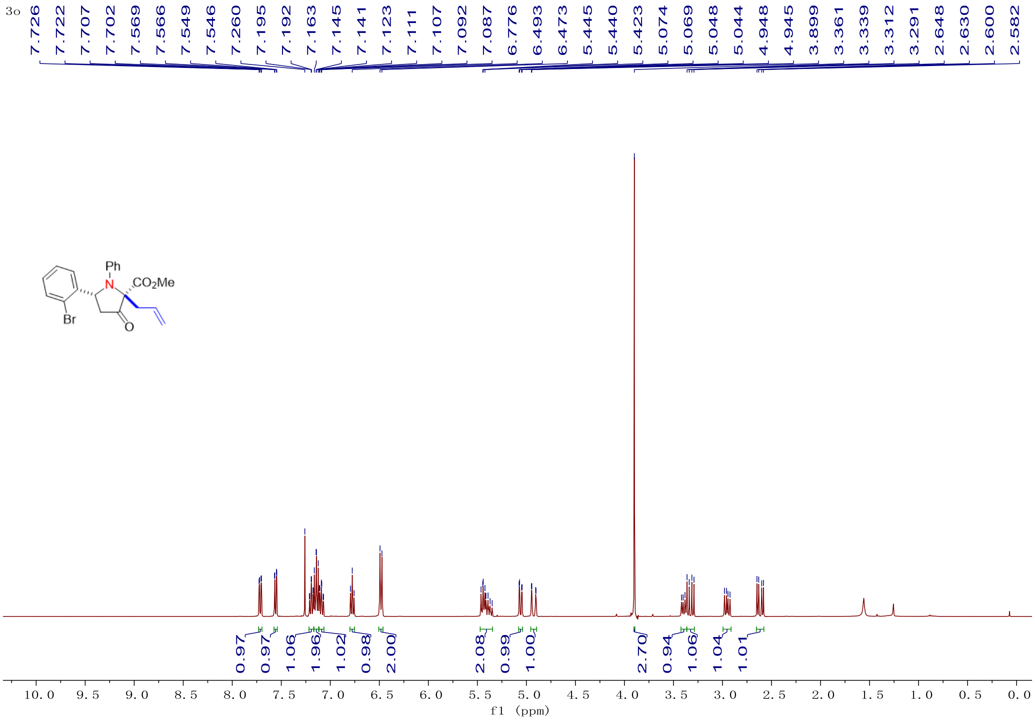

^{1}H NMR spectrum ($CDCl_3$, 400 MHz) of **3o**

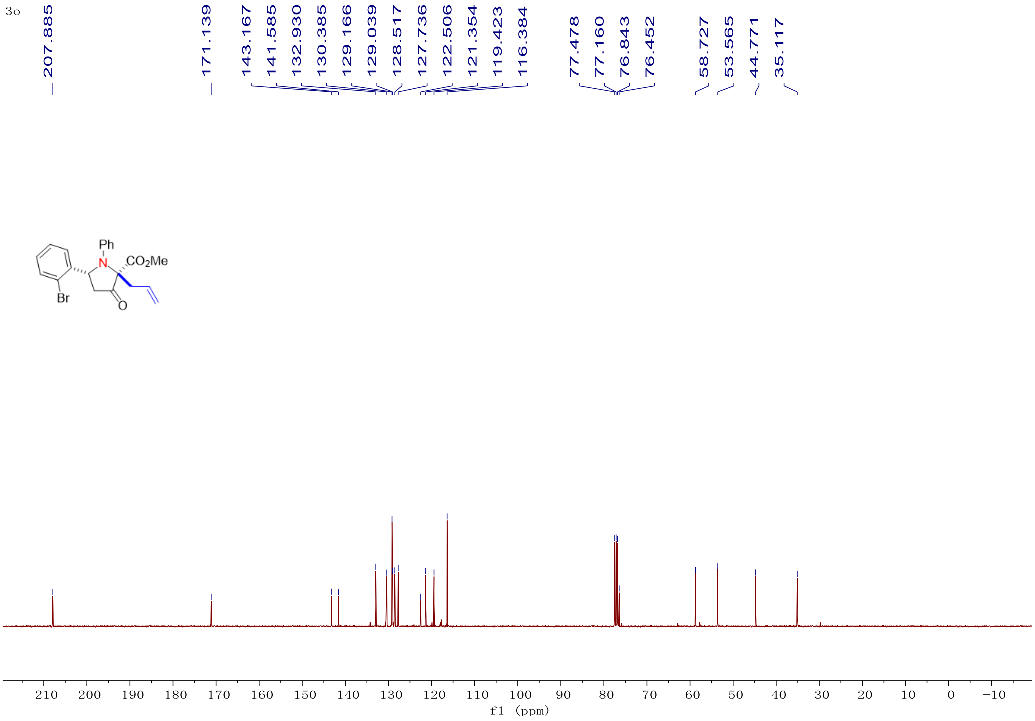

^{13}C NMR spectrum ($CDCl_3$, 100 MHz) of **3o**

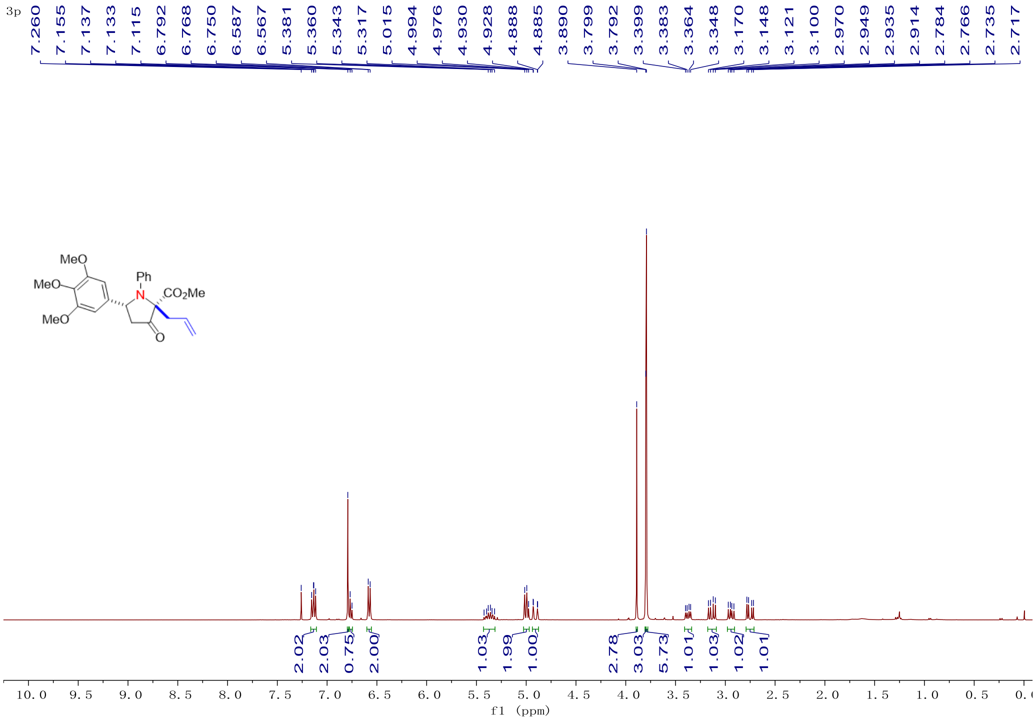

^{1}H NMR spectrum (CDCl$_3$, 400 MHz) of **3p**

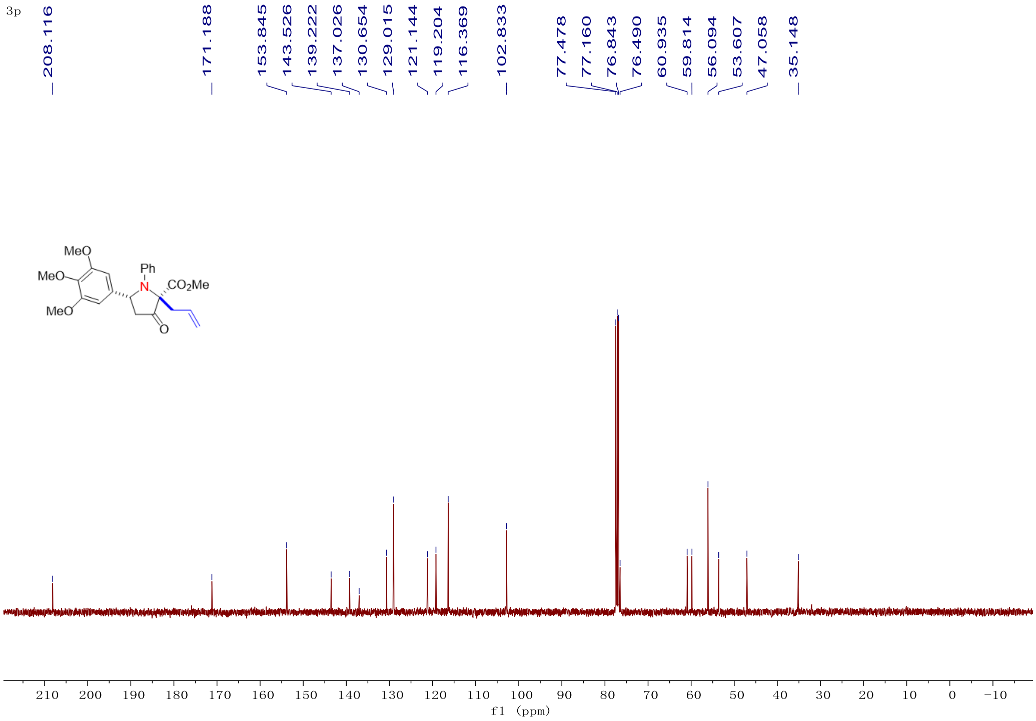

^{13}C NMR spectrum (CDCl$_3$, 100 MHz) of **3p**

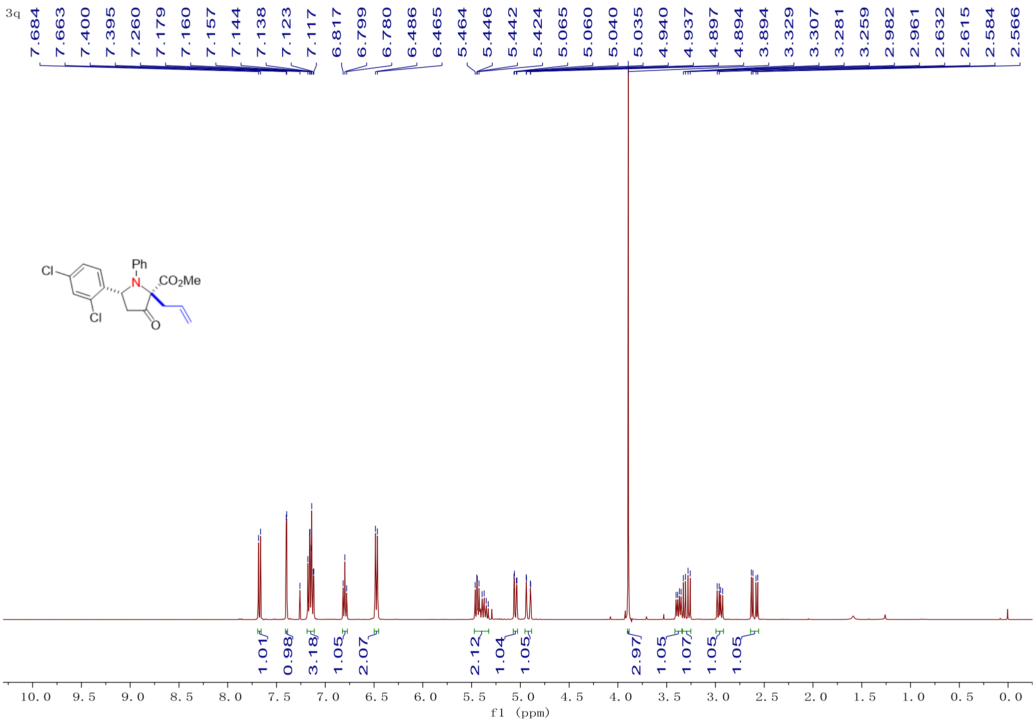

^{1}H NMR spectrum ($CDCl_3$, 400 MHz) of **3q**

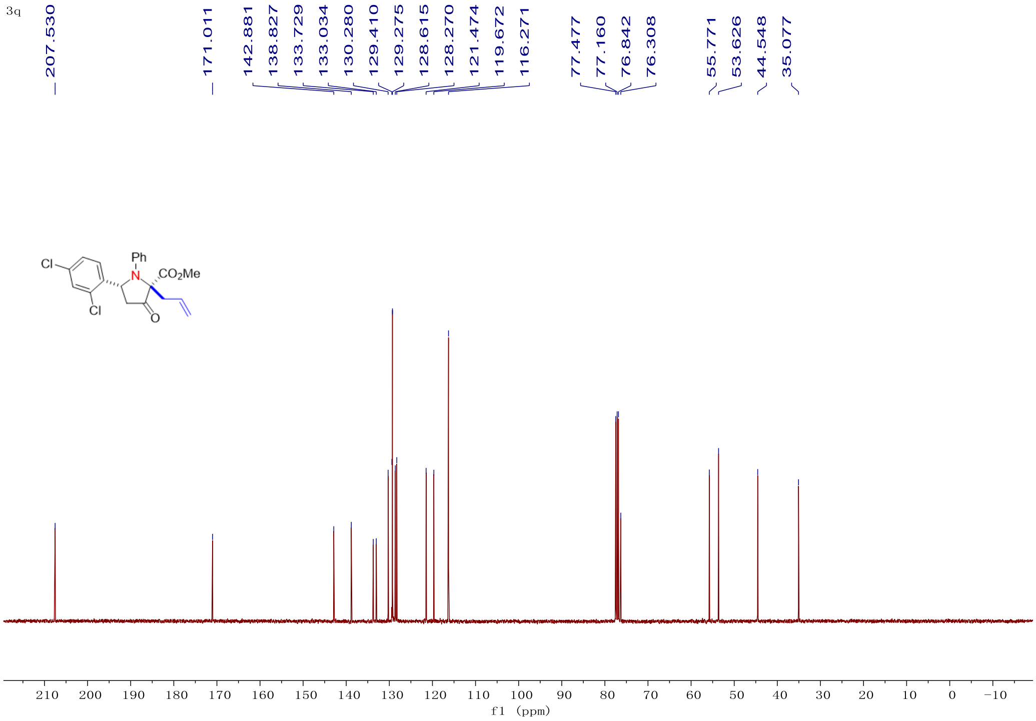

^{13}C NMR spectrum ($CDCl_3$, 100 MHz) of **3q**

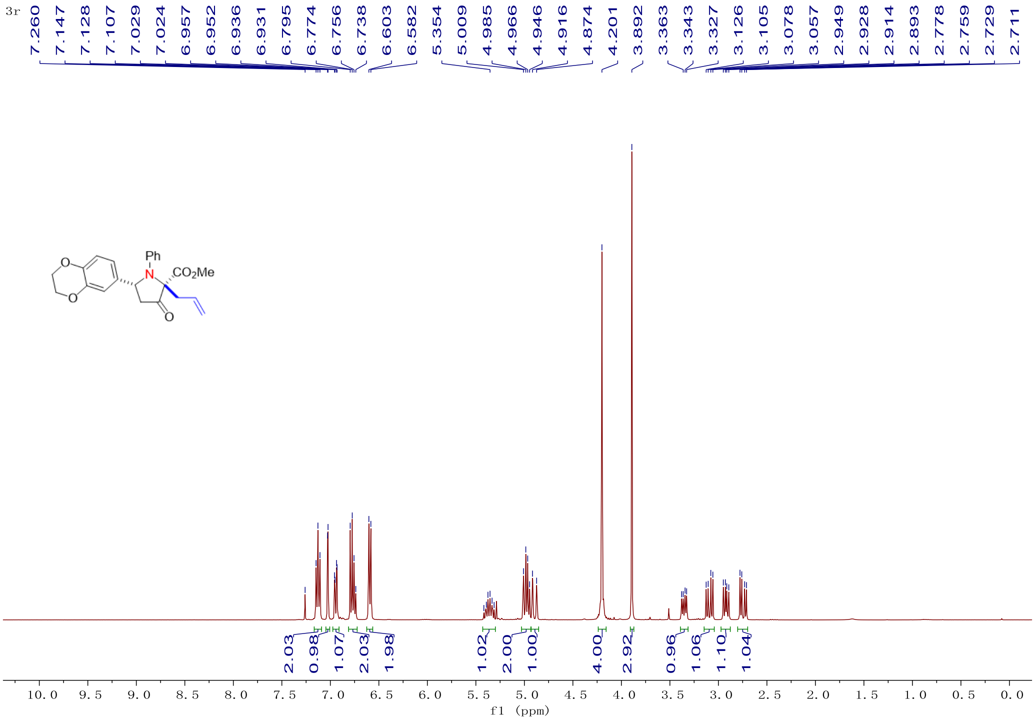

^{1}H NMR spectrum ($CDCl_3$, 400 MHz) of **3r**

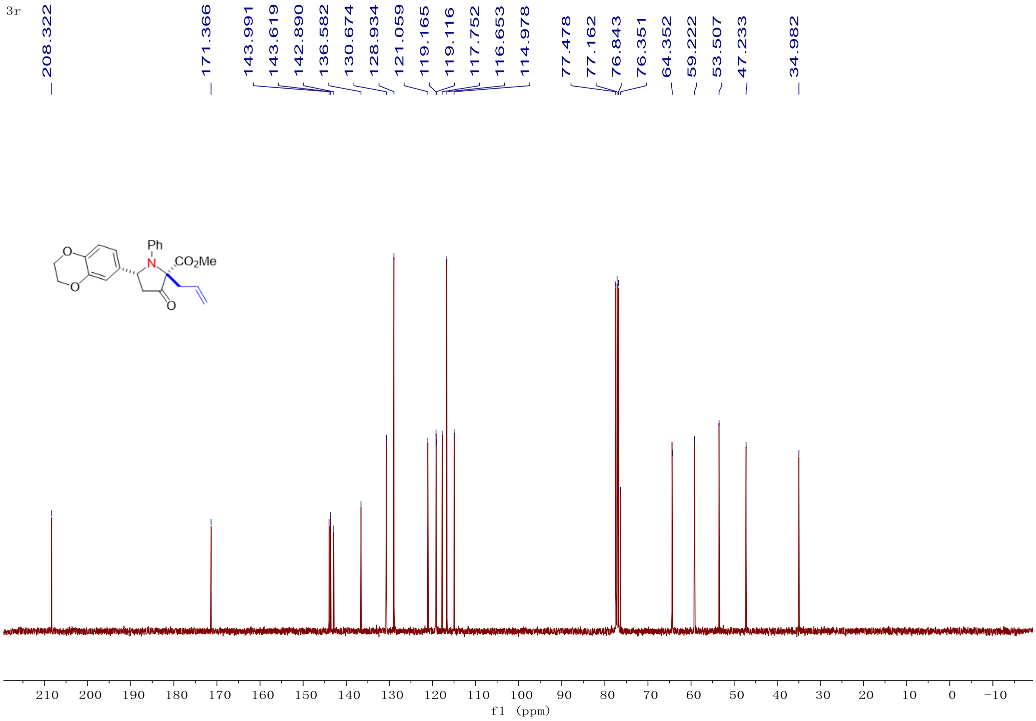

^{13}C NMR spectrum ($CDCl_3$, 100 MHz) of **3r**

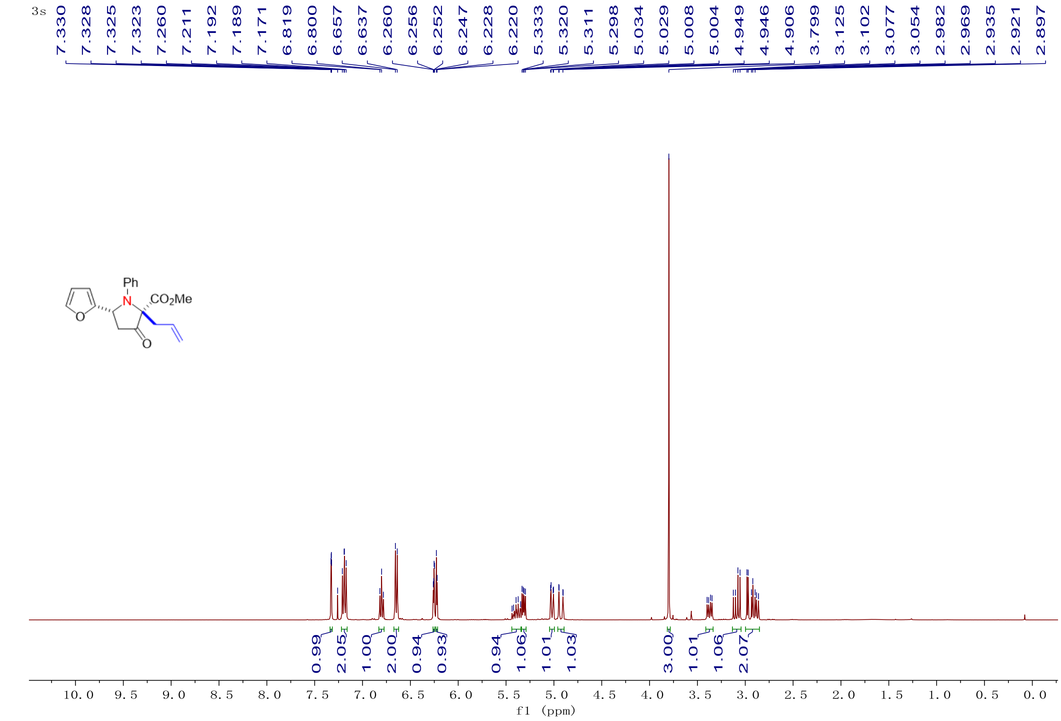

^{1}H NMR spectrum ($CDCl_3$, 400 MHz) of **3s**

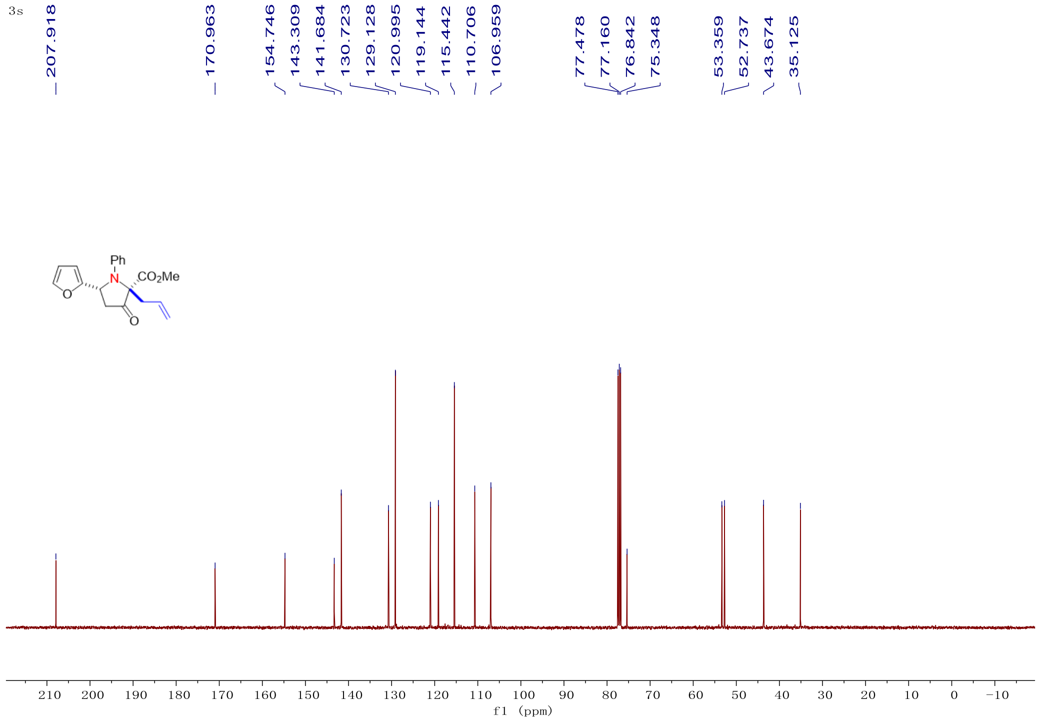

^{13}C NMR spectrum ($CDCl_3$, 100 MHz) of **3s**

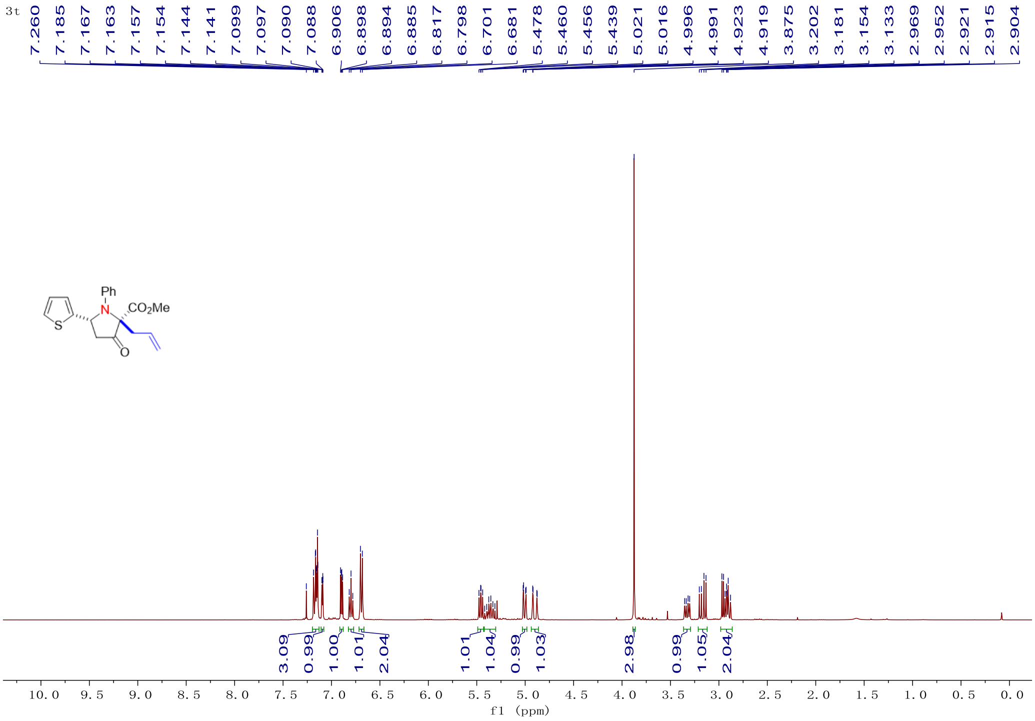

^{1}H NMR spectrum ($CDCl_3$, 400 MHz) of **3t**

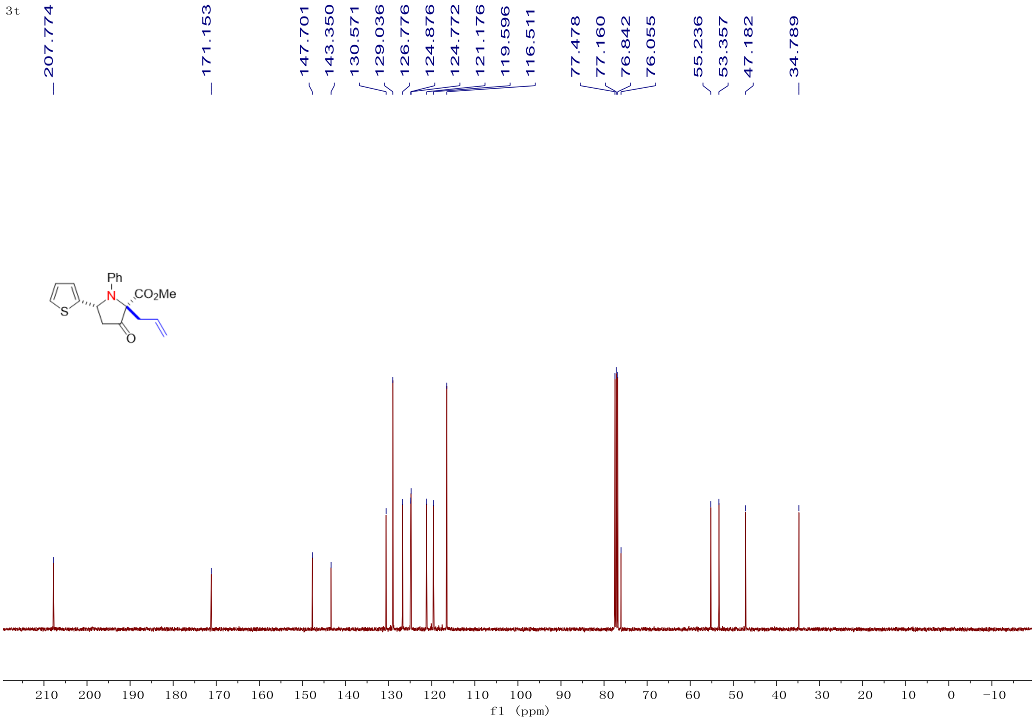

^{13}C NMR spectrum ($CDCl_3$, 100 MHz) of **3t**

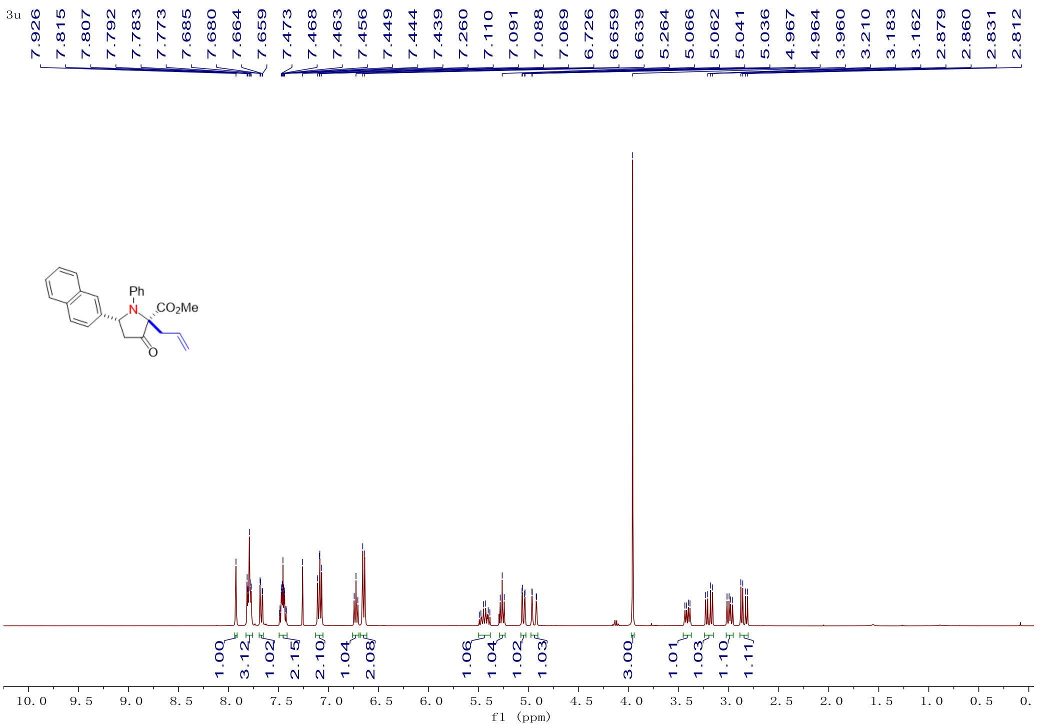

^{1}H NMR spectrum ($CDCl_3$, 400 MHz) of **3u**

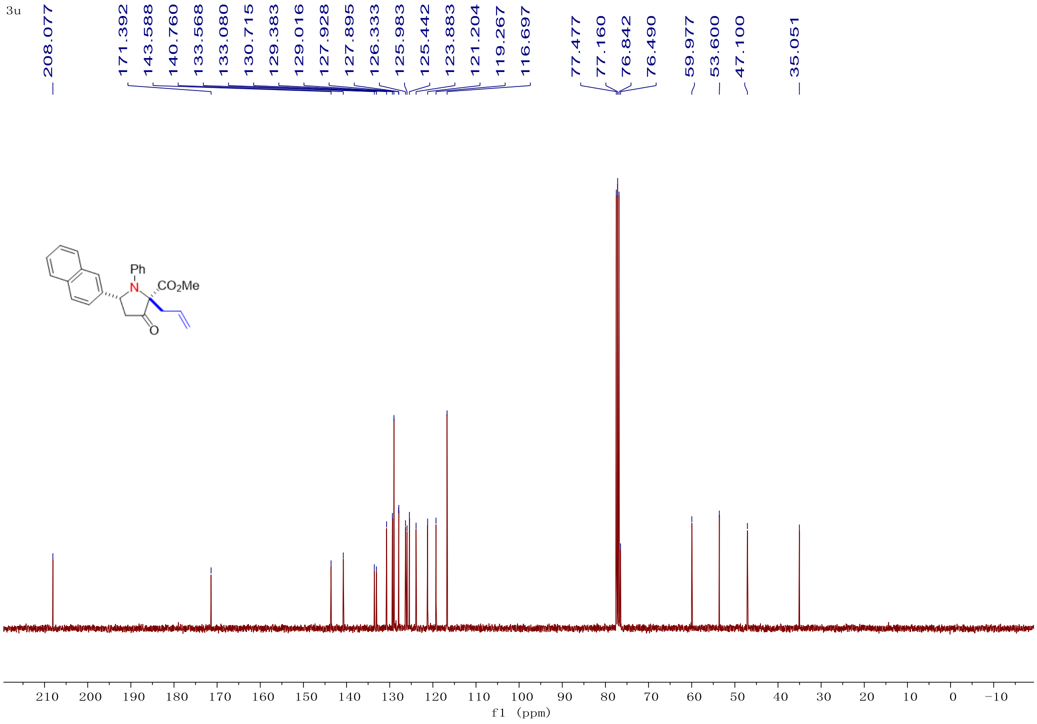

^{13}C NMR spectrum ($CDCl_3$, 100 MHz) of **3u**

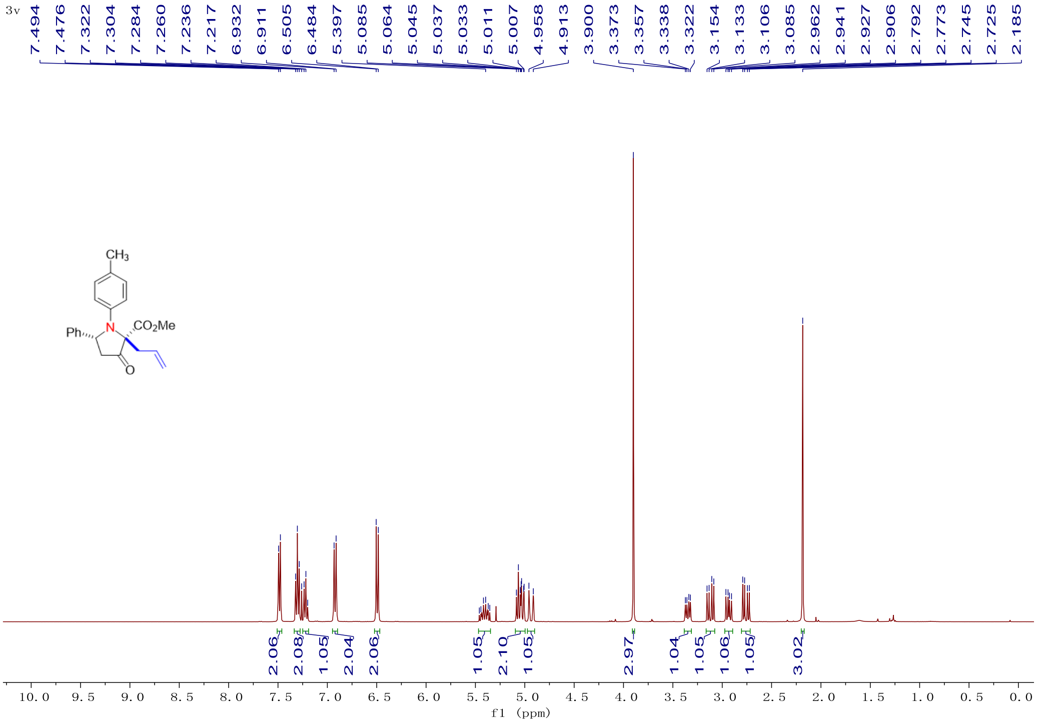

^{1}H NMR spectrum ($CDCl_3$, 400 MHz) of **3v**

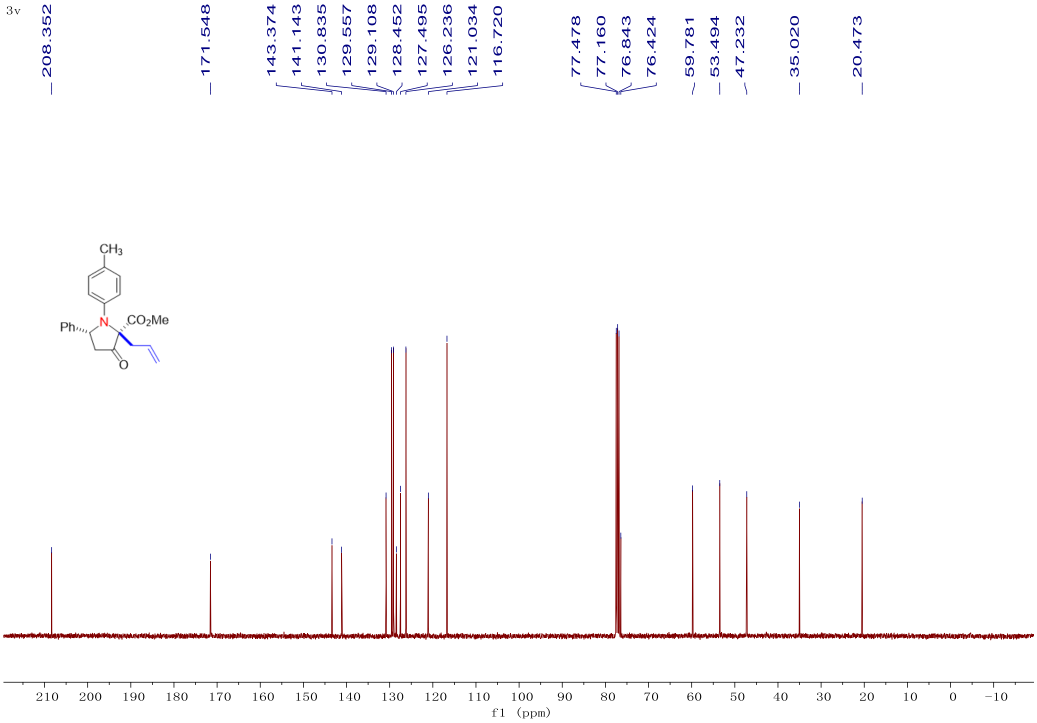

^{13}C NMR spectrum ($CDCl_3$, 100 MHz) of **3v**

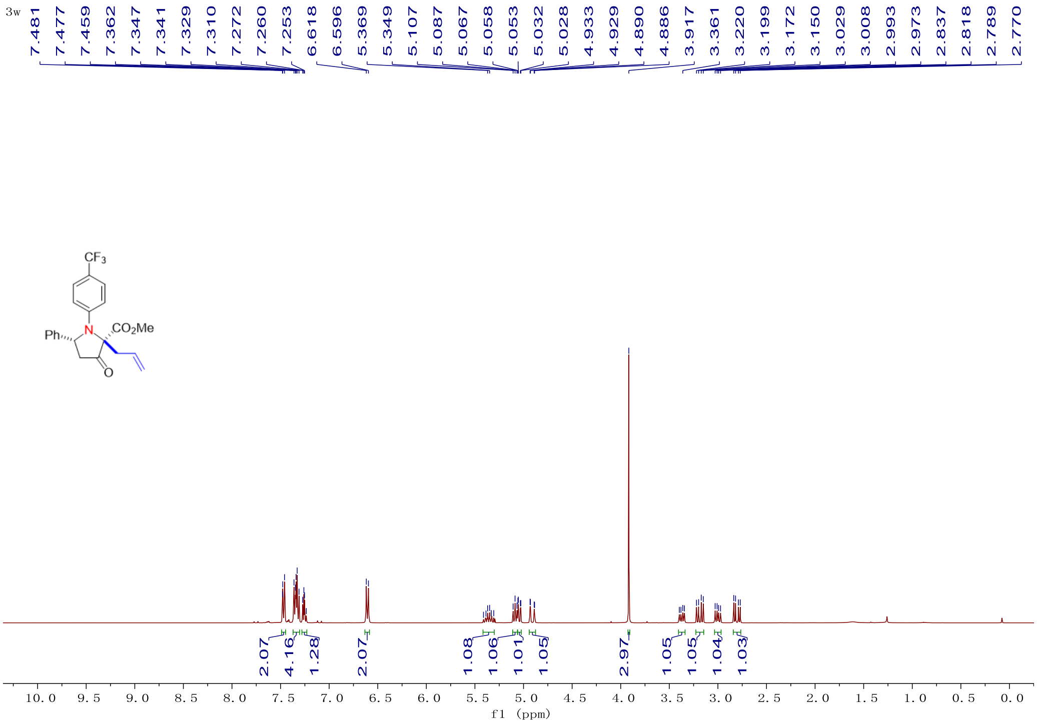

^{1}H NMR spectrum (CDCl3, 400 MHz) of **3w**

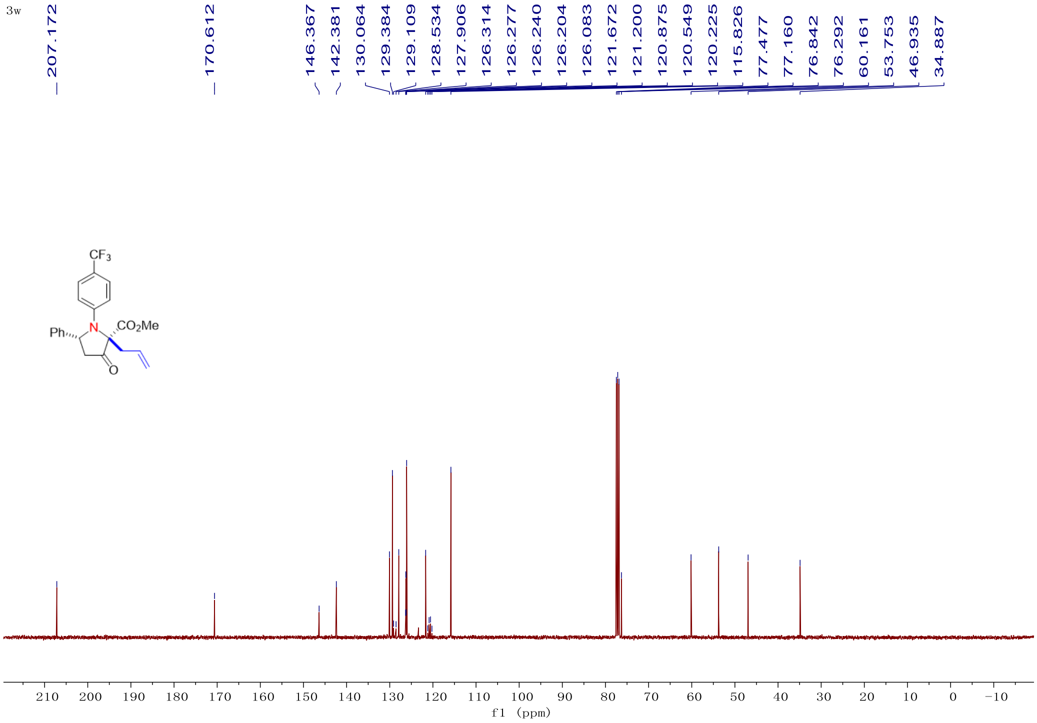

^{13}CNMR spectrum (CDCl3, 100 MHz) of **3w**

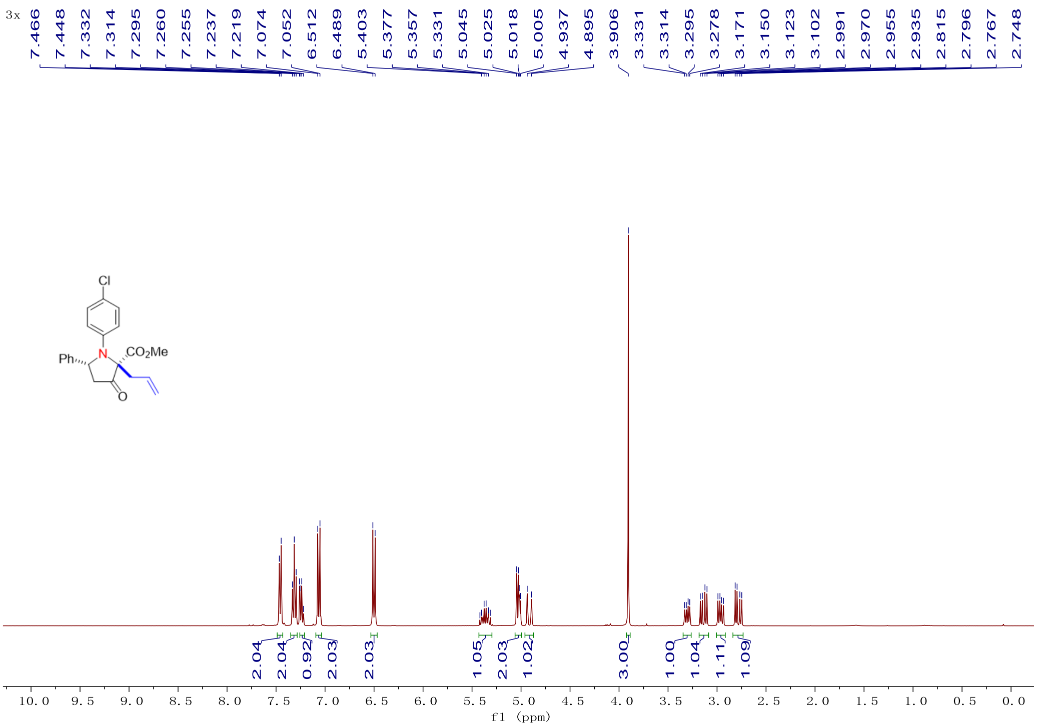

^{1}H NMR spectrum (CDCl$_3$, 400 MHz) of **3x**

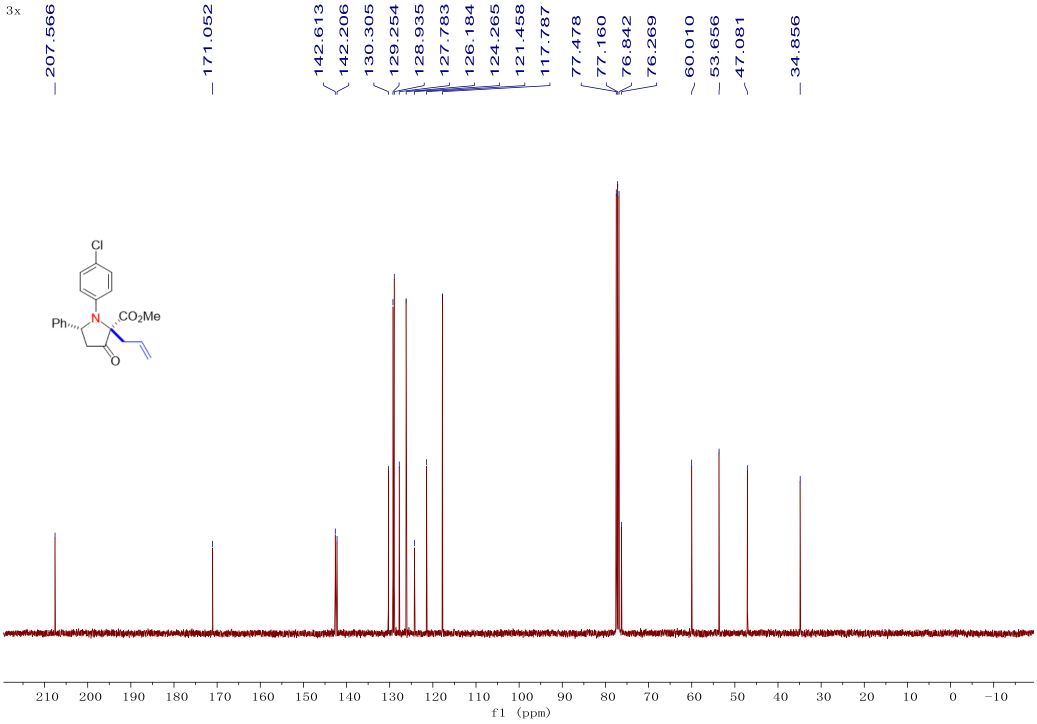

^{13}C NMR spectrum (CDCl$_3$, 100 MHz) of **3x**

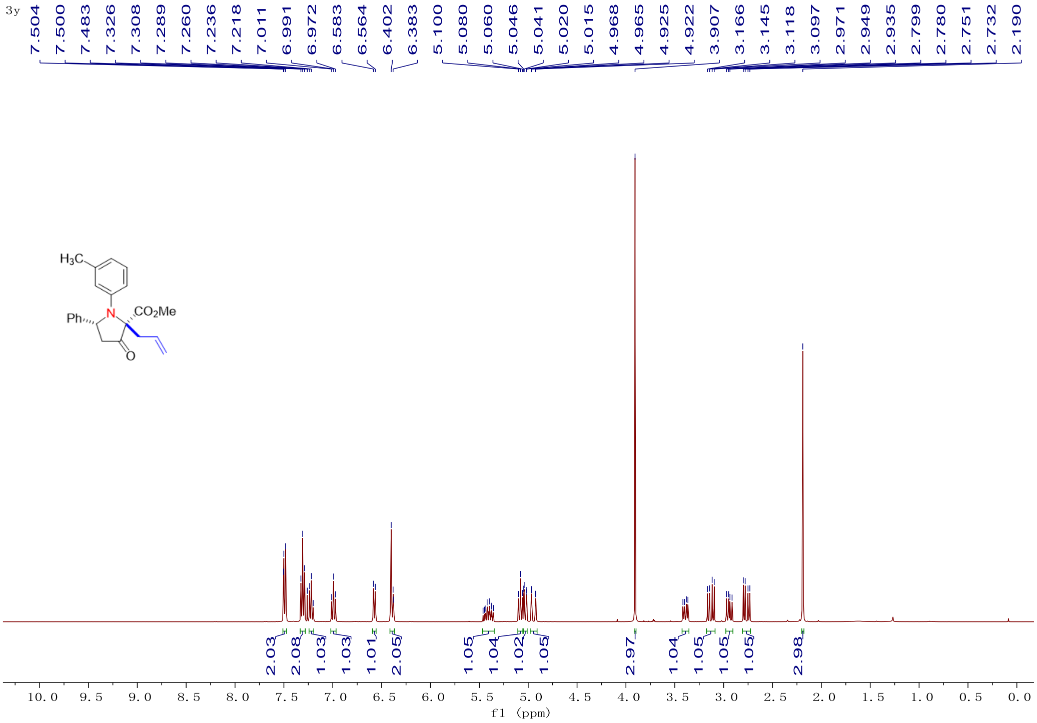

^{1}H NMR spectrum ($CDCl_3$, 400 MHz) of **3y**

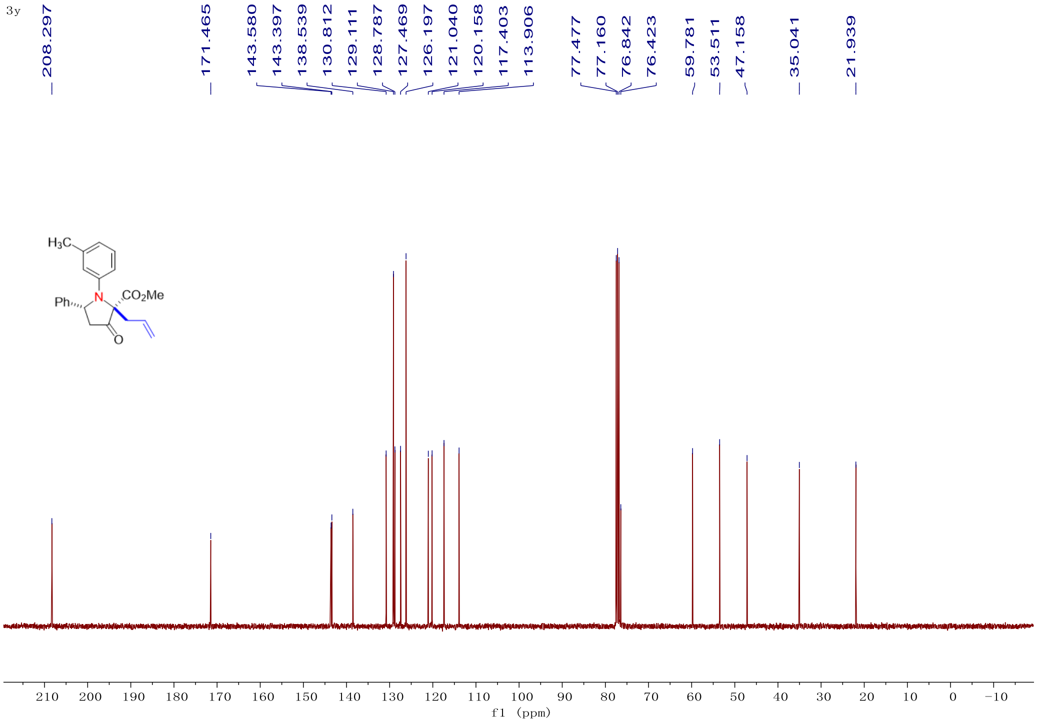

^{13}C NMR spectrum ($CDCl_3$, 100 MHz) of **3y**

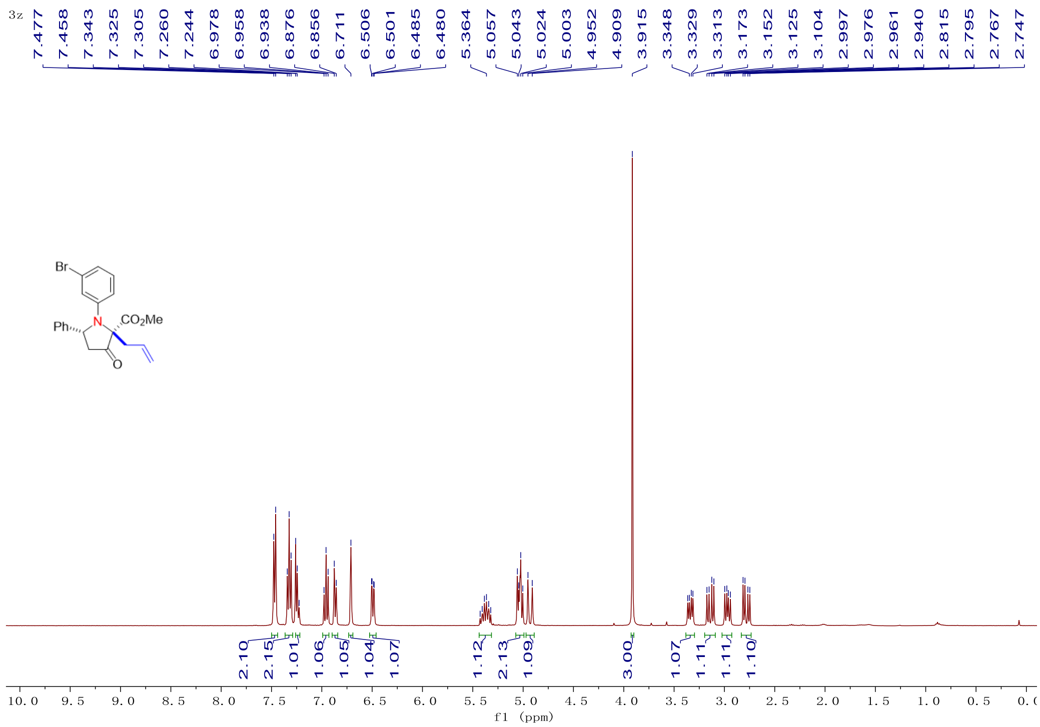

^{1}H NMR spectrum (CDCl$_3$, 400 MHz) of **3z**

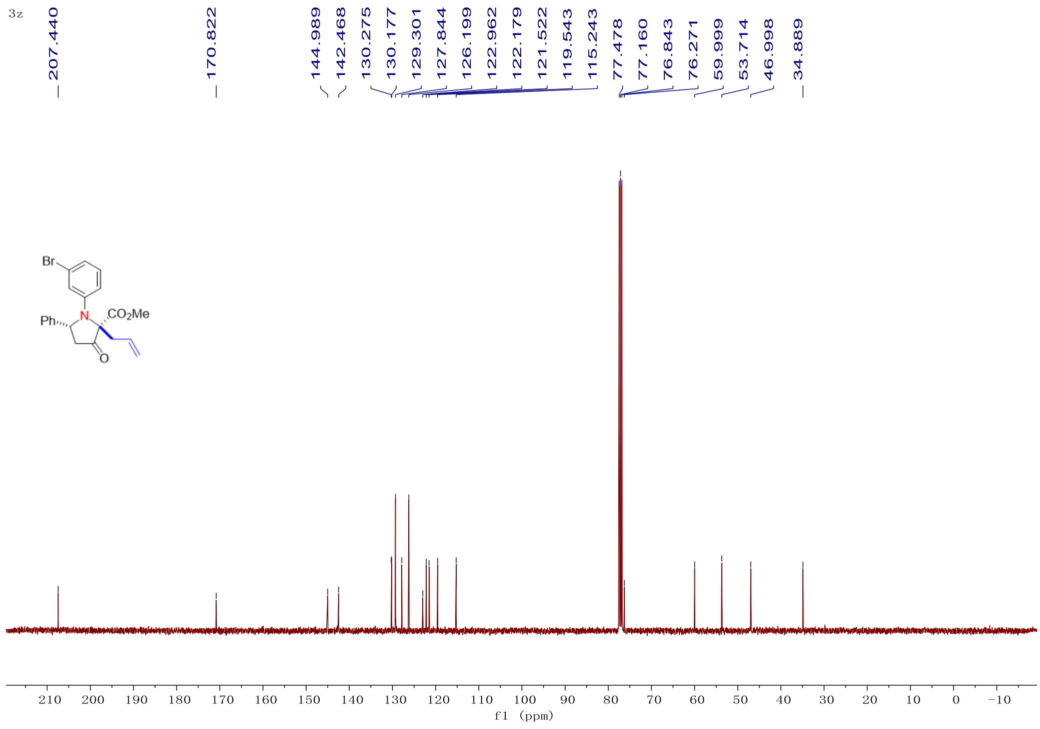

^{13}C NMR spectrum (CDCl$_3$, 100 MHz) of **3z**

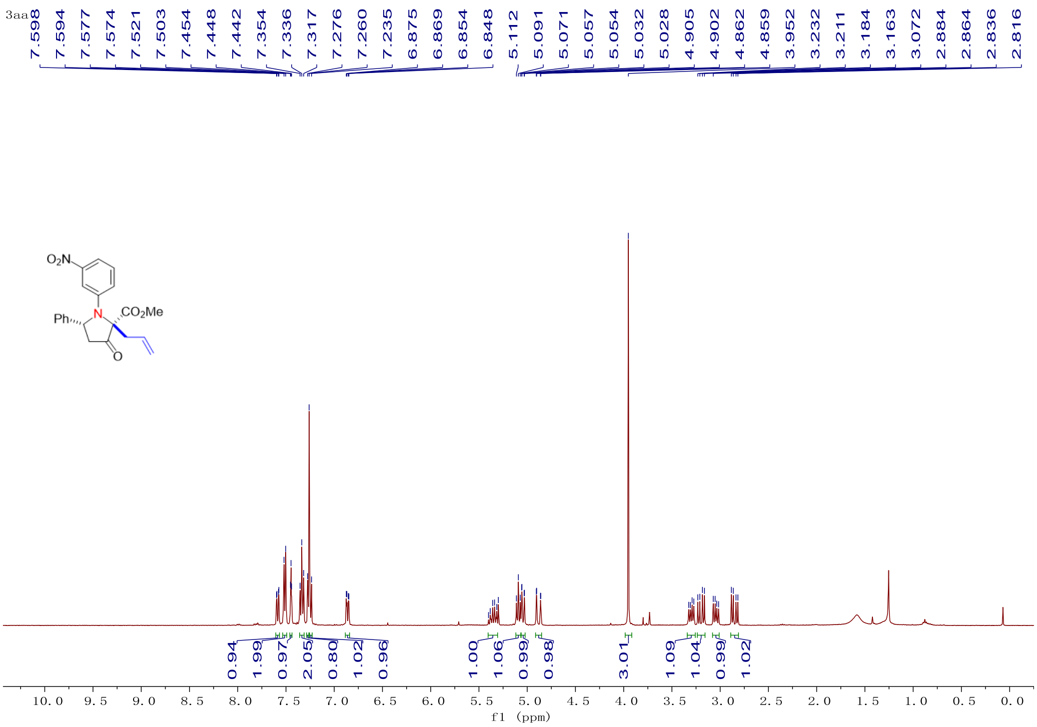

^{1}H NMR spectrum ($CDCl_3$, 400 MHz) of **3aa**

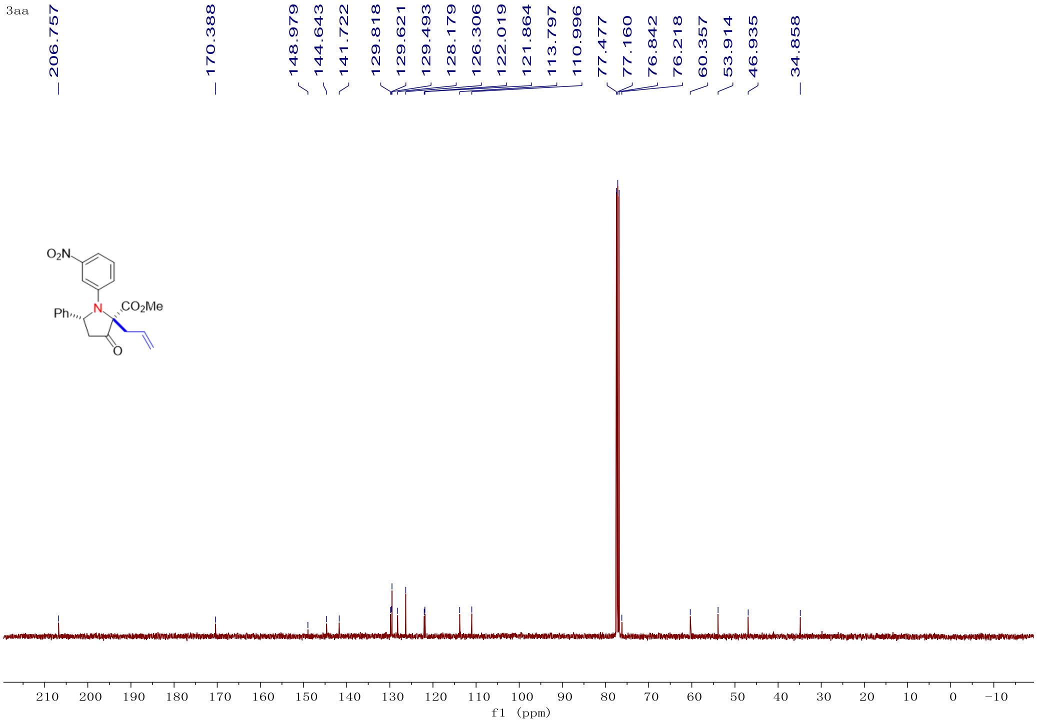

^{13}C NMR spectrum ($CDCl_3$, 100 MHz) of **3aa**

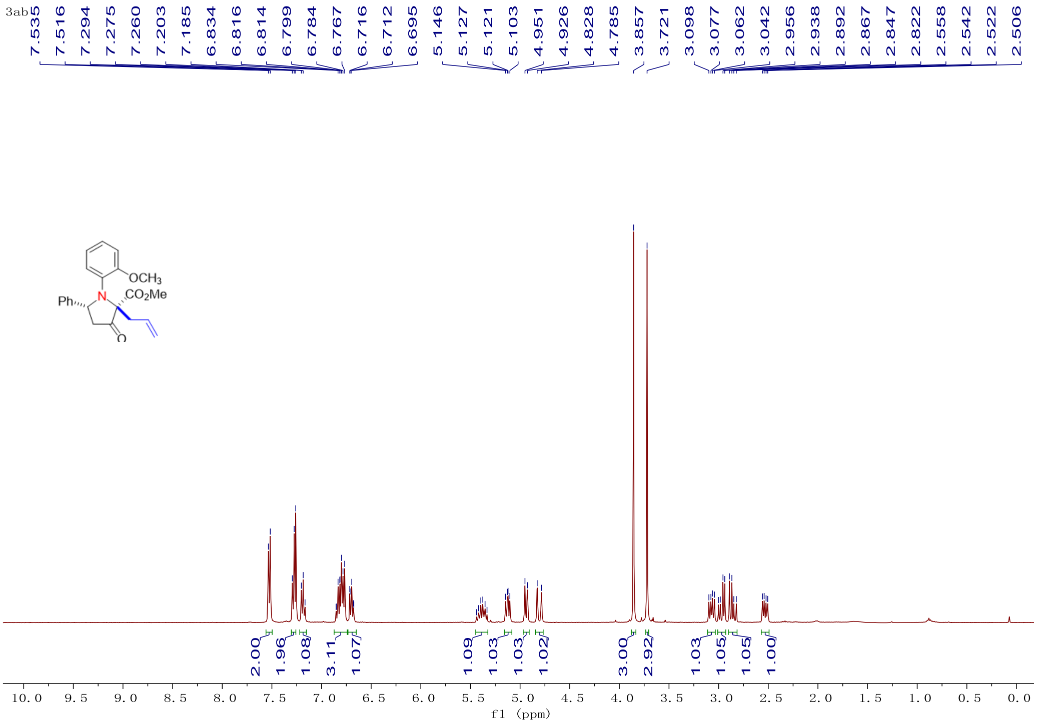

^{1}H NMR spectrum ($CDCl_3$, 400 MHz) of **3ab**

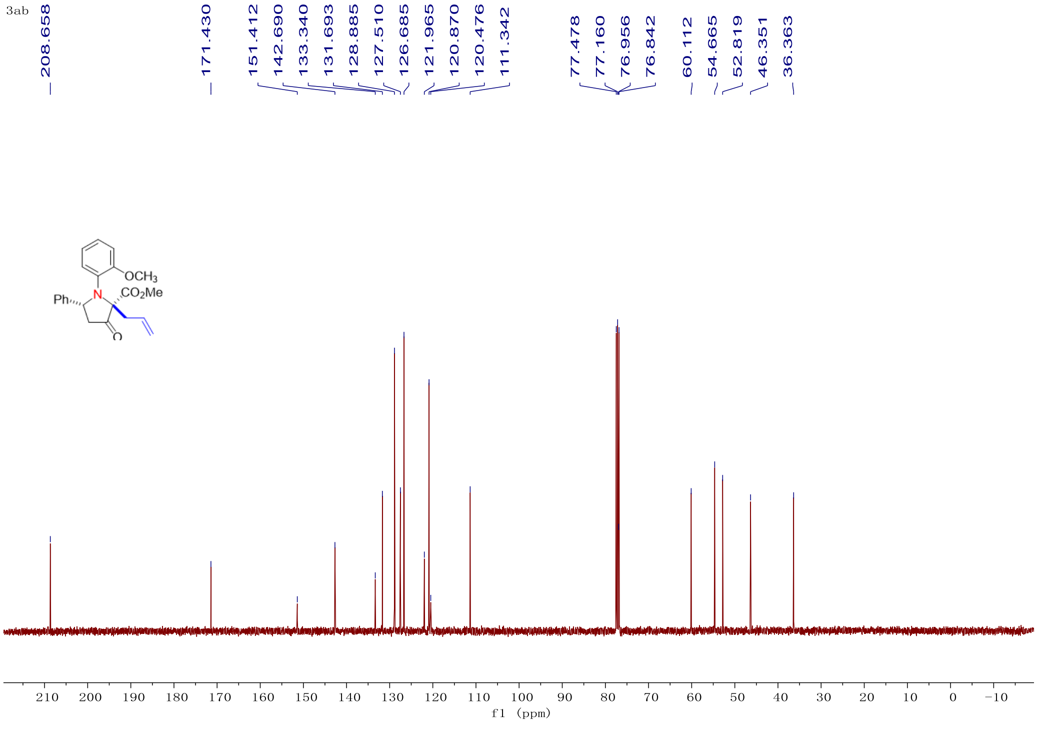

^{13}C NMR spectrum ($CDCl_3$, 100 MHz) of **3ab**

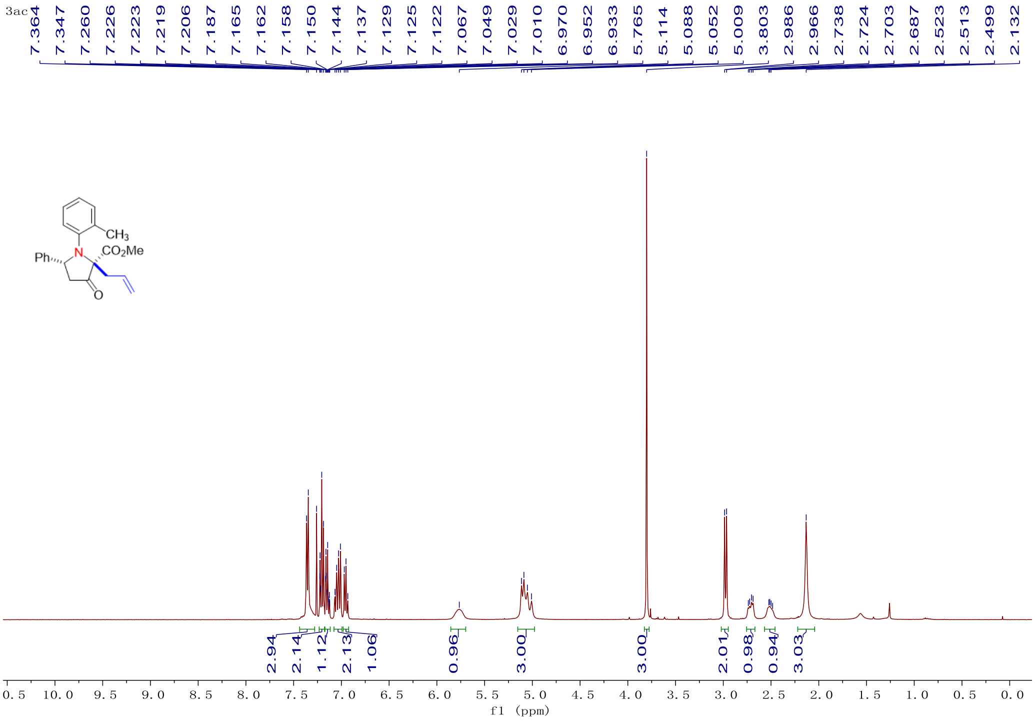

^{1}H NMR spectrum ($CDCl_3$, 400 MHz) of **3ac**

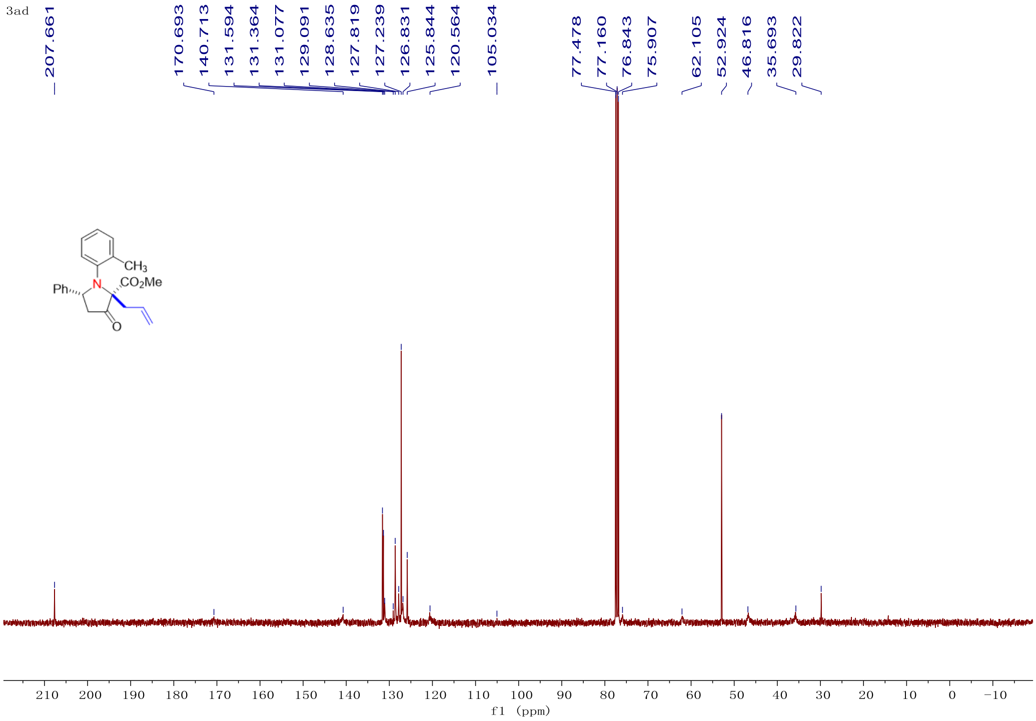

^{13}C NMR spectrum ($CDCl_3$, 100 MHz) of **3ac**

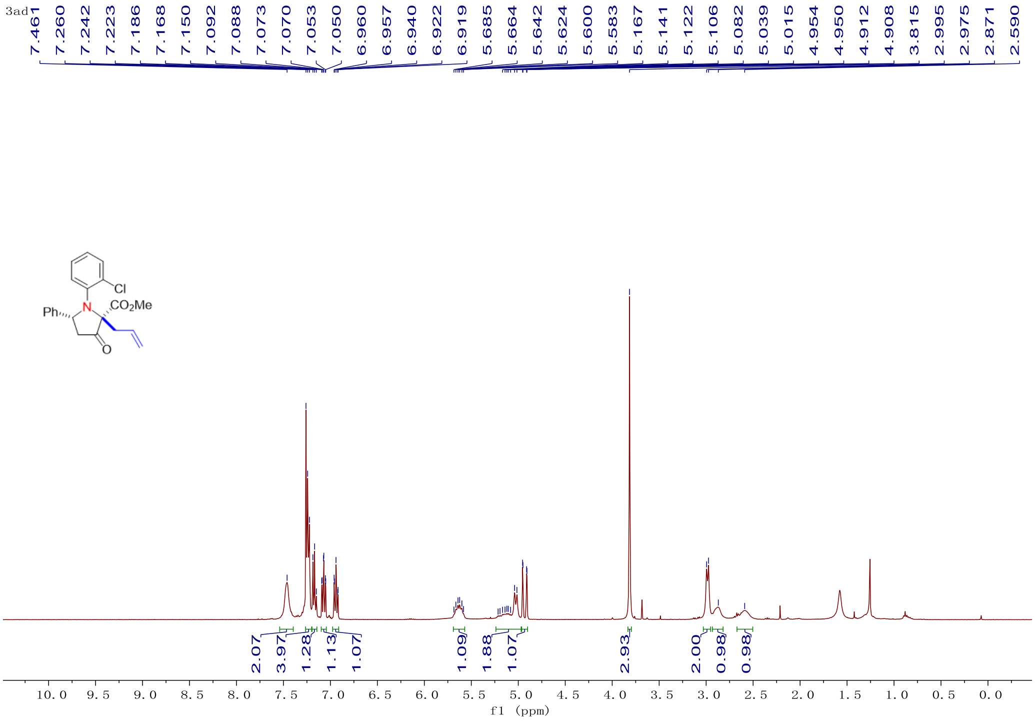

^{1}H NMR spectrum ($CDCl_3$, 400 MHz) of **3ad**

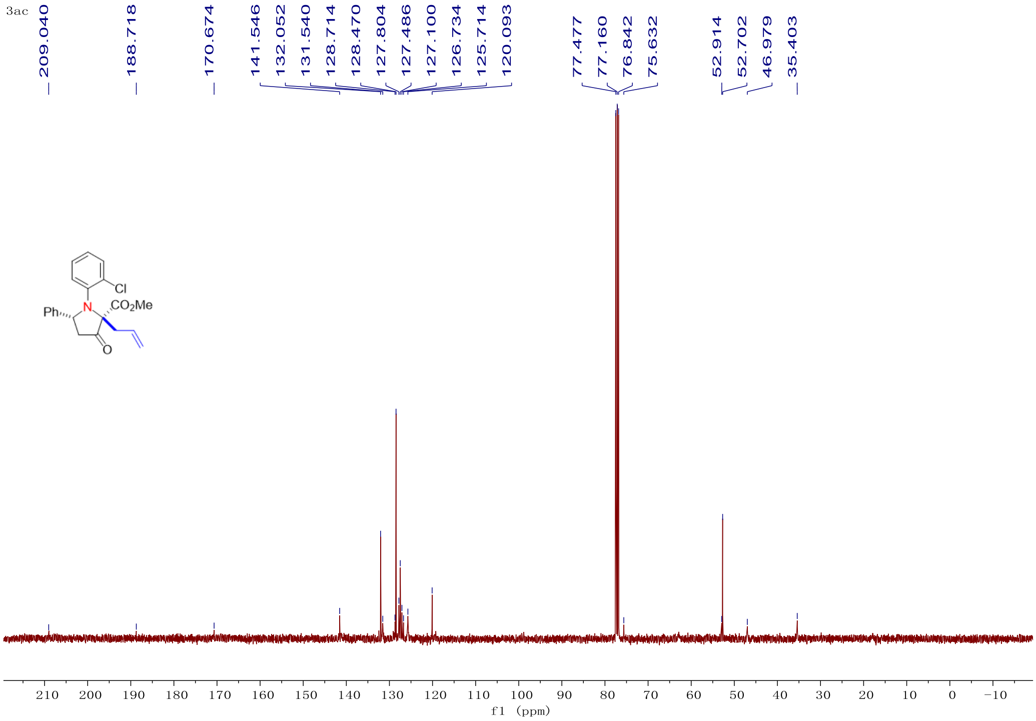

^{13}C NMR spectrum ($CDCl_3$, 100 MHz) of **3ad**

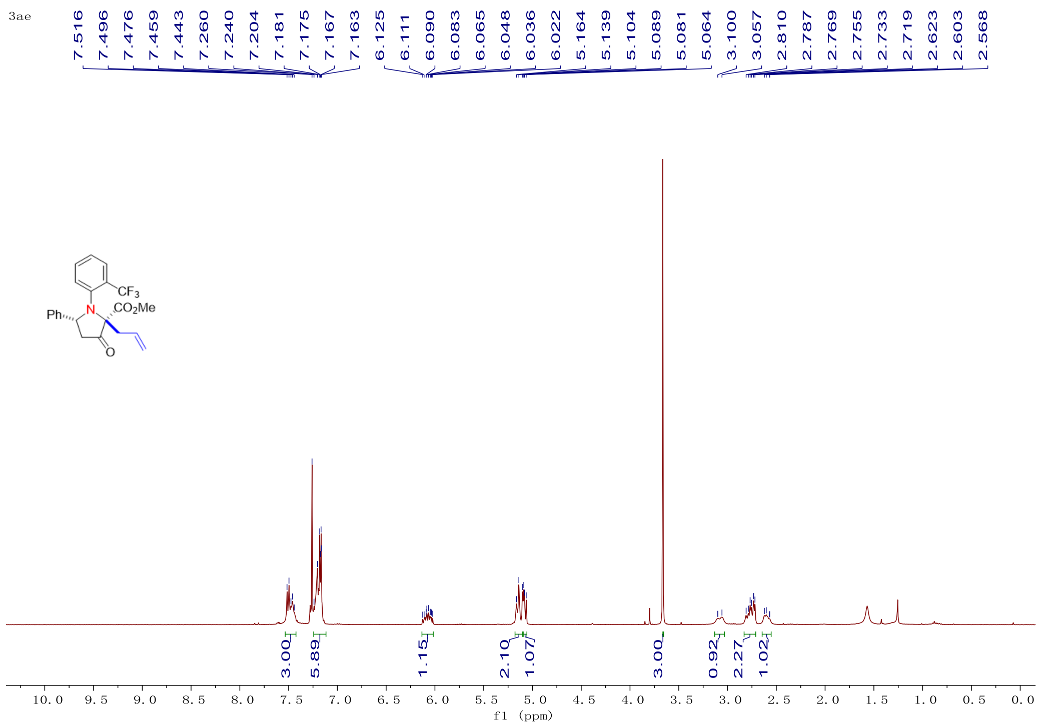

^{1}H NMR spectrum ($CDCl_3$, 400 MHz) of **3ae**

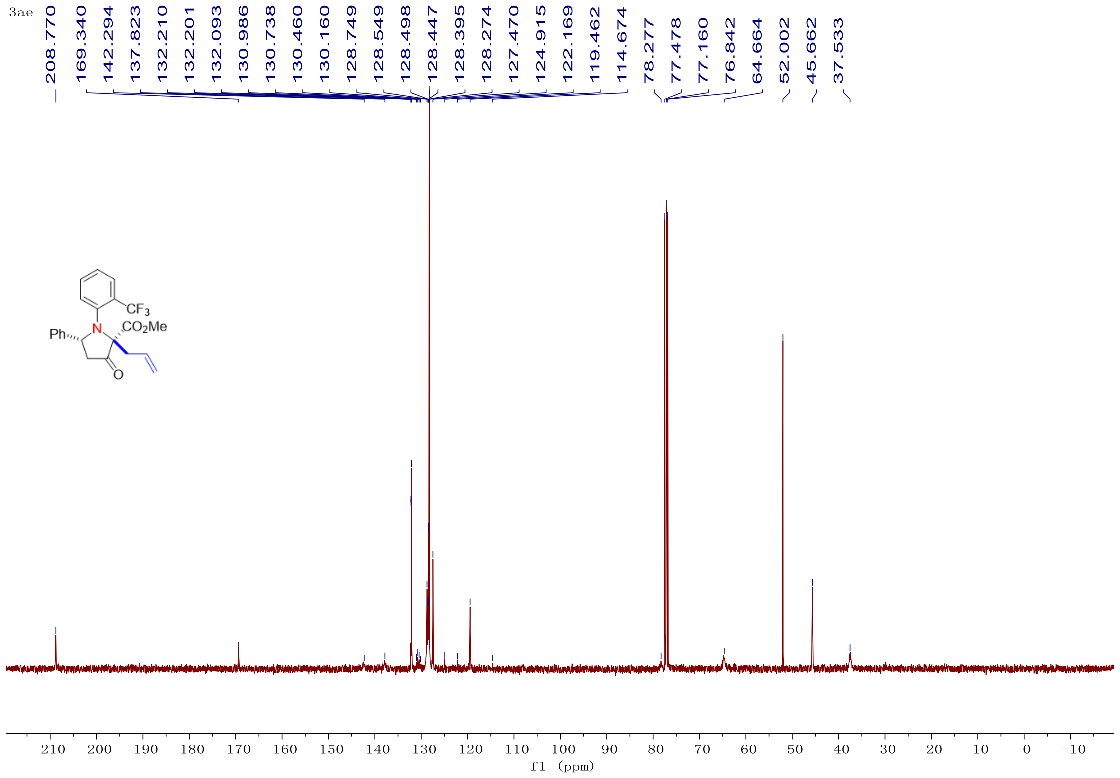

^{13}C NMR spectrum ($CDCl_3$, 100 MHz) of **3ae**

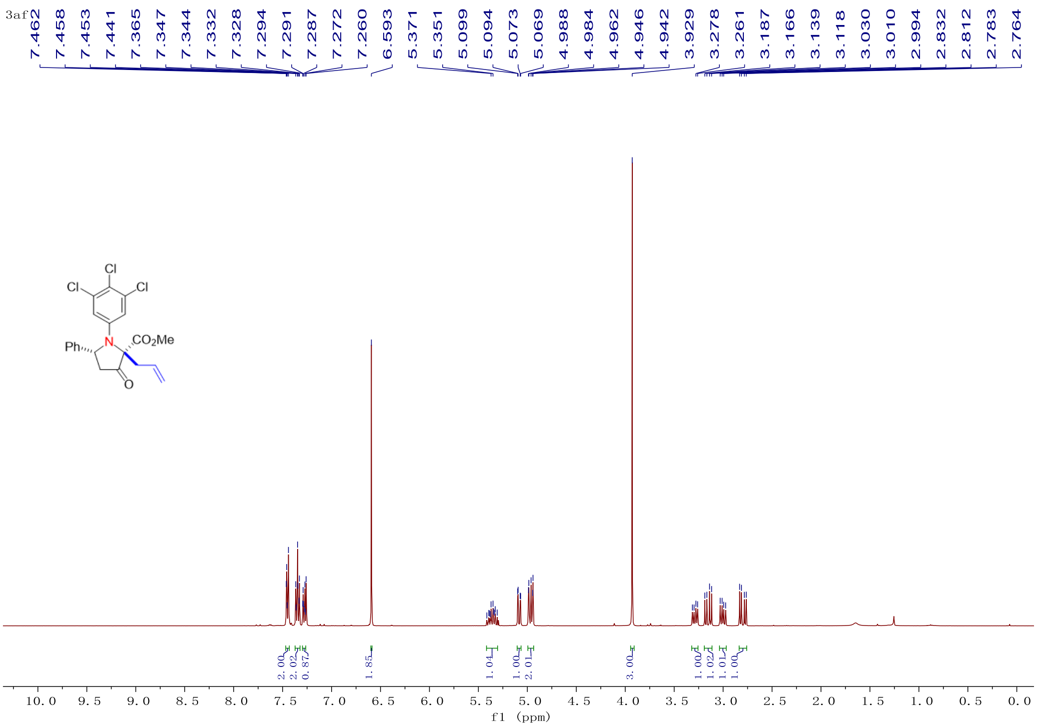

^{1}H NMR spectrum (CDCl$_3$, 400 MHz) of **3af**

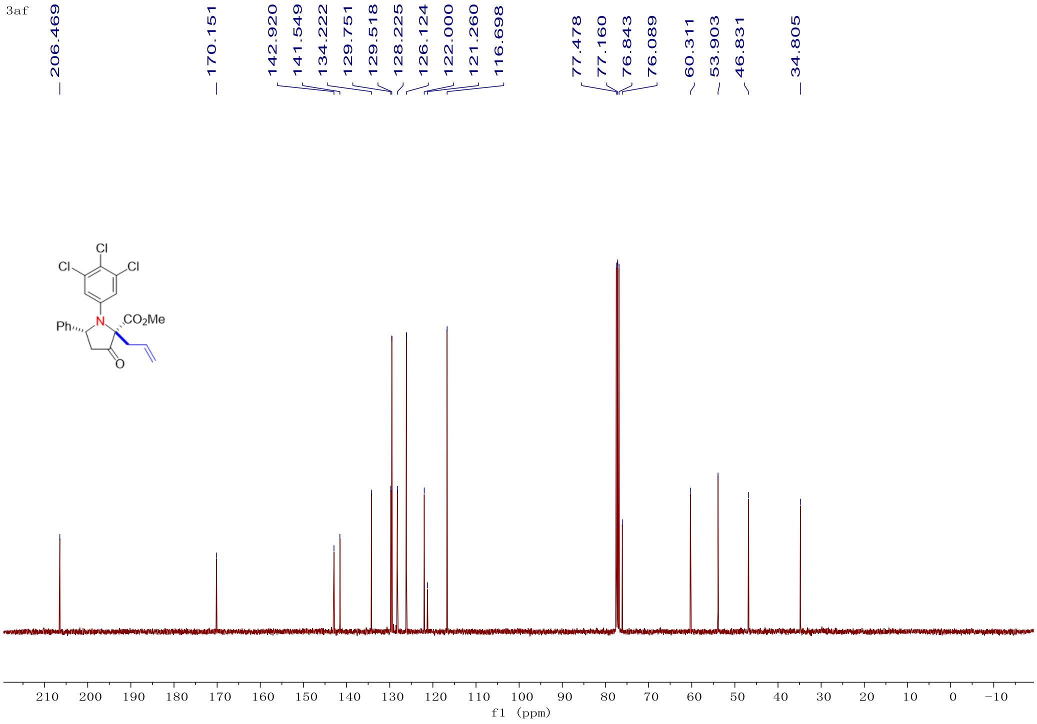

^{13}C NMR spectrum (CDCl$_3$, 100 MHz) of **3af**

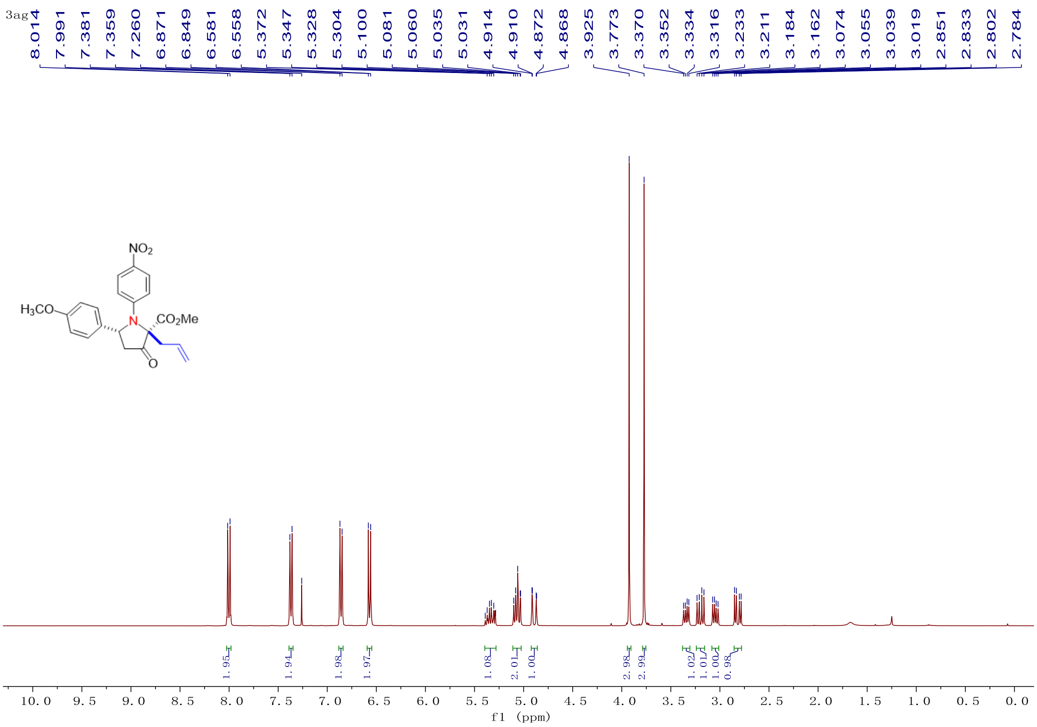

^{1}H NMR spectrum ($CDCl_3$, 400 MHz) of **3ag**

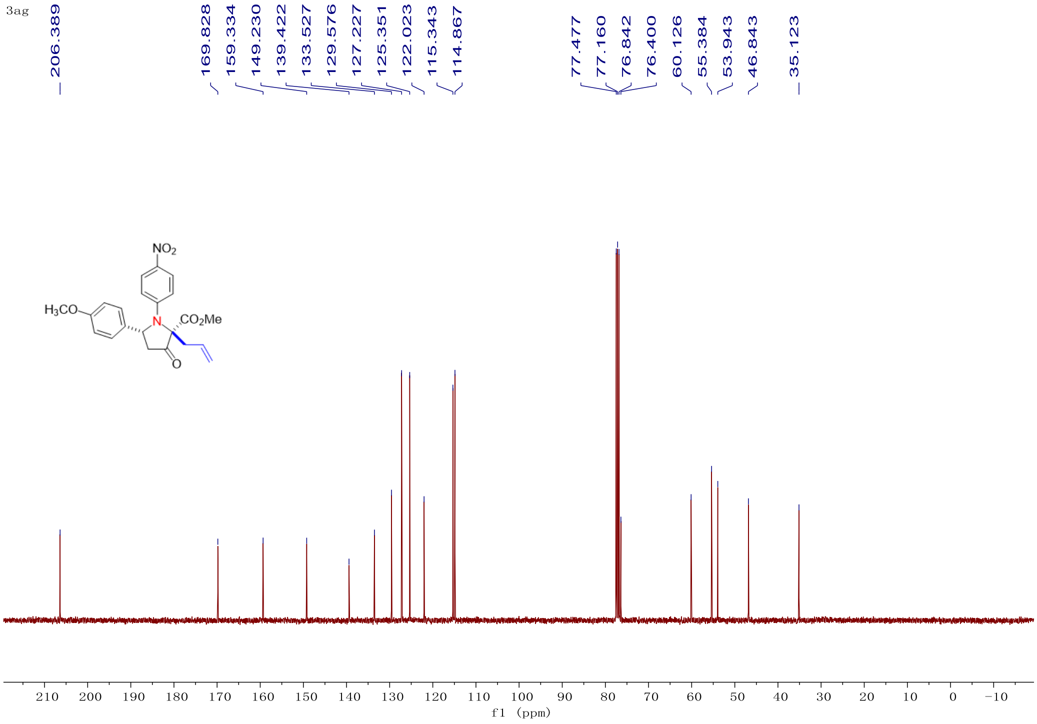

^{13}C NMR spectrum ($CDCl_3$, 100 MHz) of **3ag**

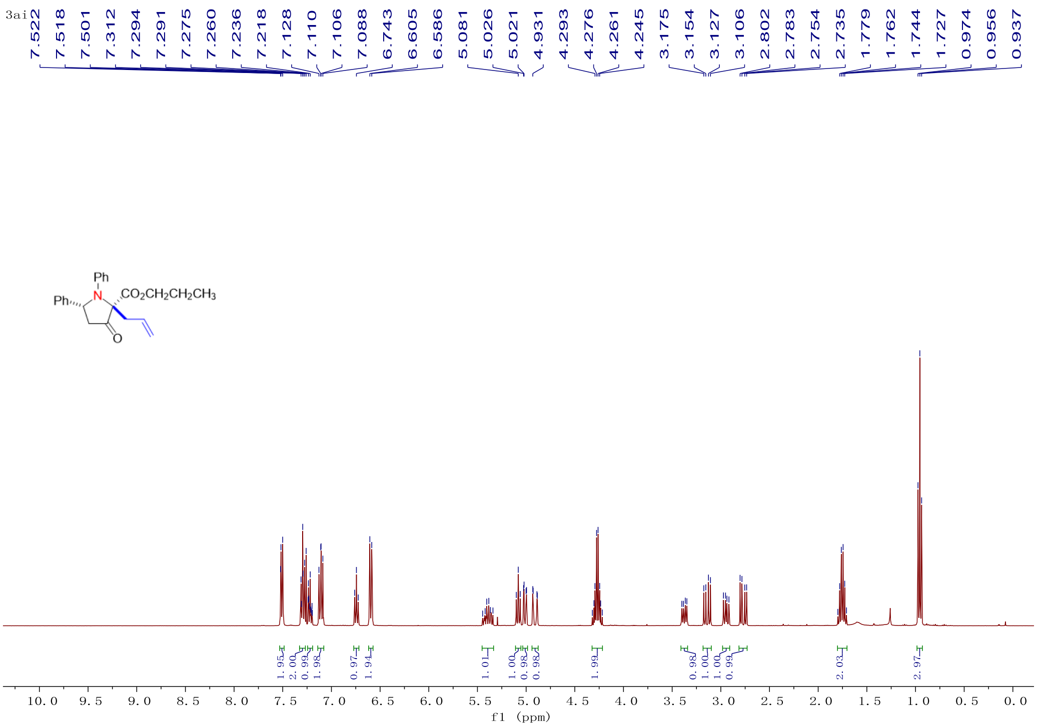

^{1}H NMR spectrum ($CDCl_3$, 400 MHz) of **3ai**

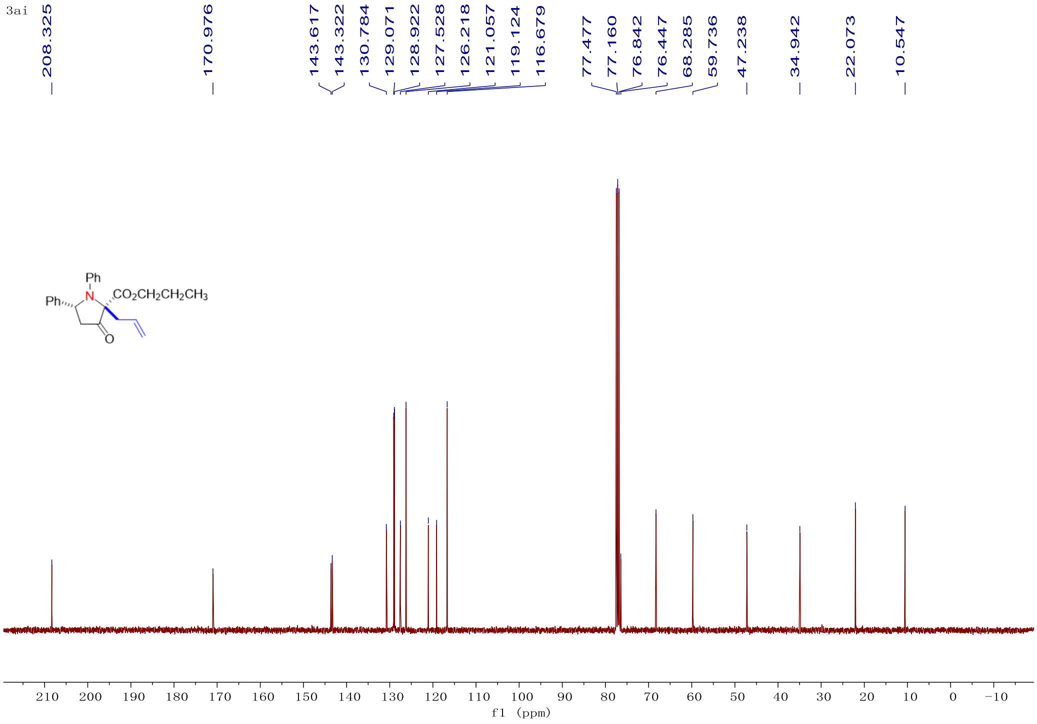

^{13}C NMR spectrum ($CDCl_3$, 100 MHz) of **3ai**

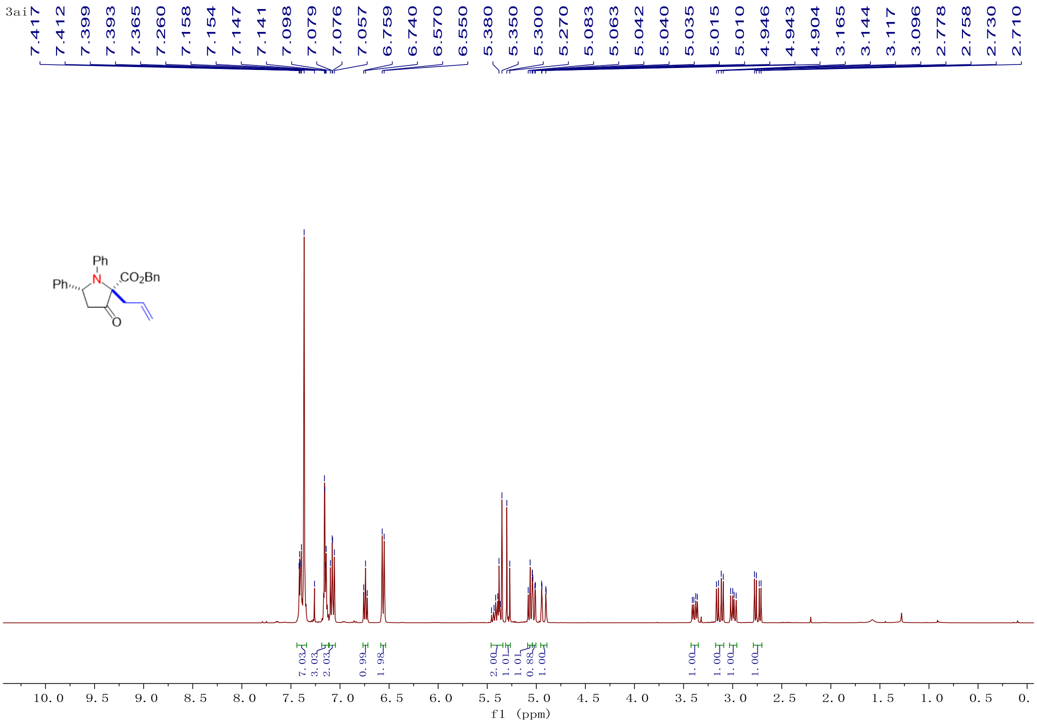

^{1}H NMR spectrum ($CDCl_3$, 400 MHz) of **3ai**

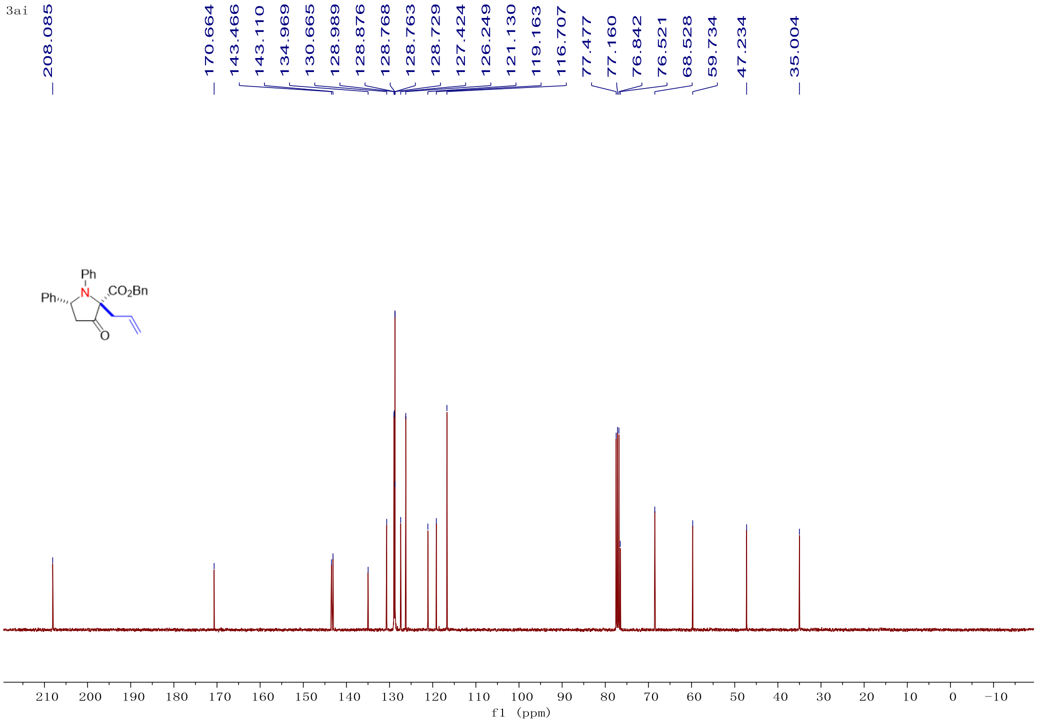

^{13}C NMR spectrum ($CDCl_3$, 100 MHz) of **3ai**

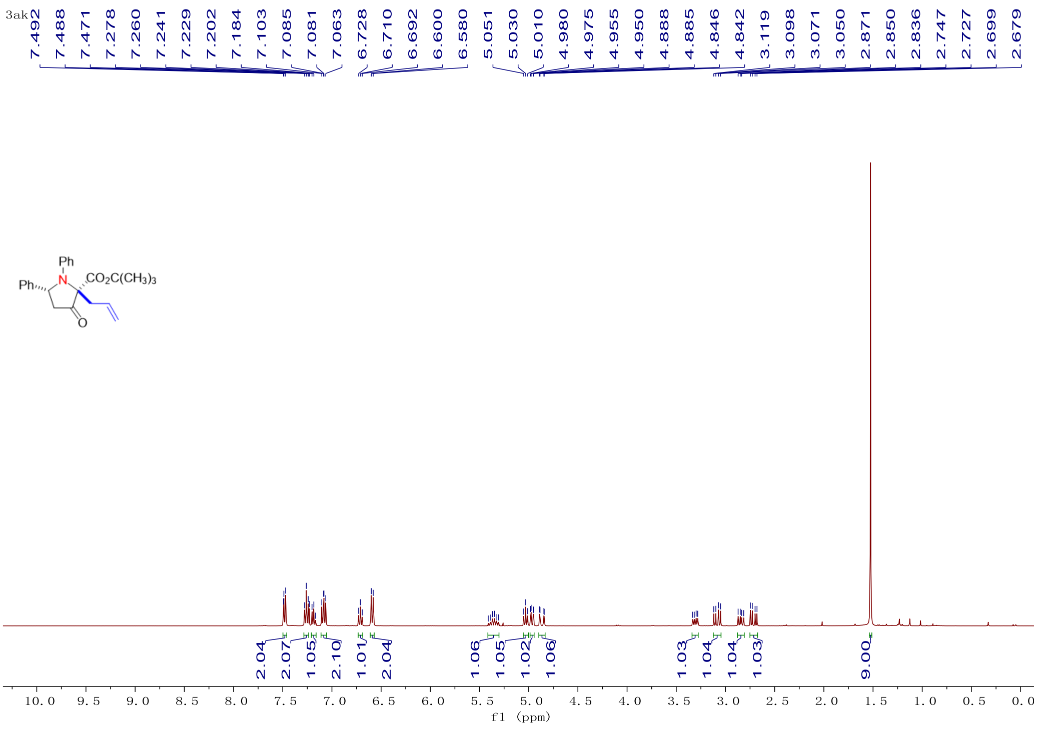

^{1}H NMR spectrum ($CDCl_3$, 400 MHz) of **3aj**

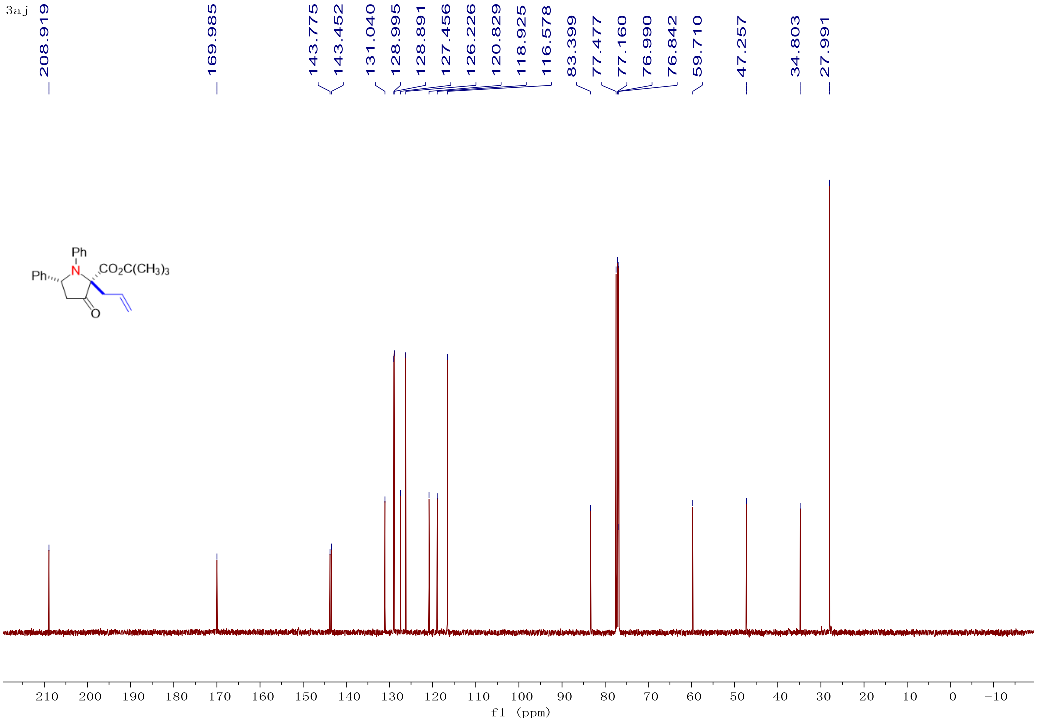

^{13}C NMR spectrum ($CDCl_3$, 100 MHz) of **3aj**

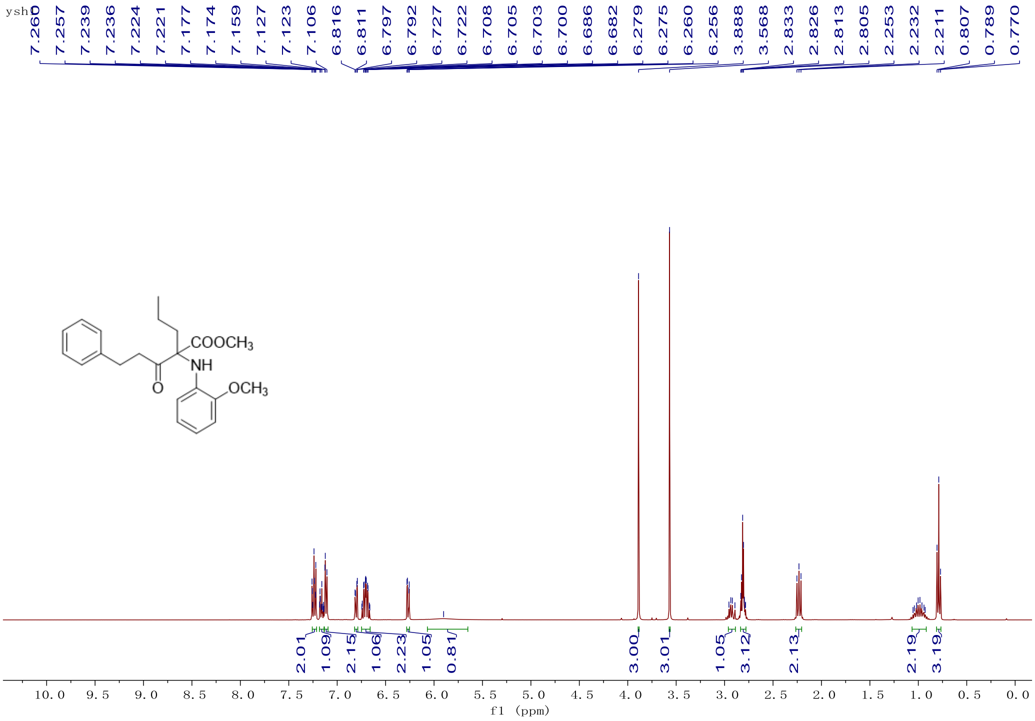

^{1}H NMR spectrum (CDCl3, 400 MHz) of **5**

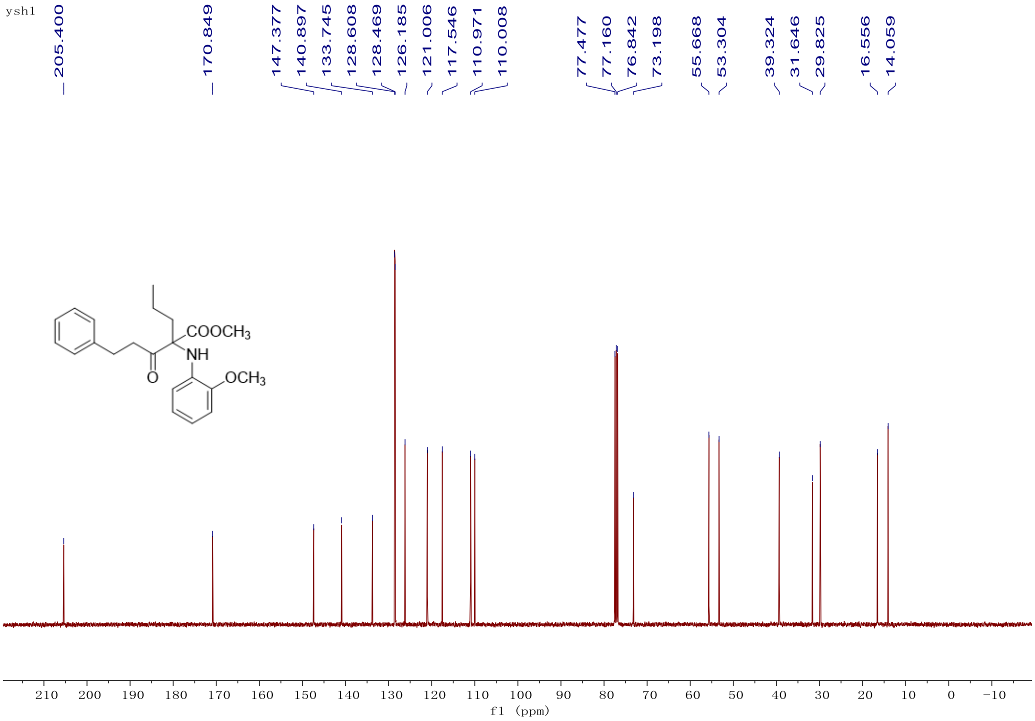

^{13}C NMR spectrum (CDCl3, 100 MHz) of **5**

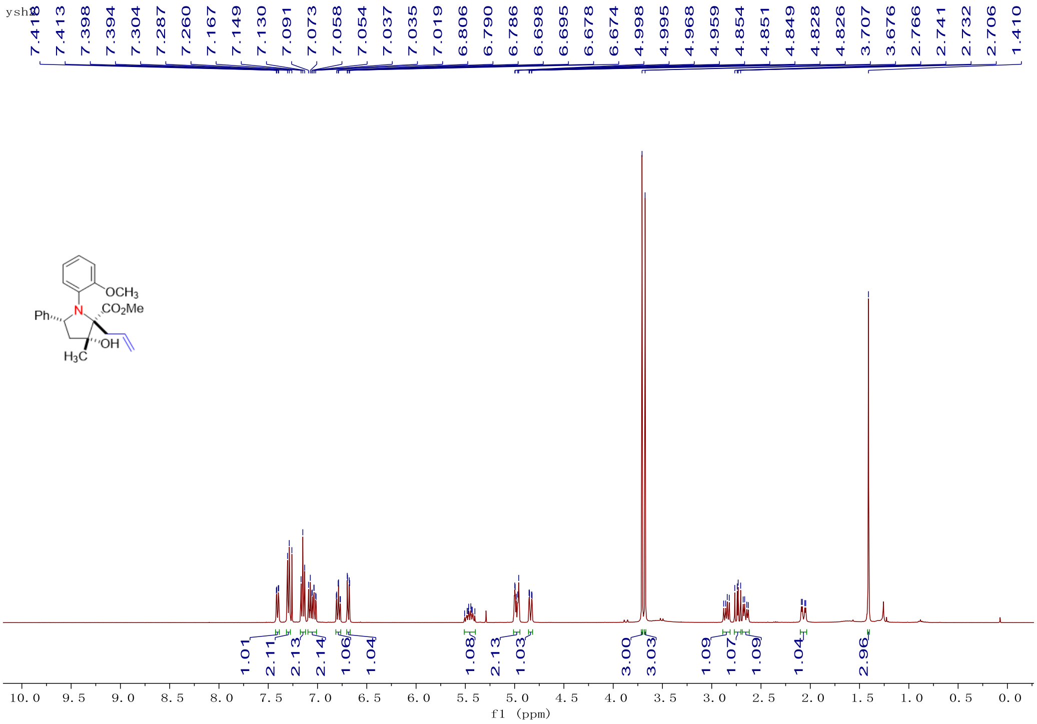

^{1}H NMR spectrum ($CDCl_3$, 400 MHz) of **6**

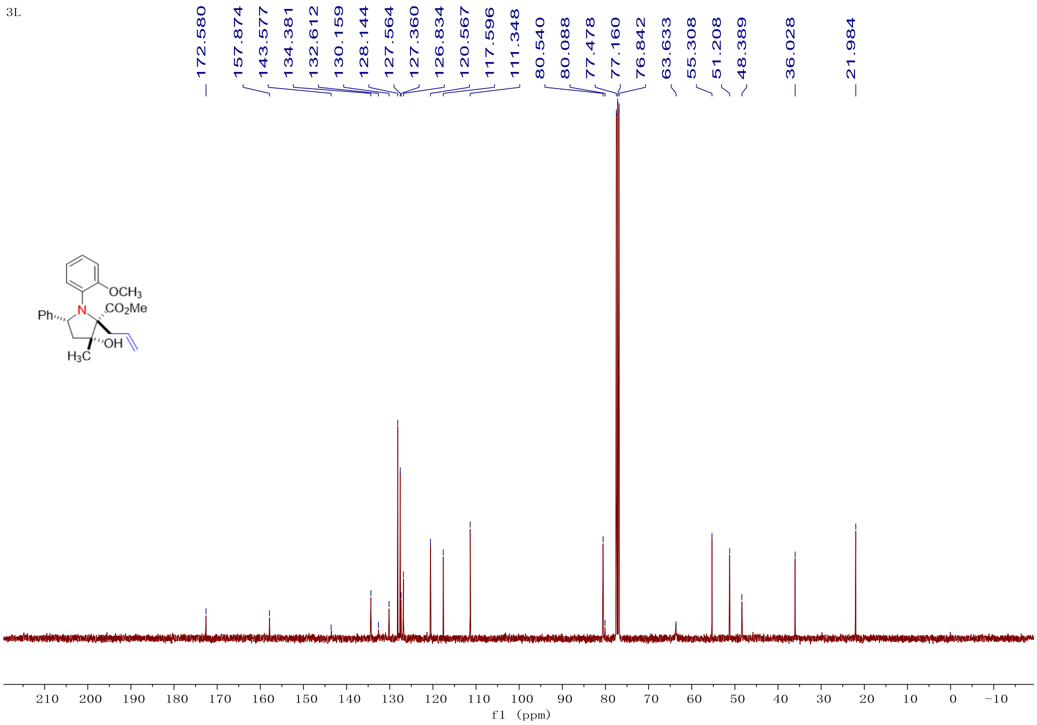

^{13}C NMR spectrum ($CDCl_3$, 100 MHz) of **6**

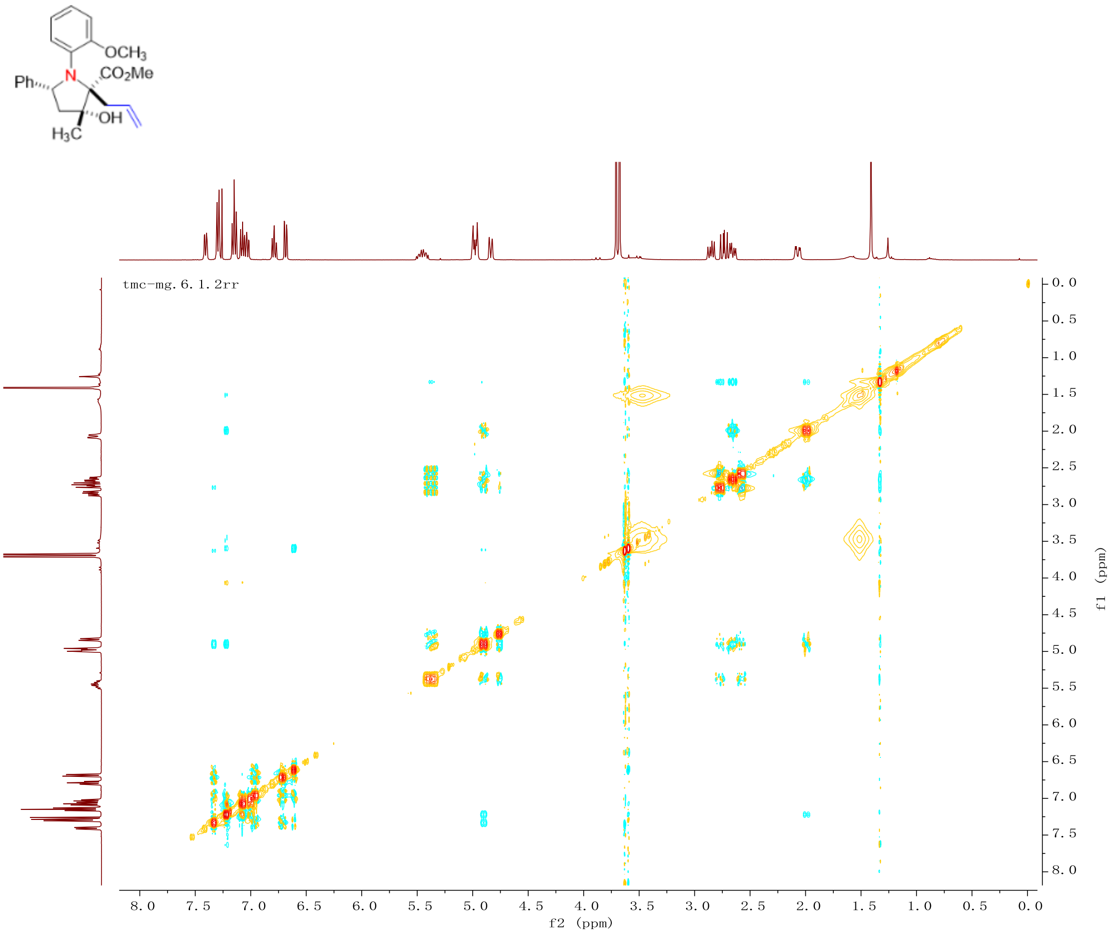

NOESY of **6** (600 MHz, CDCl3)

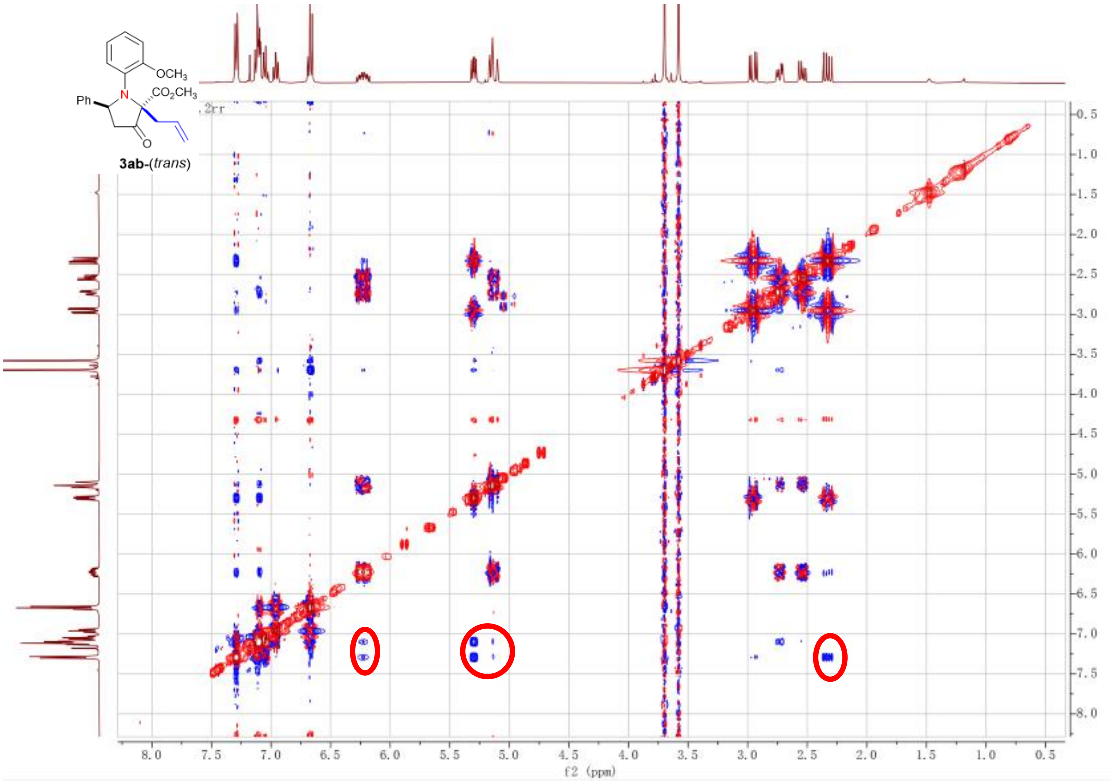

NOESY of **3ab-(*trans*)** (400 MHz, CDCl3)

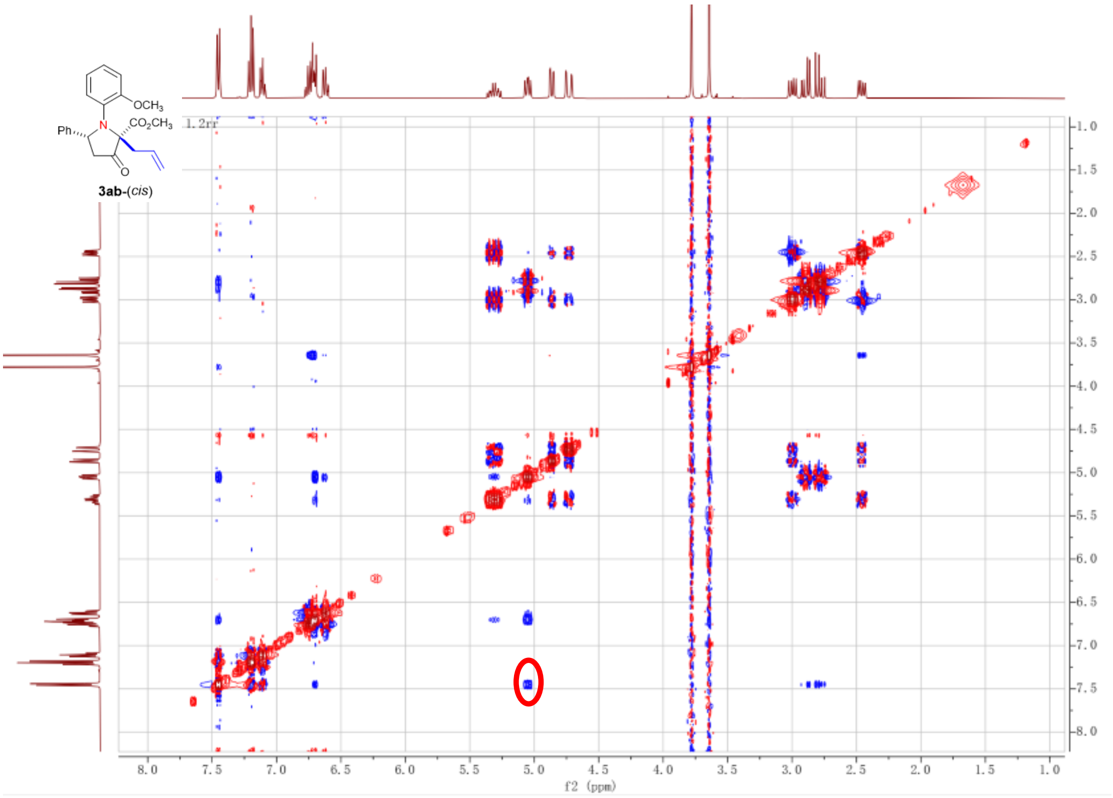

NOESY of **3ab-(*cis*)** (400 MHz, $CDCl_3$)

Figure S1. X-Ray crystal structure of the compound 3ab

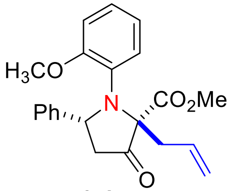

3ab-(*cis*)

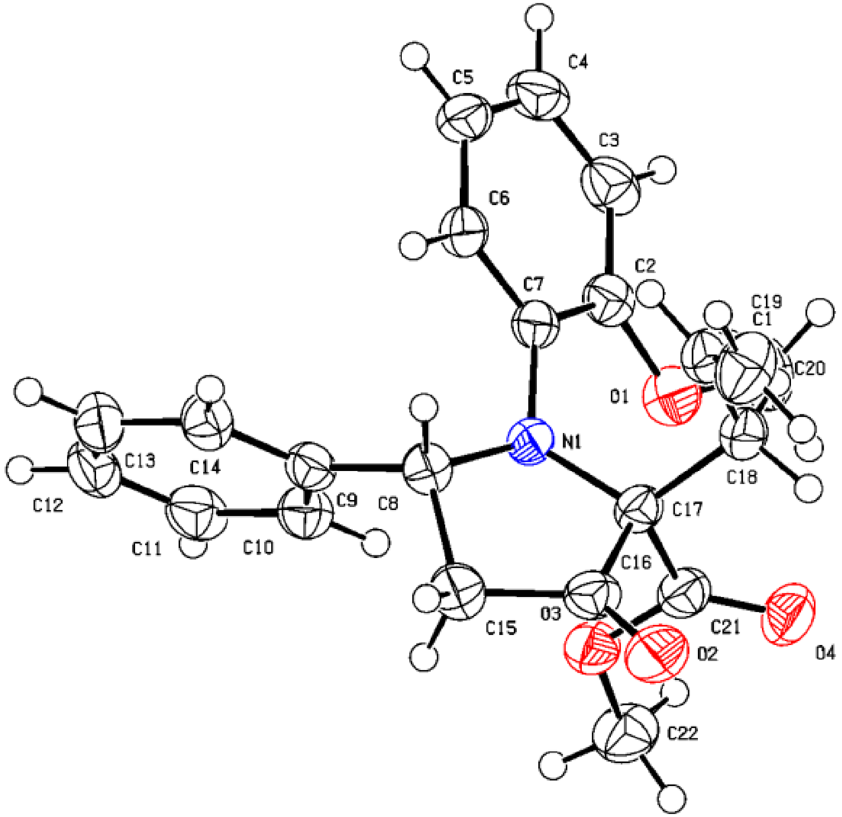

Table S3. Crystal data and structure refinement for compound 3ab-(*cis*)

Bond precision:	C-C = 0.0025 A		Wavelength=1.54178
Cell:	a=14.5890(4)	b=8.7443(2)	c=16.3333(4)
	alpha=90	beta=111.370(2)	gamma=90
Temperature:	173 K		

	Calculated	Reported
Volume	1940.39(9)	1940.39(9)
Space group	P 21/n	P 1 21/n 1
Hall group	-P 2yn	-P 2yn
Moiety formula	C22 H23 N O4	C22 H23 N O4
Sum formula	C22 H23 N O4	C22 H23 N O4
Mr	365.41	365.41
Dx,g cm-3	1.251	1.251
Z	4	4
Mu (mm-1)	0.697	0.697
F000	776.0	776.0
F000’	778.40	
h,k,lmax	18,10,20	17,10,19
Nref	3812	3785
Tmin,Tmax	0.913,0.939	0.695,0.754
Tmin’	0.913	

Correction method= # Reported T Limits: Tmin=0.695 Tmax=0.754
AbsCorr = MULTI-SCAN

Data completeness= 0.993 Theta(max)= 72.103

R(reflections)= 0.0438(3077) wR2(reflections)= 0.1036(3785)

S = 1.040 Npar= 246

References

[1] Wang, D.-C.; Cheng, P.-P.; Yang, T.-T.; Wu, P.-P.; Qu, G.-R.; Guo, H.-M., Asymmetric Domino Heck/Dearomatization Reaction of β-Naphthols to Construct Indole–Terpenoid Frameworks. *Organic Letters* **2021,** *23,* 7865-7872.
